# Causal relationship between gut microbiota and immune thrombocytopenia: a Mendelian randomization study of two samples

**DOI:** 10.3389/fmicb.2023.1190866

**Published:** 2023-11-23

**Authors:** Dongmei Guo, Qian Chen, Guojun Wang, ChunPu Li, FinnGen consortium

**Affiliations:** ^1^Department of Hematology, Qilu Hospital (Qingdao), Cheeloo College of Medicine, Shandong University, Shandong, China; ^2^The Affiliated Taian City Central Hospital of Qingdao University, Taian, China; ^3^Centre of Neuro-Encephalology, Taian City Central Hospital, Qingdao University, Shandong, China; ^4^Department of Orthopedics, Qilu Hospital (Qingdao), Cheeloo College of Medicine, Shandong University, Shandong, China; ^5^Department of Orthopedics, The Affiliated Taian City Central Hospital of Qingdao University, Taian, China

**Keywords:** immune thrombocytopenia, ITP, gut microbiota, Mendelian randomization study, the causal relationship

## Abstract

**Background:**

Some observational studies have shown that immune thrombocytopenia (ITP) is highly associated with the alteration-composition of gut microbiota. However, the causality of gut microbiota on ITP has not yet been determined.

**Methods:**

Based on accessible summary statistics of the genome-wide union, the latent connection between ITP and gut microbiota was estimated using bi-directional Mendelian randomization (MR) and multivariable MR (MVMR) analyses. Inverse variance weighted (IVW), weighted median analyses, and MR-Egger regression methods were performed to examine the causal correlation between ITP and the gut microbiota. Several sensitivity analyses verified the MR results. The strength of causal relationships was evaluated using the MR-Steiger test. MVMR analysis was undertaken to test the independent causal effect. MR analyses of reverse direction were made to exclude the potential of reverse correlations. Finally, GO enrichment analyses were carried out to explore the biological functions.

**Results:**

After FDR adjustment, two microbial taxa were identified to be causally associated with ITP (*P*_*FDR*_ < 0.10), namely *Alcaligenaceae* (*P*_*FDR*_ = 7.31 × 10^–2^) and *Methanobacteriaceae* (*P*_*FDR*_ = 7.31 × 10^–2^). In addition, eight microbial taxa were considered as potentially causal features under the nominal significance (*P* < 0.05): *Actinobacteria*, *Lachnospiraceae*, *Methanobacteria*, *Bacillales*, *Methanobacteriales*, *Coprococcus2*, *Gordonibacter*, and *Veillonella*. According to the reverse-direction MR study findings, the gut microbiota was not significantly affected by ITP. There was no discernible horizontal pleiotropy or instrument heterogeneity. Finally, GO enrichment analyses showed how the identified microbial taxa participate in ITP through their underlying biological mechanisms.

**Conclusion:**

Several microbial taxa were discovered to be causally linked to ITP in this MR investigation. The findings improve our understanding of the gut microbiome in the risk of ITP.

## Background

Immune thrombocytopenia (ITP) is defined as an acquired autoimmune disorder resulting in bleeding symptoms caused by the destruction of megakaryocytes and the decrease of peripheral blood platelets (counts of platelet < 100 × 10^9^/L) (Anat, 2023; Liu et al., 2023). The annual incidence of ITP was about 2–4/100,000 adults (Lambert and Gernsheimer, 2017). Disregarding common hemorrhage events, patients with ITP often feel fatigued in their daily activities and anxious about the burden of monitoring or treatment (Efficace et al., 2016). The pathogenesis of ITP is intricate. A variety of triggering mechanisms have been identified, such as predisposing factors (Audia et al., 2017), viral infection factors (Provan and Semple, 2022), drug-induced factors (Marini et al., 2022), and vaccine-induced factors (Arepally and Ortel, 2021), as well as those without a clear underlying cause (Swinkels et al., 2018).

The human gut microbiome is considered the biggest and most complicated symbiotic ecosystem, with the host playing a pivotal role in maintaining gut homeostasis (Shi et al., 2017). An imbalanced composition in the gut microbiome has been shown to play a role in autoimmune diseases (Clemente et al., 2018; Beam et al., 2021; Tong et al., 2021; Pianko and Golob, 2022). Several studies have found a connection between gut microbiota and ITP. Borody et al. (2011) reported that the first immune-mediated ITP was successfully reversed using fecal microbiota transplantation (FMT) in 2011. Further studies on probiotics may promote the prevention and treatment of ITP. However, results from previously published studies have been inconsistent. For example, Liu C. et al. (2020) discovered that the compositional change of intestinal microbiota occurred in ITP, with a more significant percentage of *Proteobacteria* and *Bacteroidetes* and a lower ratio of *Firmicutes*/*Bacteroidetes* compared with healthy controls. Yu et al. (2022) obtained results showing that *Actinobacteria* and the *Firmicutes*/*Bacteroidetes* ratio decreased, while Zhang et al. (2020) reported the opposite results about the *Firmicutes*/*Bacteroidetes* ratio. Most of the previous studies about ITP were conducted as case-control studies, in which it was difficult to confirm the causal correlation between the exposure and outcome. Moreover, for observational studies, the relationship between ITP and gut microbiome is vulnerable to confounders such as environment, age, sex, dietary habits, and lifestyle (Wang et al., 2021). Furthermore, it is not easy to prevent these confounding factors from affecting the results of observational studies. The above circumstances restrict us from investigating the causality between ITP and the gut microbiome.

Studies using Mendelian randomization (MR) have widely examined the causal relationship between the gut microbiome and disorders such as autoimmune disorders (Xiang et al., 2021; Xu et al., 2022), metabolic disorders (Sanna et al., 2019; Xu et al., 2021), and psychiatric disorders (Ni et al., 2022). Using MR in studies takes advantage of genetic variants serving as the instrumental variable (IV) to determine the assumption that exposure causally affects the outcome. Confounders cannot affect the link between outcome and genetic variants because genotype variation is randomized among children by their parents. Therefore, we can get a reasonable causal inference from studies using MR (Birney, 2022). This study uses the summary statistics from the genome-wide association study (GWAS) and then assesses the causal relationship between ITP and gut microbiota via a two-sample MR design.

## Materials and method

### Study design

**FIGURE 1:**
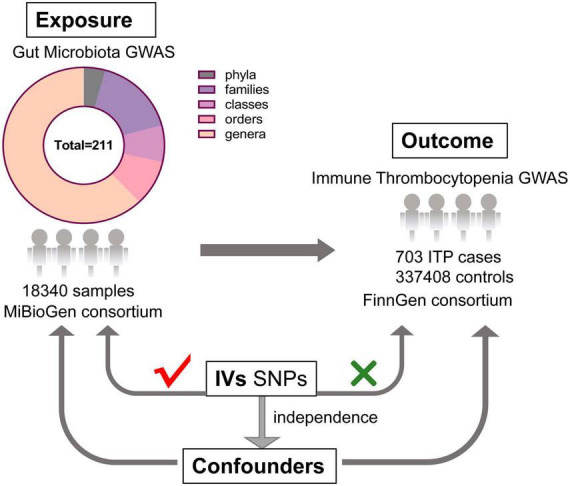
The design of this MR study of the association between gut microbiota and immune thrombocytopenia disease (GWAS, genome-wide association study; IVs, instrumental variables; SNPs, single nucleotide polymorphisms).

As shown in Figure 1, this study aims to reveal the causal effect of gut microbiota and ITP based on two-sample MR approach (Bowden and Holmes, 2019).

### Data sources

GWAS comprises 18,340 persons from 24 cohorts, of various ethnicities, a large amount of whom are of European descent, which produced the most extensively published GWAS summary data for gut microbiota (Davey Smith and Hemani, 2014; Kurilshikov et al., 2021). As Kurilshikov et al. (2021) described, after post-sequencing quality control, standardized 16S rRNA processing pipelines were implemented for all participating cohorts, and the taxonomic resolution was truncated to the genus level. In quantitative microbiome signature loci (mbQTL) mapping, adequate sample sizes of at least 3,000 samples and the presence in no less than three studies were used as study-wide cutoffs, while taxa with less than 10% of representatives present in each participating cohort were also discarded.

To ensure no overlap between the sample and the exposure, we sourced the ITP-GWAS summary statistics from the FinnGen consortium’s R8 release (Hartwig et al., 2017). The FinnGen project aims to generate genomic data linked to the national health registry data of 500,000 Finnish individuals enriched for disease endpoints. Demographic information, such as age and gender, of participants in the FinnGen study was provided. We set the first 10 main components during analysis: gender, age, and genotyping batch. For our analysis, ITP-GWAS included 703 ITP cases and 337,408 controls. Table 1 shows the ITP and GWAS summary of ITP and GWAS data.

**TABLE 1:** Information of ITP and GWAS summary data.

GWAS summary data	Resource	Sample size	Population ancestry	Reference	Data download
ITP	FinnGen consortium	Number of cases: 703 Number of controls: 337,408	European	FinnGen-tutkimushanke vie suomalaiset löytöretkelle genomitietoon (internet). In: FinnGen.	https://risteys.finregistry.fi/endpoints/D3_ITP
Gut microbiota	MiBioGen consortium	18,340 individuals	Predominantly European	Kurilshikov et al., 2021	www.mibiogen.org

### Instrument selection

Our criteria for selecting each feature’s IVs is as follows: (1) candidate single nucleotide polymorphisms (SNPs) with *P* < 1.0 × 10^–5^ statistical significance were selected as being connected with each feature (Hartwig et al., 2017); (2) the linkage disequilibrium (LD) threshold between the SNPs was fixed as *r*^2^ < 0.001 (clumping window size = 10,000 kb) based on the reference panel data from 1,000 Genomes Project European samples (phase 3), to retain the independent SNPs with the lowest *P*-values (Bowden et al., 2015); (3) SNPs with ambiguous alleles (for example, A/C vs. A/G) between the exposure and outcome GWAS were excluded; (4) a sensitivity analysis was conducted to prevent distortion from allele coding or strand orientation, where palindromic SNPs (for example., with G/C or A/T alleles) were taken forward to ruled out.

Weak instrument bias could lead to misleading estimates of causal effects. Thus, the F-statistic was obtained to evaluate the intensity of IVs via the formula $\text{F}=\left(\frac{\text{N}-\text{K}-1}{\text{K}}\right)\left(\frac{{\text{R}}^{2}}{1-{\text{R}}^{2}}\right)$, where the ratio of variance in the phenotype shown by the number of instruments (K), sample size (N), and genetic variants (*R*^2^) was needed (Bowden et al., 2016). If the corresponding F-statistic was larger than 10, this indicated sufficient strength to ensure the validity of IVs. *R*^2^ was worked out via the formula “*R*^2^ = 2 × MAF × (1–MAF) × *beta*^2^,” where “beta” denotes the genetically estimated impact on the exposure, and means denotes the frequency of the minor allele (Verbanck et al., 2018).

### Statistical analysis

We conducted several analyses based on the two-sample MR framework to investigate the potential causal correlation between ITP and gut microbiome, including inverse variance weighted (IVW), weighted median analysis, and MR-Egger regression method. The IVW approach was used as the primary analysis, assuming that all SNPs were valid but vulnerable to horizontal pleiotropy (Abecasis et al., 2010). A weighted median analysis can allow consistent evaluations if up to 50% of the instrumental variables are invalid (Lin et al., 2022). The MR-Egger regression model is similar to the IVW model, except for its additional intercept term for estimating the presence of pleiotropy (Burgess et al., 2013). Additionally, Mendelian Randomization Pleiotropy Residual Sum and Outlier (MR-PRESSO) were taken to identify and adjust for the influence of outliers in the data (Bowden et al., 2015). We also performed a leave-one-out analysis, where each SNP was taken off one at a time while calculating Cochran’s Q-statistic to determine the heterogeneity of IVs to confirm all results were not skewed by a single SNP (Bowden et al., 2016).

If the results of all MR analyses were significant at a statistics level (*P* < 0.05), we speculated that the gut microbiome may be linked to the risk of ITP. The directional causality of the gut microbiome on ITP was evaluated using the MR-Steiger test (Hemani et al., 2017), and MR analysis in the reverse direction was performed. The procedures and settings used were in accordance with those of the forward MR study. If the subsequent four conditions were satisfied, we held the belief that a substantial causal link between gut microbiome and ITP risk existed: (1) a substantial disparity was evident using the IVW method (*P* < 0.05); (2) the outcome estimations of the IVW, weighted median, and MR-Egger methodologies demonstrated congruence; (3) both the MR-Egger intercept test and the MR-PRESSO global test yielded non-significant results (*P* > 0.05); and (4) the MR-Steiger directionality tests indicated TRUE (*P* < 0.05). Multivariable MR (MVMR) analysis was performed to estimate the independent causal relationship between gut microbiota and ITP conditioning on the effects of other exposures. IVW was also the primary analysis method. Moreover, we conducted GO enrichment analysis based on lead SNPs for all identified gut microbial taxa to further study the biological function of gut microbial taxa on the risk of ITP.

Two Sample MR software (version 0.5.6) was used for all analyses. MR-PRESSO (version 1.0) R package was done by the R version 4.2.1 software package. GO enrichment analysis was carried out by the “FUMA” website tool (Watanabe et al., 2017). In order to exclude any potential false positive signal, the false discovery rate (FDR) method was used to adjust for the number of exposures tested under each attribute. *P*_*FDR*_ < 0.1 was set as the significant threshold. Microbial taxa and ITP were considered to have a potential association when *P* < 0.05 but *P*_*FDR*_ ≥ 0.1.

## Results

### MR results

A total of 2,248 SNPs were employed as IVs for 211 bacterial taxa in accordance with the selection criteria for IVs. The bias of weak IVs was eliminated by the F-statistics of the IVs, which ranged from 12.91 to 187.70. Supplementary Table 1 provides additional information regarding the chosen instrumental factors.

**FIGURE 2:**
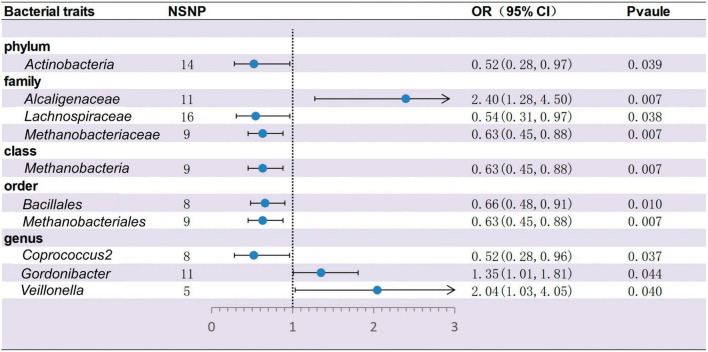
Forest plots for the causal association between gut microbiota and ITP on the IVW method (IVW, inverse variance-weighted; NSNP, numbers of single nucleotide polymorphism; OR, odds ratio; 95% CI, 95% confidence interval).

**FIGURE 3:**
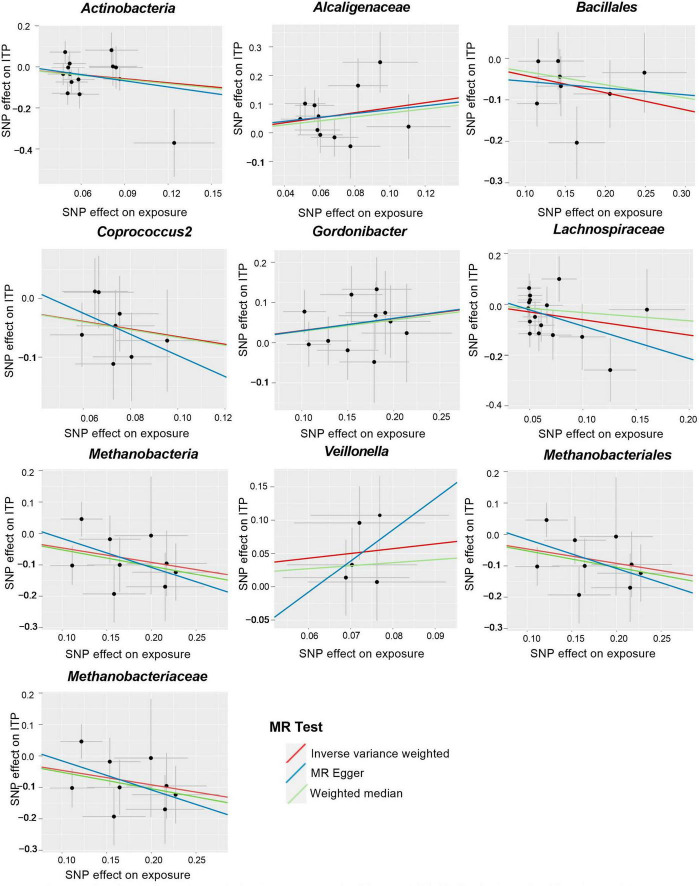
Scatter plots for the causal association between gut microbiota and ITP (SNP, single nucleotide polymorphism; ITP, immune thrombocytopenia).

Based on IVW methods, a total of 10 microbial taxa were identified to be associated with ITP, where one belongs to phyla, three belong to families, one belongs to classes, two belong to orders, and three belong to genera (Figures 2, 3 and Supplementary Table 2). After FDR correction (Supplementary Table 13), two significant taxa were identified. *Alcaligenaceae* (OR = 2.40; 95% CI, 1.28–4.50; *P* = 6.56 × 10^–3^, *P*_*FDR*_ = 7.31 × 10^–2^) had a risk effect on ITP, and *Methanobacteriaceae* (OR = 0.63; 95% CI, 0.45–0.88; *P* = 6.85 × 10^–3^, *P*_*FDR*_ = 7.31 × 10^–2^) is negatively associated with ITP risk. In addition, eight microbial taxa were considered as potentially causal features under the nominal significance (*P* < 0.05). Specifically, *Gordonibacter* (OR = 1.35; 95% CI, 1.01–1.81; *P* = 4.38 × 10^–2^, *P*_*FDR*_ = 9.07 × 10^–1^) and *Veillonella* (OR = 2.04; 95% CI, 1.03–4.05; *P* = 4.04 × 10^–2^, *P*_*FDR*_ = 9.07 × 10^–1^) had a risk effect on ITP, and *Methanobacteria* (OR = 0.63; 95% CI, 0.45–0.88; *P* = 6.85 × 10^–3^, *P*_*FDR*_ = 1.10 × 10^–1^), *Methanobacteriales* (OR = 0.63; 95% CI, 0.45–088; *P* = 6.85 × 10^–3^, *P*_*FDR*_ = 1.02 × 10^–1^), *Lachnospiraceae* (OR = 0.54; 95% CI, 0.31–0.97; *P* = 3.80 × 10^–2^, *P*_*FDR*_ = 3.40 × 10^–1^), *Coprococcus2* (OR = 0.52; 95% CI, 0.28–0.96; *P* = 3.74 × 10^–2^, *P*_*FDR*_ = 9.07 × 10^–1^), *Bacillales* (OR = 0.66; 95% CI, 0.48–0.91; *P* = 1.01 × 10^–2^, *P*_*FDR*_ = 1.02 × 10^–1^), and *Actinobacteria* (OR = 0.52; 95% CI, 0.28–0.97; *P* = 3.89 × 10^–2^, *P*_*FDR*_ = 3.50 × 10^–1^) had a protective effect on ITP.

**FIGURE 4:**
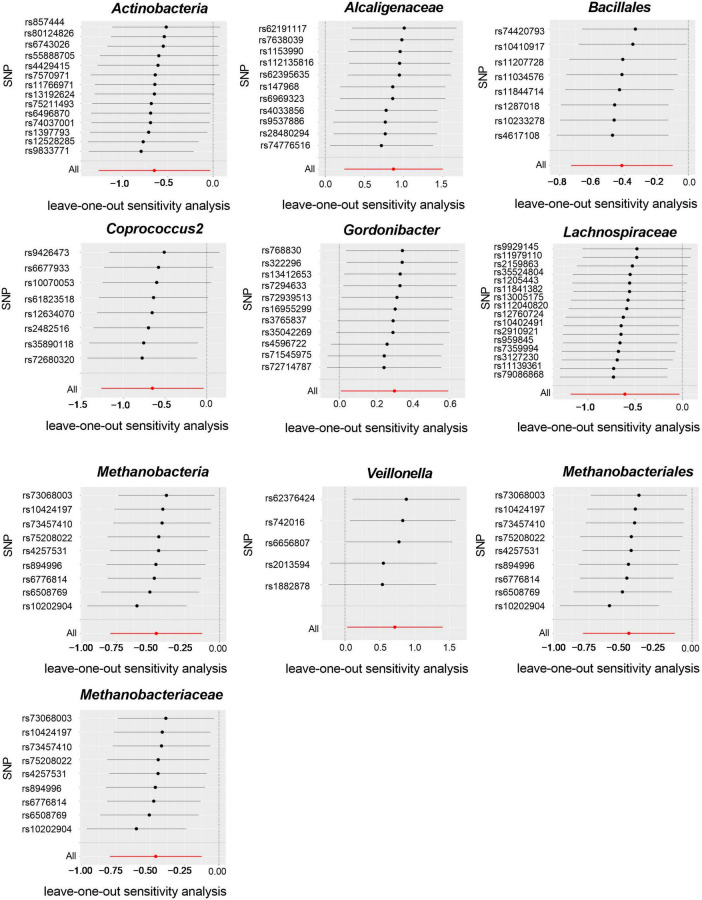
Leave-one-out plots for the causal association between gut microbiota and ITP (SNP, single nucleotide polymorphism).

Cochran’s IVW Q test results showed no evidence of significant heterogeneity in these IVs (Supplementary Table 3). The results of the intercept analysis of the MR-Egger regression likewise suggested that there was no substantial horizontal pleiotropy in either direction (Supplementary Table 4). Further testing of the MR-Egger regression’s accuracy using MR-PRESSO revealed no horizontal pleiotropy (Supplementary Table 5). Also, the leave-one-out results offered additional proof of the data’s reliability (Figure 4). The IVW results were accurate when pleiotropy and heterogeneity were absent. The results of the MR-Steiger directionality tests were TRUE (Supplementary Table 11). Thereby, these bacteria were causally related to ITP.

To eliminate the error in estimating causal effects caused by the potential correlations between exposures, MVMR was performed based on the findings from the univariate analysis at genus, family, and order levels. At the genus level, three independent causal associations were found, including family *Coprococcus2* (OR = 0.55; 95% CI, 0.34–0.89; *P*_*IVW*_ = 1.40 × 10^–2^), *Gordonibacter* (OR = 1.36; 95% CI, 1.10–1.67; *P*_*IVW*_ = 4.0 × 10^–3^), and *Veillonella* (OR = 1.71; 95% CI, 1.05–2.78; *P*_*IVW*_ = 3.0 × 10^–2^). At the family level, we found the independent causal effect in *Alcaligenaceae* (OR = 2.37; 95% CI, 1.20–4.67; *P*_*IVW*_ = 1.3 × 10^–2^). However, the results for suggestive microbial taxa turned out to be insignificant after adjustment, including *Lachnospiraceae* (OR = 0.59; 95% CI, 0.32–1.12; *P*_*IVW*_ = 1.06 × 10^–1^) and *Methanobacteriaceae* (OR = 0.73; 95% CI, 0.53–1.02; *P*_*IVW*_ = 6.7 × 10^–2^). At the order level, the MVMR confirmed the results for *Bacillales* (OR = 0.65; 95% CI, 0.48–0.89; *P*_*IVW*_ = 8.0 × 10^–3^), while the results for *Methanobacteriales* (OR = 0.69; 95% CI, 0.48–1.00; *P*_*IVW*_ = 5.1 × 10^–2^) were not significant. All the results above indicated a potential pleiotropy for gut microbial taxa with ITP at the family and order levels, respectively (Supplementary Table 14).

### Reverse-direction MR analyses

ITP had no significant causal relationship with the other gut microbes (Supplementary Tables 6, 7). Cochran’s IVW Q test revealed no substantial heterogeneity in ITP IVs (Supplementary Table 8). There was no discernible horizontal pleiotropy in the MR-Egger regression intercepted item analysis (Supplementary Table 9) or the MR-PRESSO analysis (Supplementary Table 10).

### FUMA analysis and GO enrichment analysis

**FIGURE 5:**
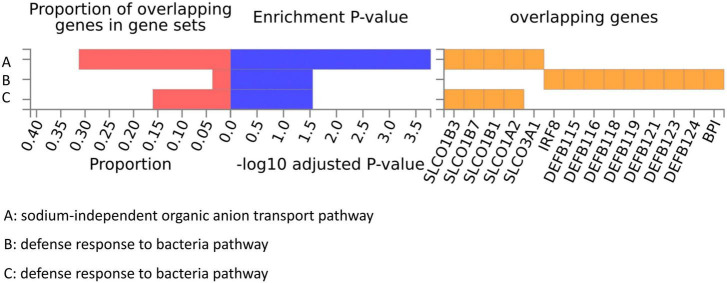
GO pathways from gut microbiota might participate in the ITP risk.

We conducted a functional mapping and annotation (FUMA) analysis to functionally map and annotate the genetic associations to understand better the underlying molecular mechanism between the 10 bacterial genera and ITP risk (Lozupone et al., 2012). Lead SNPs for which *P* < 5 × 10^–6^ and *r*^2^ < 0.1 were identified from the 10 GWAS results (Supplementary Table 12). After SNP-to-genes mapping and annotating the mapped genes in biological contexts, three GO biological processes (“sodium-independent organic anion transport,” “defense response to bacteria,” and “bile acid and bile salt transport”) were observed that could affect ITP (Figure 5).

## Discussion

To the best of our knowledge, this is the first investigation of the causal link between gut microbiome and ITP using publicly accessible genetic datasets. In our study, we utilized the summary statistics of the gut microbiome from the MiBioGen consortium GWAS meta-analysis and the summary statistics of ITP from the FinnGen consortium (R8 released data) to conduct a two-sample MR analysis to find the possible impact of gut microbiota on the risk of ITP. Our MR analysis identified 10 gut microbial taxa as having potential effects on the risk of ITP. The family *Alcaligenaceae*, genus *Gordonibacter*, and genus *Veillonella* could lead to a higher risk of ITP. In contrast, the phylum *Actinobacteria*, order *Bacillales*, genus *Coprococcus2*, family *Methanobacteriaceae*, class *Methanobacteria*, family *Lachnospiraceae*, and order *Methanobacteriales* were linked to a decrease in ITP risk. MVMR indicated a potential pleiotropy at the family and order levels, respectively.

The human intestinal mucosa houses a diverse microbiota population, including over 1,000 species that serve vital roles in maintaining good health (Tremaroli and Bäckhed, 2012; Yu et al., 2021). The gut microbiota primarily comprises *Actinobacteria*, *Bacteroidetes*, *Cyanobacteria*, *Proteobacteria*, *Firmicutes*, *Fusobacteria*, and *Verrucomicrobia* (Lozupone et al., 2012; Adak and Khan, 2019). It is worth noting that an imbalance in gut microbiota can lead to various diseases related to immunology, psychology, and metabolism (Wang and Zhao, 2018; Góralczyk-Bińkowska et al., 2022; Miyauchi et al., 2023). Dysbiosis of the gut microbiome can significantly affect the development of autoimmune conditions such as type 1 diabetes (T1DM) (Murri et al., 2013), Crohn’s disease (CD) (Caparrós et al., 2021), multiple sclerosis (MS) (Pröbstel et al., 2020), inflammatory bowel diseases (IBD) (Parada Venegas et al., 2019), autoimmune hepatitis disease (AHD) (Cheng et al., 2022), asthma (Arrieta et al., 2015), allergies (Liu X. et al., 2020), and psoriasis (Tan et al., 2018). Recent studies have suggested that *Veillonella* and *Gordonibacter* may promote inflammation, while *Lachnospiraceae*, *Actinobacteria*, and *Methanobrevibacter* may have protective effects against autoimmune conditions. The abundance of *Veillonella* increased in AHD (Cheng et al., 2022) and CD patients (Caparrós et al., 2021) compared to healthy controls. Meanwhile, *Gordonibacter* was found to stimulate the release of pro-inflammatory cytokines and damage the epithelial barrier in intestinal epithelium-specific Fut2 deficiency mice (Tang et al., 2021). Conversely, *Lachnospiraceae* was noted to exert anti-inflammatory effects in intestinal epithelial cells and immune cells by producing short-chain fatty acids (SCFAs) (Parada Venegas et al., 2019). *Actinobacteria*, specifically *Bifidobacteria* species, can regulate the immune system’s inflammatory and autoimmune responses by activating regulatory Treg cells (Binda et al., 2018). *Methanobrevibacter*, the predominant anaerobic archaeon enriched among MS gut microbiota, was found to participate in the immunomodulatory process and recruit inflammatory cells (Bang et al., 2014; Zoledziewska, 2019). These studies corroborate the findings of our MR study. However, further research is necessary to understand the specific mechanisms of gut microbiota in autoimmune disease development.

In recent years, advancements in macrogenomics and sequencing technologies have enabled researchers to explore variations in the gut microbiota between ITP patients and healthy individuals (Liu C. et al., 2020; Zhang et al., 2020; Yu et al., 2022). Through our MR study, we found three gut microbiota taxa, *Bacillales*, *Coprococcus2*, and *Lachnospiraceae*, belonging to the phylum *Firmicutes*, consistent with other studies’ findings (Liu C. et al., 2020; Yu et al., 2022). However, at the phylum level, we did not find any causal connections between *Firmicutes* and ITP risk. Another study explored the differences in gut microbiota between children with ITP and healthy individuals, and found a decrease in the proportion of *Actinobacteria* in the phylum level, which aligns with our findings (Li et al., 2023). There are several reasons to explain the different results, including the vulnerability of gut microbiota composition to age, dietary habits, geographical environment, and other factors (Milani et al., 2017). Moreover, the microbiome contains a large variety of taxa, and multiple taxa interacting with each other may ultimately override the role of the phylum. Additionally, although compositional variations of the gut microbiome in ITP patients were observed in previous studies, they could not ensure a causal connection between ITP and gut microbiota. In contrast, our two-sample MR study eliminated the reverse causality for the ITP effects on gut microbiota, providing a more robust understanding of the relationship between the ITP and gut microbiota.

GO enrichment analysis highlighted three biological processes that may be linked to the relationship between the gut microbiota and ITP (“defense response to bacteria,” “bile acid and bile salt transport,” and “sodium-independent organic anion transport.”). Gut microbiome dysbiosis has been linked to various immune-related diseases, but its role in initiating ITP is not yet known. When an imbalance of intestinal flora occurs due to invasive infections, it may activate T cells and B cells to secrete multiple inflammatory factors, which could have a protective or harmful effect on the human body (Lozupone et al., 2012). For instance, chronic exposure to certain infections such as *Helicobacter pylori*, *VZV* (*Varicella-Zoster virus*), *CMV* (*cytomegalovirus*), and *hepatitis C* increases the risk of secondary ITP (LeVine and Brooks, 2019). Studies have shown that treating *H. pylori* infection effectively increases platelet counts in ITP patients, which supports the “defense response to bacteria” finding in our GO enrichment study (Vishnu et al., 2021; Dogan et al., 2022; Takeuchi and Okamoto, 2022). Moreover, the target taxa identified in this study, such as *Actinobacteria*, *Methanobrevibacter*, and *Lachnospiraceae*, activate T cells to promote inflammatory responses, further confirming the “defense response” of gut microbiota in ITP risk (Arrieta et al., 2015; Binda et al., 2018; Tan et al., 2018; Parada Venegas et al., 2019; Zoledziewska, 2019; Liu X. et al., 2020; Tang et al., 2021). Bile acids (BA) produced in the liver and transformed in the intestine are closely associated with the gut microbiota’s bioconversion (Thomas et al., 2008; Malhotra et al., 2019; Vishnu et al., 2021; Dogan et al., 2022). Several studies have shown that BA signaling through farnesoid X receptor (FXR) and TGR5 binding can attenuate pro-inflammatory innate immune responses such as MS (Martín et al., 2010). Additionally, BA-dependent FXR can induce the transcription of various genes involved in intestinal mucosal defense against microbes to control bacterial overgrowth and maintain mucosal integrity in the intestine under physiological conditions (Rizzetto et al., 2018). These findings suggest that ITP may involve “bile acid and bile salt transport” of the gut microbiota. However, the influence of “sodium-independent organic anion transport” in immune-related disorders, especially ITP, has been overlooked by researchers.

There are some advantages to this study. The MR design was used to speculate the causal correlation between ITP and gut microbiota, excluding the deviation of confounders and the inference of reverse causality. With the MiBioGen consortium, the most significant GWAS meta-analysis was applied to gather genetic variants related to the gut microbiota, assuring the correctness of the tools. Horizontal pleiotropy was discovered and eliminated using MR-Egger regression and MR-PRESSO. A two-sample MR study can effectively avoid bias in comparison to observational studies.

Despite its benefits, the MR analysis conducted has a few limitations. Firstly, the analysis only explored the bacterial taxa at the genus level, preventing us from establishing a causal correlation between ITP and gut microbiota at the species level. Secondly, the MR analysis did not allow for subgroup analyses, such as distinguishing between primary (isolate) ITP and secondary ITP, as only summary data was used instead of raw data. Lastly, since the GWAS meta-analysis of gut microbiome data only included individuals of European origin, our MR investigation was unable to assess the non-European population.

## Conclusion

In brief, this two-sample MR study established a causal link between gut microbiota components and ITP. Future clinical and animal studies research is required to fully understand the potential mechanism between gut microbiota and ITP.

## Data availability statement

The datasets presented in this study can be found in online repositories. The names of the repository/repositories and accession number(s) can be found in this article/Supplementary material.

## Author contributions

DG and GW: study design. CL and DG: data collection and data analysis. CL and QC: data interpretation and drafting the manuscript. All authors participated in and define the final version of this manuscript, responsible for the integrity of data analysis, and approved the submitted version.
