# Erratum: Causal relationship between gut microbiota and immune thrombocytopenia: a Mendelian randomization study of two samples

**DOI:** 10.3389/fmicb.2023.1355276

**Published:** 2024-01-05

**Authors:** Frontiers Production Office

**Affiliations:** Frontiers Media SA, Lausanne, Switzerland

**Keywords:** immune thrombocytopenia, ITP, gut microbiota, Mendelian randomization study, the causal relationship

Due to a production error, the affiliation of author “Chunpu Li” was listed incorrectly. The correct affiliations can be found below:

Dongmei Guo^1, 2†^, Qian Chen^2, 3†^, Guojun Wang^2, 3*^, ChunPu Li^4, 5*^ and FinnGen consortium

^1^Department of Hematology, Qilu Hospital (Qingdao), Cheeloo College of Medicine, Shandong University, Shandong, China

^2^The Affiliated Taian City Central Hospital of Qingdao University, Taian, China

^3^Centre of Neuro-Encephalology, Taian City Central Hospital, Qingdao University, Shandong, China

^4^Department of Orthopedics, Qilu Hospital (Qingdao), Cheeloo College of Medicine, Shandong University, Shandong, China

^5^Department of Orthopedics, The Affiliated Taian City Central Hospital of Qingdao University, Taian, China

The publisher apologizes for this mistake. The original article has been updated.

