# Supplementary material for: Causal relationship between gut microbiota and immune thrombocytopenia: a Mendelian randomization study of two samples
Source: Front Microbiol. 2023 Nov 23;14:1190866. doi: 10.3389/fmicb.2023.1190866 (PMC10702357; doi:10.3389/fmicb.2023.1190866)
Supplement: Supplementary file 1 [file Data_Sheet_1.PDF]

Supplementary Tables

Table S1 Instrumental variables used in MR analysis of the association between gut microbiota and ITP.

Bacterial taxa (exposure)	SNP	effect allele	other allele	MAF	Exposure (Bacteria)			Outcome (ITP)			SNP_F
					Beta	Se	Pval	Beta	Se	Pval	
Actinobacteria	rs11655079	T	C	0.250	-0.056	0.012	5.92512E-06	0.035	0.069	0.608	21.614
Actinobacteria	rs11745923	G	T	0.397	0.056	0.012	1.58131E-06	-0.048	0.055	0.378	27.937
Actinobacteria	rs12049045	A	G	0.382	0.051	0.011	8.62622E-06	-0.042	0.055	0.450	22.581
Actinobacteria	rs134366	G	A	0.060	0.112	0.024	1.50428E-06	0.019	0.104	0.858	25.765
Actinobacteria	rs1376754	G	A	0.470	0.051	0.011	6.71385E-06	-0.039	0.054	0.471	23.724
Actinobacteria	rs1515761	T	C	0.133	0.076	0.017	4.96362E-06	-0.043	0.101	0.670	24.641
Actinobacteria	rs182549	C	T	0.491	0.111	0.012	3.79006E-20	-0.077	0.055	0.163	114.646
Actinobacteria	rs4945008	A	G	0.300	0.054	0.012	5.39237E-06	0.025	0.055	0.644	22.557
Actinobacteria	rs6660520	A	G	0.215	0.071	0.013	1.10652E-07	-0.102	0.060	0.090	31.356
Actinobacteria	rs72767435	T	C	0.068	-0.126	0.027	2.56804E-06	-0.068	0.118	0.565	36.980
Actinobacteria	rs7322849	T	C	0.096	0.094	0.019	6.20794E-07	0.145	0.093	0.121	28.542
Actinobacteria	rs80083040	T	G	0.052	0.156	0.035	8.62105E-06	-0.032	0.130	0.804	43.742
Actinobacteria	rs857444	C	T	0.361	0.051	0.012	8.92233E-06	-0.129	0.056	0.020	21.988
Actinobacteria	rs961091	G	A	0.433	0.050	0.011	8.67593E-06	0.023	0.055	0.684	22.657
Alphaproteobacteria	rs140912403	C	T	0.065	-0.161	0.032	6.20363E-07	0.083	0.115	0.468	57.395
Alphaproteobacteria	rs34569731	A	G	0.335	0.071	0.016	7.37916E-06	-0.011	0.056	0.848	40.716
Alphaproteobacteria	rs62285697	C	T	0.200	0.081	0.018	9.75933E-06	-0.065	0.062	0.294	38.102
Alphaproteobacteria	rs76784716	A	G	0.072	0.133	0.027	5.09141E-07	0.018	0.085	0.830	43.404

Alphaproteobacteria	rs7960664	G	A	0.151	0.097	0.022	8.84301E-06	0.104	0.095	0.274	44.324
Alphaproteobacteria	rs9813022	A	G	0.302	-0.075	0.015	1.05333E-06	-0.033	0.055	0.553	43.757
Bacilli	rs11110282	A	G	0.068	-0.101	0.022	4.85442E-06	0.063	0.123	0.608	23.701
Bacilli	rs11730038	G	A	0.241	-0.063	0.013	1.96025E-06	0.057	0.059	0.339	26.699
Bacilli	rs12797734	T	C	0.253	0.057	0.013	7.21166E-06	0.035	0.063	0.580	22.689
Bacilli	rs13068444	A	G	0.193	0.060	0.014	9.53048E-06	0.017	0.070	0.810	20.642
Bacilli	rs1595463	C	A	0.487	0.048	0.011	7.9738E-06	0.067	0.054	0.211	20.860
Bacilli	rs28564647	T	G	0.206	-0.061	0.014	7.81199E-06	-0.053	0.072	0.463	22.556
Bacilli	rs2952251	A	G	0.253	-0.060	0.012	1.07895E-06	-0.020	0.064	0.755	24.860
Bacilli	rs34989881	A	G	0.066	0.111	0.025	6.54586E-06	0.095	0.126	0.447	27.769
Bacilli	rs35344081	G	A	0.241	0.062	0.013	1.01498E-06	-0.026	0.061	0.663	25.756
Bacilli	rs4028634	T	C	0.425	0.052	0.011	2.2076E-06	-0.003	0.056	0.953	24.331
Bacilli	rs4459992	T	C	0.320	0.054	0.012	4.29836E-06	0.105	0.057	0.065	22.948
Bacilli	rs57872228	C	T	0.171	-0.071	0.015	9.21977E-07	-0.120	0.083	0.148	26.601
Bacilli	rs694949	A	G	0.122	-0.081	0.018	7.59585E-06	0.088	0.091	0.333	25.960
Bacilli	rs74663707	C	T	0.073	0.098	0.022	8.45686E-06	0.002	0.110	0.986	23.706
Bacilli	rs7666190	C	A	0.063	-0.104	0.025	8.46829E-06	0.113	0.084	0.176	23.328
Bacilli	rs77558518	A	G	0.065	-0.107	0.022	1.33772E-06	-0.038	0.091	0.677	25.530
Bacilli	rs78938557	T	C	0.068	0.108	0.023	1.06891E-06	0.302	0.160	0.060	27.007
Bacilli	rs9581006	C	T	0.046	0.225	0.047	1.79458E-06	-0.234	0.141	0.097	81.558
Bacteroidia	rs11146701	A	G	0.425	0.047	0.011	7.08228E-06	0.013	0.056	0.813	20.130
Bacteroidia	rs17343978	A	C	0.260	-0.055	0.012	8.35928E-06	-0.081	0.066	0.221	21.552
Bacteroidia	rs2032750	T	C	0.451	-0.051	0.011	1.91509E-06	-0.113	0.054	0.035	23.504
Bacteroidia	rs4916508	G	A	0.435	-0.047	0.011	8.47116E-06	-0.019	0.054	0.727	19.661
Bacteroidia	rs55773148	G	A	0.053	-0.122	0.024	3.89585E-07	-0.149	0.118	0.208	27.075
Bacteroidia	rs62531359	T	G	0.145	0.066	0.015	9.08665E-06	-0.023	0.070	0.745	19.583

Bacteroidia	rs62575403	C	T	0.044	0.140	0.031	7.06129E-06	-0.158	0.133	0.235	30.114
Bacteroidia	rs72706335	T	C	0.066	-0.222	0.049	7.65519E-06	-0.005	0.174	0.977	111.884
Bacteroidia	rs73975615	G	A	0.011	-0.207	0.044	1.21616E-06	0.065	0.316	0.837	16.962
Bacteroidia	rs7631304	G	A	0.170	-0.065	0.013	8.36781E-07	-0.036	0.076	0.631	21.606
Bacteroidia	rs79585701	A	C	0.129	0.065	0.015	9.98589E-06	0.131	0.077	0.088	17.280
Bacteroidia	rs929878	C	T	0.245	-0.055	0.012	4.73377E-06	0.045	0.065	0.495	20.407
Betaproteobacteria	rs11128180	A	G	0.224	0.059	0.013	3.67144E-06	-0.030	0.063	0.639	22.420
Betaproteobacteria	rs1511453	A	G	0.064	0.092	0.020	4.75765E-06	-0.323	0.124	0.009	18.647
Betaproteobacteria	rs1928341	A	G	0.422	0.053	0.011	2.01529E-06	-0.052	0.054	0.344	24.737
Betaproteobacteria	rs2321387	G	A	0.530	-0.049	0.011	5.80036E-06	-0.096	0.054	0.074	22.377
Betaproteobacteria	rs2613606	C	T	0.394	-0.051	0.011	2.19817E-06	0.147	0.053	0.006	23.076
Betaproteobacteria	rs320161	A	G	0.275	0.057	0.013	7.33242E-06	0.070	0.063	0.268	23.996
Betaproteobacteria	rs4033856	C	T	0.129	0.083	0.017	5.17166E-07	0.165	0.095	0.083	28.637
Betaproteobacteria	rs6087811	T	G	0.082	-0.098	0.020	7.44305E-07	0.020	0.090	0.821	26.307
Betaproteobacteria	rs62395635	T	C	0.071	0.110	0.024	2.94091E-06	0.021	0.113	0.850	29.028
Betaproteobacteria	rs75242906	C	T	0.054	-0.121	0.028	9.27278E-06	0.011	0.098	0.913	27.365
Clostridia	rs10774377	A	G	0.334	0.053	0.011	3.24401E-06	-0.138	0.054	0.011	22.651
Clostridia	rs112334273	G	A	0.246	0.064	0.013	3.81407E-07	0.012	0.060	0.845	27.956
Clostridia	rs13105690	T	C	0.267	-0.053	0.012	8.7779E-06	-0.025	0.060	0.678	20.135
Clostridia	rs13179700	T	C	0.355	0.051	0.011	3.37083E-06	0.009	0.057	0.873	22.033
Clostridia	rs1842454	G	A	0.207	-0.055	0.013	8.72023E-06	-0.035	0.069	0.608	18.032
Clostridia	rs2273429	A	G	0.107	-0.072	0.015	4.52136E-06	-0.137	0.087	0.118	18.450
Clostridia	rs6797343	G	T	0.174	0.059	0.013	9.35647E-06	0.055	0.068	0.422	18.510
Clostridia	rs6814436	T	C	0.132	0.074	0.015	9.65098E-07	-0.003	0.077	0.967	23.069
Clostridia	rs6815608	T	C	0.085	0.104	0.021	4.01857E-07	0.012	0.075	0.873	30.623
Clostridia	rs72738886	T	C	0.078	0.087	0.019	8.24241E-06	0.098	0.101	0.334	19.693

Clostridia	rs72915163	T	C	0.265	-0.058	0.012	1.34295E-06	0.011	0.066	0.871	24.171
Coriobacteriia	rs11073596	T	G	0.340	0.051	0.011	8.14449E-06	0.062	0.055	0.267	21.449
Coriobacteriia	rs11250875	T	C	0.209	0.061	0.013	4.82844E-06	0.059	0.065	0.362	22.380
Coriobacteriia	rs11656361	A	C	0.129	0.077	0.018	8.02017E-06	-0.049	0.069	0.476	24.690
Coriobacteriia	rs12974142	G	A	0.108	0.079	0.018	8.51232E-06	-0.057	0.105	0.588	22.120
Coriobacteriia	rs13307134	C	T	0.236	0.057	0.013	7.79931E-06	-0.089	0.072	0.216	21.161
Coriobacteriia	rs1397793	G	A	0.386	-0.050	0.011	9.77381E-06	-0.016	0.059	0.785	21.651
Coriobacteriia	rs1816223	A	G	0.211	-0.059	0.013	4.84114E-06	0.060	0.067	0.374	20.988
Coriobacteriia	rs240104	T	C	0.245	-0.060	0.013	1.51603E-06	-0.062	0.060	0.295	24.702
Coriobacteriia	rs2442778	G	A	0.046	-0.116	0.026	9.02703E-06	0.035	0.122	0.772	21.700
Coriobacteriia	rs3025411	A	G	0.104	0.093	0.021	8.26912E-06	-0.043	0.088	0.623	29.487
Coriobacteriia	rs34739816	G	T	0.082	0.097	0.021	3.88358E-06	-0.142	0.114	0.215	25.603
Coriobacteriia	rs67561917	A	G	0.142	-0.071	0.015	5.39129E-06	-0.007	0.070	0.924	22.845
Coriobacteriia	rs719099	A	G	0.136	0.078	0.016	5.43317E-07	-0.017	0.090	0.855	26.182
Coriobacteriia	rs8010111	G	A	0.065	-0.103	0.023	6.89624E-06	-0.149	0.097	0.123	23.715
Deltaproteobacteria	rs1035691	A	G	0.338	-0.055	0.012	9.65172E-06	-0.016	0.055	0.777	25.018
Deltaproteobacteria	rs11599763	T	C	0.390	-0.054	0.012	3.94123E-06	-0.043	0.055	0.439	25.871
Deltaproteobacteria	rs17084793	G	A	0.161	-0.071	0.016	5.68634E-06	0.046	0.076	0.547	25.062
Deltaproteobacteria	rs17791387	A	G	0.141	-0.074	0.015	1.60223E-06	0.137	0.091	0.135	24.115
Deltaproteobacteria	rs2692012	A	G	0.064	0.110	0.025	3.14186E-06	-0.164	0.118	0.165	26.629
Deltaproteobacteria	rs2838334	G	A	0.323	0.056	0.012	5.44551E-06	0.116	0.056	0.040	25.345
Deltaproteobacteria	rs3935584	C	T	0.496	-0.052	0.012	7.50121E-06	-0.066	0.054	0.220	25.159
Deltaproteobacteria	rs4506934	C	T	0.112	-0.094	0.020	3.58887E-06	0.014	0.083	0.863	32.123
Deltaproteobacteria	rs55744759	A	G	0.116	-0.078	0.017	7.3137E-06	-0.086	0.084	0.307	22.943
Deltaproteobacteria	rs6058181	C	T	0.123	0.083	0.017	3.39554E-07	-0.056	0.073	0.438	27.071
Deltaproteobacteria	rs62020470	A	G	0.259	-0.059	0.013	4.85084E-06	-0.161	0.070	0.021	24.179

Deltaproteobacteria	rs9928243	C	A	0.425	-0.054	0.012	5.02081E-06	0.028	0.054	0.601	26.029
Erysipelotrichia	rs1074800	A	G	0.399	0.049	0.011	6.14583E-06	-0.128	0.054	0.019	21.345
Erysipelotrichia	rs10781552	C	T	0.312	-0.055	0.012	2.33114E-06	-0.055	0.060	0.352	24.043
Erysipelotrichia	rs17530232	A	G	0.077	0.103	0.022	2.79123E-06	-0.198	0.119	0.096	27.557
Erysipelotrichia	rs1884466	C	T	0.451	-0.048	0.011	9.52701E-06	-0.067	0.054	0.211	20.546
Erysipelotrichia	rs2300774	G	A	0.408	0.052	0.011	8.95466E-07	-0.100	0.054	0.062	24.363
Erysipelotrichia	rs290833	T	G	0.369	-0.050	0.011	8.029E-06	-0.007	0.054	0.897	21.145
Erysipelotrichia	rs35161940	T	C	0.127	-0.081	0.017	1.8451E-06	-0.023	0.087	0.792	26.493
Erysipelotrichia	rs4078432	C	T	0.181	-0.061	0.013	4.23104E-06	0.032	0.071	0.656	20.173
Erysipelotrichia	rs56970041	T	G	0.111	0.072	0.016	5.40344E-06	0.241	0.111	0.030	19.043
Erysipelotrichia	rs62504403	C	T	0.236	0.068	0.013	1.12265E-07	-0.123	0.068	0.068	30.697
Erysipelotrichia	rs7234058	T	C	0.097	-0.095	0.019	9.12341E-07	0.056	0.093	0.545	28.886
Erysipelotrichia	rs7826267	T	G	0.075	-0.084	0.020	9.28449E-06	-0.152	0.109	0.165	17.843
Erysipelotrichia	rs8003149	C	T	0.282	0.054	0.012	4.08475E-06	-0.011	0.057	0.848	21.597
Gammaproteobacteria	rs11181912	G	A	0.345	-0.058	0.012	9.94762E-07	-0.068	0.057	0.231	27.837
Gammaproteobacteria	rs12404135	A	G	0.117	-0.079	0.017	8.89485E-06	0.175	0.100	0.082	23.661
Gammaproteobacteria	rs2234691	T	C	0.154	0.076	0.017	8.39264E-06	-0.052	0.081	0.518	27.868
Gammaproteobacteria	rs6706173	A	C	0.150	0.074	0.015	1.99495E-07	-0.003	0.078	0.969	25.831
Gammaproteobacteria	rs75101789	C	T	0.156	0.073	0.016	8.78789E-06	0.137	0.095	0.148	25.713
Gammaproteobacteria	rs79795896	A	G	0.054	-0.159	0.035	7.92404E-06	0.043	0.126	0.734	47.364
Gammaproteobacteria	rs9494710	C	T	0.376	-0.055	0.012	4.54607E-06	0.123	0.057	0.032	26.028
Lentisphaeria	rs1002941	G	A	0.279	0.105	0.023	8.14836E-06	-0.007	0.062	0.906	81.795
Lentisphaeria	rs11770843	C	T	0.259	0.109	0.023	1.90729E-06	-0.031	0.057	0.589	84.765

Lentisphaeria	rs17114848	G	A	0.109	0.152	0.032	4.05865E-06	0.059	0.090	0.509	83.280
Lentisphaeria	rs2031282	A	G	0.170	0.122	0.027	4.3826E-06	0.034	0.071	0.630	77.819
Lentisphaeria	rs2825714	A	G	0.157	-0.137	0.029	1.72211E-06	0.050	0.071	0.485	92.161
Lentisphaeria	rs62570196	C	T	0.069	-0.216	0.044	1.07926E-06	0.069	0.132	0.599	110.348
Lentisphaeria	rs72640280	A	G	0.054	0.220	0.049	5.18035E-06	-0.040	0.117	0.734	90.822
Lentisphaeria	rs77599476	A	G	0.066	0.230	0.048	1.86133E-06	-0.090	0.119	0.445	120.007
Melainabacteria	rs10148250	G	A	0.456	0.086	0.019	8.67135E-06	0.070	0.056	0.211	67.848
Melainabacteria	rs10738747	G	A	0.496	0.081	0.018	9.95804E-06	0.033	0.054	0.548	61.045
Melainabacteria	rs11150282	T	C	0.321	0.099	0.020	6.02892E-07	0.017	0.056	0.764	78.615
Melainabacteria	rs113884518	T	C	0.049	-0.205	0.045	8.0567E-06	-0.236	0.171	0.168	71.933
Melainabacteria	rs28678345	T	C	0.047	0.215	0.047	6.69088E-06	0.034	0.125	0.788	75.680
Melainabacteria	rs367480	G	A	0.427	-0.084	0.019	8.19925E-06	0.070	0.056	0.210	63.185
Melainabacteria	rs4129395	G	A	0.426	0.090	0.019	1.48056E-06	0.009	0.054	0.862	72.329
Melainabacteria	rs789069	A	C	0.172	-0.104	0.023	6.84635E-06	0.027	0.077	0.728	56.167
Melainabacteria	rs79790072	T	C	0.057	0.227	0.049	3.29009E-06	-0.119	0.156	0.446	101.390
Melainabacteria	rs9864379	T	C	0.113	-0.160	0.029	5.3606E-08	-0.143	0.076	0.060	94.497
Methanobacteria	rs10202904	T	G	0.467	-0.122	0.024	3.0143E-07	-0.046	0.055	0.401	136.342
Methanobacteria	rs10424197	G	A	0.325	-0.111	0.025	9.27887E-06	0.102	0.062	0.098	100.167
Methanobacteria	rs4257531	G	A	0.114	0.164	0.036	7.44344E-06	-0.100	0.087	0.252	101.024
Methanobacteria	rs6508769	T	C	0.165	0.154	0.034	8.22529E-06	-0.018	0.075	0.808	119.869
Methanobacteria	rs6776814	T	C	0.090	-0.200	0.041	1.63062E-06	0.007	0.188	0.971	119.808
Methanobacteria	rs73068003	G	T	0.122	-0.158	0.035	8.44583E-06	0.193	0.090	0.033	98.987
Methanobacteria	rs73457410	A	G	0.067	0.215	0.044	1.40855E-06	-0.170	0.109	0.118	106.337
Methanobacteria	rs75208022	C	T	0.077	-0.227	0.049	5.92103E-06	0.123	0.091	0.176	134.787
Methanobacteria	rs894996	C	A	0.072	0.217	0.045	1.87557E-06	-0.096	0.105	0.360	115.524
Mollicutes	rs10108398	G	A	0.291	0.077	0.015	1.09E-06	-0.001	0.060	0.993	44.901

Mollicutes	rs11890098	A	G	0.256	0.074	0.015	9.56571E-07	-0.057	0.060	0.339	38.739
Mollicutes	rs12566890	T	G	0.095	-0.101	0.023	3.65095E-06	-0.105	0.080	0.188	32.439
Mollicutes	rs17214486	C	A	0.402	0.061	0.014	6.60866E-06	-0.105	0.057	0.068	32.844
Mollicutes	rs2464826	A	C	0.126	0.094	0.021	8.39505E-06	0.032	0.085	0.705	36.130
Mollicutes	rs28537087	G	A	0.164	0.082	0.019	8.07399E-06	-0.071	0.062	0.258	33.946
Mollicutes	rs3768491	A	G	0.286	-0.068	0.015	4.23352E-06	-0.050	0.059	0.397	34.826
Mollicutes	rs4885016	T	C	0.157	-0.082	0.018	7.26986E-06	0.039	0.079	0.626	32.683
Mollicutes	rs6043847	T	C	0.070	-0.115	0.025	4.55476E-06	-0.102	0.114	0.373	31.429
Mollicutes	rs72901605	T	C	0.186	-0.084	0.018	3.2579E-06	0.029	0.084	0.728	39.422
Mollicutes	rs74603314	T	C	0.046	0.222	0.046	1.55689E-06	-0.026	0.137	0.850	78.912
Mollicutes	rs78169027	A	G	0.108	-0.108	0.024	5.8761E-06	0.067	0.113	0.552	41.623
Negativicutes	rs1135612	G	A	0.245	0.053	0.012	9.26307E-06	0.065	0.066	0.328	19.035
Negativicutes	rs13086907	G	A	0.200	0.063	0.013	1.95488E-06	-0.011	0.065	0.868	22.957
Negativicutes	rs1643968	T	C	0.280	-0.057	0.011	4.14596E-07	-0.061	0.056	0.271	23.673
Negativicutes	rs1649999	A	G	0.097	0.075	0.017	7.5812E-06	-0.059	0.091	0.518	18.106
Negativicutes	rs2834062	A	G	0.365	0.049	0.011	8.4392E-06	0.014	0.059	0.813	20.349
Negativicutes	rs4463806	T	C	0.204	-0.054	0.013	7.80662E-06	0.023	0.068	0.738	17.627
Negativicutes	rs4722181	T	G	0.476	0.050	0.011	2.00112E-06	0.003	0.054	0.955	23.019
Negativicutes	rs60274479	T	C	0.223	-0.066	0.013	1.1623E-06	0.002	0.068	0.982	27.665
Negativicutes	rs61249479	A	C	0.088	0.078	0.017	2.95471E-06	0.004	0.074	0.958	17.700
Negativicutes	rs71405394	G	A	0.052	-0.114	0.024	2.16631E-06	0.121	0.106	0.253	23.471
Negativicutes	rs73232831	G	A	0.064	-0.152	0.031	1.87452E-06	-0.003	0.144	0.981	50.490
Negativicutes	rs9423647	G	A	0.515	0.048	0.011	6.06114E-06	-0.026	0.054	0.629	20.970
Verrucomicrobiae	rs111862613	T	C	0.166	0.091	0.020	3.73846E-06	0.024	0.072	0.741	41.865
Verrucomicrobiae	rs117107102	A	G	0.045	0.205	0.043	2.91821E-06	-0.052	0.128	0.686	65.849
Verrucomicrobiae	rs11729256	T	C	0.239	0.075	0.015	6.7309E-07	0.075	0.071	0.288	37.535

Verrucomicrobiae	rs12908520	G	A	0.456	0.062	0.013	2.17163E-06	0.032	0.054	0.558	34.922
Verrucomicrobiae	rs2602429	C	T	0.222	0.075	0.016	2.58297E-06	0.007	0.061	0.903	35.366
Verrucomicrobiae	rs4242783	G	A	0.287	0.069	0.015	2.63612E-06	-0.100	0.060	0.093	35.750
Verrucomicrobiae	rs4936098	A	G	0.340	0.065	0.014	1.12221E-06	-0.013	0.056	0.815	34.714
Verrucomicrobiae	rs61779207	G	A	0.178	-0.076	0.017	6.72285E-06	0.000	0.065	0.995	30.896
Verrucomicrobiae	rs74542928	T	C	0.079	0.112	0.024	1.63222E-06	0.209	0.125	0.093	33.438
Verrucomicrobiae	rs9349825	A	G	0.279	-0.070	0.015	2.53687E-06	0.046	0.068	0.502	36.666
Verrucomicrobiae	rs941682	G	A	0.311	-0.063	0.014	9.61003E-06	0.095	0.060	0.112	31.393
Acidaminococcaceae	rs262812	T	C	0.266	-0.066	0.014	3.24906E-06	0.061	0.058	0.296	30.968
Acidaminococcaceae	rs2933324	A	G	0.254	-0.066	0.014	2.23676E-06	-0.038	0.064	0.549	30.518
Acidaminococcaceae	rs45497800	T	C	0.083	-0.118	0.026	5.86428E-06	0.023	0.077	0.767	38.647
Acidaminococcaceae	rs6589457	A	G	0.055	0.166	0.035	2.31508E-06	-0.122	0.124	0.324	52.351
Acidaminococcaceae	rs6923842	T	C	0.169	-0.080	0.017	2.21303E-06	-0.146	0.084	0.082	32.703
Acidaminococcaceae	rs74540770	G	A	0.065	-0.109	0.024	7.08804E-06	0.065	0.100	0.517	26.354
Acidaminococcaceae	rs78702810	T	C	0.047	-0.144	0.032	9.16468E-06	-0.052	0.088	0.551	33.805
Actinomycetaceae	rs2889192	G	T	0.207	0.089	0.020	3.64417E-06	0.063	0.075	0.401	47.513
Actinomycetaceae	rs34583783	G	T	0.102	0.124	0.026	5.47971E-06	-0.069	0.112	0.537	51.775
Actinomycetaceae	rs35011108	A	G	0.064	0.242	0.050	1.8267E-06	-0.063	0.108	0.558	128.630
Actinomycetaceae	rs4073240	G	A	0.383	0.075	0.016	6.05333E-06	0.124	0.055	0.025	48.547
Alcaligenaceae	rs112135816	T	G	0.147	-0.078	0.017	5.27662E-06	0.047	0.111	0.671	27.683
Alcaligenaceae	rs1153990	A	G	0.221	-0.059	0.013	5.96787E-06	-0.010	0.058	0.868	21.781
Alcaligenaceae	rs147968	C	T	0.441	0.049	0.011	9.12701E-06	0.048	0.054	0.373	21.692
Alcaligenaceae	rs28480294	T	C	0.344	-0.052	0.012	6.606E-06	-0.102	0.056	0.069	22.143

Alcaligenaceae	rs4033856	C	T	0.129	0.082	0.017	1.02902E-06	0.165	0.095	0.083	27.675
Alcaligenaceae	rs62191117	A	G	0.206	0.068	0.013	2.75943E-07	-0.016	0.066	0.808	28.114
Alcaligenaceae	rs62395635	T	C	0.071	0.111	0.024	3.34696E-06	0.021	0.113	0.850	29.476
Alcaligenaceae	rs6969323	A	C	0.252	-0.059	0.013	3.88581E-06	-0.058	0.064	0.367	24.357
Alcaligenaceae	rs74776516	T	G	0.076	-0.094	0.021	6.85049E-06	-0.246	0.106	0.020	22.844
Alcaligenaceae	rs7638039	T	C	0.233	0.060	0.013	2.70479E-06	-0.007	0.062	0.905	23.861
Alcaligenaceae	rs9537886	A	C	0.523	-0.057	0.011	2.35108E-07	-0.096	0.054	0.076	29.869
Bacteroidaceae	rs11585893	A	G	0.148	-0.074	0.015	1.79511E-06	0.037	0.063	0.555	25.425
Bacteroidaceae	rs13207588	A	G	0.233	-0.059	0.013	7.48504E-06	0.024	0.068	0.720	22.976
Bacteroidaceae	rs1340391	T	C	0.192	-0.059	0.013	6.7301E-06	-0.103	0.078	0.189	19.947
Bacteroidaceae	rs17619981	T	G	0.066	0.088	0.019	2.68683E-06	0.016	0.078	0.843	17.465
Bacteroidaceae	rs2023437	T	C	0.124	-0.078	0.017	5.01824E-06	0.116	0.082	0.154	24.466
Bacteroidaceae	rs66710942	C	T	0.416	0.049	0.011	5.86259E-06	-0.027	0.054	0.610	21.240
Bacteroidaceae	rs6795673	C	T	0.412	0.054	0.011	3.37909E-07	0.011	0.054	0.833	25.798
Bacteroidaceae	rs9507307	C	T	0.190	0.060	0.013	2.1271E-06	-0.072	0.062	0.250	20.638
BacteroidalesS24	rs10872669	A	G	0.107	-0.123	0.028	9.4901E-06	0.164	0.093	0.079	53.409
BacteroidalesS24	rs12748533	G	T	0.297	-0.082	0.017	2.59019E-06	0.103	0.059	0.083	51.795
BacteroidalesS24	rs17043785	T	C	0.060	-0.176	0.035	5.11837E-07	0.036	0.094	0.703	64.033
BacteroidalesS24	rs61508842	T	C	0.122	0.123	0.027	7.82782E-06	0.039	0.095	0.684	59.341
BacteroidalesS24	rs689695	C	A	0.366	0.081	0.017	1.28039E-06	-0.099	0.059	0.093	56.631
BacteroidalesS24	rs738193	T	C	0.355	0.085	0.017	3.82163E-07	-0.082	0.056	0.145	60.485
BacteroidalesS24	rs78609301	A	G	0.217	-0.087	0.020	7.09297E-06	0.012	0.059	0.835	46.923
BacteroidalesS24	rs941000	C	T	0.409	0.085	0.016	3.15557E-07	-0.063	0.056	0.257	64.292
Bifidobacteriaceae	rs10831953	G	A	0.333	0.054	0.012	9.94742E-06	0.035	0.058	0.554	23.568
Bifidobacteriaceae	rs12446429	T	C	0.136	0.081	0.019	8.52886E-06	0.068	0.069	0.324	28.361
Bifidobacteriaceae	rs13020688	G	A	0.353	0.058	0.012	1.57375E-06	0.003	0.058	0.964	28.614

Bifidobacteriaceae	rs182549	C	T	0.491	0.117	0.013	5.942E-20	-0.077	0.055		0.163	126.491
Bifidobacteriaceae	rs4957061	T	C	0.465	0.057	0.012	1.1517E-06	-0.033	0.055		0.546	29.680
Bifidobacteriaceae	rs540489	T	G	0.234	-0.063	0.014	5.36811E-06	-0.038	0.070		0.585	26.307
Bifidobacteriaceae	rs55888705	A	G	0.387	0.054	0.012	8.66208E-06	-0.074	0.059		0.213	25.092
Bifidobacteriaceae	rs6899771	A	G	0.095	-0.091	0.020	7.27995E-06	-0.011	0.089		0.905	26.497
Bifidobacteriaceae	rs7174549	C	T	0.356	0.055	0.012	6.8678E-06	-0.005	0.056		0.923	25.628
Bifidobacteriaceae	rs7322849	T	C	0.096	0.111	0.020	1.74032E-08	0.145	0.093		0.121	39.216
Bifidobacteriaceae	rs857444	C	T	0.361	0.055	0.012	3.82492E-06	-0.129	0.056		0.020	25.994
Christensenellaceae	rs117186816	G	A	0.065	-0.206	0.044	6.51964E-06	-0.065	0.095		0.494	94.971
Christensenellaceae	rs12380890	A	G	0.427	-0.050	0.011	5.78132E-06	-0.026	0.054		0.635	22.826
Christensenellaceae	rs12657403	A	G	0.106	0.078	0.017	5.59276E-06	0.188	0.119		0.114	21.299
Christensenellaceae	rs4076564	G	A	0.039	-0.189	0.039	8.06105E-06	0.016	0.119		0.891	48.795
Christensenellaceae	rs62573205	G	A	0.192	-0.065	0.013	1.48853E-06	-0.052	0.067		0.436	24.409
Christensenellaceae	rs7211194	C	T	0.431	0.049	0.011	9.93998E-06	0.008	0.054		0.874	21.811
Christensenellaceae	rs72706624	G	T	0.101	0.088	0.020	6.3775E-06	0.007	0.087		0.932	26.071
Christensenellaceae	rs77867022	G	T	0.026	-0.160	0.038	8.81983E-06	0.004	0.103		0.966	23.760
Christensenellaceae	rs870002	C	T	0.496	0.049	0.011	6.54323E-06	0.052	0.054		0.334	21.945
Christensenellaceae	rs892686	A	G	0.451	0.051	0.011	4.12367E-06	0.020	0.054		0.716	23.293
Christensenellaceae	rs9608766	T	C	0.148	-0.068	0.015	3.33084E-06	0.006	0.061		0.927	21.556
Clostridiaceae1	rs10875374	C	T	0.448	-0.054	0.012	8.09821E-06	0.077	0.054		0.148	26.172
Clostridiaceae1	rs12186080	G	A	0.161	0.075	0.016	5.33957E-06	0.027	0.072		0.706	27.778
Clostridiaceae1	rs12341505	G	A	0.111	0.081	0.018	4.54486E-06	0.037	0.092		0.691	24.098
Clostridiaceae1	rs2795528	G	A	0.049	-0.181	0.039	3.80626E-06	0.155	0.116		0.180	55.816
Clostridiaceae1	rs2817172	C	T	0.413	0.056	0.012	5.27026E-06	0.033	0.055		0.544	28.253
Clostridiaceae1	rs4723021	T	C	0.058	-0.106	0.024	7.42147E-06	-0.067	0.107		0.531	22.593
Clostridiaceae1	rs550843	T	C	0.149	-0.073	0.017	7.09119E-06	-0.033	0.060		0.585	25.157

Clostridiaceae1	rs56188186	A	G	0.076	0.097	0.022	8.24444E-06	-0.128	0.125	0.309	24.008
Clostridiaceae1	rs62397761	A	G	0.286	0.062	0.014	9.07706E-06	0.050	0.057	0.384	28.463
Clostridiaceae1	rs881532	A	G	0.477	-0.053	0.012	7.89662E-06	-0.071	0.054	0.188	26.034
ClostridialesvadinBB60 group	rs10517600	T	G	0.536	0.063	0.014	6.82768E-06	0.074	0.055	0.177	35.931
ClostridialesvadinBB60 group	rs10904722	C	T	0.281	-0.067	0.015	5.04837E-06	0.059	0.063	0.350	33.577
ClostridialesvadinBB60 group	rs118104867	C	T	0.049	0.214	0.046	3.43598E-06	0.142	0.107	0.184	78.486
ClostridialesvadinBB60 group	rs13409132	A	G	0.045	-0.165	0.035	4.37231E-06	0.111	0.141	0.433	42.955
ClostridialesvadinBB60 group	rs17121075	G	A	0.168	0.077	0.017	7.91417E-06	-0.020	0.065	0.757	30.386
ClostridialesvadinBB60 group	rs2191834	G	T	0.240	0.075	0.016	2.50195E-06	-0.045	0.063	0.476	37.300
ClostridialesvadinBB60 group	rs28691777	C	T	0.072	0.137	0.027	6.95697E-07	-0.024	0.132	0.857	45.963
ClostridialesvadinBB60 group	rs34088226	A	G	0.071	-0.118	0.027	7.66216E-06	0.111	0.115	0.334	33.460
ClostridialesvadinBB60 group	rs55682560	C	T	0.070	-0.132	0.026	4.97034E-07	0.053	0.099	0.596	41.173
ClostridialesvadinBB60 group	rs6588624	G	A	0.471	-0.066	0.014	1.79287E-06	0.068	0.054	0.206	40.175
ClostridialesvadinBB60 group	rs66714985	A	C	0.085	0.117	0.025	4.85334E-06	-0.098	0.088	0.265	38.860
ClostridialesvadinBB60	rs7226487	A	G	0.476	-0.064	0.014	3.58282E-06	0.002	0.054	0.976	37.981

group											
ClostridialesvadinBB60 group	rs7538034	T	G	0.208	-0.079	0.017	2.36706E-06	-0.010	0.073	0.893	37.374
ClostridialesvadinBB60 group	rs7725895	A	G	0.090	-0.116	0.024	3.94356E-06	-0.039	0.083	0.638	40.461
ClostridialesvadinBB60 group	rs989682	A	G	0.282	0.070	0.016	6.8471E-06	0.088	0.064	0.164	36.686
Coriobacteriaceae	rs11073596	T	G	0.340	0.051	0.011	8.14449E-06	0.062	0.055	0.267	21.449
Coriobacteriaceae	rs11250875	T	C	0.209	0.061	0.013	4.82844E-06	0.059	0.065	0.362	22.380
Coriobacteriaceae	rs11656361	A	C	0.129	0.077	0.018	8.02017E-06	-0.049	0.069	0.476	24.690
Coriobacteriaceae	rs12974142	G	A	0.108	0.079	0.018	8.51232E-06	-0.057	0.105	0.588	22.120
Coriobacteriaceae	rs13307134	C	T	0.236	0.057	0.013	7.79931E-06	-0.089	0.072	0.216	21.161
Coriobacteriaceae	rs1397793	G	A	0.386	-0.050	0.011	9.77381E-06	-0.016	0.059	0.785	21.651
Coriobacteriaceae	rs1816223	A	G	0.211	-0.059	0.013	4.84114E-06	0.060	0.067	0.374	20.988
Coriobacteriaceae	rs240104	T	C	0.245	-0.060	0.013	1.51603E-06	-0.062	0.060	0.295	24.702
Coriobacteriaceae	rs2442778	G	A	0.046	-0.116	0.026	9.02703E-06	0.035	0.122	0.772	21.700
Coriobacteriaceae	rs3025411	A	G	0.104	0.093	0.021	8.26912E-06	-0.043	0.088	0.623	29.487
Coriobacteriaceae	rs34739816	G	T	0.082	0.097	0.021	3.88358E-06	-0.142	0.114	0.215	25.603
Coriobacteriaceae	rs67561917	A	G	0.142	-0.071	0.015	5.39129E-06	-0.007	0.070	0.924	22.845
Coriobacteriaceae	rs719099	A	G	0.136	0.078	0.016	5.43317E-07	-0.017	0.090	0.855	26.182
Coriobacteriaceae	rs8010111	G	A	0.065	-0.103	0.023	6.89624E-06	-0.149	0.097	0.123	23.715
Defluviitaleaceae	rs112893842	T	C	0.097	0.111	0.023	2.75032E-06	0.059	0.093	0.524	39.641
Defluviitaleaceae	rs1582238	T	C	0.372	0.080	0.017	1.692E-06	0.049	0.056	0.378	55.254
Defluviitaleaceae	rs17051335	C	T	0.080	-0.134	0.029	4.58117E-06	0.076	0.089	0.391	48.547
Defluviitaleaceae	rs1908593	T	C	0.538	0.070	0.016	7.859E-06	0.043	0.054	0.426	45.067
Defluviitaleaceae	rs4344384	G	T	0.474	0.071	0.016	5.86011E-06	-0.105	0.054	0.050	46.058

Defluviitaleaceae	rs4677103	A	G	0.171	0.098	0.020	9.42189E-07	-0.023	0.071	0.742	49.783
Defluviitaleaceae	rs540220	T	C	0.060	-0.124	0.029	9.48137E-06	0.052	0.094	0.582	31.638
Defluviitaleaceae	rs55658617	T	C	0.070	0.177	0.036	1.40608E-06	0.185	0.144	0.198	74.976
Defluviitaleaceae	rs72731813	C	T	0.082	-0.150	0.029	2.75777E-07	0.087	0.125	0.487	61.776
Defluviitaleaceae	rs9608282	T	G	0.066	0.139	0.030	4.60831E-06	0.070	0.150	0.640	43.548
Defluviitaleaceae	rs9725395	A	G	0.088	-0.138	0.030	3.4137E-06	-0.075	0.085	0.373	56.228
Desulfovibrionaceae	rs11599763	T	C	0.390	-0.056	0.012	2.49704E-06	-0.043	0.055	0.439	26.998
Desulfovibrionaceae	rs17791387	A	G	0.141	-0.073	0.015	2.09678E-06	0.137	0.091	0.135	23.682
Desulfovibrionaceae	rs2692012	A	G	0.064	0.114	0.025	1.5639E-06	-0.164	0.118	0.165	28.546
Desulfovibrionaceae	rs2838334	G	A	0.323	0.057	0.012	3.82375E-06	0.116	0.056	0.040	26.229
Desulfovibrionaceae	rs3935584	C	T	0.496	-0.053	0.012	6.78137E-06	-0.066	0.054	0.220	25.386
Desulfovibrionaceae	rs4506934	C	T	0.112	-0.094	0.020	3.16043E-06	0.014	0.083	0.863	32.597
Desulfovibrionaceae	rs6058181	C	T	0.123	0.083	0.017	2.69912E-07	-0.056	0.073	0.438	27.667
Desulfovibrionaceae	rs72647048	T	C	0.116	-0.077	0.017	9.60885E-06	-0.085	0.084	0.314	22.381
Desulfovibrionaceae	rs9928243	C	A	0.425	-0.054	0.012	4.48478E-06	0.028	0.054	0.601	26.347
Enterobacteriaceae	rs11026530	T	C	0.134	0.082	0.019	9.42684E-06	0.152	0.075	0.044	28.868
Enterobacteriaceae	rs2374342	C	A	0.357	0.058	0.013	4.51618E-06	-0.032	0.054	0.554	28.649
Enterobacteriaceae	rs35673018	G	A	0.094	0.090	0.020	7.63109E-06	0.148	0.094	0.114	25.412
Enterobacteriaceae	rs504442	T	G	0.139	0.084	0.019	5.17092E-06	-0.051	0.088	0.562	31.179
Enterobacteriaceae	rs62210023	A	G	0.352	0.061	0.013	3.13163E-06	-0.029	0.056	0.612	30.846
Enterobacteriaceae	rs78143293	A	G	0.144	-0.085	0.017	1.19564E-06	0.029	0.082	0.725	32.626
Enterobacteriaceae	rs79757635	C	A	0.161	0.076	0.017	9.31857E-06	0.085	0.079	0.281	28.554
Erysipelotrichaceae	rs1074800	A	G	0.399	0.049	0.011	6.14583E-06	-0.128	0.054	0.019	21.345
Erysipelotrichaceae	rs10781552	C	T	0.312	-0.055	0.012	2.33114E-06	-0.055	0.060	0.352	24.043
Erysipelotrichaceae	rs17530232	A	G	0.077	0.103	0.022	2.79123E-06	-0.198	0.119	0.096	27.557
Erysipelotrichaceae	rs1884466	C	T	0.451	-0.048	0.011	9.52701E-06	-0.067	0.054	0.211	20.546

Erysipelotrichaceae	rs2300774	G	A	0.408	0.052	0.011	8.95466E-07	-0.100	0.054	0.062	24.363
Erysipelotrichaceae	rs290833	T	G	0.369	-0.050	0.011	8.029E-06	-0.007	0.054	0.897	21.145
Erysipelotrichaceae	rs35161940	T	C	0.127	-0.081	0.017	1.8451E-06	-0.023	0.087	0.792	26.493
Erysipelotrichaceae	rs4078432	C	T	0.181	-0.061	0.013	4.23104E-06	0.032	0.071	0.656	20.173
Erysipelotrichaceae	rs56970041	T	G	0.111	0.072	0.016	5.40344E-06	0.241	0.111	0.030	19.043
Erysipelotrichaceae	rs62504403	C	T	0.236	0.068	0.013	1.12265E-07	-0.123	0.068	0.068	30.697
Erysipelotrichaceae	rs7234058	T	C	0.097	-0.095	0.019	9.12341E-07	0.056	0.093	0.545	28.886
Erysipelotrichaceae	rs7826267	T	G	0.075	-0.084	0.020	9.28449E-06	-0.152	0.109	0.165	17.843
Erysipelotrichaceae	rs8003149	C	T	0.282	0.054	0.012	4.08475E-06	-0.011	0.057	0.848	21.597
FamilyXI	rs10759623	C	T	0.181	-0.162	0.032	5.78312E-07	-0.047	0.064	0.468	143.945
FamilyXI	rs11547158	A	G	0.120	-0.178	0.037	2.70219E-06	-0.061	0.079	0.439	123.246
FamilyXI	rs17379710	T	C	0.435	-0.116	0.025	3.97107E-06	-0.156	0.054	0.004	122.941
FamilyXI	rs2155352	A	G	0.229	-0.151	0.030	6.63432E-07	-0.055	0.063	0.386	147.745
FamilyXI	rs2156611	T	C	0.492	-0.112	0.025	9.42661E-06	-0.080	0.053	0.136	116.726
FamilyXI	rs3733511	A	G	0.294	0.128	0.027	3.38554E-06	-0.041	0.059	0.485	126.131
FamilyXI	rs488164	T	G	0.384	0.118	0.026	4.79785E-06	-0.002	0.056	0.976	121.557
FamilyXI	rs697771	A	G	0.432	-0.118	0.025	3.19372E-06	0.121	0.054	0.024	125.504
FamilyXIII	rs10404377	C	A	0.423	-0.050	0.011	6.99027E-06	-0.074	0.055	0.181	22.686
FamilyXIII	rs118170811	A	G	0.040	0.152	0.032	1.80357E-06	-0.095	0.154	0.535	32.277
FamilyXIII	rs482905	G	T	0.250	0.060	0.013	3.72345E-06	0.085	0.062	0.167	24.471
FamilyXIII	rs6501525	A	G	0.386	0.056	0.012	1.23726E-06	-0.082	0.057	0.150	27.444
FamilyXIII	rs66753613	G	A	0.205	0.065	0.014	8.08288E-06	-0.031	0.070	0.656	25.359
FamilyXIII	rs6797051	C	T	0.117	-0.081	0.017	4.89281E-06	0.054	0.091	0.554	24.702
FamilyXIII	rs7514702	T	C	0.177	-0.066	0.014	3.91642E-06	0.028	0.075	0.712	23.521
Lachnospiraceae	rs10402491	C	T	0.120	0.066	0.015	7.58351E-06	-0.004	0.072	0.957	17.010
Lachnospiraceae	rs11139361	T	C	0.363	0.049	0.011	4.26262E-06	0.064	0.057	0.265	20.753

Lachnospiraceae	rs112040820	A	G	0.270	0.055	0.012	2.41836E-06	-0.050	0.061	0.416	21.881
Lachnospiraceae	rs11841382	G	T	0.074	-0.072	0.017	9.57773E-06	0.121	0.097	0.210	12.905
Lachnospiraceae	rs11979110	T	C	0.447	-0.050	0.011	1.81843E-06	0.116	0.054	0.031	22.780
Lachnospiraceae	rs1205443	A	G	0.336	0.050	0.011	7.28844E-06	-0.068	0.056	0.227	20.595
Lachnospiraceae	rs12760724	A	C	0.344	-0.048	0.011	7.26734E-06	0.016	0.057	0.778	19.451
Lachnospiraceae	rs13005175	A	G	0.089	0.099	0.022	8.37071E-06	-0.128	0.130	0.324	29.248
Lachnospiraceae	rs2159863	A	G	0.198	-0.059	0.013	3.69557E-06	0.115	0.070	0.101	19.994
Lachnospiraceae	rs2910921	T	C	0.045	0.160	0.036	8.41974E-06	-0.021	0.159	0.895	40.333
Lachnospiraceae	rs3127230	C	T	0.334	-0.050	0.011	6.20325E-06	-0.035	0.058	0.553	20.709
Lachnospiraceae	rs35524804	T	C	0.267	-0.061	0.013	2.44999E-06	0.083	0.065	0.205	26.552
Lachnospiraceae	rs7359994	T	C	0.332	-0.050	0.011	5.35899E-06	-0.017	0.054	0.755	20.614
Lachnospiraceae	rs79086868	T	C	0.109	0.078	0.016	3.01422E-06	0.100	0.089	0.263	21.566
Lachnospiraceae	rs959845	C	T	0.445	-0.049	0.011	5.1695E-06	-0.008	0.055	0.887	22.105
Lachnospiraceae	rs9929145	G	A	0.062	-0.126	0.025	2.83594E-07	0.259	0.125	0.037	33.571
Lactobacillaceae	rs1530559	G	A	0.568	0.077	0.018	9.65254E-06	-0.031	0.054	0.566	53.868
Lactobacillaceae	rs16861661	G	A	0.050	-0.193	0.038	2.70131E-07	0.036	0.109	0.739	64.944
Lactobacillaceae	rs62314653	C	A	0.063	0.177	0.039	6.5919E-06	-0.142	0.114	0.213	67.738
Lactobacillaceae	rs74599091	A	G	0.035	0.192	0.043	7.69522E-06	-0.219	0.197	0.267	45.342
Lactobacillaceae	rs768253	T	G	0.391	-0.079	0.017	3.60763E-06	-0.027	0.054	0.612	54.977
Lactobacillaceae	rs77478751	A	G	0.054	-0.219	0.047	5.95946E-06	-0.027	0.084	0.745	89.971
Lactobacillaceae	rs921925	A	C	0.262	0.100	0.020	5.77195E-07	0.138	0.065	0.034	71.178
Lactobacillaceae	rs9345899	A	G	0.131	-0.124	0.028	9.4459E-06	-0.068	0.087	0.438	64.550
Methanobacteriaceae	rs10202904	T	G	0.467	-0.122	0.024	3.0143E-07	-0.046	0.055	0.401	136.342
Methanobacteriaceae	rs10424197	G	A	0.325	-0.111	0.025	9.27887E-06	0.102	0.062	0.098	100.167
Methanobacteriaceae	rs4257531	G	A	0.114	0.164	0.036	7.44344E-06	-0.100	0.087	0.252	101.024

Methanobacteriaceae	rs6508769	T	C	0.165	0.154	0.034	8.22529E-06	-0.018	0.075	0.808	119.869
Methanobacteriaceae	rs6776814	T	C	0.090	-0.200	0.041	1.63062E-06	0.007	0.188	0.971	119.808
Methanobacteriaceae	rs73068003	G	T	0.122	-0.158	0.035	8.44583E-06	0.193	0.090	0.033	98.987
Methanobacteriaceae	rs73457410	A	G	0.067	0.215	0.044	1.40855E-06	-0.170	0.109	0.118	106.337
Methanobacteriaceae	rs75208022	C	T	0.077	-0.227	0.049	5.92103E-06	0.123	0.091	0.176	134.787
Methanobacteriaceae	rs894996	C	A	0.072	0.217	0.045	1.87557E-06	-0.096	0.105	0.360	115.524
Oxalobacteraceae	rs111966731	T	C	0.072	0.204	0.045	4.56318E-06	-0.069	0.096	0.468	102.055
Oxalobacteraceae	rs11246212	T	C	0.125	0.136	0.029	4.50836E-06	0.064	0.077	0.409	74.677
Oxalobacteraceae	rs12002250	A	C	0.060	0.196	0.045	5.53202E-06	0.328	0.135	0.015	79.428
Oxalobacteraceae	rs1569853	T	C	0.138	-0.140	0.028	7.45179E-07	0.015	0.082	0.860	86.070
Oxalobacteraceae	rs17138946	G	T	0.047	-0.189	0.043	8.09008E-06	-0.017	0.107	0.872	58.787
Oxalobacteraceae	rs36057338	G	T	0.076	0.182	0.040	6.25787E-06	-0.096	0.148	0.517	84.819
Oxalobacteraceae	rs4428215	G	A	0.260	0.126	0.023	4.87931E-08	0.098	0.062	0.110	112.128
Oxalobacteraceae	rs561239	A	G	0.282	0.106	0.024	7.1938E-06	0.056	0.066	0.398	83.121
Oxalobacteraceae	rs6000536	C	T	0.211	-0.118	0.024	7.38718E-07	0.175	0.068	0.010	85.881
Oxalobacteraceae	rs62435498	C	A	0.065	0.182	0.040	7.45831E-06	-0.018	0.089	0.838	73.316
Oxalobacteraceae	rs736744	C	T	0.416	0.106	0.020	1.49416E-07	0.005	0.054	0.930	99.947
Oxalobacteraceae	rs7993559	C	A	0.462	0.092	0.020	5.03729E-06	-0.034	0.054	0.533	77.668
Oxalobacteraceae	rs80330081	A	C	0.066	-0.188	0.042	6.63998E-06	-0.275	0.093	0.003	79.428
Oxalobacteraceae	rs934049	G	A	0.228	0.110	0.024	4.21147E-06	0.068	0.066	0.300	77.994
Pasteurellaceae	rs10965428	C	A	0.080	-0.120	0.026	4.29151E-06	-0.040	0.116	0.732	38.667
Pasteurellaceae	rs111582866	G	A	0.101	-0.114	0.026	7.06627E-06	-0.019	0.095	0.837	43.501
Pasteurellaceae	rs12050685	A	G	0.360	-0.067	0.015	9.18513E-06	-0.028	0.059	0.635	38.110

Pasteurellaceae	rs16970009	A	G	0.044	0.187	0.043	7.31531E-06	0.312	0.190	0.101	53.974
Pasteurellaceae	rs4822728	T	C	0.437	0.069	0.015	4.71847E-06	-0.027	0.053	0.614	42.506
Pasteurellaceae	rs6972479	A	G	0.243	-0.078	0.018	7.74817E-06	0.009	0.066	0.893	41.277
Pasteurellaceae	rs72756943	G	A	0.086	0.140	0.030	3.35218E-06	0.069	0.107	0.522	56.283
Pasteurellaceae	rs73139353	A	C	0.065	-0.223	0.048	8.71314E-06	-0.045	0.095	0.636	110.449
Pasteurellaceae	rs76022354	C	T	0.033	0.243	0.050	1.82858E-06	-0.288	0.123	0.019	68.900
Pasteurellaceae	rs78909003	T	C	0.050	-0.241	0.050	2.04893E-06	-0.125	0.118	0.288	101.298
Pasteurellaceae	rs9382510	C	T	0.246	-0.088	0.017	2.476E-07	0.065	0.061	0.292	52.985
Pasteurellaceae	rs9895850	T	C	0.050	-0.176	0.041	9.08251E-06	0.009	0.132	0.947	54.064
Pasteurellaceae	rs9938097	T	C	0.367	-0.071	0.016	8.22604E-06	0.137	0.055	0.013	43.088
Peptococcaceae	rs117452796	A	G	0.068	-0.258	0.055	3.15296E-06	0.061	0.152	0.689	155.156
Peptococcaceae	rs12144792	C	T	0.359	0.064	0.014	5.82242E-06	0.013	0.055	0.821	35.060
Peptococcaceae	rs12634826	T	G	0.295	-0.074	0.015	1.01109E-06	-0.025	0.056	0.654	41.868
Peptococcaceae	rs12992764	T	G	0.403	0.068	0.014	1.46158E-06	0.012	0.054	0.819	41.389
Peptococcaceae	rs150600492	A	C	0.063	0.136	0.029	2.31305E-06	0.031	0.115	0.784	39.761
Peptococcaceae	rs35703006	G	T	0.216	0.081	0.016	4.94775E-07	-0.047	0.062	0.445	41.067
Peptococcaceae	rs4990837	A	G	0.174	0.091	0.019	1.74194E-06	0.032	0.069	0.637	43.792
Peptococcaceae	rs75430375	C	T	0.065	-0.148	0.032	3.40849E-06	-0.069	0.128	0.591	48.634
Peptococcaceae	rs75898026	A	G	0.183	-0.082	0.017	2.0221E-06	-0.115	0.066	0.082	37.090
Peptostreptococcaceae	rs10805326	G	A	0.278	0.057	0.012	4.03396E-06	0.010	0.059	0.861	23.682
Peptostreptococcaceae	rs117020988	C	T	0.064	0.182	0.037	1.02794E-06	0.041	0.098	0.675	72.967
Peptostreptococcaceae	rs12377846	C	A	0.049	-0.252	0.051	7.26254E-07	0.304	0.162	0.060	108.541
Peptostreptococcacea	rs12986312	T	G	0.322	0.057	0.013	5.76718E-06	-0.105	0.058	0.072	26.223

e											
Peptostreptococcaceae	rs1467258	G	A	0.167	0.073	0.016	7.89804E-06	-0.057	0.069	0.413	26.919
Peptostreptococcaceae	rs1520207	C	T	0.437	0.053	0.011	3.16548E-06	0.033	0.054	0.541	24.972
Peptostreptococcaceae	rs4692811	C	T	0.264	0.064	0.013	4.21123E-07	0.025	0.057	0.660	29.455
Peptostreptococcaceae	rs59865771	C	T	0.281	-0.057	0.013	7.69029E-06	-0.012	0.057	0.835	24.442
Peptostreptococcaceae	rs61841503	G	A	0.137	0.092	0.016	9.8012E-09	0.037	0.080	0.643	36.793
Peptostreptococcaceae	rs6721459	A	G	0.561	0.051	0.011	5.07756E-06	0.050	0.054	0.362	23.512
Peptostreptococcaceae	rs76982728	T	C	0.039	0.124	0.027	3.24058E-06	-0.204	0.185	0.271	21.125
Peptostreptococcaceae	rs77540684	T	G	0.052	0.107	0.025	8.13712E-06	0.038	0.091	0.679	20.504
Peptostreptococcaceae	rs9573937	A	G	0.177	-0.069	0.014	1.70589E-06	-0.020	0.072	0.787	25.730
Porphyromonadaceae	rs10762312	G	A	0.261	-0.052	0.012	8.70354E-06	-0.053	0.058	0.358	19.440
Porphyromonadaceae	rs10858364	G	T	0.276	0.055	0.012	4.31063E-06	-0.053	0.063	0.398	22.467
Porphyromonadaceae	rs17065783	A	G	0.224	-0.059	0.012	1.79351E-06	0.055	0.073	0.450	22.298
Porphyromonadaceae	rs1980561	A	G	0.386	-0.049	0.011	8.94542E-06	0.053	0.054	0.321	20.488

Porphyromonadaceae	rs35233670	T	C	0.441	-0.047	0.011	7.90987E-06	-0.064	0.054	0.234	20.304
Porphyromonadaceae	rs35961441	A	C	0.074	0.092	0.021	8.37393E-06	-0.108	0.139	0.437	20.972
Porphyromonadaceae	rs6953849	A	G	0.147	0.072	0.015	2.4448E-06	-0.058	0.070	0.408	23.762
Porphyromonadaceae	rs7330827	T	C	0.076	-0.104	0.024	8.04891E-06	-0.173	0.115	0.133	27.646
Porphyromonadaceae	rs864093	A	C	0.257	-0.053	0.012	9.598E-06	-0.013	0.065	0.847	19.516
Prevotellaceae	rs12057990	C	T	0.241	0.059	0.013	8.9748E-06	-0.015	0.060	0.808	23.347
Prevotellaceae	rs12118202	T	C	0.190	-0.075	0.015	5.54234E-07	0.047	0.069	0.497	31.961
Prevotellaceae	rs13069367	A	C	0.355	-0.054	0.012	7.39489E-06	0.040	0.055	0.466	24.497
Prevotellaceae	rs148376875	T	G	0.120	0.085	0.018	2.08029E-06	0.063	0.075	0.400	27.766
Prevotellaceae	rs2206482	T	G	0.442	-0.057	0.012	1.30377E-06	0.047	0.055	0.391	29.348
Prevotellaceae	rs2278540	G	A	0.326	0.055	0.012	8.43592E-06	0.035	0.056	0.525	24.789
Prevotellaceae	rs34660375	A	G	0.191	-0.081	0.018	7.40327E-06	0.076	0.076	0.317	36.865
Prevotellaceae	rs3758087	T	C	0.292	0.056	0.012	8.60581E-06	0.090	0.060	0.135	24.177
Prevotellaceae	rs3860225	A	G	0.138	0.084	0.017	5.49682E-07	0.001	0.093	0.995	30.826
Prevotellaceae	rs4493272	T	C	0.471	-0.060	0.012	3.02312E-07	-0.069	0.054	0.198	33.510
Prevotellaceae	rs4685827	T	C	0.224	-0.068	0.015	2.77192E-06	-0.049	0.063	0.431	29.457
Prevotellaceae	rs7252711	A	G	0.127	-0.074	0.016	5.56586E-06	0.096	0.086	0.263	22.377
Prevotellaceae	rs7975087	C	A	0.229	-0.060	0.014	7.59459E-06	-0.104	0.071	0.142	23.425
Prevotellaceae	rs912860	G	A	0.027	-0.229	0.048	9.2992E-07	-0.155	0.178	0.382	50.322
Prevotellaceae	rs9586501	G	A	0.314	0.059	0.013	2.58988E-06	0.058	0.061	0.336	27.798
Prevotellaceae	rs9958960	G	A	0.165	-0.091	0.017	1.06324E-07	-0.021	0.074	0.775	42.385
Rhodospirillaceae	rs1035406	G	A	0.099	-0.114	0.025	5.84451E-06	0.069	0.083	0.406	42.423
Rhodospirillaceae	rs11591293	G	T	0.420	0.074	0.016	2.67148E-06	-0.059	0.054	0.274	49.463
Rhodospirillaceae	rs13336560	C	T	0.456	-0.070	0.016	9.17333E-06	-0.014	0.055	0.795	44.914

Rhodospirillaceae	rs1549633	A	C	0.156	0.100	0.022	4.70392E-06	-0.023	0.084	0.782	48.375
Rhodospirillaceae	rs1923415	A	G	0.137	-0.100	0.023	9.63701E-06	0.025	0.094	0.791	43.452
Rhodospirillaceae	rs3754624	C	T	0.162	0.097	0.020	1.7148E-06	0.003	0.070	0.971	47.102
Rhodospirillaceae	rs4278423	T	C	0.106	0.108	0.024	3.12021E-06	-0.116	0.110	0.294	40.409
Rhodospirillaceae	rs61933850	G	A	0.091	0.165	0.036	7.22582E-06	-0.015	0.079	0.846	82.364
Rhodospirillaceae	rs6679026	T	C	0.107	0.112	0.025	9.94679E-06	-0.072	0.089	0.414	44.198
Rhodospirillaceae	rs7001029	C	T	0.093	0.117	0.026	5.3475E-06	-0.099	0.093	0.288	42.879
Rhodospirillaceae	rs72714493	A	G	0.216	0.082	0.018	7.34924E-06	-0.048	0.075	0.524	41.494
Rhodospirillaceae	rs74354280	C	T	0.153	-0.091	0.020	6.67295E-06	-0.004	0.060	0.953	39.629
Rhodospirillaceae	rs76784716	A	G	0.072	0.136	0.029	1.48988E-06	0.018	0.085	0.830	45.237
Rhodospirillaceae	rs9813022	A	G	0.302	-0.084	0.016	2.53124E-07	-0.033	0.055	0.553	55.054
Rikenellaceae	rs10217435	C	T	0.088	-0.088	0.020	6.5076E-06	-0.089	0.077	0.247	22.900
Rikenellaceae	rs10832801	A	C	0.267	-0.053	0.012	7.50417E-06	0.032	0.060	0.593	20.569
Rikenellaceae	rs1939881	G	A	0.058	-0.106	0.021	5.64373E-07	-0.011	0.116	0.923	22.259
Rikenellaceae	rs2447496	G	A	0.283	-0.055	0.012	6.0861E-06	0.003	0.061	0.955	22.489
Rikenellaceae	rs2833282	G	A	0.128	0.071	0.016	4.31106E-06	0.098	0.075	0.192	20.753
Rikenellaceae	rs36021379	A	G	0.134	-0.066	0.014	7.1952E-06	-0.035	0.073	0.629	18.357
Rikenellaceae	rs4264350	T	C	0.394	-0.053	0.011	1.34832E-06	0.029	0.054	0.591	24.288
Rikenellaceae	rs59663348	G	A	0.195	0.057	0.013	6.12244E-06	-0.019	0.062	0.755	18.770
Rikenellaceae	rs62532512	C	A	0.483	-0.050	0.011	2.76426E-06	-0.003	0.054	0.956	23.317
Rikenellaceae	rs6744030	C	T	0.138	0.070	0.016	9.31514E-06	0.067	0.066	0.313	21.230
Rikenellaceae	rs6837275	A	G	0.254	0.057	0.012	1.447E-06	0.075	0.059	0.199	22.606
Rikenellaceae	rs74474130	T	G	0.066	0.138	0.030	3.61378E-06	-0.180	0.145	0.214	42.756
Rikenellaceae	rs77885767	C	T	0.048	-0.156	0.034	2.84694E-06	0.260	0.127	0.041	40.743
Rikenellaceae	rs9389714	C	T	0.179	-0.064	0.014	8.79066E-06	0.011	0.094	0.908	21.876
Rikenellaceae	rs9578457	G	A	0.057	-0.141	0.032	3.99449E-06	-0.061	0.125	0.624	39.348

Rikenellaceae	rs9603208	G	T	0.103	0.082	0.016	1.91801E-07	0.131	0.089	0.141	22.957
Ruminococcaceae	rs10093275	C	T	0.282	0.053	0.012	5.35228E-06	0.052	0.057	0.365	21.261
Ruminococcaceae	rs10166469	T	C	0.276	-0.053	0.012	8.5216E-06	-0.011	0.061	0.860	20.886
Ruminococcaceae	rs1158100	A	G	0.355	-0.049	0.011	8.60858E-06	-0.040	0.054	0.453	20.227
Ruminococcaceae	rs1612733	T	C	0.060	0.109	0.024	4.22436E-06	0.158	0.118	0.180	24.324
Ruminococcaceae	rs17376049	T	C	0.108	0.085	0.017	7.29619E-07	-0.093	0.086	0.277	25.537
Ruminococcaceae	rs2113833	T	C	0.042	0.169	0.036	1.14466E-06	-0.024	0.149	0.869	42.028
Ruminococcaceae	rs3009418	C	A	0.079	0.093	0.021	8.69024E-06	0.133	0.127	0.296	22.857
Ruminococcaceae	rs55793120	T	C	0.051	0.138	0.027	1.44338E-07	-0.134	0.114	0.238	33.786
Ruminococcaceae	rs56199908	T	C	0.054	-0.199	0.041	1.66414E-06	-0.005	0.118	0.964	74.349
Ruminococcaceae	rs76724913	T	G	0.077	0.090	0.020	9.60494E-06	-0.001	0.091	0.989	21.061
Streptococcaceae	rs10028567	C	T	0.082	-0.093	0.019	3.72497E-06	-0.024	0.082	0.770	23.983
Streptococcaceae	rs11110281	T	C	0.068	-0.131	0.023	1.40137E-08	0.049	0.123	0.691	39.486
Streptococcaceae	rs16950051	A	G	0.059	0.107	0.024	5.33808E-06	0.116	0.109	0.286	23.197
Streptococcaceae	rs2370083	G	T	0.089	-0.084	0.018	4.25666E-06	0.072	0.110	0.514	21.037
Streptococcaceae	rs2952251	A	G	0.253	-0.064	0.013	3.72237E-07	-0.020	0.064	0.755	28.336
Streptococcaceae	rs35344081	G	A	0.241	0.061	0.013	2.63845E-06	-0.026	0.061	0.663	24.916
Streptococcaceae	rs57646748	G	A	0.068	-0.088	0.020	7.88359E-06	0.116	0.138	0.399	17.928
Streptococcaceae	rs6806351	T	C	0.209	-0.062	0.014	6.93793E-06	0.045	0.065	0.484	23.253
Streptococcaceae	rs77968078	G	A	0.057	-0.099	0.022	7.9334E-06	0.081	0.112	0.470	19.363
Streptococcaceae	rs7916711	A	G	0.065	0.096	0.022	6.32732E-06	-0.100	0.078	0.197	20.432
Streptococcaceae	rs957755	T	G	0.191	-0.064	0.014	7.4151E-06	0.033	0.077	0.666	23.411
unknownfamily	rs11150282	T	C	0.321	0.098	0.020	7.36053E-07	0.017	0.056	0.764	77.480
unknownfamily	rs113884518	T	C	0.049	-0.206	0.046	7.73987E-06	-0.236	0.171	0.168	72.326
unknownfamily	rs28678345	T	C	0.047	0.213	0.047	8.05953E-06	0.034	0.125	0.788	74.436
unknownfamily	rs367480	G	A	0.427	-0.084	0.019	7.52418E-06	0.070	0.056	0.210	63.803

unknownfamily	rs4129395	G	A	0.426	0.090	0.019	1.21742E-06	0.009	0.054	0.862	73.644
unknownfamily	rs789069	A	C	0.172	-0.104	0.023	6.50003E-06	0.027	0.077	0.728	56.811
unknownfamily	rs79790072	T	C	0.057	0.226	0.049	3.5381E-06	-0.119	0.156	0.446	100.834
unknownfamily	rs8028558	A	G	0.405	0.083	0.019	9.78236E-06	0.022	0.056	0.697	61.749
unknownfamily	rs9864379	T	C	0.113	-0.161	0.029	4.65873E-08	-0.143	0.076	0.060	95.435
unknownfamily	rs11779863	G	A	0.207	-0.077	0.017	6.68845E-06	0.128	0.074	0.083	35.997
unknownfamily	rs12566890	T	G	0.095	-0.103	0.024	8.11201E-06	-0.105	0.080	0.188	33.728
unknownfamily	rs13100746	C	T	0.410	0.064	0.014	7.28846E-06	-0.078	0.054	0.146	36.282
unknownfamily	rs17235252	T	C	0.094	-0.122	0.026	2.15879E-06	-0.115	0.083	0.166	46.788
unknownfamily	rs3932485	C	T	0.428	0.063	0.014	9.92857E-06	-0.076	0.055	0.168	35.286
unknownfamily	rs515984	T	C	0.174	-0.088	0.019	6.61366E-06	0.195	0.084	0.019	40.489
unknownfamily	rs638542	G	A	0.286	-0.071	0.016	5.17285E-06	-0.054	0.059	0.358	37.421
unknownfamily	rs74603314	T	C	0.046	0.231	0.049	2.27641E-06	-0.026	0.137	0.850	85.576
unknownfamily	rs7706512	G	A	0.504	0.066	0.014	2.27207E-06	0.000	0.054	1.000	39.703
unknownfamily	rs7801843	A	G	0.129	-0.087	0.019	9.46882E-06	-0.060	0.075	0.418	31.249
unknownfamily	rs7853673	G	A	0.514	-0.062	0.014	6.7325E-06	0.020	0.054	0.704	35.789
unknownfamily	rs949341	G	A	0.320	0.066	0.015	7.73282E-06	0.030	0.060	0.620	34.477
unknownfamily	rs11251024	G	A	0.330	0.104	0.021	6.62801E-07	-0.027	0.061	0.654	88.472
unknownfamily	rs11606187	A	G	0.107	-0.155	0.033	3.30586E-06	-0.014	0.077	0.853	84.355
unknownfamily	rs13385922	T	C	0.406	0.093	0.020	3.96829E-06	-0.023	0.056	0.685	76.712
unknownfamily	rs166849	G	A	0.390	0.091	0.020	7.73898E-06	0.015	0.054	0.784	72.688
unknownfamily	rs2172426	C	T	0.447	-0.102	0.020	3.17425E-07	-0.014	0.055	0.800	95.046
unknownfamily	rs267959	A	G	0.343	0.099	0.021	2.61993E-06	0.075	0.060	0.209	81.124
unknownfamily	rs4383094	T	C	0.096	0.149	0.032	4.28134E-06	0.050	0.077	0.519	71.382
unknownfamily	rs60775321	T	C	0.301	-0.096	0.021	7.1046E-06	-0.028	0.059	0.637	71.783
unknownfamily	rs72671304	T	C	0.070	0.172	0.037	3.79578E-06	0.169	0.102	0.096	70.797

unknownfamily	rs7911787	G	T	0.066	-0.223	0.047	3.38824E-06	0.307	0.152	0.044	112.554
unknownfamily	rs8126061	T	C	0.095	-0.159	0.035	7.36334E-06	0.010	0.081	0.902	80.505
unknownfamily	rs9542068	T	C	0.321	0.099	0.022	6.52339E-06	0.060	0.057	0.290	78.810
Veillonellaceae	rs111810795	C	T	0.107	-0.087	0.018	1.72927E-06	-0.036	0.090	0.688	26.474
Veillonellaceae	rs114889439	A	G	0.053	-0.254	0.054	6.19028E-06	-0.040	0.147	0.785	118.713
Veillonellaceae	rs12186441	G	A	0.037	0.208	0.045	4.52857E-06	-0.103	0.132	0.437	56.469
Veillonellaceae	rs12668619	A	G	0.355	0.055	0.012	2.57364E-06	0.122	0.057	0.033	25.715
Veillonellaceae	rs12741784	C	T	0.336	-0.062	0.012	1.28494E-07	0.169	0.059	0.004	31.656
Veillonellaceae	rs1442060	G	A	0.494	-0.051	0.011	4.50675E-06	-0.045	0.054	0.409	24.255
Veillonellaceae	rs1693340	T	C	0.126	0.082	0.018	9.25271E-06	-0.056	0.104	0.593	27.164
Veillonellaceae	rs2175069	A	G	0.386	-0.053	0.011	4.63558E-06	0.141	0.054	0.009	24.143
Veillonellaceae	rs2561116	T	G	0.113	-0.084	0.019	7.88518E-06	-0.166	0.114	0.146	25.798
Veillonellaceae	rs2585520	G	T	0.083	-0.090	0.020	5.26922E-06	0.151	0.138	0.276	22.764
Veillonellaceae	rs4263802	G	A	0.384	0.051	0.011	7.45003E-06	0.083	0.057	0.146	22.535
Veillonellaceae	rs4461038	A	G	0.306	-0.055	0.012	3.73474E-06	-0.025	0.059	0.673	24.028
Veillonellaceae	rs4797169	T	C	0.251	0.059	0.013	4.49346E-06	0.071	0.063	0.259	23.822
Veillonellaceae	rs61264131	A	C	0.050	0.202	0.046	6.74544E-06	-0.031	0.091	0.737	71.213
Veillonellaceae	rs6692542	A	G	0.373	0.053	0.012	8.67975E-06	0.018	0.056	0.744	24.523
Veillonellaceae	rs6909981	C	T	0.202	-0.064	0.014	5.47618E-06	-0.054	0.081	0.507	24.131
Veillonellaceae	rs79535861	A	C	0.089	0.101	0.021	1.57687E-06	0.090	0.093	0.335	30.030
Veillonellaceae	rs9345168	C	A	0.466	0.051	0.011	8.48887E-06	-0.033	0.054	0.538	23.687
Verrucomicrobiaceae	rs111862613	T	C	0.166	0.091	0.020	3.73127E-06	0.024	0.072	0.741	41.872
Verrucomicrobiaceae	rs117107102	A	G	0.045	0.205	0.043	2.91821E-06	-0.052	0.128	0.686	65.849
Verrucomicrobiaceae	rs11729256	T	C	0.239	0.075	0.015	6.7309E-07	0.075	0.071	0.288	37.535

Verrucomicrobiaceae	rs12908520	G	A	0.456	0.062	0.013	2.15138E-06	0.032	0.054	0.558	34.942
Verrucomicrobiaceae	rs2602429	C	T	0.222	0.075	0.016	2.69939E-06	0.007	0.061	0.903	35.240
Verrucomicrobiaceae	rs4242783	G	A	0.287	0.069	0.015	2.75444E-06	-0.100	0.060	0.093	35.615
Verrucomicrobiaceae	rs4936098	A	G	0.340	0.065	0.014	1.12929E-06	-0.013	0.056	0.815	34.698
Verrucomicrobiaceae	rs61779207	G	A	0.178	-0.076	0.017	6.63029E-06	0.000	0.065	0.995	30.937
Verrucomicrobiaceae	rs74542928	T	C	0.079	0.112	0.024	1.64613E-06	0.209	0.125	0.093	33.414
Verrucomicrobiaceae	rs9349825	A	G	0.279	-0.070	0.015	2.51287E-06	0.046	0.068	0.502	36.699
Verrucomicrobiaceae	rs941682	G	A	0.311	-0.063	0.014	9.58114E-06	0.095	0.060	0.112	31.402
Victivallaceae	rs11671100	A	C	0.201	-0.160	0.035	4.08042E-06	0.011	0.067	0.870	151.989
Victivallaceae	rs11764871	G	T	0.251	0.127	0.026	7.49289E-07	-0.018	0.058	0.758	111.915
Victivallaceae	rs2944282	T	C	0.300	-0.124	0.026	1.56601E-06	-0.016	0.060	0.784	119.622
Victivallaceae	rs34962571	A	C	0.083	-0.187	0.042	6.24824E-06	-0.006	0.089	0.946	97.538
Victivallaceae	rs4396289	C	T	0.160	-0.153	0.029	1.53782E-07	-0.077	0.079	0.331	115.994
Victivallaceae	rs61702987	T	C	0.175	0.146	0.030	3.07921E-06	0.033	0.086	0.696	112.790
Victivallaceae	rs62570196	C	T	0.069	-0.246	0.048	2.69852E-07	0.069	0.132	0.599	143.036
Victivallaceae	rs6545794	A	G	0.089	-0.198	0.041	5.97005E-07	0.011	0.082	0.897	116.472
Victivallaceae	rs7077363	G	A	0.164	0.149	0.032	2.83376E-06	-0.067	0.072	0.355	112.232
Victivallaceae	rs7314815	G	A	0.503	0.101	0.023	6.40086E-06	-0.059	0.054	0.274	94.174
Victivallaceae	rs7627405	C	T	0.167	-0.134	0.030	8.1942E-06	-0.055	0.068	0.419	92.291
Actinomyces	rs2715439	C	T	0.454	0.075	0.016	6.27E-06	-0.104	0.054	0.053	50.834
Actinomyces	rs34583783	G	T	0.102	0.127	0.027	4.48529E-06	-0.069	0.112	0.537	54.186
Actinomyces	rs35011108	A	G	0.064	0.233	0.051	6.33832E-06	-0.063	0.108	0.558	118.975
Actinomyces	rs4073240	G	A	0.383	0.075	0.017	7.94274E-06	0.124	0.055	0.025	48.826

Actinomyces	rs4146653	G	A	0.197	0.099	0.021	4.49647E-06	-0.024	0.077	0.756	56.446
Actinomyces	rs71315246	A	G	0.143	-0.097	0.022	9.82978E-06	-0.007	0.078	0.933	42.396
Actinomyces	rs7915461	T	C	0.058	0.188	0.040	5.91984E-06	-0.055	0.107	0.608	70.570
Adlercreutzia	rs11604400	C	T	0.115	-0.103	0.023	9.74463E-06	-0.088	0.088	0.315	39.409
Adlercreutzia	rs13231526	C	A	0.064	0.143	0.031	4.81355E-06	0.000	0.098	0.998	44.924
Adlercreutzia	rs2717140	C	T	0.111	-0.119	0.025	2.04594E-06	0.065	0.090	0.467	51.712
Adlercreutzia	rs55719207	G	A	0.418	-0.070	0.016	9.60505E-06	-0.125	0.055	0.024	43.714
Adlercreutzia	rs6664405	T	C	0.144	-0.095	0.021	5.23422E-06	0.189	0.076	0.013	41.182
Adlercreutzia	rs7680684	C	T	0.325	-0.083	0.017	9.76904E-07	-0.109	0.057	0.055	56.109
Adlercreutzia	rs9490822	C	T	0.447	-0.073	0.016	2.54421E-06	-0.053	0.054	0.324	49.049
Adlercreutzia	rs9915817	T	C	0.329	0.075	0.017	8.21673E-06	0.013	0.059	0.833	45.561
Akkermansia	rs111862613	T	C	0.166	0.091	0.020	3.39236E-06	0.024	0.072	0.741	42.255
Akkermansia	rs117107102	A	G	0.045	0.204	0.043	3.01139E-06	-0.052	0.128	0.686	65.670
Akkermansia	rs11729256	T	C	0.239	0.075	0.015	6.58393E-07	0.075	0.071	0.288	37.603
Akkermansia	rs12908520	G	A	0.456	0.062	0.013	2.26482E-06	0.032	0.054	0.558	34.786
Akkermansia	rs2602429	C	T	0.222	0.075	0.016	2.71569E-06	0.007	0.061	0.903	35.225
Akkermansia	rs4242783	G	A	0.287	0.069	0.015	2.99799E-06	-0.100	0.060	0.093	35.352
Akkermansia	rs4936098	A	G	0.340	0.065	0.014	1.10359E-06	-0.013	0.056	0.815	34.755
Akkermansia	rs61779207	G	A	0.178	-0.076	0.017	6.32116E-06	0.000	0.065	0.995	31.079
Akkermansia	rs74542928	T	C	0.079	0.113	0.024	1.4802E-06	0.209	0.125	0.093	33.713
Akkermansia	rs9349825	A	G	0.279	-0.070	0.015	2.59886E-06	0.046	0.068	0.502	36.600
Akkermansia	rs941682	G	A	0.311	-0.063	0.014	9.17126E-06	0.095	0.060	0.112	31.545
Alistipes	rs1107244	G	A	0.079	0.076	0.017	3.59059E-06	0.133	0.100	0.181	15.279
Alistipes	rs11769002	G	A	0.401	-0.053	0.011	1.44962E-06	-0.017	0.055	0.749	24.658
Alistipes	rs11958296	A	G	0.071	-0.098	0.022	9.29574E-06	-0.038	0.129	0.765	23.199
Alistipes	rs12990744	C	T	0.123	-0.078	0.017	8.20725E-06	-0.005	0.088	0.959	23.943

Alistipes	rs1689282	A	C	0.340	-0.052	0.011	5.27578E-06	0.024	0.057	0.681	22.286
Alistipes	rs2290844	C	T	0.113	0.081	0.019	9.10135E-06	-0.092	0.086	0.286	24.445
Alistipes	rs2450745	A	C	0.083	-0.081	0.018	7.11836E-06	0.215	0.105	0.040	18.019
Alistipes	rs2875322	T	C	0.207	-0.058	0.013	8.77703E-06	0.095	0.072	0.183	20.327
Alistipes	rs34417064	A	G	0.512	-0.048	0.011	7.00749E-06	0.051	0.054	0.343	21.316
Alistipes	rs4810359	A	G	0.147	-0.065	0.015	7.49669E-06	-0.048	0.081	0.557	19.607
Alistipes	rs7129639	C	A	0.402	-0.052	0.011	1.78422E-06	0.012	0.057	0.828	24.321
Alistipes	rs8130320	A	G	0.482	-0.049	0.011	4.83612E-06	-0.035	0.054	0.519	22.015
Allisonella	rs1901739	T	G	0.495	0.116	0.025	3.58801E-06	-0.100	0.054	0.063	123.703
Allisonella	rs35110698	T	C	0.149	-0.146	0.032	5.72322E-06	-0.114	0.076	0.135	100.167
Allisonella	rs35778461	C	T	0.192	0.147	0.030	1.2063E-06	-0.013	0.064	0.835	123.137
Allisonella	rs594561	C	T	0.441	0.112	0.025	9.41103E-06	-0.186	0.053	0.000	114.616
Allisonella	rs602075	A	G	0.231	0.169	0.030	3.56575E-08	-0.088	0.061	0.146	187.697
Allisonella	rs6742198	G	A	0.196	0.149	0.032	3.34514E-06	-0.080	0.063	0.202	129.381
Allisonella	rs76904847	G	A	0.190	0.149	0.033	6.08751E-06	-0.090	0.071	0.203	125.312
Allisonella	rs7898615	T	G	0.115	0.168	0.037	8.87277E-06	-0.114	0.079	0.148	106.159
Alloprevotella	rs2154444	T	G	0.223	0.138	0.031	8.36755E-06	0.031	0.061	0.611	122.125
Alloprevotella	rs34619204	G	A	0.200	-0.156	0.034	8.8419E-06	-0.068	0.071	0.336	143.888
Alloprevotella	rs4364940	A	G	0.279	0.126	0.028	8.58353E-06	0.029	0.058	0.621	118.748
Alloprevotella	rs4680035	A	G	0.546	-0.120	0.026	4.99052E-06	-0.005	0.055	0.931	130.987
Alloprevotella	rs58212166	A	G	0.133	-0.162	0.036	7.93614E-06	-0.069	0.069	0.321	111.123
Anaerofilum	rs10794359	T	C	0.487	-0.095	0.020	2.23496E-06	-0.014	0.054	0.798	83.690
Anaerofilum	rs1563175	A	C	0.444	0.092	0.020	5.53848E-06	-0.010	0.054	0.855	77.611
Anaerofilum	rs17012738	T	G	0.408	0.090	0.020	7.24168E-06	0.040	0.054	0.459	72.564
Anaerofilum	rs17096874	C	T	0.181	-0.126	0.027	2.8603E-06	-0.083	0.066	0.206	87.152
Anaerofilum	rs4244069	G	A	0.113	-0.147	0.033	9.81202E-06	0.050	0.081	0.540	79.721

Anaerofilum	rs4506496	G	A	0.337	0.103	0.021	1.4866E-06	0.018	0.059	0.753	87.503
Anaerofilum	rs712981	A	C	0.419	0.101	0.020	6.83115E-07	0.007	0.055	0.906	91.065
Anaerofilum	rs79598899	C	T	0.083	0.183	0.036	3.74728E-07	0.065	0.127	0.608	93.019
Anaerofilum	rs816292	T	C	0.243	-0.113	0.022	2.64038E-07	-0.007	0.059	0.910	86.436
Anaerofilum	rs9299345	T	C	0.117	-0.136	0.030	8.04309E-06	-0.115	0.091	0.205	70.924
Anaerostipes	rs10502061	A	G	0.094	0.084	0.019	7.93539E-06	0.092	0.084	0.274	21.926
Anaerostipes	rs2014785	T	C	0.389	0.052	0.011	4.68061E-06	-0.072	0.054	0.183	23.204
Anaerostipes	rs2396460	T	C	0.439	-0.051	0.011	2.91088E-06	0.054	0.054	0.317	23.774
Anaerostipes	rs2804244	A	G	0.455	-0.053	0.011	2.04051E-06	0.041	0.055	0.455	25.672
Anaerostipes	rs3900776	G	A	0.071	-0.110	0.024	2.74765E-06	-0.015	0.161	0.928	29.172
Anaerostipes	rs60983350	G	A	0.435	-0.054	0.012	4.42054E-06	-0.014	0.057	0.809	26.319
Anaerostipes	rs62157625	T	C	0.113	0.089	0.019	1.44909E-06	0.045	0.082	0.587	28.952
Anaerostipes	rs62215703	G	A	0.220	0.064	0.014	1.9777E-06	0.034	0.065	0.601	26.192
Anaerostipes	rs6474958	A	G	0.403	-0.050	0.011	6.74483E-06	-0.010	0.058	0.865	22.085
Anaerostipes	rs6726833	C	A	0.094	-0.088	0.019	3.32217E-06	-0.177	0.099	0.073	24.174
Anaerostipes	rs6854026	T	C	0.512	-0.051	0.011	3.19578E-06	-0.004	0.054	0.947	23.724
Anaerostipes	rs7193624	C	T	0.149	0.075	0.015	5.35482E-07	0.208	0.097	0.032	26.256
Anaerostipes	rs78735375	A	C	0.053	-0.137	0.031	5.33384E-06	0.014	0.136	0.917	34.646
Anaerotruncus	rs10150232	A	G	0.254	0.057	0.012	6.67667E-06	-0.098	0.067	0.142	22.347
Anaerotruncus	rs11018566	A	G	0.052	-0.156	0.037	6.13709E-06	0.331	0.107	0.002	44.126
Anaerotruncus	rs115414803	A	C	0.059	-0.144	0.032	6.83473E-06	-0.094	0.112	0.397	42.259
Anaerotruncus	rs1272208	G	T	0.280	-0.061	0.013	4.27982E-06	0.054	0.063	0.393	27.730
Anaerotruncus	rs1431492	C	T	0.183	-0.065	0.015	7.35565E-06	-0.034	0.074	0.641	23.545
Anaerotruncus	rs17734739	T	C	0.190	0.066	0.015	7.42772E-06	0.103	0.077	0.181	24.614
Anaerotruncus	rs34449434	A	C	0.456	-0.050	0.011	9.85065E-06	-0.050	0.056	0.373	22.503
Anaerotruncus	rs4669806	G	T	0.250	0.058	0.012	2.4206E-06	0.064	0.065	0.326	22.844

Anaerotruncus	rs6494922	A	G	0.073	0.090	0.020	6.62362E-06	-0.024	0.119	0.839	20.162
Anaerotruncus	rs6563550	T	C	0.079	0.088	0.018	2.34517E-07	0.133	0.100	0.181	20.434
Anaerotruncus	rs7155595	C	A	0.309	0.054	0.012	7.54982E-06	0.083	0.058	0.153	22.812
Anaerotruncus	rs8005030	C	T	0.302	0.055	0.012	2.28298E-06	-0.048	0.057	0.401	23.806
Anaerotruncus	rs9347879	T	C	0.460	0.051	0.011	4.22333E-06	-0.106	0.054	0.048	23.374
Bacteroides	rs11585893	A	G	0.148	-0.074	0.015	1.79511E-06	0.037	0.063	0.555	25.425
Bacteroides	rs13207588	A	G	0.233	-0.059	0.013	7.48504E-06	0.024	0.068	0.720	22.976
Bacteroides	rs1340391	T	C	0.192	-0.059	0.013	6.7301E-06	-0.103	0.078	0.189	19.947
Bacteroides	rs17619981	T	G	0.066	0.088	0.019	2.68683E-06	0.016	0.078	0.843	17.465
Bacteroides	rs2023437	T	C	0.124	-0.078	0.017	5.01824E-06	0.116	0.082	0.154	24.466
Bacteroides	rs66710942	C	T	0.416	0.049	0.011	5.86259E-06	-0.027	0.054	0.610	21.240
Bacteroides	rs6795673	C	T	0.412	0.054	0.011	3.37909E-07	0.011	0.054	0.833	25.798
Bacteroides	rs9507307	C	T	0.190	0.060	0.013	2.1271E-06	-0.072	0.062	0.250	20.638
Barnesiella	rs11155559	T	C	0.095	0.096	0.021	8.91914E-06	0.120	0.093	0.197	28.974
Barnesiella	rs12909713	C	T	0.469	-0.055	0.012	4.94527E-06	-0.030	0.053	0.568	27.745
Barnesiella	rs13242616	T	C	0.370	-0.058	0.012	2.29017E-06	0.013	0.058	0.827	29.172
Barnesiella	rs199035	G	A	0.509	0.056	0.012	3.00294E-06	0.026	0.054	0.629	28.738
Barnesiella	rs2276875	A	G	0.255	-0.070	0.014	4.65027E-07	-0.012	0.062	0.851	33.885
Barnesiella	rs2428166	G	A	0.049	-0.166	0.034	8.50874E-07	0.012	0.227	0.958	46.861
Barnesiella	rs35177866	A	G	0.124	0.092	0.019	2.94763E-06	0.021	0.101	0.833	33.640
Barnesiella	rs62251337	A	G	0.158	-0.069	0.015	4.24161E-06	0.004	0.076	0.962	23.323
Barnesiella	rs72684847	T	C	0.066	-0.114	0.025	6.76169E-06	0.010	0.101	0.925	29.453
Barnesiella	rs76181748	C	T	0.144	-0.078	0.017	6.7766E-06	0.012	0.064	0.853	27.460
Barnesiella	rs77455852	T	G	0.121	-0.089	0.020	3.16354E-06	0.010	0.076	0.891	31.121
Barnesiella	rs79795328	A	G	0.125	-0.082	0.018	4.23302E-06	0.009	0.078	0.905	26.960
Bifidobacterium	rs12022129	G	A	0.221	0.062	0.014	7.99643E-06	-0.099	0.060	0.100	24.229

Bifidobacterium	rs182549	C	T	0.491	0.120	0.013	1.27822E-20	-0.077	0.055	0.163	132.288
Bifidobacterium	rs2491158	G	A	0.182	0.071	0.016	8.04716E-06	0.015	0.078	0.851	27.759
Bifidobacterium	rs2686790	T	C	0.115	0.071	0.016	7.49893E-06	0.013	0.075	0.865	18.741
Bifidobacterium	rs540489	T	G	0.234	-0.064	0.014	5.19458E-06	-0.038	0.070	0.585	26.736
Bifidobacterium	rs55888705	A	G	0.387	0.055	0.012	6.67019E-06	-0.074	0.059	0.213	25.998
Bifidobacterium	rs56108664	T	C	0.185	0.073	0.016	2.4402E-06	-0.059	0.080	0.457	29.501
Bifidobacterium	rs5746486	T	C	0.354	-0.054	0.012	8.99955E-06	0.069	0.055	0.212	24.144
Bifidobacterium	rs62181700	G	A	0.281	-0.062	0.013	2.17246E-06	-0.012	0.062	0.849	28.977
Bifidobacterium	rs7322849	T	C	0.096	0.112	0.020	1.08368E-08	0.145	0.093	0.121	40.471
Bifidobacterium	rs75344046	C	T	0.050	0.232	0.051	4.86354E-06	0.274	0.127	0.030	93.998
Bifidobacterium	rs857444	C	T	0.361	0.056	0.012	3.57097E-06	-0.129	0.056	0.020	26.396
Bilophila	rs11069458	T	C	0.190	-0.068	0.016	7.71547E-06	-0.040	0.069	0.564	26.199
Bilophila	rs1241171	G	A	0.216	-0.069	0.015	4.24037E-06	-0.064	0.076	0.396	29.816
Bilophila	rs1571225	C	T	0.149	0.083	0.017	1.12289E-06	-0.041	0.072	0.571	31.865
Bilophila	rs1969927	G	A	0.380	0.056	0.013	9.0722E-06	-0.023	0.057	0.685	27.577
Bilophila	rs2728491	G	T	0.310	-0.063	0.014	6.33255E-06	-0.052	0.063	0.406	30.939
Bilophila	rs3827020	C	T	0.190	0.077	0.016	1.78875E-06	0.000	0.068	0.999	33.207
Bilophila	rs4798126	G	A	0.150	0.073	0.017	7.14662E-06	-0.042	0.068	0.534	25.165
Bilophila	rs542415	T	C	0.340	-0.061	0.013	4.70948E-06	-0.043	0.055	0.436	31.042
Bilophila	rs60178956	G	A	0.235	-0.062	0.014	8.05619E-06	0.015	0.064	0.815	25.746
Bilophila	rs6793291	C	A	0.092	0.113	0.024	3.1132E-06	-0.161	0.121	0.185	39.169
Bilophila	rs72676854	T	C	0.066	0.123	0.027	5.62268E-06	0.114	0.121	0.348	34.191
Bilophila	rs7802841	C	A	0.351	0.067	0.014	1.76952E-06	-0.027	0.058	0.649	37.587
Bilophila	rs9899990	A	G	0.066	-0.103	0.023	9.06691E-06	-0.116	0.097	0.234	23.743
Blautia	rs11149971	C	T	0.054	0.118	0.023	1.03934E-06	-0.061	0.118	0.603	25.813
Blautia	rs115043014	G	A	0.044	-0.207	0.044	5.186E-06	0.244	0.206	0.238	65.659

Blautia	rs117001700	T	C	0.044	0.196	0.044	8.83912E-06	0.175	0.222	0.431	59.321
Blautia	rs12453000	C	T	0.220	0.063	0.013	1.26117E-06	-0.056	0.078	0.470	24.620
Blautia	rs16892041	T	C	0.171	-0.062	0.014	8.82028E-06	0.016	0.065	0.812	20.176
Blautia	rs2788271	T	G	0.165	-0.058	0.013	7.16388E-06	-0.096	0.071	0.175	16.757
Blautia	rs3005511	A	G	0.336	0.050	0.011	6.18905E-06	0.056	0.058	0.341	20.568
Blautia	rs4926264	T	C	0.079	0.083	0.018	5.10293E-06	0.004	0.087	0.966	18.128
Blautia	rs67794373	C	T	0.214	0.060	0.012	1.00003E-06	0.036	0.064	0.579	22.340
Blautia	rs682885	A	G	0.417	-0.049	0.011	4.4908E-06	-0.028	0.057	0.627	21.722
Blautia	rs72973581	A	G	0.055	0.125	0.027	1.73934E-06	0.159	0.115	0.165	29.760
Blautia	rs7860714	A	G	0.334	-0.050	0.011	4.09147E-06	-0.070	0.056	0.210	20.597
Butyricicoccus	rs10084203	A	G	0.242	0.055	0.012	8.58646E-06	-0.014	0.079	0.862	20.329
Butyricicoccus	rs12034718	A	G	0.165	0.070	0.016	9.57686E-06	0.040	0.064	0.538	24.878
Butyricicoccus	rs12585793	T	C	0.053	-0.262	0.056	5.79191E-06	-0.052	0.160	0.744	126.753
Butyricicoccus	rs2017189	G	T	0.422	-0.051	0.011	3.87256E-06	0.054	0.054	0.317	23.013
Butyricicoccus	rs4962426	G	T	0.198	0.061	0.014	7.3849E-06	0.006	0.068	0.932	21.981
Butyricicoccus	rs56221232	T	C	0.108	0.083	0.017	7.6194E-07	-0.047	0.089	0.595	24.316
Butyricicoccus	rs62478070	T	G	0.044	0.224	0.049	5.9377E-06	-0.105	0.169	0.537	77.256
Butyricicoccus	rs7322368	T	C	0.085	0.082	0.018	5.5178E-06	-0.151	0.093	0.106	18.899
Butyricimonas	rs11228830	A	G	0.071	0.135	0.030	6.55162E-06	0.094	0.095	0.322	44.137
Butyricimonas	rs113054641	G	A	0.085	-0.145	0.027	1.73694E-07	0.018	0.130	0.892	59.749
Butyricimonas	rs12304031	G	A	0.129	-0.086	0.020	6.70067E-06	0.068	0.081	0.405	30.767
Butyricimonas	rs12458763	A	C	0.072	0.122	0.027	6.36646E-06	-0.118	0.128	0.359	36.381
Butyricimonas	rs1862649	G	A	0.092	0.113	0.025	4.76256E-06	-0.075	0.105	0.477	39.099
Butyricimonas	rs2114713	G	T	0.451	0.063	0.014	6.87713E-06	-0.100	0.054	0.063	35.798
Butyricimonas	rs62130338	G	A	0.262	-0.073	0.016	3.8981E-06	-0.043	0.056	0.450	38.178
Butyricimonas	rs62390301	T	C	0.183	-0.087	0.017	7.41905E-07	0.022	0.067	0.744	41.867

Butyricimonas	rs7083431	A	C	0.355	0.070	0.014	8.8546E-07	0.036	0.060	0.552	41.656
Butyricimonas	rs71428626	G	T	0.073	-0.133	0.029	4.80413E-06	0.195	0.150	0.192	43.909
Butyricimonas	rs72814525	A	G	0.323	0.066	0.015	8.24599E-06	0.013	0.062	0.832	35.450
Butyricimonas	rs78453362	A	G	0.058	-0.149	0.033	4.0581E-06	-0.055	0.172	0.750	44.665
Butyricimonas	rs9657374	C	T	0.302	0.068	0.015	4.50359E-06	0.082	0.059	0.161	35.908
Butyrivibrio	rs1007475	G	T	0.294	0.118	0.026	7.92312E-06	-0.049	0.060	0.416	106.776
Butyrivibrio	rs11761679	T	C	0.156	0.155	0.032	2.20231E-06	-0.011	0.077	0.885	116.474
Butyrivibrio	rs142855850	A	G	0.101	0.205	0.046	6.85543E-06	0.076	0.092	0.408	141.543
Butyrivibrio	rs16934069	T	C	0.199	-0.134	0.030	8.8591E-06	-0.043	0.070	0.541	105.155
Butyrivibrio	rs16941336	C	T	0.264	0.127	0.027	1.53182E-06	-0.036	0.064	0.575	116.674
Butyrivibrio	rs17163238	G	A	0.200	0.141	0.031	5.51196E-06	-0.006	0.068	0.933	117.301
Butyrivibrio	rs4537857	T	C	0.292	-0.125	0.026	1.80192E-06	0.042	0.057	0.458	118.504
Butyrivibrio	rs486484	A	G	0.471	-0.108	0.024	6.61084E-06	-0.011	0.054	0.839	107.913
Butyrivibrio	rs4928024	A	G	0.098	-0.175	0.039	8.18641E-06	0.033	0.070	0.633	99.858
Butyrivibrio	rs72723662	C	T	0.100	0.224	0.045	7.86241E-07	0.012	0.078	0.873	167.850
Butyrivibrio	rs74622183	A	G	0.088	-0.201	0.043	2.45748E-06	-0.057	0.094	0.545	119.109
Butyrivibrio	rs77356209	T	C	0.048	0.217	0.048	6.6643E-06	0.155	0.129	0.230	78.696
Butyrivibrio	rs7752361	A	G	0.504	-0.119	0.024	7.69113E-07	-0.029	0.054	0.592	131.270
Butyrivibrio	rs7763512	G	A	0.383	0.120	0.025	3.10655E-06	-0.061	0.054	0.262	125.296
Butyrivibrio	rs9349693	A	G	0.311	0.118	0.026	5.54958E-06	-0.051	0.059	0.383	110.053
CandidatusSoleaferrea	rs10090365	A	G	0.559	-0.083	0.018	4.17309E-06	-0.096	0.054	0.073	63.179
CandidatusSoleaferrea	rs10809135	T	C	0.491	0.083	0.018	5.47174E-06	0.083	0.054	0.129	64.110
CandidatusSoleaferrea	rs36155147	C	T	0.362	0.105	0.024	5.41138E-06	0.034	0.057	0.555	93.782
CandidatusSoleaferrea	rs4294381	T	C	0.180	0.112	0.023	1.3685E-06	0.024	0.073	0.741	68.368

CandidatusSoleaferrea	rs4678258	T	C	0.276	0.099	0.022	5.53183E-06	0.121	0.063	0.053	71.596
CandidatusSoleaferrea	rs6489992	A	G	0.385	-0.084	0.019	7.88786E-06	-0.040	0.055	0.468	61.526
CandidatusSoleaferrea	rs6494306	A	G	0.197	-0.097	0.021	5.80051E-06	0.089	0.057	0.119	54.631
CandidatusSoleaferrea	rs7400877	T	C	0.251	-0.095	0.021	9.29448E-06	0.085	0.066	0.194	62.500
CandidatusSoleaferrea	rs9973954	A	G	0.306	0.089	0.020	5.94796E-06	0.083	0.056	0.138	62.215
Catenibacterium	rs12404911	C	T	0.170	0.141	0.030	2.79891E-06	-0.066	0.068	0.334	103.047
Catenibacterium	rs212393	G	A	0.310	-0.135	0.029	3.62489E-06	-0.002	0.066	0.974	144.671
Catenibacterium	rs73128290	A	G	0.263	0.130	0.028	4.29372E-06	-0.098	0.058	0.094	120.540
Catenibacterium	rs7742829	C	T	0.420	0.114	0.025	5.60729E-06	0.010	0.054	0.859	117.038
ChristensenellaceaeR	rs10461257	A	G	0.285	-0.055	0.012	6.50746E-06	0.027	0.057	0.633	22.813
ChristensenellaceaeR	rs17081797	A	G	0.069	-0.090	0.020	3.34031E-06	-0.009	0.109	0.936	19.184
ChristensenellaceaeR	rs62132810	A	G	0.108	-0.083	0.018	5.66711E-06	-0.056	0.077	0.470	24.365
ChristensenellaceaeR	rs62190261	A	C	0.091	0.096	0.021	8.74023E-06	0.123	0.096	0.197	27.770
ChristensenellaceaeR	rs62467127	C	T	0.045	0.114	0.025	3.25362E-06	-0.241	0.171	0.159	20.415
ChristensenellaceaeR	rs73952017	C	T	0.088	-0.086	0.019	8.45769E-06	-0.119	0.088	0.178	21.793
ChristensenellaceaeR	rs78521377	C	T	0.053	0.125	0.027	5.60674E-06	0.231	0.152	0.129	28.650
ChristensenellaceaeR	rs892686	A	G	0.451	0.051	0.011	3.96861E-06	0.020	0.054	0.716	24.035
Clostridiuminnocuumgroup	rs10506058	A	G	0.397	0.100	0.022	8.92443E-06	-0.053	0.054	0.332	87.668
Clostridiuminnocuumgr	rs1942371	G	A	0.130	-0.158	0.034	4.06337E-06	0.004	0.082	0.957	104.195

oup											
Clostridiuminnocuumgroup	rs40656	C	T	0.189	0.143	0.031	8.6152E-06	0.106	0.067	0.114	115.090
Clostridiuminnocuumgroup	rs4869133	G	A	0.089	-0.181	0.041	7.24455E-06	0.027	0.071	0.707	96.998
Clostridiuminnocuumgroup	rs61267978	T	C	0.124	0.147	0.032	5.58513E-06	0.001	0.082	0.986	86.769
Clostridiuminnocuumgroup	rs6577484	G	A	0.148	0.160	0.036	8.40602E-06	0.058	0.088	0.512	119.867
Clostridiuminnocuumgroup	rs6890185	T	C	0.311	0.113	0.023	1.12244E-06	-0.050	0.057	0.386	101.683
Clostridiuminnocuumgroup	rs77845139	A	G	0.295	-0.115	0.026	8.40622E-06	0.058	0.062	0.346	101.462
Clostridiumsensustricto1	rs11264403	G	A	0.040	-0.139	0.033	7.75544E-06	-0.078	0.102	0.448	27.145
Clostridiumsensustricto1	rs116847295	C	T	0.076	0.110	0.025	4.58118E-06	0.082	0.081	0.311	31.040
Clostridiumsensustricto1	rs12341505	G	A	0.111	0.081	0.018	4.82174E-06	0.037	0.092	0.691	23.875
Clostridiumsensustricto1	rs2795528	G	A	0.049	-0.184	0.039	2.7204E-06	0.155	0.116	0.180	57.905
Clostridiumsensustricto1	rs2817172	C	T	0.413	0.058	0.012	2.76655E-06	0.033	0.055	0.544	30.093
Clostridiumsensustricto1	rs550843	T	C	0.149	-0.078	0.017	2.05071E-06	-0.033	0.060	0.585	28.590
Collinsella	rs10890671	T	C	0.527	-0.054	0.012	6.52136E-06	-0.007	0.054	0.892	26.442

Collinsella	rs11597285	G	T	0.425	-0.054	0.012	9.37615E-06	-0.047	0.055	0.395	25.966
Collinsella	rs1496626	T	C	0.154	-0.072	0.016	6.78175E-06	-0.082	0.079	0.297	24.977
Collinsella	rs149807560	C	A	0.090	-0.104	0.024	7.0987E-06	-0.020	0.106	0.851	32.548
Collinsella	rs2103510	G	A	0.131	0.079	0.017	2.42474E-06	-0.007	0.083	0.931	25.900
Collinsella	rs62448871	C	A	0.461	-0.054	0.012	6.78253E-06	-0.076	0.054	0.163	26.645
Collinsella	rs73052258	G	A	0.096	0.093	0.020	1.7158E-06	-0.135	0.101	0.180	27.684
Collinsella	rs75672793	A	G	0.071	-0.109	0.024	6.13549E-06	-0.139	0.127	0.272	28.588
Collinsella	rs9541268	C	A	0.096	0.096	0.020	8.79419E-07	-0.104	0.093	0.262	29.472
Coprobacter	rs11532348	C	T	0.183	-0.104	0.023	5.71184E-06	0.023	0.075	0.759	59.397
Coprobacter	rs12684609	T	C	0.198	0.101	0.022	6.10177E-06	-0.038	0.067	0.570	59.354
Coprobacter	rs12996055	A	C	0.192	0.092	0.021	8.07639E-06	0.071	0.061	0.246	48.438
Coprobacter	rs143662916	C	T	0.058	0.253	0.054	3.07252E-06	-0.206	0.159	0.193	128.805
Coprobacter	rs189356	G	A	0.522	0.078	0.017	6.26291E-06	-0.028	0.054	0.610	56.008
Coprobacter	rs213863	C	T	0.309	-0.089	0.019	2.34883E-06	0.098	0.056	0.078	61.888
Coprobacter	rs28402691	T	C	0.167	0.111	0.025	9.55668E-06	0.098	0.075	0.196	62.731
Coprobacter	rs305411	A	G	0.135	0.129	0.026	1.00906E-06	-0.043	0.089	0.630	71.882
Coprobacter	rs3828477	G	T	0.281	-0.091	0.020	2.88974E-06	-0.009	0.057	0.876	61.905
Coprobacter	rs72821405	T	C	0.089	-0.147	0.032	4.7576E-06	0.072	0.096	0.452	64.476
Coprobacter	rs74919520	G	A	0.128	0.126	0.028	5.76326E-06	-0.055	0.086	0.527	65.000
Coprococcus1	rs1010560	C	A	0.296	0.058	0.012	1.95915E-06	0.046	0.061	0.445	25.777
Coprococcus1	rs12794898	G	T	0.094	0.090	0.020	4.91531E-06	0.001	0.081	0.986	25.619
Coprococcus1	rs1519491	T	C	0.409	0.050	0.011	8.95283E-06	-0.041	0.055	0.450	22.110
Coprococcus1	rs1576241	A	G	0.439	-0.051	0.011	3.32862E-06	0.035	0.055	0.525	23.560
Coprococcus1	rs1762123	C	T	0.072	-0.089	0.020	8.01468E-06	-0.046	0.090	0.607	19.398
Coprococcus1	rs2907920	A	G	0.290	0.056	0.013	7.65327E-06	-0.087	0.060	0.147	23.827
Coprococcus1	rs4277593	G	A	0.483	-0.059	0.011	1.13969E-07	0.006	0.054	0.918	31.466

Coprococcus1	rs56405618	A	G	0.111	-0.090	0.019	1.57195E-06	0.036	0.084	0.672	29.190
Coprococcus1	rs73031725	T	C	0.061	0.168	0.036	1.97917E-06	-0.060	0.152	0.695	58.830
Coprococcus1	rs73167075	T	C	0.240	0.057	0.013	8.56864E-06	-0.034	0.065	0.607	21.976
Coprococcus1	rs74101919	T	C	0.187	-0.072	0.014	1.03291E-06	0.039	0.086	0.649	28.858
Coprococcus1	rs946513	C	T	0.055	0.206	0.046	8.61855E-06	0.040	0.129	0.754	80.752
Coprococcus2	rs10070053	A	G	0.408	0.059	0.014	7.64883E-06	-0.062	0.055	0.256	31.333
Coprococcus2	rs12634070	T	C	0.193	0.074	0.016	9.95104E-06	-0.046	0.061	0.449	31.012
Coprococcus2	rs2482516	C	T	0.226	0.075	0.016	4.72333E-06	-0.026	0.065	0.688	36.540
Coprococcus2	rs35890118	A	G	0.317	-0.067	0.015	8.25962E-06	-0.011	0.062	0.860	35.228
Coprococcus2	rs61823518	A	C	0.095	-0.096	0.022	6.68258E-06	0.072	0.087	0.409	28.935
Coprococcus2	rs6677933	C	T	0.242	-0.080	0.016	1.18501E-06	0.100	0.076	0.188	43.587
Coprococcus2	rs72680320	T	C	0.414	-0.065	0.014	2.27115E-06	-0.012	0.057	0.830	37.585
Coprococcus2	rs9426473	A	G	0.279	0.073	0.016	6.30687E-06	-0.112	0.061	0.068	39.141
Coprococcus3	rs10810043	A	G	0.390	0.052	0.012	9.27424E-06	0.032	0.057	0.571	23.211
Coprococcus3	rs11077359	T	C	0.200	-0.065	0.015	9.63598E-06	0.041	0.071	0.567	24.461
Coprococcus3	rs11080344	C	T	0.474	0.052	0.011	4.78692E-06	-0.016	0.054	0.763	24.468
Coprococcus3	rs13247359	G	A	0.480	0.051	0.011	7.32611E-06	0.086	0.054	0.110	24.040
Coprococcus3	rs178271	T	C	0.053	0.145	0.029	7.81088E-07	0.163	0.206	0.429	38.714
Coprococcus3	rs4575475	G	A	0.237	0.062	0.014	7.04284E-06	-0.030	0.064	0.635	25.460
Coprococcus3	rs7521171	G	A	0.338	-0.060	0.013	4.32242E-06	-0.012	0.058	0.836	29.240
Coprococcus3	rs8100692	T	C	0.427	0.058	0.011	4.16459E-07	0.121	0.054	0.024	29.974
DefluviitaleaceaeUCG011	rs112893842	T	C	0.097	0.114	0.023	1.44535E-06	0.059	0.093	0.524	41.859
DefluviitaleaceaeUCG011	rs1582238	T	C	0.372	0.081	0.017	1.56775E-06	0.049	0.056	0.378	55.685
DefluviitaleaceaeUCG0	rs2892880	G	A	0.260	0.082	0.018	6.82881E-06	0.049	0.062	0.428	47.357

11											
DefluviitaleaceaeUCG011	rs4344384	G	T	0.474	0.072	0.016	4.83238E-06	-0.105	0.054	0.050	46.998
DefluviitaleaceaeUCG011	rs4677103	A	G	0.171	0.098	0.020	9.59557E-07	-0.023	0.071	0.742	49.855
DefluviitaleaceaeUCG011	rs55658617	T	C	0.070	0.174	0.036	2.15292E-06	0.185	0.144	0.198	72.496
DefluviitaleaceaeUCG011	rs72731813	C	T	0.082	-0.147	0.029	4.33116E-07	0.087	0.125	0.487	59.831
DefluviitaleaceaeUCG011	rs9608282	T	G	0.066	0.143	0.030	2.52045E-06	0.070	0.150	0.640	46.048
DefluviitaleaceaeUCG011	rs9725395	A	G	0.088	-0.138	0.030	3.51921E-06	-0.075	0.085	0.373	56.214
Desulfovibrio	rs12031543	T	C	0.076	-0.127	0.028	6.54764E-06	-0.068	0.078	0.382	41.504
Desulfovibrio	rs13066142	G	A	0.093	0.119	0.025	3.78665E-06	-0.087	0.092	0.348	44.189
Desulfovibrio	rs16863365	A	G	0.117	0.109	0.023	1.7945E-06	-0.112	0.127	0.378	45.564
Desulfovibrio	rs2032031	A	G	0.473	-0.065	0.015	9.14277E-06	0.050	0.053	0.348	39.300
Desulfovibrio	rs2590913	G	A	0.073	0.154	0.034	6.65421E-06	-0.018	0.122	0.882	59.131
Desulfovibrio	rs2853179	C	T	0.249	0.081	0.017	2.42439E-06	-0.020	0.064	0.751	45.268
Desulfovibrio	rs4797774	G	A	0.069	0.213	0.047	5.64315E-06	0.012	0.136	0.932	106.515
Desulfovibrio	rs6580353	T	C	0.260	0.077	0.017	4.93857E-06	0.020	0.068	0.771	42.082
Desulfovibrio	rs72647089	T	G	0.106	-0.107	0.024	8.29763E-06	0.064	0.098	0.515	39.708
Desulfovibrio	rs7729080	C	A	0.289	-0.070	0.016	9.95869E-06	0.092	0.059	0.116	37.329
Dialister	rs10138457	T	C	0.072	-0.113	0.026	7.88122E-06	-0.102	0.092	0.267	31.213
Dialister	rs10938938	G	A	0.194	-0.077	0.017	7.37063E-06	0.018	0.073	0.803	34.350
Dialister	rs11071887	T	C	0.263	0.066	0.015	5.91407E-06	0.084	0.057	0.142	31.276

Dialister	rs11166701	G	A	0.516	-0.066	0.013	5.50746E-07	-0.043	0.054	0.424	39.418
Dialister	rs2314294	T	C	0.118	0.087	0.019	8.07899E-06	-0.109	0.079	0.165	28.730
Dialister	rs2435610	A	C	0.260	0.065	0.014	5.93269E-06	-0.012	0.062	0.847	29.628
Dialister	rs4747450	C	A	0.266	0.067	0.015	5.83729E-06	-0.030	0.064	0.640	32.088
Dialister	rs4753063	G	A	0.441	-0.060	0.013	4.85684E-06	0.075	0.054	0.162	32.211
Dialister	rs75416973	A	G	0.226	0.073	0.016	9.46091E-06	0.012	0.065	0.851	33.949
Dialister	rs764177	C	A	0.338	-0.060	0.014	9.60772E-06	-0.006	0.056	0.913	29.735
Dialister	rs76680460	G	A	0.048	-0.161	0.036	8.19213E-06	-0.184	0.132	0.165	43.446
Dorea	rs11150408	T	G	0.445	0.049	0.011	7.05777E-06	-0.007	0.054	0.893	21.622
Dorea	rs12537781	T	C	0.230	-0.056	0.013	9.15137E-06	0.027	0.063	0.669	20.036
Dorea	rs13279148	G	A	0.166	0.072	0.015	2.25283E-06	0.211	0.085	0.013	26.017
Dorea	rs1899291	C	T	0.128	0.070	0.015	4.56686E-06	-0.085	0.074	0.252	19.948
Dorea	rs3005511	A	G	0.336	0.052	0.011	5.28702E-06	0.056	0.058	0.341	21.823
Dorea	rs345219	T	G	0.376	-0.050	0.011	8.8006E-06	0.054	0.054	0.322	21.305
Dorea	rs3752849	G	A	0.033	0.164	0.037	7.68057E-06	0.052	0.124	0.676	31.288
Dorea	rs4793307	C	T	0.246	0.057	0.012	4.0069E-06	-0.125	0.063	0.047	22.423
Dorea	rs62503162	A	G	0.088	-0.097	0.019	7.46605E-07	0.216	0.131	0.100	27.828
Dorea	rs73729431	C	T	0.061	-0.137	0.030	3.1674E-06	-0.030	0.189	0.874	39.530
Eggerthella	rs112205261	T	C	0.058	-0.189	0.040	3.34648E-06	-0.104	0.099	0.291	71.219
Eggerthella	rs13070736	A	C	0.157	-0.121	0.027	7.61915E-06	-0.042	0.073	0.566	71.775
Eggerthella	rs1784446	G	A	0.442	0.091	0.020	5.23136E-06	-0.022	0.053	0.679	74.848
Eggerthella	rs2223081	G	A	0.292	0.103	0.022	3.88889E-06	-0.112	0.060	0.062	80.153
Eggerthella	rs2240838	A	G	0.502	0.098	0.020	7.36205E-07	0.058	0.054	0.286	88.588
Eggerthella	rs3851328	T	G	0.266	-0.108	0.024	4.17589E-06	0.075	0.065	0.248	83.634
Eggerthella	rs6430926	C	T	0.514	0.088	0.020	8.37122E-06	0.087	0.054	0.108	71.183
Eggerthella	rs67490567	T	C	0.198	0.108	0.025	8.94216E-06	-0.013	0.062	0.828	68.760

Eggerthella	rs76663501	C	T	0.082	0.175	0.038	4.82574E-06	0.046	0.122	0.707	84.807
Eisenbergiella	rs11027642	C	T	0.124	0.129	0.028	4.91871E-06	-0.127	0.076	0.096	66.681
Eisenbergiella	rs11079158	T	C	0.220	0.101	0.023	7.34849E-06	0.031	0.065	0.628	63.887
Eisenbergiella	rs11938607	T	C	0.263	0.098	0.022	8.2182E-06	0.018	0.062	0.766	68.334
Eisenbergiella	rs12257723	A	C	0.339	-0.095	0.021	8.85121E-06	-0.020	0.057	0.730	74.904
Eisenbergiella	rs12710729	C	A	0.371	0.089	0.020	9.8441E-06	-0.004	0.057	0.949	68.550
Eisenbergiella	rs13258851	A	G	0.086	0.137	0.030	7.74648E-06	0.076	0.077	0.323	53.983
Eisenbergiella	rs1508033	A	C	0.350	0.092	0.020	3.22889E-06	0.075	0.058	0.201	70.185
Eisenbergiella	rs1553971	T	G	0.161	0.121	0.026	5.26837E-06	-0.001	0.064	0.989	72.779
Eisenbergiella	rs2683098	C	T	0.224	0.107	0.023	2.2375E-06	-0.041	0.065	0.528	73.650
Eisenbergiella	rs3812426	G	A	0.208	0.106	0.022	2.71687E-06	-0.142	0.074	0.054	68.668
Eisenbergiella	rs4462860	G	A	0.344	0.094	0.020	4.16221E-06	-0.010	0.055	0.858	73.268
Enterorhabdus	rs10098492	T	C	0.091	0.132	0.029	6.40572E-06	0.041	0.114	0.722	53.010
Enterorhabdus	rs114731706	T	G	0.084	0.182	0.038	2.17342E-06	0.054	0.151	0.720	93.762
Enterorhabdus	rs2051957	C	T	0.235	0.084	0.019	8.89859E-06	0.014	0.068	0.832	46.953
Enterorhabdus	rs3017103	A	G	0.190	0.098	0.021	2.94044E-06	0.002	0.068	0.972	54.448
Enterorhabdus	rs73331712	T	C	0.043	0.262	0.055	4.84923E-06	-0.120	0.134	0.373	103.483
Enterorhabdus	rs77655283	G	A	0.069	0.133	0.030	5.88042E-06	-0.049	0.105	0.644	41.538
Erysipelatoclostridium	rs1434153	G	A	0.321	-0.068	0.015	6.85295E-06	-0.027	0.054	0.615	37.540
Erysipelatoclostridium	rs16936671	C	T	0.136	-0.097	0.022	6.04309E-06	0.109	0.078	0.163	40.523
Erysipelatoclostridium	rs17804233	T	C	0.482	-0.066	0.014	4.58605E-06	-0.125	0.054	0.020	40.330
Erysipelatoclostridium	rs2901723	C	A	0.503	0.064	0.014	8.79469E-06	0.019	0.054	0.726	37.790
Erysipelatoclostridium	rs340991	A	G	0.298	-0.074	0.016	3.74942E-06	0.013	0.061	0.832	42.182

Erysipelatoclostridium	rs3804326	A	G	0.068	0.141	0.034	9.85273E-06	-0.005	0.124	0.965	46.383
Erysipelatoclostridium	rs45480394	T	G	0.362	-0.069	0.015	7.66155E-06	-0.028	0.056	0.619	40.072
Erysipelatoclostridium	rs4697572	A	G	0.246	-0.081	0.016	7.5935E-07	-0.109	0.067	0.106	44.751
Erysipelatoclostridium	rs58236560	G	T	0.137	-0.111	0.023	2.1612E-06	-0.166	0.082	0.043	53.801
Erysipelatoclostridium	rs61806970	C	T	0.065	0.143	0.032	9.0941E-06	-0.024	0.107	0.821	45.144
Erysipelatoclostridium	rs622418	A	G	0.511	-0.067	0.014	3.68156E-06	0.012	0.054	0.824	40.993
Erysipelatoclostridium	rs6474512	A	C	0.451	0.067	0.014	3.01546E-06	-0.136	0.055	0.014	40.878
Erysipelatoclostridium	rs710230	T	C	0.087	0.143	0.028	6.3312E-07	-0.053	0.104	0.614	59.773
Erysipelatoclostridium	rs7221249	A	G	0.547	0.084	0.014	4.30951E-09	0.053	0.054	0.321	64.336
Erysipelatoclostridium	rs9590927	G	A	0.494	-0.065	0.014	6.39042E-06	-0.023	0.054	0.672	38.339
ErysipelotrichaceaeUCG003	rs10164067	T	G	0.075	-0.103	0.021	1.13086E-06	0.088	0.121	0.466	27.037
ErysipelotrichaceaeUCG003	rs11666127	A	G	0.171	-0.072	0.016	7.90414E-06	-0.071	0.071	0.314	26.931
ErysipelotrichaceaeUCG003	rs11994308	C	T	0.070	0.115	0.024	1.32563E-06	0.069	0.095	0.464	31.692
ErysipelotrichaceaeUCG003	rs12251396	A	G	0.194	-0.071	0.016	9.51839E-06	-0.055	0.074	0.463	28.558
ErysipelotrichaceaeUCG003	rs17798136	G	A	0.062	0.159	0.035	3.23598E-06	0.049	0.110	0.659	53.631
ErysipelotrichaceaeUCG003	rs28568391	A	G	0.449	-0.058	0.012	6.41953E-07	-0.024	0.054	0.652	30.993

ErysipelotrichaceaeUCG003	rs4758231	G	T	0.350	-0.055	0.012	6.54912E-06	-0.026	0.059	0.657	25.433
ErysipelotrichaceaeUCG003	rs59068084	T	G	0.422	0.056	0.012	3.11742E-06	-0.071	0.055	0.199	28.570
ErysipelotrichaceaeUCG003	rs59104037	A	G	0.082	-0.095	0.020	4.48256E-06	0.052	0.080	0.516	24.878
ErysipelotrichaceaeUCG003	rs62403464	T	C	0.164	-0.073	0.016	3.43738E-06	-0.071	0.070	0.309	26.964
ErysipelotrichaceaeUCG003	rs6875357	C	T	0.047	0.166	0.035	6.69637E-06	-0.045	0.139	0.746	44.926
ErysipelotrichaceaeUCG003	rs73074432	C	T	0.166	0.072	0.016	9.9902E-06	0.033	0.086	0.697	26.473
ErysipelotrichaceaeUCG003	rs74988980	G	A	0.044	-0.133	0.035	8.64276E-06	0.078	0.131	0.553	27.210
ErysipelotrichaceaeUCG003	rs75949021	T	C	0.046	-0.170	0.037	3.57507E-06	-0.182	0.136	0.179	46.160
ErysipelotrichaceaeUCG003	rs76502207	T	C	0.091	0.145	0.029	6.41121E-07	-0.130	0.136	0.338	63.553
ErysipelotrichaceaeUCG003	rs8053479	A	G	0.141	-0.084	0.019	5.83413E-06	-0.007	0.084	0.938	31.301
Escherichia	rs112767262	T	C	0.196	0.073	0.016	8.20636E-06	0.045	0.065	0.492	31.078
Escherichia	rs113127095	A	G	0.052	0.151	0.032	3.33349E-06	-0.132	0.136	0.330	41.096
Escherichia	rs113513883	A	G	0.058	0.172	0.038	5.27716E-06	0.081	0.150	0.591	59.380
Escherichia	rs1154904	A	G	0.464	-0.061	0.013	3.03751E-06	-0.053	0.053	0.322	34.384
Escherichia	rs118526	C	A	0.393	-0.059	0.014	7.99566E-06	-0.015	0.059	0.797	30.953
Escherichia	rs2798105	A	G	0.118	-0.101	0.022	8.24501E-06	0.060	0.090	0.506	38.991

Escherichia	rs4731451	G	A	0.372	-0.061	0.014	7.4731E-06	-0.143	0.058	0.013	31.918
Escherichia	rs57024273	T	C	0.318	0.063	0.014	9.70464E-06	-0.058	0.061	0.344	31.246
Escherichia	rs592299	T	C	0.468	-0.059	0.013	4.76927E-06	-0.038	0.054	0.483	32.060
Escherichia	rs73208162	A	G	0.059	-0.119	0.025	2.19391E-06	0.297	0.149	0.047	28.839
Eubacteriumbrachygroup	rs112617308	T	C	0.080	-0.171	0.036	2.38033E-06	-0.128	0.092	0.165	78.701
Eubacteriumbrachygroup	rs12151423	A	G	0.479	0.101	0.023	9.2705E-06	-0.025	0.054	0.634	94.462
Eubacteriumbrachygroup	rs13139592	T	C	0.122	-0.146	0.033	7.96753E-06	-0.055	0.079	0.484	84.301
Eubacteriumbrachygroup	rs1384962	A	G	0.211	0.121	0.027	6.99292E-06	0.007	0.058	0.898	89.572
Eubacteriumbrachygroup	rs2913110	C	T	0.378	0.105	0.023	4.55806E-06	-0.078	0.056	0.167	95.797
Eubacteriumbrachygroup	rs4862235	G	A	0.444	0.105	0.023	3.73143E-06	0.082	0.054	0.129	100.008
Eubacteriumbrachygroup	rs62348779	T	C	0.082	-0.201	0.043	3.78122E-06	-0.168	0.101	0.096	112.133
Eubacteriumbrachygroup	rs6591893	G	A	0.348	0.108	0.024	7.34344E-06	0.104	0.057	0.066	97.977
Eubacteriumbrachygroup	rs720439	A	G	0.252	-0.112	0.025	7.02827E-06	-0.012	0.062	0.843	86.903
Eubacteriumbrachygroup	rs73199919	T	C	0.057	-0.237	0.053	8.15524E-06	0.017	0.125	0.891	110.582
Eubacteriumcoprostanoligenesgroup	rs1020520	T	G	0.204	-0.059	0.013	8.88975E-06	-0.057	0.075	0.450	20.789

Eubacteriumcoprostanoligenesgroup	rs10444197	A	G	0.368	-0.051	0.011	5.98464E-06	-0.091	0.056	0.105	21.846
Eubacteriumcoprostanoligenesgroup	rs11052069	T	C	0.434	0.048	0.011	9.37949E-06	0.003	0.054	0.951	20.598
Eubacteriumcoprostanoligenesgroup	rs11720857	C	T	0.177	0.063	0.014	9.26255E-06	0.025	0.070	0.719	21.272
Eubacteriumcoprostanoligenesgroup	rs12906958	C	T	0.302	-0.053	0.012	4.34803E-06	-0.064	0.059	0.274	22.011
Eubacteriumcoprostanoligenesgroup	rs17159861	C	T	0.133	0.096	0.017	1.03787E-08	-0.085	0.087	0.326	39.291
Eubacteriumcoprostanoligenesgroup	rs2644213	G	A	0.237	0.054	0.012	9.85638E-06	0.014	0.059	0.815	19.255
Eubacteriumcoprostanoligenesgroup	rs4076415	T	G	0.401	0.052	0.011	1.98974E-06	0.089	0.056	0.111	23.401
Eubacteriumcoprostanoligenesgroup	rs62024432	C	T	0.125	-0.077	0.017	7.50207E-06	0.012	0.091	0.893	23.829
Eubacteriumcoprostanoligenesgroup	rs6762473	C	A	0.357	0.052	0.011	4.26113E-06	0.036	0.056	0.520	22.930
Eubacteriumcoprostanoligenesgroup	rs76898927	G	A	0.048	0.123	0.027	4.78979E-06	0.152	0.117	0.197	25.262
Eubacteriumcoprostanoligenesgroup	rs9648214	T	C	0.118	-0.083	0.016	2.5203E-07	0.127	0.095	0.183	26.310
Eubacteriumeligensgroup	rs182318	G	A	0.078	-0.082	0.020	8.39837E-06	-0.021	0.098	0.833	17.862
Eubacteriumeligensgroup	rs2200429	A	G	0.074	-0.089	0.020	5.29721E-06	-0.120	0.091	0.186	19.777

Eubacteriumeligensgroup	rs265534	T	G	0.455	-0.056	0.012	2.26758E-06	0.049	0.054	0.359	28.954
Eubacteriumeligensgroup	rs4583233	A	C	0.334	0.067	0.013	2.83852E-07	0.028	0.059	0.638	36.719
Eubacteriumeligensgroup	rs56080211	C	T	0.056	0.123	0.028	9.1429E-06	-0.075	0.101	0.459	29.276
Eubacteriumeligensgroup	rs6923695	T	G	0.057	0.103	0.023	4.87406E-06	0.074	0.107	0.492	20.943
Eubacteriumfissicatena group	rs10147907	T	G	0.069	0.172	0.040	8.27246E-06	-0.108	0.102	0.289	69.803
Eubacteriumfissicatena group	rs11818408	G	A	0.477	0.106	0.024	8.20015E-06	-0.050	0.055	0.362	103.093
Eubacteriumfissicatena group	rs11876297	T	C	0.216	0.131	0.028	2.66556E-06	0.041	0.061	0.502	107.873
Eubacteriumfissicatena group	rs151257695	A	G	0.081	0.210	0.045	3.09681E-06	0.029	0.104	0.781	119.941
Eubacteriumfissicatena group	rs1768152	T	C	0.137	0.139	0.032	8.70054E-06	-0.073	0.087	0.405	84.865
Eubacteriumfissicatena group	rs2733072	G	A	0.465	0.110	0.023	1.49089E-06	-0.009	0.054	0.874	110.354
Eubacteriumfissicatena group	rs3771393	C	T	0.284	0.131	0.027	7.37909E-07	0.066	0.068	0.327	128.654
Eubacteriumfissicatena group	rs6934739	A	G	0.295	0.111	0.025	9.75326E-06	0.017	0.057	0.770	95.297
Eubacteriumfissicatena group	rs7104872	G	A	0.178	0.139	0.029	2.7315E-06	-0.032	0.085	0.702	103.641

Eubacteriumhalliigroup	rs10501370	C	T	0.053	-0.116	0.025	5.41918E-06	0.093	0.111	0.401	24.496
Eubacteriumhalliigroup	rs10798999	C	T	0.216	0.060	0.013	2.60558E-06	0.044	0.061	0.466	22.488
Eubacteriumhalliigroup	rs117748144	T	C	0.043	-0.127	0.029	7.86002E-06	-0.133	0.120	0.270	24.053
Eubacteriumhalliigroup	rs13116360	T	C	0.063	0.154	0.030	2.94487E-07	-0.073	0.108	0.499	51.267
Eubacteriumhalliigroup	rs17074066	T	C	0.092	-0.081	0.019	9.34905E-06	0.085	0.188	0.652	20.223
Eubacteriumhalliigroup	rs17474256	G	A	0.123	0.081	0.018	9.45445E-06	-0.076	0.090	0.398	26.100
Eubacteriumhalliigroup	rs281379	A	G	0.460	-0.050	0.011	9.32796E-06	0.124	0.054	0.022	22.762
Eubacteriumhalliigroup	rs28584818	A	G	0.069	0.126	0.027	4.43235E-06	0.107	0.099	0.281	37.348
Eubacteriumhalliigroup	rs60254196	A	G	0.541	-0.052	0.011	2.69672E-06	0.027	0.054	0.617	24.931
Eubacteriumhalliigroup	rs630939	C	T	0.400	-0.051	0.011	9.15759E-06	0.034	0.054	0.528	22.818
Eubacteriumhalliigroup	rs6550770	T	C	0.052	-0.198	0.044	4.823E-06	0.062	0.133	0.642	70.828
Eubacteriumhalliigroup	rs74018587	C	T	0.037	0.209	0.044	3.70257E-06	-0.275	0.138	0.047	56.931
Eubacteriumhalliigroup	rs78056098	G	T	0.361	-0.051	0.011	8.28818E-06	0.034	0.056	0.537	21.803
Eubacteriumhalliigroup	rs949971	T	G	0.384	-0.054	0.012	3.29002E-06	0.014	0.057	0.810	25.341
Eubacteriumnodatumgroup	rs10263623	C	T	0.104	0.193	0.044	8.90706E-06	-0.144	0.135	0.287	129.298
Eubacteriumnodatumgroup	rs10458299	T	C	0.081	-0.188	0.042	8.36714E-06	-0.030	0.101	0.769	96.200
Eubacteriumnodatumgroup	rs11006576	A	G	0.513	-0.110	0.025	7.98664E-06	0.069	0.054	0.198	111.887

Eubacteriumnodatumgroup	rs113893692	C	T	0.098	-0.185	0.040	5.75702E-06	-0.018	0.082	0.824	112.172
Eubacteriumnodatumgroup	rs34297067	A	G	0.139	-0.187	0.034	6.59784E-08	0.059	0.076	0.435	154.819
Eubacteriumnodatumgroup	rs61841040	G	T	0.166	0.161	0.034	3.56141E-06	0.032	0.067	0.631	131.940
Eubacteriumnodatumgroup	rs6818880	A	G	0.455	-0.110	0.025	7.8254E-06	-0.009	0.054	0.867	110.886
Eubacteriumnodatumgroup	rs77910827	C	T	0.103	0.202	0.041	9.05381E-07	0.083	0.086	0.338	139.504
Eubacteriumnodatumgroup	rs7827125	C	T	0.282	0.122	0.027	7.17355E-06	0.028	0.060	0.641	111.822
Eubacteriumnodatumgroup	rs7880204	T	C	0.248	-0.125	0.028	6.83884E-06	-0.063	0.062	0.307	108.140
Eubacteriumnodatumgroup	rs9425984	T	C	0.239	-0.130	0.029	7.20681E-06	-0.005	0.065	0.934	113.710
Eubacteriumoxidoreducensgroup	rs12129908	C	A	0.447	0.089	0.020	5.79516E-06	-0.036	0.055	0.513	72.637
Eubacteriumoxidoreducensgroup	rs12423772	G	T	0.150	0.141	0.030	2.62925E-06	0.067	0.076	0.380	93.465
Eubacteriumoxidoreducensgroup	rs2973294	G	T	0.421	0.092	0.020	2.38836E-06	-0.072	0.054	0.186	76.549
Eubacteriumoxidoreducensgroup	rs34561138	G	A	0.059	0.216	0.046	2.51163E-06	-0.196	0.138	0.156	95.015
Eubacteriumoxidoreducensgroup	rs440215	C	T	0.462	0.093	0.020	1.65407E-06	0.060	0.054	0.265	79.664

Eubacteriumrectalegroup	rs10248854	C	A	0.360	-0.053	0.011	4.20676E-06	-0.030	0.055	0.591	23.567
Eubacteriumrectalegroup	rs10797540	A	G	0.434	0.050	0.011	3.53384E-06	0.044	0.054	0.411	22.844
Eubacteriumrectalegroup	rs143694765	T	C	0.094	0.087	0.020	9.75369E-06	-0.043	0.089	0.630	23.764
Eubacteriumrectalegroup	rs2884897	A	G	0.053	-0.129	0.029	6.44426E-06	-0.022	0.146	0.880	30.692
Eubacteriumrectalegroup	rs314726	T	C	0.457	0.053	0.011	1.37967E-06	-0.066	0.054	0.225	25.490
Eubacteriumrectalegroup	rs35398954	A	G	0.121	-0.090	0.017	5.39945E-07	0.079	0.073	0.278	31.818
Eubacteriumrectalegroup	rs59427698	A	G	0.203	-0.058	0.013	5.36906E-06	-0.092	0.068	0.176	19.696
Eubacteriumrectalegroup	rs62547233	A	G	0.307	0.054	0.012	9.8982E-06	0.011	0.059	0.850	22.480
Eubacteriumruminantiumgroup	rs10131724	A	C	0.064	-0.200	0.041	2.38663E-06	0.038	0.091	0.672	87.640
Eubacteriumruminantiumgroup	rs10923018	G	A	0.472	0.073	0.016	6.79541E-06	-0.015	0.054	0.779	48.364
Eubacteriumruminantiumgroup	rs11637981	G	T	0.484	-0.073	0.016	5.43671E-06	0.007	0.054	0.893	49.290
Eubacteriumruminantiumgroup	rs13025464	T	C	0.383	-0.074	0.016	6.96842E-06	-0.033	0.055	0.551	47.193
Eubacteriumruminantiumgroup	rs139749	C	T	0.342	-0.085	0.017	8.58845E-07	0.112	0.057	0.048	59.175

Eubacteriumruminantiu mgroup	rs16891896	G	A	0.059	-0.175	0.039	2.3812E-06	0.032	0.096	0.739	62.021
Eubacteriumruminantiu mgroup	rs17519472	C	T	0.154	0.108	0.023	4.70356E-06	-0.025	0.078	0.749	55.730
Eubacteriumruminantiu mgroup	rs209813	G	A	0.166	-0.103	0.024	9.23146E-06	0.014	0.076	0.853	54.541
Eubacteriumruminantiu mgroup	rs2116427	A	G	0.257	0.091	0.018	4.67364E-07	-0.165	0.062	0.008	58.291
Eubacteriumruminantiu mgroup	rs2229917	A	G	0.067	0.154	0.032	2.15894E-06	0.077	0.133	0.564	53.905
Eubacteriumruminantiu mgroup	rs2418654	C	T	0.478	-0.075	0.017	6.17247E-06	-0.007	0.055	0.902	51.467
Eubacteriumruminantiu mgroup	rs2817174	C	T	0.429	-0.073	0.016	7.8696E-06	0.029	0.055	0.602	48.582
Eubacteriumruminantiu mgroup	rs57340348	T	C	0.215	-0.098	0.021	4.93278E-06	0.041	0.067	0.539	59.512
Eubacteriumruminantiu mgroup	rs606117	A	G	0.332	0.083	0.018	4.81639E-06	-0.026	0.060	0.664	56.647
Eubacteriumruminantiu mgroup	rs6676699	G	T	0.222	-0.089	0.020	6.37979E-06	0.013	0.059	0.822	50.052
Eubacteriumruminantiu mgroup	rs7000472	A	G	0.362	-0.076	0.017	4.06848E-06	0.067	0.055	0.222	49.341
Eubacteriumruminantiu mgroup	rs72836424	C	T	0.095	-0.140	0.030	2.62125E-06	-0.004	0.086	0.963	62.090
Eubacteriumruminantiu mgroup	rs73139629	A	C	0.132	-0.115	0.025	5.36199E-06	0.039	0.093	0.677	55.910

Eubacteriumventriosumgroup	rs11617697	A	G	0.048	-0.143	0.029	7.21757E-07	0.037	0.118	0.751	34.298
Eubacteriumventriosumgroup	rs12964517	G	A	0.316	0.059	0.012	2.07499E-06	0.010	0.060	0.873	27.379
Eubacteriumventriosumgroup	rs13082419	C	T	0.170	-0.072	0.016	9.55987E-06	0.032	0.085	0.704	26.533
Eubacteriumventriosumgroup	rs16884680	G	T	0.107	-0.091	0.019	1.73969E-06	0.051	0.088	0.566	28.933
Eubacteriumventriosumgroup	rs35179274	C	T	0.244	-0.063	0.014	5.75792E-06	0.036	0.069	0.602	26.621
Eubacteriumventriosumgroup	rs3809430	T	C	0.348	-0.055	0.012	3.55317E-06	-0.047	0.058	0.417	25.037
Eubacteriumventriosumgroup	rs66746423	C	T	0.156	0.075	0.016	6.11142E-06	0.062	0.074	0.401	27.326
Eubacteriumventriosumgroup	rs6704822	A	G	0.116	0.074	0.017	6.6223E-06	0.018	0.080	0.823	20.618
Eubacteriumventriosumgroup	rs72783037	C	A	0.187	0.066	0.014	6.54762E-06	-0.028	0.066	0.673	24.232
Eubacteriumventriosumgroup	rs73615400	T	C	0.091	-0.096	0.019	9.53613E-07	0.007	0.091	0.943	27.649
Eubacteriumventriosumgroup	rs73849225	T	C	0.080	0.098	0.022	5.20909E-06	0.034	0.097	0.729	25.587
Eubacteriumventriosumgroup	rs78250280	G	A	0.149	0.075	0.016	3.35791E-06	-0.024	0.076	0.751	26.194
Eubacteriumventriosumgroup	rs876734	C	T	0.289	-0.062	0.013	2.88908E-06	0.023	0.060	0.696	28.911

Eubacteriumventriosumgroup	rs9316536	T	G	0.136	-0.082	0.018	7.83519E-06	-0.027	0.078	0.731	28.855
Eubacteriumxylanophilumgroup	rs10140184	A	C	0.413	0.058	0.013	4.95862E-06	0.020	0.054	0.716	29.618
Eubacteriumxylanophilumgroup	rs10917203	A	C	0.335	0.061	0.013	3.15155E-06	-0.063	0.055	0.252	30.717
Eubacteriumxylanophilumgroup	rs112176119	C	T	0.065	-0.113	0.025	3.32766E-06	-0.033	0.092	0.723	28.572
Eubacteriumxylanophilumgroup	rs13239072	G	A	0.275	0.069	0.014	1.81783E-06	-0.065	0.060	0.276	34.643
Eubacteriumxylanophilumgroup	rs17830032	G	A	0.054	-0.161	0.031	2.38893E-07	-0.020	0.099	0.840	48.195
Eubacteriumxylanophilumgroup	rs1999224	G	T	0.089	-0.095	0.020	3.75492E-06	-0.099	0.090	0.274	26.687
Eubacteriumxylanophilumgroup	rs2012708	A	G	0.364	0.057	0.013	6.5254E-06	-0.024	0.056	0.665	27.933
Eubacteriumxylanophilumgroup	rs2213117	T	G	0.161	0.088	0.019	4.21329E-06	-0.105	0.074	0.153	38.253
Eubacteriumxylanophilumgroup	rs75586835	A	G	0.067	-0.114	0.026	9.38708E-06	-0.139	0.105	0.186	29.938
Faecalibacterium	rs10927394	G	T	0.027	-0.232	0.051	7.01885E-06	0.017	0.197	0.930	51.749
Faecalibacterium	rs114946999	C	T	0.098	-0.086	0.019	5.69574E-06	-0.094	0.081	0.243	24.185
Faecalibacterium	rs11776390	T	C	0.090	-0.078	0.017	6.39675E-06	0.026	0.108	0.809	18.367
Faecalibacterium	rs1271565	C	T	0.272	-0.058	0.012	1.30169E-06	-0.041	0.061	0.506	24.170
Faecalibacterium	rs12753492	A	C	0.149	0.064	0.015	8.80273E-06	0.029	0.085	0.736	19.157
Faecalibacterium	rs2835874	T	C	0.070	-0.087	0.020	7.54405E-06	0.221	0.142	0.120	17.843

Faecalibacterium	rs6910935	A	G	0.054	0.135	0.028	1.37848E-06	0.044	0.112	0.692	33.961
Faecalibacterium	rs75499067	C	T	0.064	0.228	0.047	1.76448E-06	0.052	0.103	0.611	113.835
Faecalibacterium	rs79656633	T	C	0.105	0.146	0.032	8.13557E-06	-0.055	0.090	0.544	73.637
Faecalibacterium	rs9536330	T	C	0.409	-0.048	0.011	5.33258E-06	-0.005	0.054	0.921	20.712
FamilyXIIIAD3011group	rs11126423	C	T	0.113	0.090	0.020	5.91249E-06	-0.050	0.094	0.591	30.187
FamilyXIIIAD3011group	rs11736617	G	A	0.120	-0.076	0.017	9.02048E-06	-0.019	0.117	0.873	22.416
FamilyXIIIAD3011group	rs12812672	T	C	0.074	-0.096	0.021	2.55807E-06	-0.107	0.103	0.302	23.107
FamilyXIIIAD3011group	rs149302	T	C	0.213	-0.065	0.014	7.4818E-06	-0.063	0.063	0.319	25.637
FamilyXIIIAD3011group	rs16840310	A	G	0.372	-0.061	0.012	6.75113E-07	-0.001	0.054	0.985	31.726
FamilyXIIIAD3011group	rs16940167	C	T	0.169	0.073	0.016	3.90837E-06	-0.065	0.068	0.343	27.682
FamilyXIIIAD3011group	rs17156849	G	A	0.063	-0.113	0.025	4.19171E-06	0.018	0.113	0.871	27.469
FamilyXIIIAD3011group	rs62029761	A	G	0.068	0.129	0.028	3.89083E-06	0.130	0.118	0.272	38.402
FamilyXIIIAD3011group	rs62200412	C	T	0.171	-0.080	0.016	5.80219E-07	-0.062	0.062	0.313	33.406
FamilyXIIIAD3011group	rs72730932	C	A	0.164	-0.090	0.018	6.88766E-07	0.010	0.092	0.912	40.781
FamilyXIIIAD3011group	rs739451	C	T	0.176	0.065	0.015	7.8781E-06	0.009	0.066	0.891	22.461

FamilyXIIIAD3011group	rs9276029	A	G	0.168	-0.081	0.019	8.92766E-06	0.038	0.067	0.573	33.812
FamilyXIIIAD3011group	rs9837139	A	G	0.082	0.108	0.024	8.70808E-06	-0.083	0.096	0.386	31.794
FamilyXIIIUCG001	rs112362903	A	G	0.043	-0.149	0.033	7.8777E-06	-0.131	0.150	0.384	33.365
FamilyXIIIUCG001	rs12049454	T	C	0.301	-0.065	0.013	1.17378E-06	0.034	0.055	0.541	32.401
FamilyXIIIUCG001	rs1426266	T	C	0.296	-0.067	0.014	1.24935E-06	0.009	0.060	0.888	33.927
FamilyXIIIUCG001	rs3842897	G	A	0.065	-0.113	0.024	5.19705E-06	-0.119	0.094	0.206	28.162
FamilyXIIIUCG001	rs62414802	C	T	0.298	-0.061	0.013	4.29181E-06	0.056	0.062	0.360	28.784
FamilyXIIIUCG001	rs7119679	G	A	0.150	-0.081	0.017	3.52494E-06	0.089	0.063	0.158	30.679
FamilyXIIIUCG001	rs76463770	A	G	0.052	0.193	0.042	3.77476E-06	0.255	0.154	0.098	67.314
FamilyXIIIUCG001	rs8076666	A	G	0.136	0.089	0.020	8.0183E-06	0.037	0.082	0.655	33.977
Flavonifractor	rs114873521	C	T	0.077	-0.130	0.029	7.12522E-06	-0.076	0.104	0.465	43.939
Flavonifractor	rs11811696	T	C	0.110	-0.116	0.024	2.07346E-06	0.022	0.098	0.821	48.611
Flavonifractor	rs12030302	A	G	0.491	-0.069	0.014	5.6084E-07	0.016	0.054	0.766	44.034
Flavonifractor	rs34066017	A	G	0.246	0.076	0.016	1.52086E-06	-0.170	0.066	0.010	39.770
Flavonifractor	rs806808	T	C	0.476	0.067	0.014	1.17972E-06	-0.020	0.054	0.718	40.823
Fusicatenibacter	rs10439674	A	G	0.202	-0.057	0.013	7.68485E-06	0.026	0.067	0.701	19.358
Fusicatenibacter	rs167879	C	T	0.167	-0.066	0.015	5.86893E-06	-0.013	0.075	0.860	22.221
Fusicatenibacter	rs1864685	A	C	0.465	-0.049	0.011	4.96161E-06	0.010	0.054	0.854	22.367
Fusicatenibacter	rs2025938	G	A	0.077	-0.097	0.021	2.98832E-06	0.154	0.107	0.151	24.276
Fusicatenibacter	rs206581	A	G	0.209	-0.057	0.013	8.95756E-06	0.065	0.065	0.316	19.585
Fusicatenibacter	rs2132128	G	A	0.165	-0.077	0.016	1.07686E-06	0.007	0.089	0.937	30.158
Fusicatenibacter	rs3303	T	C	0.077	-0.095	0.020	3.93891E-06	-0.014	0.109	0.894	23.595
Fusicatenibacter	rs4378146	A	C	0.242	-0.062	0.013	7.20451E-07	0.051	0.061	0.407	25.593
Fusicatenibacter	rs60254196	A	G	0.541	-0.049	0.011	5.46739E-06	0.027	0.054	0.617	22.112

Fusicatenibacter	rs62187631	T	C	0.140	-0.071	0.016	4.54526E-06	-0.045	0.068	0.507	22.350
Fusicatenibacter	rs62353480	A	G	0.171	-0.070	0.015	1.56768E-06	-0.082	0.072	0.250	25.613
Fusicatenibacter	rs6515626	G	A	0.062	0.142	0.031	7.28654E-06	-0.083	0.107	0.442	42.592
Fusicatenibacter	rs704418	T	C	0.186	0.074	0.015	7.77428E-07	-0.112	0.081	0.167	30.373
Fusicatenibacter	rs73103914	A	G	0.178	-0.060	0.013	8.29563E-06	-0.106	0.075	0.158	19.158
Fusicatenibacter	rs792108	T	C	0.354	-0.051	0.011	8.49535E-06	0.048	0.054	0.379	21.697
Fusicatenibacter	rs8028026	A	G	0.092	-0.079	0.018	8.0633E-06	-0.220	0.092	0.017	19.151
Fusicatenibacter	rs8063430	T	C	0.060	-0.104	0.022	4.9289E-06	-0.174	0.122	0.154	22.272
Fusicatenibacter	rs9905659	G	A	0.188	-0.062	0.014	7.30993E-06	0.023	0.069	0.741	21.273
Gordonibacter	rs13412653	A	C	0.354	0.108	0.024	8.61141E-06	-0.004	0.055	0.936	97.605
Gordonibacter	rs16955299	G	A	0.084	-0.196	0.043	6.37019E-06	-0.053	0.090	0.560	108.932
Gordonibacter	rs322296	G	A	0.119	0.179	0.038	4.02422E-06	-0.048	0.102	0.636	123.872
Gordonibacter	rs35042269	C	A	0.189	-0.180	0.040	8.10852E-06	-0.067	0.084	0.426	184.498
Gordonibacter	rs3765837	T	G	0.079	-0.191	0.043	7.16906E-06	-0.074	0.104	0.474	97.022
Gordonibacter	rs4596722	A	G	0.476	0.103	0.023	9.0568E-06	0.077	0.054	0.149	97.393
Gordonibacter	rs71545975	A	G	0.173	-0.154	0.034	7.03944E-06	-0.120	0.071	0.092	125.249
Gordonibacter	rs72714787	C	A	0.104	0.181	0.038	1.42889E-06	0.133	0.079	0.094	113.547
Gordonibacter	rs72939513	A	G	0.054	-0.214	0.049	7.98199E-06	-0.024	0.122	0.846	85.743
Gordonibacter	rs7294633	C	T	0.351	0.129	0.025	3.43763E-07	0.004	0.060	0.942	139.354
Gordonibacter	rs768830	G	A	0.129	0.150	0.033	7.75583E-06	-0.019	0.074	0.797	93.130
Haemophilus	rs10781340	G	A	0.166	0.095	0.020	4.32266E-06	0.047	0.081	0.561	45.835
Haemophilus	rs111582866	G	A	0.101	-0.124	0.026	1.27347E-06	-0.019	0.095	0.837	51.750
Haemophilus	rs35509	G	A	0.079	0.128	0.027	2.00952E-06	-0.163	0.133	0.222	43.741
Haemophilus	rs4822728	T	C	0.437	0.071	0.015	3.47849E-06	-0.027	0.053	0.614	45.078
Haemophilus	rs76022354	C	T	0.033	0.245	0.051	1.83031E-06	-0.288	0.123	0.019	69.899
Haemophilus	rs78909003	T	C	0.050	-0.246	0.050	1.66745E-06	-0.125	0.118	0.288	105.649

Haemophilus	rs9328464	T	C	0.492	0.072	0.015	1.42269E-06	-0.079	0.054	0.144	48.055
Haemophilus	rs9382510	C	T	0.246	-0.094	0.017	7.12051E-08	0.065	0.061	0.292	59.610
Haemophilus	rs9895850	T	C	0.050	-0.193	0.042	2.1406E-06	0.009	0.132	0.947	64.722
Holdemanella	rs12513188	G	A	0.222	0.090	0.020	4.6548E-06	-0.015	0.061	0.800	51.852
Holdemanella	rs17586763	T	C	0.018	-0.227	0.051	7.71949E-06	-0.087	0.121	0.473	33.367
Holdemanella	rs1926302	G	A	0.153	-0.108	0.023	7.50141E-06	0.054	0.065	0.403	55.606
Holdemanella	rs34187114	C	A	0.166	-0.105	0.023	5.13495E-06	-0.286	0.090	0.001	55.622
Holdemanella	rs35228298	G	A	0.247	0.093	0.020	7.29849E-06	0.048	0.073	0.515	59.726
Holdemanella	rs4541991	T	C	0.236	-0.093	0.019	2.10094E-06	0.017	0.057	0.766	56.977
Holdemanella	rs607782	T	C	0.382	-0.085	0.017	7.19447E-07	0.067	0.056	0.226	63.378
Holdemanella	rs62113381	T	C	0.159	-0.105	0.023	5.53879E-06	-0.012	0.079	0.884	54.694
Holdemanella	rs73011279	T	C	0.208	-0.096	0.020	1.36046E-06	0.045	0.064	0.480	56.006
Holdemanella	rs75764681	T	C	0.063	-0.283	0.060	1.94273E-06	0.160	0.131	0.222	174.130
Holdemanella	rs8113760	G	A	0.352	0.079	0.017	4.62395E-06	0.082	0.058	0.157	52.351
Holdemania	rs10885477	T	C	0.062	-0.135	0.030	8.59833E-06	0.073	0.122	0.549	38.798
Holdemania	rs11080063	G	A	0.466	-0.067	0.015	6.66887E-06	-0.032	0.054	0.558	40.480
Holdemania	rs111745969	A	G	0.080	0.121	0.027	3.70776E-06	-0.021	0.078	0.792	39.169
Holdemania	rs113593397	A	G	0.102	-0.129	0.028	9.35728E-06	0.053	0.091	0.559	56.213
Holdemania	rs116500994	G	T	0.052	-0.138	0.029	2.34479E-06	0.124	0.128	0.334	34.084
Holdemania	rs12701617	A	G	0.458	-0.066	0.015	9.51844E-06	-0.048	0.054	0.376	39.823
Holdemania	rs1867876	T	C	0.294	0.084	0.016	2.7388E-07	0.100	0.059	0.088	54.270
Holdemania	rs4146507	C	T	0.222	0.079	0.018	7.22973E-06	0.019	0.062	0.758	40.072
Holdemania	rs73139538	G	A	0.053	-0.149	0.033	7.77178E-06	0.069	0.159	0.667	40.517
Holdemania	rs77293403	A	G	0.046	0.165	0.034	1.77466E-06	0.011	0.144	0.941	43.415
Holdemania	rs80149660	C	T	0.059	-0.233	0.052	6.04217E-06	0.037	0.132	0.782	110.491
Holdemania	rs9500080	C	T	0.235	0.093	0.018	4.08515E-07	0.088	0.071	0.217	56.739

Holdemania	rs9529719	T	C	0.325	0.074	0.016	5.96669E-06	-0.001	0.057	0.988	44.210
Holdemania	rs967319	T	C	0.247	0.079	0.018	8.38155E-06	-0.015	0.063	0.806	42.466
Howardella	rs10048062	C	T	0.107	-0.147	0.034	8.58873E-06	-0.200	0.093	0.032	76.660
Howardella	rs12452946	A	G	0.501	-0.106	0.023	3.80393E-06	0.130	0.054	0.015	103.277
Howardella	rs1484873	A	G	0.069	-0.228	0.046	2.55918E-06	0.060	0.075	0.425	122.450
Howardella	rs17167098	G	A	0.113	-0.169	0.035	1.12323E-06	0.026	0.078	0.743	106.304
Howardella	rs2154047	C	A	0.068	-0.193	0.042	9.97042E-06	-0.149	0.094	0.113	86.116
Howardella	rs36081916	T	C	0.111	-0.181	0.040	4.7004E-06	-0.071	0.096	0.455	119.928
Howardella	rs3791893	A	G	0.123	0.147	0.034	9.49716E-06	0.061	0.077	0.432	86.114
Howardella	rs609430	T	G	0.339	-0.112	0.024	3.34484E-06	0.048	0.056	0.392	103.758
Howardella	rs672217	G	A	0.141	0.164	0.035	3.52468E-06	-0.120	0.068	0.077	120.619
Hungatella	rs10044993	C	A	0.117	0.140	0.032	8.06668E-06	0.030	0.096	0.753	74.249
Hungatella	rs13128780	T	C	0.145	-0.150	0.031	1.74998E-06	-0.130	0.068	0.057	102.560
Hungatella	rs13249325	T	G	0.367	-0.100	0.023	9.69162E-06	0.070	0.054	0.191	85.619
Hungatella	rs17092615	G	A	0.124	0.152	0.034	7.37677E-06	0.194	0.079	0.014	92.989
Hungatella	rs72759041	G	T	0.235	-0.126	0.028	3.86095E-06	-0.023	0.066	0.729	105.195
Intestinibacter	rs10805326	G	A	0.278	0.078	0.014	3.54544E-08	0.010	0.059	0.861	44.368
Intestinibacter	rs11109097	C	T	0.312	0.062	0.014	5.49484E-06	0.041	0.054	0.446	30.738
Intestinibacter	rs118030283	G	A	0.038	-0.152	0.032	2.67439E-06	-0.079	0.127	0.536	30.802
Intestinibacter	rs16938435	T	C	0.061	-0.112	0.024	1.80097E-06	0.025	0.090	0.780	26.316
Intestinibacter	rs2098844	C	T	0.425	-0.058	0.013	6.78879E-06	-0.003	0.056	0.953	29.702
Intestinibacter	rs2702387	A	G	0.352	0.061	0.013	4.25994E-06	-0.079	0.054	0.149	31.030
Intestinibacter	rs4327025	G	A	0.195	-0.081	0.015	1.64326E-07	0.039	0.069	0.574	37.854
Intestinibacter	rs447950	A	G	0.299	0.063	0.014	5.63911E-06	-0.011	0.055	0.842	30.412
Intestinibacter	rs478972	T	C	0.060	-0.143	0.030	1.81991E-06	0.054	0.094	0.566	41.944
Intestinibacter	rs6062862	A	G	0.094	0.092	0.020	6.67984E-06	0.105	0.098	0.282	26.838

Intestinibacter	rs62430350	T	C	0.058	0.151	0.035	6.84229E-06	-0.120	0.142	0.397	45.638
Intestinibacter	rs68093214	C	T	0.258	0.066	0.015	9.25769E-06	-0.014	0.062	0.819	30.806
Intestinibacter	rs6875660	C	T	0.123	0.089	0.019	3.05781E-06	-0.095	0.112	0.397	31.489
Intestinibacter	rs893394	G	A	0.420	0.058	0.013	7.85038E-06	-0.023	0.055	0.670	30.441
Intestinibacter	rs9348442	C	T	0.088	0.099	0.022	6.25936E-06	-0.076	0.081	0.351	28.793
Intestinimonas	rs10262702	T	C	0.129	0.092	0.019	2.06073E-06	-0.045	0.082	0.582	34.841
Intestinimonas	rs11258178	A	G	0.415	0.066	0.013	6.98195E-07	-0.014	0.054	0.793	38.942
Intestinimonas	rs12226153	A	G	0.062	-0.151	0.031	5.12171E-07	0.307	0.223	0.170	48.559
Intestinimonas	rs17067892	C	T	0.077	0.107	0.025	6.37926E-06	0.010	0.095	0.918	29.821
Intestinimonas	rs1859797	G	A	0.504	0.060	0.013	4.12235E-06	-0.045	0.054	0.405	33.473
Intestinimonas	rs2276760	A	G	0.220	-0.069	0.015	7.84062E-06	0.055	0.063	0.377	29.570
Intestinimonas	rs2731794	C	T	0.060	0.121	0.026	1.91744E-06	-0.195	0.145	0.179	29.962
Intestinimonas	rs2930225	G	T	0.258	0.073	0.015	1.34622E-06	-0.054	0.063	0.396	37.481
Intestinimonas	rs4113676	A	C	0.062	-0.219	0.049	7.41635E-06	0.019	0.232	0.934	101.785
Intestinimonas	rs4784055	T	C	0.056	-0.175	0.039	8.71812E-07	0.026	0.122	0.834	59.489
Intestinimonas	rs62240188	G	A	0.069	0.130	0.027	2.20308E-06	-0.002	0.092	0.984	39.734
Intestinimonas	rs6934519	C	T	0.275	0.069	0.015	8.56956E-06	-0.102	0.061	0.096	35.145
Intestinimonas	rs716604	A	G	0.188	0.082	0.017	8.5663E-07	-0.170	0.065	0.008	37.531
Intestinimonas	rs7170984	T	C	0.290	-0.066	0.014	2.98356E-06	-0.145	0.060	0.015	32.786
Intestinimonas	rs72982915	C	T	0.046	0.183	0.040	4.91157E-06	-0.072	0.117	0.537	53.815
Intestinimonas	rs9823439	T	C	0.441	-0.058	0.013	9.85876E-06	-0.042	0.054	0.433	30.677
Lachnoclostridium	rs1031599	G	T	0.085	-0.079	0.018	6.31376E-06	0.199	0.109	0.067	17.557
Lachnoclostridium	rs12566975	T	C	0.493	-0.047	0.011	9.57204E-06	0.074	0.054	0.169	20.109
Lachnoclostridium	rs1528479	G	A	0.370	-0.050	0.011	9.63976E-06	-0.010	0.055	0.858	21.205
Lachnoclostridium	rs1997204	T	C	0.053	-0.108	0.024	5.97081E-06	0.220	0.129	0.089	21.411
Lachnoclostridium	rs2385421	A	G	0.104	0.075	0.018	7.13717E-06	-0.119	0.083	0.152	19.114

Lachnoclostridium	rs3821998	C	A	0.089	-0.086	0.019	6.72052E-06	0.003	0.088	0.977	22.116
Lachnoclostridium	rs4738679	G	A	0.365	-0.052	0.011	4.41754E-06	-0.056	0.055	0.306	23.033
Lachnoclostridium	rs6112314	A	C	0.397	-0.056	0.011	2.43212E-07	-0.006	0.057	0.918	27.735
Lachnoclostridium	rs615997	T	C	0.499	0.051	0.011	2.02677E-06	-0.006	0.053	0.910	24.044
Lachnoclostridium	rs62285313	A	G	0.086	0.086	0.018	1.58331E-06	0.011	0.090	0.907	21.442
Lachnoclostridium	rs72829893	G	T	0.066	0.117	0.027	5.57766E-06	0.030	0.088	0.733	31.076
Lachnoclostridium	rs78068103	A	G	0.092	0.089	0.019	3.66519E-06	-0.015	0.084	0.863	23.975
Lachnoclostridium	rs789029	C	T	0.189	-0.064	0.014	3.75328E-06	-0.051	0.077	0.510	23.139
Lachnospira	rs13157098	A	G	0.167	-0.077	0.016	5.99315E-07	-0.120	0.073	0.100	30.147
Lachnospira	rs159484	G	A	0.113	0.079	0.018	6.67622E-06	-0.123	0.103	0.231	23.298
Lachnospira	rs2520509	A	G	0.318	0.052	0.012	7.41854E-06	-0.018	0.059	0.755	21.459
Lachnospira	rs4686798	T	C	0.386	0.053	0.011	2.73936E-06	-0.076	0.056	0.173	24.611
Lachnospira	rs4923324	G	A	0.239	-0.062	0.013	2.43593E-06	0.145	0.073	0.046	25.427
Lachnospira	rs56791201	T	C	0.457	0.052	0.011	2.92823E-06	-0.014	0.056	0.806	24.478
LachnospiraceaeFCS020group	rs10093861	G	A	0.498	-0.057	0.012	3.05624E-06	-0.092	0.054	0.093	29.720
LachnospiraceaeFCS020group	rs1254846	G	A	0.092	0.106	0.023	5.59664E-06	0.050	0.077	0.513	34.296
LachnospiraceaeFCS020group	rs1363769	T	C	0.033	-0.201	0.045	1.58163E-06	0.077	0.149	0.605	46.953
LachnospiraceaeFCS020group	rs2322265	C	T	0.256	-0.067	0.014	5.20809E-06	-0.068	0.061	0.262	31.025
LachnospiraceaeFCS020group	rs2862811	T	C	0.454	0.056	0.012	3.91911E-06	-0.088	0.058	0.132	29.064
LachnospiraceaeFCS020group	rs35035870	T	C	0.036	-0.191	0.041	2.62028E-06	0.055	0.137	0.685	46.115

LachnospiraceaeFCS020group	rs3999074	G	T	0.478	-0.055	0.012	6.55048E-06	-0.029	0.054	0.590	27.784
LachnospiraceaeFCS020group	rs4452603	T	G	0.286	0.060	0.014	8.9819E-06	0.011	0.061	0.854	27.399
LachnospiraceaeFCS020group	rs7249113	G	A	0.281	0.068	0.013	3.72097E-07	-0.064	0.059	0.274	34.298
LachnospiraceaeFCS020group	rs72793667	A	G	0.084	-0.117	0.025	1.63421E-06	0.118	0.137	0.388	38.423
LachnospiraceaeFCS020group	rs9308097	A	G	0.452	0.055	0.012	7.4729E-06	-0.014	0.054	0.795	27.908
LachnospiraceaeFCS020group	rs9788306	C	T	0.284	-0.063	0.013	1.38618E-06	-0.159	0.060	0.008	29.482
LachnospiraceaeNC2004group	rs117467633	T	C	0.063	-0.170	0.038	9.13317E-06	-0.028	0.137	0.838	62.180
LachnospiraceaeNC2004group	rs12127733	G	A	0.178	0.115	0.025	3.10594E-06	-0.019	0.070	0.786	71.439
LachnospiraceaeNC2004group	rs12208226	C	A	0.106	-0.155	0.034	9.7484E-06	0.040	0.087	0.644	83.878
LachnospiraceaeNC2004group	rs12863463	G	A	0.055	-0.156	0.035	6.03984E-06	-0.120	0.100	0.232	46.485
LachnospiraceaeNC2004group	rs17067076	G	A	0.077	-0.155	0.035	5.61206E-06	-0.100	0.084	0.231	62.165
LachnospiraceaeNC2004group	rs1928659	T	C	0.250	0.103	0.023	6.17241E-06	0.051	0.067	0.447	72.469
LachnospiraceaeNC2004group	rs1929743	T	C	0.312	0.084	0.019	9.0551E-06	0.021	0.057	0.710	55.357

LachnospiraceaeNC2004group	rs3756315	A	G	0.339	-0.088	0.019	3.32583E-06	0.045	0.059	0.442	64.371
LachnospiraceaeNC2004group	rs6116753	G	A	0.240	0.099	0.021	2.9225E-06	-0.035	0.070	0.611	66.360
LachnospiraceaeND3007group	rs2861203	G	A	0.319	0.057	0.013	7.36931E-06	-0.100	0.059	0.088	26.138
LachnospiraceaeND3007group	rs72776675	T	C	0.175	-0.065	0.015	8.71689E-06	0.002	0.071	0.979	22.197
LachnospiraceaeND3007group	rs9932954	A	G	0.428	-0.056	0.012	1.24882E-06	0.016	0.057	0.779	28.396
LachnospiraceaeNK4A136group	rs10952110	G	T	0.448	0.049	0.011	9.08113E-06	0.000	0.054	1.000	21.602
LachnospiraceaeNK4A136group	rs11263806	A	G	0.278	-0.052	0.012	5.06517E-06	0.015	0.056	0.790	20.296
LachnospiraceaeNK4A136group	rs12611395	A	G	0.090	-0.090	0.020	5.83039E-06	-0.039	0.087	0.654	24.377
LachnospiraceaeNK4A136group	rs160061	A	G	0.468	0.051	0.011	2.12198E-06	-0.057	0.054	0.287	24.142
LachnospiraceaeNK4A136group	rs28540839	A	C	0.487	0.051	0.011	9.34319E-06	-0.102	0.054	0.056	23.703
LachnospiraceaeNK4A136group	rs2880566	T	C	0.188	0.060	0.013	5.61428E-06	-0.053	0.076	0.487	20.141
LachnospiraceaeNK4A136group	rs4955932	T	C	0.396	-0.049	0.011	7.05426E-06	-0.059	0.055	0.286	21.276
LachnospiraceaeNK4A136group	rs59805249	T	C	0.091	0.094	0.021	9.45385E-06	-0.051	0.093	0.581	26.495

LachnospiraceaeNK4A136group	rs68104925	T	C	0.371	-0.055	0.012	2.36779E-06	-0.045	0.058	0.440	25.836
LachnospiraceaeNK4A136group	rs7073658	T	G	0.405	-0.050	0.011	5.2691E-06	-0.027	0.054	0.614	22.081
LachnospiraceaeNK4A136group	rs73044693	A	G	0.067	-0.108	0.023	3.57256E-06	0.007	0.105	0.944	26.425
LachnospiraceaeNK4A136group	rs7616165	G	T	0.037	-0.231	0.048	2.77391E-06	-0.117	0.167	0.485	69.356
LachnospiraceaeNK4A136group	rs76193507	A	G	0.057	-0.230	0.050	2.9309E-06	-0.023	0.096	0.814	104.114
LachnospiraceaeNK4A136group	rs7832116	A	G	0.146	-0.071	0.015	3.57084E-06	0.116	0.080	0.147	23.405
LachnospiraceaeNK4A136group	rs954878	A	G	0.450	-0.052	0.011	1.78203E-06	-0.043	0.056	0.443	24.643
LachnospiraceaeUCG001	rs12131224	C	T	0.116	0.117	0.026	7.39863E-06	0.080	0.086	0.354	51.829
LachnospiraceaeUCG001	rs2050911	G	A	0.304	0.075	0.015	1.10648E-06	-0.042	0.057	0.454	43.942
LachnospiraceaeUCG001	rs2371284	T	C	0.223	-0.076	0.017	7.55967E-06	-0.078	0.064	0.223	36.928
LachnospiraceaeUCG001	rs437876	T	C	0.373	0.078	0.014	7.1655E-08	0.109	0.056	0.052	52.948
LachnospiraceaeUCG001	rs4981345	T	C	0.351	-0.068	0.015	6.08596E-06	0.056	0.057	0.328	38.934
LachnospiraceaeUCG001	rs573933	T	C	0.102	-0.108	0.023	3.10508E-06	-0.019	0.087	0.831	39.330

LachnospiraceaeUCG001	rs62496417	T	G	0.271	-0.075	0.017	5.87504E-06	-0.083	0.065	0.205	40.722
LachnospiraceaeUCG001	rs7341608	T	C	0.190	-0.078	0.018	9.48124E-06	0.131	0.078	0.094	34.813
LachnospiraceaeUCG001	rs74034332	G	A	0.046	0.168	0.038	3.33351E-06	0.047	0.112	0.673	45.280
LachnospiraceaeUCG001	rs78848836	A	G	0.084	-0.119	0.026	3.38171E-06	0.110	0.088	0.213	39.744
LachnospiraceaeUCG001	rs8104225	A	G	0.149	0.089	0.020	8.04216E-06	-0.021	0.065	0.750	37.106
LachnospiraceaeUCG001	rs9403580	C	T	0.109	0.108	0.023	3.46785E-06	0.140	0.079	0.077	41.587
LachnospiraceaeUCG001	rs985416	C	T	0.206	0.097	0.018	1.45591E-07	0.073	0.070	0.292	56.607
LachnospiraceaeUCG004	rs11128180	A	G	0.224	0.065	0.014	4.51737E-06	-0.030	0.063	0.639	26.807
LachnospiraceaeUCG004	rs12072562	T	C	0.052	0.133	0.030	7.0737E-06	-0.035	0.137	0.799	31.912
LachnospiraceaeUCG004	rs12673420	G	A	0.441	0.055	0.012	2.98079E-06	0.046	0.053	0.387	27.832
LachnospiraceaeUCG004	rs12747809	G	A	0.340	-0.062	0.013	8.64875E-07	-0.019	0.059	0.754	31.893
LachnospiraceaeUCG004	rs12894272	A	G	0.300	0.058	0.013	4.33949E-06	-0.089	0.056	0.114	25.937
LachnospiraceaeUCG004	rs233486	A	G	0.124	-0.080	0.018	6.28457E-06	-0.150	0.077	0.052	25.527

LachnospiraceaeUCG004	rs2444793	C	T	0.470	-0.054	0.012	4.77076E-06	-0.039	0.055	0.473	26.922
LachnospiraceaeUCG004	rs2726805	A	G	0.409	0.055	0.012	6.30399E-06	0.022	0.054	0.679	26.690
LachnospiraceaeUCG004	rs2882478	G	A	0.455	-0.058	0.012	1.20811E-06	0.063	0.054	0.243	30.342
LachnospiraceaeUCG004	rs35182105	A	G	0.073	-0.110	0.024	4.86508E-06	0.060	0.118	0.613	29.778
LachnospiraceaeUCG004	rs6656451	C	T	0.439	-0.054	0.012	5.56781E-06	-0.048	0.054	0.366	26.735
LachnospiraceaeUCG004	rs7629954	A	G	0.069	0.108	0.024	5.77222E-06	0.138	0.129	0.283	27.598
LachnospiraceaeUCG008	rs10741777	T	C	0.261	-0.097	0.019	7.68659E-07	-0.070	0.058	0.226	67.401
LachnospiraceaeUCG008	rs10793103	C	T	0.304	0.097	0.018	9.34937E-08	0.083	0.054	0.122	73.994
LachnospiraceaeUCG008	rs10801803	G	A	0.162	-0.117	0.024	1.40029E-06	0.030	0.077	0.699	68.446
LachnospiraceaeUCG008	rs13024781	T	C	0.503	-0.080	0.017	2.29166E-06	-0.001	0.054	0.984	58.686
LachnospiraceaeUCG008	rs57091572	A	G	0.140	-0.110	0.024	2.85822E-06	0.010	0.079	0.902	54.085
LachnospiraceaeUCG008	rs61944774	A	G	0.058	0.180	0.039	6.34122E-06	-0.046	0.120	0.703	64.720
LachnospiraceaeUCG008	rs62277846	C	T	0.236	0.102	0.021	1.58892E-06	-0.072	0.068	0.291	69.375

LachnospiraceaeUCG008	rs67078837	T	C	0.458	-0.085	0.017	7.68189E-07	0.021	0.054	0.696	65.376
LachnospiraceaeUCG008	rs75356640	G	A	0.089	0.137	0.030	9.83206E-06	-0.040	0.082	0.629	55.310
LachnospiraceaeUCG008	rs955844	A	C	0.200	0.112	0.023	1.80994E-06	-0.074	0.077	0.334	73.936
LachnospiraceaeUCG010	rs10414815	T	C	0.119	0.105	0.023	4.23977E-06	-0.173	0.127	0.175	42.201
LachnospiraceaeUCG010	rs11192447	A	G	0.075	0.127	0.024	4.68957E-07	-0.396	0.136	0.004	40.665
LachnospiraceaeUCG010	rs12346653	C	T	0.274	0.066	0.014	2.70321E-06	-0.054	0.066	0.409	31.641
LachnospiraceaeUCG010	rs17730011	G	A	0.219	-0.070	0.016	7.84597E-06	-0.178	0.067	0.008	30.967
LachnospiraceaeUCG010	rs2833528	C	T	0.361	-0.056	0.013	9.9199E-06	0.042	0.055	0.450	26.768
LachnospiraceaeUCG010	rs336138	G	T	0.163	0.078	0.017	7.48417E-06	-0.025	0.084	0.762	30.458
LachnospiraceaeUCG010	rs4576377	A	C	0.384	-0.057	0.013	7.63056E-06	0.026	0.056	0.645	28.421
LachnospiraceaeUCG010	rs72894957	G	A	0.048	0.222	0.049	5.68428E-06	-0.089	0.180	0.621	82.673
LachnospiraceaeUCG010	rs74315802	G	T	0.121	0.087	0.018	3.19006E-06	0.106	0.070	0.130	29.450
LachnospiraceaeUCG010	rs9981767	A	C	0.316	0.066	0.013	9.96373E-07	-0.085	0.061	0.168	34.086

Lactobacillus	rs12693845	C	T	0.357	-0.081	0.018	8.9632E-06	-0.014	0.055	0.800	54.774
Lactobacillus	rs1530559	G	A	0.568	0.080	0.018	4.93109E-06	-0.031	0.054	0.566	58.372
Lactobacillus	rs16861661	G	A	0.050	-0.183	0.038	1.2781E-06	0.036	0.109	0.739	58.288
Lactobacillus	rs62314653	C	A	0.063	0.188	0.039	2.24383E-06	-0.142	0.114	0.213	76.133
Lactobacillus	rs7399658	G	A	0.216	-0.107	0.022	3.12099E-06	-0.076	0.070	0.277	71.492
Lactobacillus	rs768253	T	G	0.391	-0.079	0.017	4.2477E-06	-0.027	0.054	0.612	54.923
Lactobacillus	rs77478751	A	G	0.054	-0.220	0.048	7.32705E-06	-0.027	0.084	0.745	90.556
Lactobacillus	rs921925	A	C	0.262	0.099	0.020	9.71665E-07	0.138	0.065	0.034	69.142
Lactococcus	rs10417872	T	G	0.258	0.118	0.025	1.28678E-06	0.066	0.059	0.262	98.899
Lactococcus	rs123059	T	C	0.222	-0.137	0.027	1.26562E-06	-0.047	0.065	0.471	119.043
Lactococcus	rs12621813	G	A	0.282	0.108	0.024	6.60829E-06	0.016	0.061	0.790	87.771
Lactococcus	rs17168302	G	A	0.066	0.192	0.042	6.28602E-06	0.001	0.088	0.992	83.126
Lactococcus	rs2293361	C	T	0.065	-0.199	0.043	1.39593E-06	0.062	0.119	0.606	88.383
Lactococcus	rs4766997	C	T	0.296	0.115	0.024	2.06145E-06	-0.003	0.054	0.954	100.963
Lactococcus	rs55910161	C	T	0.135	0.146	0.031	2.36214E-06	0.041	0.085	0.635	92.405
Lactococcus	rs6674304	C	T	0.069	0.201	0.044	6.17759E-06	-0.136	0.139	0.327	94.936
Marvinbryantia	rs11620597	T	C	0.053	0.119	0.027	7.80178E-06	0.034	0.170	0.843	26.175
Marvinbryantia	rs1187983	C	T	0.122	-0.094	0.019	2.02427E-06	0.075	0.088	0.390	34.516
Marvinbryantia	rs146541147	G	A	0.071	0.119	0.027	6.86288E-06	-0.110	0.150	0.461	34.053
Marvinbryantia	rs2724813	A	G	0.201	-0.084	0.017	6.28451E-07	-0.088	0.063	0.160	41.700
Marvinbryantia	rs2842896	C	T	0.528	-0.065	0.013	7.25363E-07	-0.048	0.055	0.378	38.629
Marvinbryantia	rs2863363	A	G	0.368	0.063	0.014	3.11281E-06	-0.114	0.062	0.065	34.437
Marvinbryantia	rs3125832	A	C	0.262	0.068	0.015	5.02731E-06	-0.009	0.064	0.888	32.817
Marvinbryantia	rs61884471	G	A	0.085	0.124	0.025	1.01163E-06	-0.007	0.086	0.934	44.031
Marvinbryantia	rs72948274	A	C	0.058	-0.126	0.027	3.2591E-06	0.223	0.110	0.042	31.892
Marvinbryantia	rs8006832	G	T	0.111	-0.095	0.022	6.58083E-06	-0.052	0.092	0.576	32.966

Methanobrevibacter	rs10202904	T	G	0.467	-0.113	0.024	3.08549E-06	-0.046	0.055	0.401	116.927
Methanobrevibacter	rs1334944	T	C	0.285	0.115	0.026	7.6138E-06	0.041	0.060	0.489	99.780
Methanobrevibacter	rs4802933	A	G	0.178	-0.136	0.031	9.73818E-06	0.053	0.064	0.405	99.204
Methanobrevibacter	rs6776814	T	C	0.090	-0.189	0.042	8.04905E-06	0.007	0.188	0.971	107.335
Methanobrevibacter	rs76029318	T	C	0.065	0.223	0.045	1.07552E-06	-0.160	0.109	0.144	110.725
Methanobrevibacter	rs894996	C	A	0.072	0.214	0.046	3.81567E-06	-0.096	0.105	0.360	112.558
Odoribacter	rs10093869	A	G	0.376	-0.058	0.013	3.67441E-06	0.000	0.054	0.998	28.764
Odoribacter	rs10423795	C	T	0.407	0.055	0.012	6.57936E-06	-0.050	0.055	0.368	26.875
Odoribacter	rs28417404	A	G	0.169	-0.073	0.016	3.68032E-06	-0.112	0.091	0.215	27.256
Odoribacter	rs4793970	A	G	0.301	-0.058	0.013	6.02696E-06	0.071	0.056	0.202	25.677
Odoribacter	rs6856150	G	A	0.118	0.088	0.019	6.0595E-06	-0.028	0.081	0.732	29.803
Odoribacter	rs74553962	T	G	0.063	0.121	0.026	9.48684E-06	0.089	0.102	0.385	31.799
Odoribacter	rs77779484	G	A	0.059	-0.133	0.027	6.56256E-07	-0.133	0.113	0.242	36.125
Olsenella	rs1035588	A	G	0.383	-0.108	0.024	4.85501E-06	-0.091	0.056	0.099	101.902
Olsenella	rs17148768	G	A	0.212	0.140	0.030	2.1984E-06	0.054	0.072	0.449	121.509
Olsenella	rs2759329	G	A	0.340	-0.111	0.024	3.43201E-06	0.050	0.056	0.367	102.210
Olsenella	rs35225860	A	G	0.057	-0.224	0.048	3.87041E-06	-0.012	0.136	0.933	98.606
Olsenella	rs61090148	A	G	0.423	-0.105	0.023	6.43728E-06	0.064	0.054	0.237	98.781
Olsenella	rs62112538	C	T	0.081	-0.199	0.041	1.1882E-06	-0.109	0.086	0.206	108.612
Olsenella	rs72691585	C	A	0.069	-0.249	0.052	2.95294E-06	-0.125	0.079	0.114	146.546
Olsenella	rs7540303	C	T	0.422	0.108	0.024	5.32027E-06	-0.011	0.056	0.849	104.993
Olsenella	rs8066522	G	A	0.347	-0.107	0.024	9.70095E-06	0.015	0.057	0.791	94.785
Olsenella	rs9460691	C	A	0.217	0.120	0.027	7.28354E-06	-0.046	0.068	0.504	90.035
Oscillibacter	rs11627628	T	C	0.073	0.144	0.029	1.0092E-06	-0.004	0.102	0.969	51.320
Oscillibacter	rs11990279	T	C	0.240	-0.082	0.018	4.94298E-06	-0.107	0.067	0.110	45.586
Oscillibacter	rs12649930	T	G	0.094	0.122	0.026	4.09379E-06	-0.081	0.089	0.365	46.470

Oscillibacter	rs133832	A	C	0.313	-0.080	0.016	1.14719E-06	0.006	0.060	0.925	50.056
Oscillibacter	rs16866406	A	G	0.139	0.099	0.021	3.07821E-06	0.015	0.075	0.839	43.065
Oscillibacter	rs16934185	A	G	0.104	-0.130	0.028	4.38031E-06	0.068	0.089	0.443	57.750
Oscillibacter	rs234108	A	G	0.480	0.075	0.015	9.16336E-07	-0.098	0.055	0.072	51.577
Oscillibacter	rs36095275	C	T	0.362	-0.075	0.016	1.39627E-06	0.008	0.055	0.881	48.062
Oscillibacter	rs4506202	A	G	0.516	-0.071	0.015	3.20825E-06	-0.031	0.054	0.564	46.464
Oscillibacter	rs61883564	A	G	0.124	-0.101	0.022	3.38712E-06	-0.080	0.078	0.302	41.100
Oscillibacter	rs75453768	G	T	0.087	0.122	0.027	5.34738E-06	-0.028	0.091	0.761	43.322
Oscillibacter	rs761240	T	G	0.067	-0.177	0.039	2.03841E-06	-0.134	0.126	0.289	71.415
Oscillibacter	rs9393920	A	G	0.435	-0.074	0.015	9.92237E-07	-0.031	0.055	0.576	50.132
Oscillospira	rs12206468	G	A	0.064	-0.133	0.027	1.04222E-06	0.015	0.100	0.881	38.728
Oscillospira	rs12925026	T	C	0.073	0.136	0.031	9.31021E-06	-0.122	0.116	0.292	45.511
Oscillospira	rs1954532	T	C	0.209	-0.083	0.018	2.26773E-06	-0.046	0.067	0.489	41.442
Oscillospira	rs28889936	A	C	0.108	0.114	0.025	3.36585E-06	0.097	0.091	0.287	46.187
Oscillospira	rs62422654	C	T	0.147	0.090	0.020	6.46754E-06	0.167	0.066	0.011	37.219
Oscillospira	rs72866977	A	C	0.070	-0.131	0.028	5.62701E-06	-0.107	0.100	0.286	40.579
Oscillospira	rs751183	T	C	0.246	-0.077	0.017	6.85127E-06	0.156	0.069	0.024	40.823
Oscillospira	rs8076323	A	G	0.258	0.072	0.016	5.61316E-06	-0.040	0.057	0.486	35.958
Oxalobacter	rs10464997	G	A	0.153	0.138	0.029	3.29754E-06	-0.027	0.069	0.691	90.603
Oxalobacter	rs11108500	A	G	0.077	-0.199	0.043	3.74279E-06	-0.018	0.096	0.849	103.290
Oxalobacter	rs111966731	T	C	0.072	0.213	0.047	7.29863E-06	-0.069	0.096	0.468	111.400
Oxalobacter	rs12002250	A	C	0.060	0.217	0.047	1.41504E-06	0.328	0.135	0.015	97.421
Oxalobacter	rs1569853	T	C	0.138	-0.138	0.030	3.64501E-06	0.015	0.082	0.860	83.660
Oxalobacter	rs36057338	G	T	0.076	0.208	0.042	8.79803E-07	-0.096	0.148	0.517	111.263
Oxalobacter	rs3862635	C	T	0.079	-0.172	0.039	9.18685E-06	-0.068	0.094	0.465	78.956
Oxalobacter	rs4428215	G	A	0.260	0.130	0.024	7.51071E-08	0.098	0.062	0.110	120.700

Oxalobacter	rs6000536	C	T	0.211	-0.131	0.025	2.06054E-07	0.175	0.068	0.010	105.260
Oxalobacter	rs6993398	G	A	0.153	0.127	0.028	7.12773E-06	-0.024	0.069	0.727	77.287
Oxalobacter	rs736744	C	T	0.416	0.118	0.021	2.57471E-08	0.005	0.054	0.930	124.616
Parabacteroides	rs115602804	G	A	0.067	0.103	0.022	1.93059E-06	-0.042	0.088	0.632	24.258
Parabacteroides	rs4236095	G	A	0.125	0.076	0.016	1.93145E-06	-0.164	0.088	0.062	23.351
Parabacteroides	rs60884758	C	T	0.165	-0.070	0.014	5.70689E-07	0.082	0.070	0.243	24.983
Parabacteroides	rs6657302	T	C	0.052	-0.105	0.023	9.75699E-06	-0.240	0.111	0.031	19.664
Parabacteroides	rs7298818	C	T	0.077	0.089	0.020	8.53629E-06	-0.096	0.089	0.280	20.493
Paraprevotella	rs10842464	T	C	0.419	-0.076	0.017	6.59715E-06	-0.053	0.058	0.366	51.455
Paraprevotella	rs140997932	T	C	0.065	-0.162	0.035	2.11119E-06	-0.010	0.117	0.935	58.619
Paraprevotella	rs145020347	A	G	0.139	-0.125	0.026	4.03009E-06	0.046	0.076	0.544	68.538
Paraprevotella	rs17109926	A	G	0.188	-0.099	0.022	6.75279E-06	0.008	0.060	0.895	54.831
Paraprevotella	rs17785622	A	G	0.060	0.248	0.052	1.92659E-06	0.121	0.139	0.387	127.374
Paraprevotella	rs2081023	A	G	0.102	-0.123	0.024	2.638E-07	0.128	0.077	0.097	50.777
Paraprevotella	rs3008582	T	C	0.132	0.106	0.023	4.35989E-06	-0.040	0.069	0.559	47.148
Paraprevotella	rs3801748	G	A	0.346	0.078	0.017	5.20177E-06	0.039	0.056	0.485	50.588
Paraprevotella	rs4756632	G	T	0.093	-0.139	0.029	3.81747E-06	-0.065	0.080	0.417	60.118
Paraprevotella	rs4767113	C	T	0.287	0.088	0.018	2.14357E-06	0.018	0.057	0.749	58.669
Paraprevotella	rs7240324	T	G	0.191	-0.102	0.023	5.95902E-06	-0.138	0.062	0.026	59.466
Paraprevotella	rs9602779	A	C	0.173	-0.107	0.022	6.92746E-07	0.101	0.063	0.107	59.926
Paraprevotella	rs9900242	A	G	0.361	-0.085	0.018	1.14068E-06	0.015	0.056	0.795	61.746
Parasutterella	rs10899911	A	G	0.272	-0.072	0.015	1.15272E-06	0.010	0.063	0.878	37.457
Parasutterella	rs11715853	G	A	0.314	-0.066	0.015	6.22913E-06	-0.015	0.059	0.800	34.793
Parasutterella	rs2090816	A	C	0.157	0.084	0.018	2.89715E-06	-0.039	0.069	0.576	34.412
Parasutterella	rs35055552	T	C	0.078	0.110	0.024	3.34853E-06	0.041	0.078	0.603	31.525
Parasutterella	rs55877868	A	C	0.096	-0.104	0.023	2.87068E-06	0.047	0.089	0.598	34.926

Parasutterella	rs62273907	A	G	0.050	0.229	0.050	5.88384E-06	-0.259	0.107	0.016	91.666
Parasutterella	rs6809952	G	A	0.273	-0.068	0.015	8.13349E-06	-0.020	0.061	0.745	34.246
Parasutterella	rs6828768	C	T	0.500	0.064	0.013	1.78328E-06	0.021	0.054	0.692	37.263
Parasutterella	rs7303158	C	T	0.442	0.065	0.013	1.32776E-06	0.060	0.054	0.268	37.933
Parasutterella	rs7311004	T	C	0.376	-0.062	0.014	5.92054E-06	-0.032	0.054	0.553	32.862
Parasutterella	rs7572229	G	A	0.490	0.066	0.013	6.31698E-07	-0.067	0.054	0.211	40.344
Parasutterella	rs78383039	T	C	0.067	-0.146	0.030	1.57438E-06	-0.250	0.136	0.067	48.939
Parasutterella	rs8039785	T	G	0.452	0.062	0.013	3.62095E-06	0.015	0.054	0.774	34.805
Parasutterella	rs823424	G	A	0.210	-0.071	0.016	4.95295E-06	0.116	0.062	0.060	30.991
Peptococcus	rs10031059	T	C	0.221	-0.121	0.023	1.23637E-07	0.003	0.063	0.966	93.079
Peptococcus	rs11001941	G	A	0.068	-0.196	0.039	1.32858E-06	0.162	0.096	0.092	88.883
Peptococcus	rs12069354	C	T	0.067	0.168	0.038	9.28339E-06	-0.036	0.109	0.743	64.290
Peptococcus	rs2054133	G	A	0.474	0.090	0.019	2.14166E-06	-0.035	0.056	0.532	73.616
Peptococcus	rs36121075	A	G	0.128	-0.141	0.031	6.9872E-06	-0.161	0.072	0.025	81.473
Peptococcus	rs413827	G	A	0.213	0.110	0.024	3.30085E-06	0.054	0.063	0.391	74.930
Peptococcus	rs5770862	T	C	0.067	0.162	0.036	3.21957E-06	-0.024	0.093	0.793	60.044
Peptococcus	rs6918730	G	A	0.097	0.135	0.029	1.14732E-06	0.026	0.112	0.813	59.224
Peptococcus	rs7033353	T	G	0.420	0.090	0.019	2.21957E-06	0.065	0.054	0.224	72.877
Peptococcus	rs72850165	T	C	0.108	-0.134	0.030	5.74152E-06	0.015	0.100	0.879	64.110
Peptococcus	rs74592222	G	A	0.117	0.138	0.030	8.55342E-06	-0.063	0.084	0.450	72.560
Peptococcus	rs77681628	C	T	0.059	0.200	0.039	2.68836E-07	0.080	0.098	0.417	81.541
Phascolarctobacterium	rs12618201	A	G	0.347	0.064	0.014	3.37696E-06	0.027	0.054	0.615	34.275
Phascolarctobacterium	rs1264476	T	G	0.200	0.077	0.017	4.29567E-06	-0.032	0.069	0.640	34.591
Phascolarctobacterium	rs28525131	G	A	0.094	-0.119	0.027	8.22677E-06	0.070	0.125	0.575	44.251

Phascolarctobacterium	rs56069061	G	A	0.101	-0.111	0.023	1.87174E-06	-0.043	0.107	0.687	41.496
Phascolarctobacterium	rs56157888	A	C	0.170	0.095	0.019	1.08506E-06	0.032	0.065	0.625	47.305
Phascolarctobacterium	rs74540770	G	A	0.065	-0.121	0.026	3.60275E-06	0.065	0.100	0.517	32.513
Phascolarctobacterium	rs75882962	T	C	0.167	0.097	0.019	3.18987E-07	0.011	0.081	0.891	47.998
Phascolarctobacterium	rs7982713	G	A	0.229	0.073	0.016	9.72164E-06	-0.063	0.060	0.291	34.239
Prevotella7	rs118038478	A	G	0.076	0.206	0.047	7.85422E-06	0.001	0.105	0.992	108.937
Prevotella7	rs12124567	A	G	0.262	-0.121	0.028	9.48982E-06	0.013	0.067	0.851	105.011
Prevotella7	rs12195431	T	C	0.079	0.197	0.044	8.72818E-06	0.161	0.091	0.077	103.045
Prevotella7	rs2240542	C	T	0.258	0.121	0.026	4.84146E-06	0.009	0.061	0.879	102.987
Prevotella7	rs2918132	C	T	0.368	-0.115	0.025	6.42409E-06	-0.086	0.055	0.119	112.784
Prevotella7	rs430270	A	C	0.201	0.139	0.030	2.86846E-06	-0.078	0.068	0.257	114.685
Prevotella7	rs57404562	C	A	0.180	0.155	0.032	6.21576E-07	0.011	0.079	0.891	131.757
Prevotella7	rs79263163	A	C	0.185	-0.144	0.032	7.51048E-06	-0.063	0.066	0.347	115.310
Prevotella7	rs9426434	T	C	0.273	-0.124	0.028	9.71711E-06	-0.083	0.056	0.142	112.089
Prevotella7	rs9608249	A	G	0.171	-0.158	0.034	2.06687E-06	0.012	0.084	0.882	131.073
Prevotella7	rs9959718	G	A	0.250	0.133	0.028	1.90058E-06	-0.185	0.068	0.006	122.313
Prevotella9	rs111509883	T	C	0.085	0.171	0.035	1.24389E-06	0.000	0.087	0.998	83.469
Prevotella9	rs11685699	C	T	0.083	-0.141	0.030	2.02552E-06	0.145	0.102	0.153	55.641
Prevotella9	rs117271932	A	G	0.047	0.208	0.044	2.81722E-06	-0.048	0.117	0.679	70.975
Prevotella9	rs12648235	T	C	0.157	0.079	0.018	7.39079E-06	0.056	0.064	0.380	30.046
Prevotella9	rs1304512	G	A	0.219	0.076	0.017	5.29082E-06	-0.072	0.060	0.232	36.421
Prevotella9	rs2104588	T	C	0.119	0.106	0.024	8.12987E-06	0.258	0.115	0.025	43.038
Prevotella9	rs2495052	A	G	0.179	0.084	0.019	8.97142E-06	-0.014	0.076	0.856	38.000
Prevotella9	rs2683313	A	G	0.308	-0.072	0.015	1.68895E-06	-0.012	0.058	0.832	41.147

Prevotella9	rs4968431	G	T	0.364	0.064	0.014	8.58441E-06	-0.041	0.056	0.465	34.848
Prevotella9	rs7237249	C	T	0.135	-0.082	0.018	8.93303E-06	0.045	0.068	0.506	29.145
Prevotella9	rs72815774	T	C	0.071	-0.176	0.039	8.78291E-06	-0.053	0.114	0.643	75.007
Prevotella9	rs746764	T	C	0.173	-0.092	0.019	2.03999E-06	-0.045	0.065	0.495	44.111
Prevotella9	rs7976209	T	C	0.167	-0.087	0.020	7.28343E-06	-0.028	0.074	0.703	38.769
Prevotella9	rs9428102	A	G	0.237	-0.078	0.018	4.61734E-06	-0.006	0.065	0.928	40.272
Prevotella9	rs9613013	G	A	0.145	0.092	0.020	6.10415E-06	-0.062	0.082	0.448	38.386
RikenellaceaeRC9gutgroup	rs12501673	A	G	0.325	0.116	0.026	6.28983E-06	-0.005	0.061	0.940	109.655
RikenellaceaeRC9gutgroup	rs17032291	T	C	0.141	-0.170	0.037	6.61004E-06	-0.112	0.081	0.167	128.792
RikenellaceaeRC9gutgroup	rs17582787	A	G	0.129	-0.158	0.034	3.55377E-06	-0.018	0.072	0.800	103.248
RikenellaceaeRC9gutgroup	rs2074881	T	C	0.179	-0.142	0.032	9.44772E-06	0.072	0.080	0.367	109.647
RikenellaceaeRC9gutgroup	rs2900503	G	T	0.171	-0.172	0.033	1.55282E-07	0.026	0.073	0.716	155.712
RikenellaceaeRC9gutgroup	rs2998141	T	C	0.222	-0.136	0.029	4.42373E-06	-0.039	0.063	0.540	118.407
RikenellaceaeRC9gutgroup	rs4270579	G	A	0.304	-0.118	0.027	5.45647E-06	-0.009	0.057	0.871	108.771
RikenellaceaeRC9gutgroup	rs4717843	G	T	0.362	-0.119	0.026	4.7173E-06	0.032	0.054	0.547	121.481
RikenellaceaeRC9gutgroup	rs7712231	A	G	0.165	0.156	0.035	7.97036E-06	0.097	0.079	0.221	124.255
RikenellaceaeRC9gutg	rs80309088	G	A	0.129	0.174	0.038	4.55642E-06	-0.136	0.082	0.098	125.858

roup											
RikenellaceaeRC9gutgroup	rs9887954	G	A	0.436	-0.115	0.025	4.80801E-06	-0.033	0.055	0.549	119.763
Romboutsia	rs10279978	A	G	0.348	-0.062	0.013	1.17009E-06	-0.090	0.058	0.118	32.268
Romboutsia	rs11221428	T	C	0.155	-0.073	0.016	6.48646E-06	-0.027	0.064	0.667	25.422
Romboutsia	rs16843578	C	T	0.083	-0.088	0.020	5.08123E-06	-0.030	0.119	0.803	21.293
Romboutsia	rs28603357	T	C	0.064	-0.215	0.047	8.52235E-06	-0.093	0.194	0.632	101.441
Romboutsia	rs34302036	A	G	0.415	0.055	0.012	5.87667E-06	0.016	0.054	0.763	26.990
Romboutsia	rs61841503	G	A	0.137	0.093	0.017	3.99597E-08	0.037	0.080	0.643	37.536
Romboutsia	rs62504452	A	G	0.166	-0.071	0.016	4.65831E-06	0.023	0.077	0.763	25.635
Romboutsia	rs7109293	A	G	0.092	0.092	0.021	6.98354E-06	-0.065	0.084	0.440	26.020
Romboutsia	rs75200530	T	G	0.047	-0.191	0.042	5.06555E-06	0.237	0.157	0.130	59.537
Romboutsia	rs75987356	G	A	0.069	-0.130	0.028	6.70799E-06	0.008	0.100	0.939	39.431
Romboutsia	rs77702691	A	G	0.102	-0.094	0.021	7.36973E-06	0.022	0.094	0.819	30.094
Romboutsia	rs9389266	T	G	0.169	0.072	0.016	9.38461E-06	-0.067	0.070	0.344	26.950
Romboutsia	rs9567264	C	T	0.320	0.058	0.013	5.75689E-06	-0.031	0.057	0.589	26.893
Roseburia	rs12740451	T	C	0.125	0.070	0.015	7.34264E-06	0.069	0.078	0.382	19.565
Roseburia	rs147990086	A	G	0.191	-0.058	0.013	8.92942E-06	0.043	0.070	0.539	19.006
Roseburia	rs16910295	T	C	0.061	-0.098	0.021	2.91111E-06	-0.115	0.119	0.333	20.092
Roseburia	rs2160994	T	C	0.380	0.055	0.011	9.6985E-07	-0.089	0.057	0.118	26.233
Roseburia	rs2943022	T	C	0.460	0.049	0.011	4.11408E-06	-0.103	0.054	0.057	22.242
Roseburia	rs302266	T	C	0.120	-0.078	0.017	8.12587E-06	0.044	0.081	0.581	23.482
Roseburia	rs329182	T	C	0.157	0.069	0.015	5.90142E-06	-0.048	0.073	0.511	23.174
Roseburia	rs55858165	A	C	0.078	0.179	0.040	9.98975E-06	-0.076	0.141	0.589	84.671
Roseburia	rs57466170	C	T	0.087	0.074	0.017	8.30102E-06	0.090	0.102	0.376	15.944
Roseburia	rs6445851	G	A	0.401	-0.050	0.011	3.52721E-06	-0.109	0.055	0.048	21.808

Roseburia	rs6930661	C	T	0.076	-0.096	0.020	2.47942E-06	0.035	0.114	0.758	23.701
Roseburia	rs75326254	C	T	0.064	-0.105	0.023	7.50204E-06	-0.025	0.113	0.821	23.942
Roseburia	rs78753150	A	C	0.059	0.097	0.021	9.98312E-06	0.076	0.090	0.397	19.007
Roseburia	rs9300744	C	T	0.239	-0.059	0.013	4.75118E-06	-0.017	0.070	0.803	23.101
Ruminiclostridium5	rs10827477	A	G	0.315	-0.055	0.012	2.19177E-06	0.046	0.056	0.416	23.747
Ruminiclostridium5	rs113753996	T	C	0.137	0.082	0.017	3.98895E-06	-0.093	0.069	0.181	29.288
Ruminiclostridium5	rs1223978	T	C	0.480	0.048	0.011	8.16186E-06	0.103	0.054	0.056	21.485
Ruminiclostridium5	rs1492620	T	C	0.081	-0.083	0.018	3.52577E-06	0.058	0.081	0.475	18.744
Ruminiclostridium5	rs2482038	C	A	0.438	0.052	0.011	1.70314E-06	-0.065	0.055	0.231	24.354
Ruminiclostridium5	rs2791343	T	C	0.340	0.052	0.011	5.53959E-06	0.036	0.055	0.508	22.039
Ruminiclostridium5	rs2833828	G	A	0.453	0.049	0.011	6.81651E-06	0.067	0.054	0.217	21.815
Ruminiclostridium5	rs4955951	A	G	0.092	-0.071	0.017	9.95519E-06	-0.068	0.083	0.411	15.682
Ruminiclostridium5	rs6121460	G	A	0.081	0.093	0.020	2.64107E-06	-0.134	0.100	0.177	23.660
Ruminiclostridium5	rs79968837	A	G	0.069	-0.095	0.019	1.14623E-06	-0.071	0.121	0.556	21.187
Ruminiclostridium5	rs8053158	A	G	0.120	-0.074	0.016	5.89806E-06	-0.067	0.082	0.416	21.310
Ruminiclostridium6	rs10829821	T	C	0.086	-0.098	0.022	3.47141E-06	0.044	0.093	0.639	27.361
Ruminiclostridium6	rs116969552	A	G	0.036	-0.167	0.038	9.15949E-06	0.080	0.160	0.616	35.300
Ruminiclostridium6	rs11992182	A	C	0.288	0.063	0.014	4.64839E-06	-0.077	0.064	0.232	29.471
Ruminiclostridium6	rs2548459	C	T	0.470	0.055	0.012	6.40206E-06	0.140	0.054	0.010	28.172
Ruminiclostridium6	rs35362464	C	A	0.151	0.072	0.017	8.99138E-06	-0.084	0.078	0.281	24.425
Ruminiclostridium6	rs61060922	T	G	0.045	0.159	0.032	1.08647E-06	0.036	0.150	0.810	39.744
Ruminiclostridium6	rs663262	T	C	0.052	-0.135	0.031	3.39417E-06	0.001	0.146	0.995	32.820
Ruminiclostridium6	rs67479537	T	C	0.080	0.119	0.026	9.302E-06	0.058	0.127	0.650	38.082
Ruminiclostridium6	rs71414120	T	G	0.077	0.201	0.041	1.07697E-06	-0.022	0.122	0.857	105.172
Ruminiclostridium6	rs72991535	T	G	0.060	0.136	0.030	4.95367E-06	0.140	0.146	0.337	37.877
Ruminiclostridium6	rs73176030	T	C	0.318	0.059	0.013	7.28883E-06	0.080	0.060	0.183	27.420

Ruminiclostridium6	rs77193512	A	G	0.167	0.074	0.015	1.29587E-06	-0.025	0.062	0.681	27.719
Ruminiclostridium6	rs792058	G	A	0.427	0.055	0.013	8.57953E-06	-0.054	0.054	0.320	27.613
Ruminiclostridium6	rs79968172	G	A	0.056	0.116	0.024	1.66054E-06	0.183	0.114	0.107	26.057
Ruminiclostridium6	rs9555756	A	C	0.124	-0.080	0.018	7.10127E-06	0.061	0.096	0.524	25.851
Ruminiclostridium9	rs12040548	G	T	0.311	0.057	0.012	3.14872E-06	0.021	0.060	0.730	25.612
Ruminiclostridium9	rs6082461	A	C	0.207	0.059	0.013	4.86594E-06	-0.026	0.066	0.699	20.712
Ruminiclostridium9	rs7137760	C	T	0.415	0.051	0.011	7.06911E-06	-0.058	0.054	0.284	22.996
Ruminiclostridium9	rs74303178	T	C	0.320	0.053	0.012	7.91566E-06	-0.080	0.058	0.164	22.682
Ruminiclostridium9	rs78191726	T	C	0.091	0.094	0.021	7.58287E-06	-0.017	0.104	0.869	26.998
Ruminiclostridium9	rs918449	A	G	0.070	-0.095	0.020	2.55994E-06	-0.213	0.104	0.041	21.500
Ruminiclostridium9	rs9522712	T	C	0.145	0.070	0.015	4.65962E-06	0.011	0.076	0.882	22.302
Ruminiclostridium9	rs9809789	C	T	0.168	-0.072	0.016	8.71968E-06	0.164	0.070	0.019	26.426
RuminococcaceaeNK4 A214group	rs11241747	C	T	0.391	0.053	0.012	6.58971E-06	0.017	0.059	0.774	24.917
RuminococcaceaeNK4 A214group	rs11586410	G	A	0.134	-0.086	0.017	3.66276E-07	-0.050	0.074	0.496	31.824
RuminococcaceaeNK4 A214group	rs12642039	T	C	0.366	-0.055	0.012	3.43005E-06	-0.054	0.056	0.328	26.057
RuminococcaceaeNK4 A214group	rs12731	A	G	0.451	-0.053	0.012	4.87237E-06	0.047	0.055	0.397	25.324
RuminococcaceaeNK4 A214group	rs13087692	T	G	0.305	0.057	0.013	8.69224E-06	0.052	0.059	0.376	25.716
RuminococcaceaeNK4 A214group	rs136761	G	A	0.429	-0.059	0.012	8.15195E-07	-0.029	0.056	0.604	31.071
RuminococcaceaeNK4 A214group	rs147475196	A	G	0.078	-0.134	0.030	4.72496E-06	0.082	0.088	0.350	47.040

RuminococcaceaeNK4A214group	rs35559912	T	C	0.066	-0.093	0.020	4.89023E-06	0.012	0.083	0.884	19.259
RuminococcaceaeNK4A214group	rs4814689	C	T	0.067	-0.108	0.023	4.5519E-06	0.282	0.132	0.032	26.780
RuminococcaceaeNK4A214group	rs5994253	A	G	0.146	-0.081	0.016	2.35183E-07	-0.109	0.077	0.154	30.166
RuminococcaceaeNK4A214group	rs62027366	T	C	0.230	0.062	0.014	6.58456E-06	0.044	0.067	0.508	24.601
RuminococcaceaeNK4A214group	rs6681678	C	T	0.050	-0.100	0.024	9.04931E-06	0.073	0.148	0.623	17.399
RuminococcaceaeNK4A214group	rs7573569	T	C	0.072	0.108	0.023	3.23139E-06	-0.084	0.112	0.451	28.343
RuminococcaceaeUCG002	rs10916131	C	T	0.138	-0.069	0.015	2.87497E-06	-0.029	0.073	0.692	21.022
RuminococcaceaeUCG002	rs10927423	C	A	0.183	-0.071	0.015	8.49518E-07	0.041	0.070	0.561	27.955
RuminococcaceaeUCG002	rs10964441	G	A	0.053	-0.149	0.034	7.45116E-06	-0.035	0.088	0.691	40.773
RuminococcaceaeUCG002	rs113147300	A	G	0.132	-0.076	0.016	7.6914E-06	0.019	0.078	0.810	24.234
RuminococcaceaeUCG002	rs11607472	A	G	0.107	-0.078	0.018	7.18671E-06	-0.061	0.107	0.569	21.429
RuminococcaceaeUCG002	rs116974815	C	A	0.058	-0.190	0.040	2.02518E-06	-0.050	0.106	0.633	72.065
RuminococcaceaeUCG002	rs11750293	G	T	0.281	-0.058	0.012	1.76136E-06	-0.035	0.056	0.529	24.831

RuminococcaceaeUCG002	rs12463378	A	G	0.418	-0.052	0.011	2.95523E-06	-0.010	0.058	0.860	24.342
RuminococcaceaeUCG002	rs15256	C	T	0.087	0.073	0.017	9.45659E-06	-0.069	0.083	0.407	15.558
RuminococcaceaeUCG002	rs55793120	T	C	0.051	0.137	0.027	4.80674E-07	-0.134	0.114	0.238	33.383
RuminococcaceaeUCG002	rs57079348	T	G	0.121	-0.077	0.017	7.21822E-06	0.110	0.115	0.338	22.950
RuminococcaceaeUCG002	rs6542556	A	G	0.345	0.051	0.011	7.86123E-06	0.008	0.055	0.880	21.557
RuminococcaceaeUCG002	rs6793778	C	T	0.262	-0.056	0.013	9.81289E-06	-0.102	0.061	0.095	22.185
RuminococcaceaeUCG002	rs7120052	A	C	0.176	0.062	0.014	1.96895E-06	-0.004	0.068	0.951	20.778
RuminococcaceaeUCG002	rs7155595	C	A	0.309	0.057	0.012	1.14931E-06	0.083	0.058	0.153	25.477
RuminococcaceaeUCG002	rs7249614	A	G	0.385	-0.049	0.011	9.07376E-06	-0.099	0.055	0.073	21.105
RuminococcaceaeUCG002	rs76847269	A	G	0.057	0.164	0.036	5.1719E-06	-0.064	0.170	0.708	52.594
RuminococcaceaeUCG002	rs77564310	A	C	0.179	-0.071	0.014	3.28614E-07	0.097	0.066	0.143	27.437
RuminococcaceaeUCG002	rs79016051	C	T	0.091	-0.089	0.019	2.33742E-06	0.019	0.079	0.809	23.822
RuminococcaceaeUCG002	rs882348	A	G	0.106	-0.080	0.018	5.44852E-06	0.059	0.083	0.475	22.342

RuminococcaceaeUCG003	rs10490280	C	T	0.218	-0.067	0.014	4.15551E-06	0.076	0.068	0.269	28.258
RuminococcaceaeUCG003	rs11243416	T	C	0.101	-0.093	0.019	1.66882E-06	0.130	0.106	0.218	28.679
RuminococcaceaeUCG003	rs11613919	G	T	0.213	0.073	0.016	1.62509E-06	0.114	0.065	0.079	32.572
RuminococcaceaeUCG003	rs16959793	A	C	0.280	-0.063	0.013	2.21709E-06	0.047	0.054	0.385	28.972
RuminococcaceaeUCG003	rs2523124	T	C	0.398	-0.055	0.012	5.7837E-06	-0.098	0.055	0.073	26.285
RuminococcaceaeUCG003	rs3013089	G	A	0.385	-0.055	0.012	4.38133E-06	0.034	0.055	0.545	26.440
RuminococcaceaeUCG003	rs4452755	A	C	0.305	-0.063	0.013	3.29335E-06	-0.033	0.057	0.559	31.360
RuminococcaceaeUCG003	rs4532474	G	A	0.149	0.077	0.017	4.82127E-06	-0.031	0.072	0.666	27.574
RuminococcaceaeUCG003	rs646327	G	A	0.468	0.059	0.012	7.8318E-07	0.132	0.054	0.015	31.487
RuminococcaceaeUCG003	rs6759615	A	G	0.070	0.103	0.020	7.86224E-07	0.193	0.090	0.031	24.997
RuminococcaceaeUCG003	rs73341549	T	C	0.051	-0.170	0.032	1.50953E-07	-0.132	0.116	0.253	51.070
RuminococcaceaeUCG003	rs78720113	A	G	0.070	-0.115	0.025	7.59261E-06	-0.104	0.100	0.298	31.654
RuminococcaceaeUCG004	rs10976229	T	G	0.141	0.096	0.021	7.04477E-06	-0.063	0.080	0.427	41.059

RuminococcaceaeUCG004	rs11961899	G	A	0.271	-0.071	0.016	9.1802E-06	0.062	0.060	0.300	36.408
RuminococcaceaeUCG004	rs12125734	G	T	0.087	0.134	0.026	2.09428E-07	0.028	0.093	0.760	52.163
RuminococcaceaeUCG004	rs2248146	T	C	0.322	0.069	0.015	8.20001E-06	-0.038	0.056	0.495	38.146
RuminococcaceaeUCG004	rs3800154	A	C	0.221	-0.080	0.018	6.11581E-06	-0.026	0.061	0.667	40.253
RuminococcaceaeUCG004	rs511258	G	A	0.263	-0.076	0.016	4.52052E-06	0.032	0.069	0.646	40.920
RuminococcaceaeUCG004	rs550351	A	C	0.440	0.079	0.018	9.43128E-06	-0.025	0.054	0.648	55.993
RuminococcaceaeUCG004	rs6769553	A	G	0.284	0.085	0.016	7.91068E-08	0.078	0.061	0.200	54.043
RuminococcaceaeUCG004	rs7569771	A	G	0.217	-0.076	0.017	8.11964E-06	-0.111	0.062	0.075	35.947
RuminococcaceaeUCG004	rs872501	G	A	0.127	0.116	0.026	5.80805E-06	0.178	0.100	0.074	55.073
RuminococcaceaeUCG004	rs9818949	G	T	0.219	0.086	0.019	5.38832E-06	-0.025	0.068	0.717	46.459
RuminococcaceaeUCG005	rs10873449	T	C	0.169	0.065	0.014	4.11169E-06	0.188	0.064	0.003	22.114
RuminococcaceaeUCG005	rs10937802	G	A	0.097	0.076	0.017	8.17353E-06	0.144	0.082	0.079	18.437
RuminococcaceaeUCG005	rs10950694	T	C	0.424	0.058	0.011	4.298E-07	0.035	0.056	0.525	29.925

RuminococcaceaeUCG005	rs114279581	A	G	0.048	-0.147	0.032	3.21853E-06	0.015	0.101	0.879	35.877
RuminococcaceaeUCG005	rs12288512	A	G	0.180	0.067	0.014	3.10278E-06	-0.093	0.063	0.139	24.058
RuminococcaceaeUCG005	rs12458218	T	C	0.177	0.068	0.014	2.41027E-06	0.082	0.070	0.240	24.532
RuminococcaceaeUCG005	rs2893871	G	A	0.152	-0.074	0.016	3.53845E-06	0.148	0.086	0.083	25.688
RuminococcaceaeUCG005	rs34781347	G	A	0.060	0.189	0.039	6.04728E-07	-0.028	0.105	0.792	73.477
RuminococcaceaeUCG005	rs55793120	T	C	0.051	0.122	0.028	7.37046E-06	-0.134	0.114	0.238	26.116
RuminococcaceaeUCG005	rs72776570	C	A	0.083	0.087	0.020	5.35716E-06	0.061	0.091	0.504	21.069
RuminococcaceaeUCG005	rs7449320	C	A	0.221	0.060	0.013	4.80776E-06	-0.130	0.063	0.040	22.673
RuminococcaceaeUCG005	rs7555878	A	G	0.280	0.059	0.013	2.81022E-06	-0.067	0.062	0.276	25.501
RuminococcaceaeUCG005	rs7586445	G	A	0.126	0.078	0.018	8.8145E-06	0.154	0.079	0.053	24.788
RuminococcaceaeUCG005	rs898577	T	C	0.055	-0.123	0.029	7.46313E-06	0.133	0.116	0.249	28.742
RuminococcaceaeUCG009	rs12508214	C	T	0.351	-0.077	0.017	4.75487E-06	0.003	0.057	0.963	50.266
RuminococcaceaeUCG009	rs138460696	A	G	0.106	0.139	0.032	9.80841E-06	0.023	0.103	0.821	67.891

RuminococcaceaeUCG009	rs1550196	G	A	0.117	0.131	0.026	1.13288E-06	-0.036	0.090	0.689	65.242
RuminococcaceaeUCG009	rs2058609	A	G	0.314	0.082	0.017	3.12485E-06	0.068	0.060	0.259	52.824
RuminococcaceaeUCG009	rs2192926	A	G	0.237	-0.089	0.019	4.87746E-06	-0.047	0.057	0.410	52.680
RuminococcaceaeUCG009	rs4079028	C	T	0.194	0.092	0.020	3.28244E-06	0.059	0.063	0.348	48.167
RuminococcaceaeUCG009	rs4708333	T	G	0.302	-0.084	0.017	1.56246E-06	-0.078	0.057	0.166	54.777
RuminococcaceaeUCG009	rs6952765	G	A	0.461	0.073	0.017	8.13062E-06	-0.082	0.058	0.153	48.977
RuminococcaceaeUCG009	rs758191	T	G	0.064	0.177	0.038	9.00992E-06	0.186	0.091	0.041	68.702
RuminococcaceaeUCG009	rs78410648	A	G	0.097	0.121	0.028	9.67203E-06	-0.024	0.086	0.778	47.320
RuminococcaceaeUCG009	rs9558661	T	C	0.187	-0.090	0.020	7.00847E-06	0.089	0.067	0.183	45.038
RuminococcaceaeUCG010	rs12597105	G	A	0.277	0.067	0.014	4.873E-06	0.030	0.068	0.664	33.138
RuminococcaceaeUCG010	rs2820282	A	C	0.452	-0.059	0.013	2.85385E-06	-0.027	0.055	0.619	31.930
RuminococcaceaeUCG010	rs682403	A	G	0.476	-0.059	0.012	2.36537E-06	-0.028	0.054	0.602	31.701
RuminococcaceaeUCG010	rs6958419	C	T	0.489	-0.059	0.012	2.84162E-06	-0.048	0.054	0.374	31.495

RuminococcaceaeUCG010	rs73218807	G	A	0.058	-0.166	0.037	6.43033E-06	-0.038	0.097	0.698	55.255
RuminococcaceaeUCG010	rs7441445	C	T	0.435	-0.057	0.013	6.80187E-06	-0.002	0.054	0.972	29.288
RuminococcaceaeUCG011	rs10274562	C	T	0.352	0.111	0.024	6.49607E-06	-0.029	0.055	0.593	103.487
RuminococcaceaeUCG011	rs12636310	G	A	0.218	0.133	0.028	2.81235E-06	-0.075	0.062	0.227	110.697
RuminococcaceaeUCG011	rs12724320	C	T	0.275	-0.121	0.025	1.51986E-06	0.072	0.055	0.194	107.547
RuminococcaceaeUCG011	rs1416041	A	C	0.134	-0.182	0.034	7.04419E-08	0.111	0.066	0.095	142.784
RuminococcaceaeUCG011	rs2729556	C	T	0.499	-0.109	0.023	3.18679E-06	0.014	0.054	0.795	109.784
RuminococcaceaeUCG011	rs4490371	T	C	0.331	-0.112	0.025	7.74624E-06	-0.002	0.055	0.965	102.108
RuminococcaceaeUCG011	rs79113084	C	T	0.155	-0.152	0.032	2.06123E-06	-0.110	0.087	0.203	111.963
RuminococcaceaeUCG011	rs9729514	A	G	0.102	0.185	0.039	2.36769E-06	-0.012	0.092	0.893	116.021
RuminococcaceaeUCG013	rs11581881	C	T	0.181	0.066	0.014	4.72582E-06	-0.012	0.064	0.851	23.790
RuminococcaceaeUCG013	rs12189346	G	A	0.163	0.068	0.015	1.67955E-06	-0.067	0.069	0.328	23.506
RuminococcaceaeUCG013	rs12336782	T	C	0.082	-0.086	0.019	8.60305E-06	0.057	0.100	0.565	20.141

RuminococcaceaeUCG013	rs12485353	G	A	0.179	-0.061	0.013	4.19379E-06	-0.090	0.062	0.146	19.931
RuminococcaceaeUCG013	rs12781711	C	T	0.313	-0.066	0.012	2.55301E-08	0.012	0.062	0.841	34.021
RuminococcaceaeUCG013	rs16918863	A	C	0.061	0.111	0.024	4.15503E-06	-0.033	0.110	0.765	25.990
RuminococcaceaeUCG013	rs2730183	G	A	0.464	-0.049	0.011	8.43787E-06	-0.187	0.055	0.001	21.811
RuminococcaceaeUCG013	rs4385846	G	T	0.207	0.060	0.013	6.46209E-06	0.018	0.068	0.796	21.568
RuminococcaceaeUCG013	rs75088940	T	C	0.093	-0.094	0.020	2.55399E-06	-0.172	0.105	0.100	27.656
RuminococcaceaeUCG013	rs76973485	G	T	0.037	0.195	0.042	3.3498E-06	-0.021	0.135	0.874	49.554
RuminococcaceaeUCG013	rs9313055	T	C	0.069	0.105	0.023	9.54713E-06	0.014	0.098	0.885	25.915
RuminococcaceaeUCG014	rs10495392	C	T	0.119	-0.082	0.019	9.96329E-06	0.208	0.103	0.043	26.256
RuminococcaceaeUCG014	rs10791168	A	G	0.184	-0.066	0.015	9.76182E-06	-0.015	0.070	0.831	24.346
RuminococcaceaeUCG014	rs10941294	C	T	0.069	-0.122	0.026	2.397E-06	-0.027	0.119	0.823	34.978
RuminococcaceaeUCG014	rs115777838	T	C	0.054	-0.188	0.039	4.62331E-07	-0.036	0.086	0.673	66.356
RuminococcaceaeUCG014	rs12638134	T	G	0.390	0.058	0.012	1.21144E-06	-0.012	0.054	0.825	29.650

RuminococcaceaeUCG014	rs34402072	C	T	0.192	-0.069	0.016	9.80426E-06	-0.153	0.076	0.043	26.952
RuminococcaceaeUCG014	rs56105232	G	A	0.073	0.139	0.030	2.9128E-06	-0.066	0.113	0.556	48.026
RuminococcaceaeUCG014	rs72809222	T	C	0.252	0.067	0.014	2.41191E-06	0.086	0.065	0.189	31.210
RuminococcaceaeUCG014	rs853612	A	G	0.403	-0.053	0.012	9.75451E-06	-0.031	0.055	0.575	24.649
RuminococcaceaeUCG014	rs995642	C	T	0.326	0.060	0.013	1.90222E-06	-0.023	0.063	0.720	29.103
Ruminococcus1	rs10167839	A	G	0.344	0.052	0.012	8.09254E-06	-0.036	0.056	0.519	22.361
Ruminococcus1	rs11783695	G	T	0.151	-0.073	0.016	4.72679E-06	-0.017	0.073	0.814	25.388
Ruminococcus1	rs17781867	C	T	0.092	0.100	0.021	1.96335E-06	0.029	0.105	0.786	30.764
Ruminococcus1	rs3819978	C	T	0.059	-0.115	0.026	8.74133E-06	-0.161	0.102	0.115	26.815
Ruminococcus1	rs6105066	T	C	0.250	-0.061	0.013	5.05914E-06	0.047	0.061	0.437	25.343
Ruminococcus1	rs6493760	C	T	0.397	0.054	0.012	3.38363E-06	0.038	0.056	0.500	25.181
Ruminococcus1	rs7117576	A	G	0.089	0.083	0.017	6.47979E-07	-0.123	0.093	0.187	20.380
Ruminococcus1	rs7583465	C	T	0.377	0.053	0.011	2.55613E-06	-0.018	0.054	0.740	24.007
Ruminococcus1	rs78572139	G	A	0.071	0.125	0.028	5.23105E-06	-0.089	0.089	0.321	37.702
Ruminococcus1	rs78613526	G	A	0.043	0.167	0.037	5.11425E-06	0.044	0.122	0.720	42.157
Ruminococcus2	rs12406309	A	C	0.191	-0.063	0.014	9.78953E-06	-0.012	0.065	0.853	22.650
Ruminococcus2	rs12986628	C	T	0.213	0.067	0.014	2.14202E-06	0.030	0.067	0.652	27.277
Ruminococcus2	rs1819812	G	T	0.116	0.084	0.018	5.28435E-06	-0.091	0.118	0.439	26.785
Ruminococcus2	rs2368224	T	G	0.048	0.200	0.044	3.6293E-06	-0.143	0.124	0.246	66.647
Ruminococcus2	rs2846589	G	T	0.454	0.052	0.012	7.58896E-06	-0.020	0.055	0.710	24.818
Ruminococcus2	rs2997412	A	G	0.280	-0.057	0.012	4.22116E-06	0.058	0.060	0.330	23.938

Ruminococcus2	rs4400279	A	G	0.364	0.055	0.012	5.79929E-06	-0.081	0.057	0.157	25.352
Ruminococcus2	rs4799823	C	T	0.139	0.084	0.018	5.40138E-06	-0.038	0.070	0.584	30.839
Ruminococcus2	rs55707116	C	A	0.102	0.087	0.019	8.00593E-06	-0.020	0.101	0.843	25.288
Ruminococcus2	rs58681734	A	G	0.151	0.072	0.016	4.18175E-06	-0.036	0.066	0.588	24.684
Ruminococcus2	rs61791565	T	C	0.369	-0.052	0.012	6.78933E-06	-0.050	0.054	0.355	23.428
Ruminococcus2	rs75140805	T	G	0.158	0.084	0.018	3.95297E-06	-0.014	0.071	0.841	34.249
Ruminococcus2	rs7635831	G	A	0.293	0.062	0.013	1.98054E-06	0.005	0.056	0.935	29.107
Ruminococcus2	rs7693984	G	A	0.083	-0.103	0.024	9.42134E-06	-0.060	0.129	0.641	29.397
Ruminococcus2	rs78120384	A	G	0.073	-0.193	0.039	3.31185E-07	0.047	0.093	0.611	92.268
Ruminococcusgauvreauiigroup	rs10931481	G	A	0.311	0.061	0.013	3.38211E-06	0.025	0.059	0.676	29.304
Ruminococcusgauvreauiigroup	rs12079579	A	G	0.073	0.096	0.021	5.03725E-06	-0.057	0.096	0.556	22.564
Ruminococcusgauvreauiigroup	rs12539819	C	T	0.095	0.111	0.024	4.49375E-06	-0.068	0.106	0.520	38.836
Ruminococcusgauvreauiigroup	rs1391597	C	T	0.381	0.059	0.012	1.85964E-06	-0.048	0.055	0.382	30.179
Ruminococcusgauvreauiigroup	rs2047242	A	G	0.297	-0.068	0.013	2.46372E-07	0.031	0.064	0.623	35.077
Ruminococcusgauvreauiigroup	rs2166943	A	C	0.438	0.057	0.012	5.27717E-06	-0.037	0.054	0.494	29.073
Ruminococcusgauvreauiigroup	rs289410	G	A	0.268	-0.065	0.014	2.27464E-06	0.060	0.060	0.315	30.942
Ruminococcusgauvreauiigroup	rs431418	A	G	0.119	-0.095	0.021	5.53667E-06	-0.056	0.092	0.543	34.651
Ruminococcusgauvrea	rs71386687	T	G	0.060	0.121	0.024	2.90761E-07	-0.114	0.085	0.182	30.164

uiigroup											
Ruminococcusgauvreauiigroup	rs73802842	C	A	0.143	0.074	0.017	7.48026E-06	0.007	0.063	0.909	24.448
Ruminococcusgauvreauiigroup	rs9870933	A	G	0.409	0.062	0.013	8.4912E-07	0.023	0.055	0.676	34.310
Ruminococcusgnavusgroup	rs11597105	A	G	0.112	0.115	0.025	6.94868E-06	-0.086	0.068	0.206	48.205
Ruminococcusgnavusgroup	rs11864644	T	C	0.071	-0.140	0.032	5.0088E-06	-0.022	0.084	0.790	47.157
Ruminococcusgnavusgroup	rs12136548	C	T	0.291	0.090	0.020	3.09631E-06	-0.039	0.059	0.511	61.803
Ruminococcusgnavusgroup	rs12989336	G	A	0.305	-0.085	0.019	7.11808E-06	0.094	0.060	0.118	55.947
Ruminococcusgnavusgroup	rs13163520	G	A	0.181	-0.127	0.023	5.61015E-08	0.002	0.069	0.981	88.600
Ruminococcusgnavusgroup	rs2909242	C	A	0.386	-0.091	0.018	7.40957E-07	-0.056	0.057	0.325	72.238
Ruminococcusgnavusgroup	rs3124783	A	G	0.120	-0.116	0.025	2.6652E-06	-0.160	0.079	0.044	52.367
Ruminococcusgnavusgroup	rs4388134	C	T	0.246	-0.090	0.020	9.11637E-06	0.086	0.060	0.153	55.809
Ruminococcusgnavusgroup	rs62167033	T	C	0.071	0.185	0.040	3.49529E-06	0.010	0.134	0.938	82.995
Ruminococcusgnavusgroup	rs78399089	T	C	0.083	0.144	0.033	6.62998E-06	-0.033	0.084	0.697	58.106

Ruminococcusgnavusgroup	rs934940	A	C	0.185	-0.105	0.023	2.73705E-06	0.055	0.078	0.479	61.197
Ruminococcustorques group	rs10904297	A	G	0.030	-0.168	0.039	2.68713E-06	0.056	0.184	0.761	29.907
Ruminococcustorques group	rs10967781	C	A	0.318	0.051	0.011	8.36865E-06	0.002	0.059	0.975	20.553
Ruminococcustorques group	rs12434631	A	G	0.140	0.075	0.015	2.77247E-06	-0.028	0.087	0.744	24.701
Ruminococcustorques group	rs1475330	T	C	0.301	0.052	0.012	8.13435E-06	0.000	0.063	0.998	21.179
Ruminococcustorques group	rs35866622	T	C	0.440	-0.061	0.011	2.21337E-08	0.105	0.056	0.064	33.919
Ruminococcustorques group	rs4073731	T	C	0.176	0.065	0.014	4.0525E-06	0.057	0.072	0.431	22.625
Ruminococcustorques group	rs77034621	T	G	0.053	-0.152	0.034	6.06872E-06	-0.302	0.206	0.144	42.151
Sellimonas	rs113379006	T	C	0.168	-0.163	0.036	7.20777E-06	0.034	0.071	0.626	136.814
Sellimonas	rs13417181	T	C	0.216	0.167	0.034	7.61748E-07	-0.035	0.064	0.584	173.654
Sellimonas	rs2016057	A	C	0.391	-0.126	0.026	1.02698E-06	0.034	0.055	0.536	139.393
Sellimonas	rs2187447	A	C	0.061	0.243	0.053	3.97806E-06	-0.026	0.112	0.814	124.592
Sellimonas	rs2371572	A	C	0.549	0.127	0.025	4.46357E-07	0.078	0.054	0.146	148.482
Sellimonas	rs41816	A	G	0.252	0.132	0.029	8.39372E-06	-0.057	0.058	0.326	121.465
Sellimonas	rs4600608	A	G	0.239	-0.137	0.030	4.94843E-06	-0.109	0.066	0.097	126.196
Sellimonas	rs553697	T	C	0.162	-0.154	0.034	6.12773E-06	0.128	0.070	0.067	118.486
Sellimonas	rs56203279	T	C	0.330	-0.124	0.027	3.71999E-06	0.043	0.057	0.451	125.630
Senegalimassilia	rs10036909	C	T	0.070	0.186	0.040	8.05438E-06	0.221	0.136	0.103	82.107

Senegalimassilia	rs11787826	C	A	0.428	0.081	0.017	2.63466E-06	-0.040	0.054	0.466	59.594
Senegalimassilia	rs1990708	A	C	0.126	-0.110	0.025	8.90578E-06	-0.021	0.099	0.836	48.728
Senegalimassilia	rs2017373	C	T	0.374	0.078	0.018	9.50288E-06	0.036	0.056	0.516	52.684
Senegalimassilia	rs7225245	G	A	0.458	0.079	0.017	4.18017E-06	-0.048	0.054	0.375	57.253
Slackia	rs10409783	A	G	0.283	0.095	0.021	7.69592E-06	0.089	0.059	0.134	67.572
Slackia	rs12440440	A	G	0.309	0.090	0.019	2.62872E-06	-0.132	0.056	0.019	63.938
Slackia	rs16894137	C	T	0.143	-0.123	0.026	2.71183E-06	0.027	0.080	0.735	68.061
Slackia	rs35156985	T	C	0.064	-0.156	0.035	8.05947E-06	0.008	0.134	0.952	53.111
Slackia	rs4492265	A	G	0.310	-0.091	0.019	2.40925E-06	-0.033	0.058	0.573	64.598
Slackia	rs8901	C	T	0.371	0.093	0.019	6.06998E-07	-0.028	0.059	0.632	75.046
Streptococcus	rs10028567	C	T	0.082	-0.092	0.019	7.30343E-06	-0.024	0.082	0.770	23.326
Streptococcus	rs10448310	A	G	0.430	-0.052	0.011	3.30706E-06	0.003	0.056	0.963	24.152
Streptococcus	rs11110281	T	C	0.068	-0.138	0.023	2.58314E-09	0.049	0.123	0.691	43.822
Streptococcus	rs11720390	G	A	0.075	0.107	0.023	3.59482E-06	0.185	0.112	0.097	29.047
Streptococcus	rs11764382	A	G	0.191	-0.070	0.014	1.28631E-06	0.033	0.077	0.668	27.431
Streptococcus	rs17708276	A	G	0.128	-0.079	0.017	3.04096E-06	0.002	0.086	0.984	25.876
Streptococcus	rs1918540	G	A	0.253	0.060	0.013	2.44067E-06	0.058	0.070	0.402	24.655
Streptococcus	rs2370083	G	T	0.089	-0.082	0.019	9.75245E-06	0.072	0.110	0.514	19.761
Streptococcus	rs57646748	G	A	0.068	-0.091	0.020	5.4754E-06	0.116	0.138	0.399	19.066
Streptococcus	rs6806351	T	C	0.209	-0.063	0.014	4.93869E-06	0.045	0.065	0.484	24.365
Streptococcus	rs71481756	T	G	0.099	0.093	0.021	6.51476E-06	-0.129	0.109	0.237	28.505
Streptococcus	rs7916711	A	G	0.065	0.103	0.022	2.71702E-06	-0.100	0.078	0.197	23.492
Subdoligranulum	rs10065321	T	C	0.368	-0.051	0.011	2.10155E-06	-0.082	0.054	0.133	22.456
Subdoligranulum	rs10497836	C	T	0.280	-0.052	0.012	8.37954E-06	0.031	0.065	0.633	20.343
Subdoligranulum	rs1667315	G	A	0.437	0.049	0.011	6.72143E-06	0.051	0.055	0.357	21.256
Subdoligranulum	rs2114677	C	T	0.056	-0.104	0.023	2.71726E-06	0.003	0.084	0.974	20.960

Subdoligranulum	rs2171249	C	T	0.074	0.107	0.023	4.50556E-06	0.023	0.102	0.824	28.538
Subdoligranulum	rs35940633	G	A	0.363	-0.051	0.011	4.21698E-06	-0.052	0.057	0.360	22.167
Subdoligranulum	rs3761728	T	G	0.243	-0.054	0.012	3.86747E-06	-0.069	0.062	0.265	19.920
Subdoligranulum	rs4347804	A	G	0.042	0.166	0.036	2.18405E-06	-0.068	0.151	0.652	40.506
Subdoligranulum	rs6555306	T	C	0.122	-0.074	0.016	2.80664E-06	0.034	0.077	0.662	21.629
Subdoligranulum	rs75158211	T	C	0.111	-0.072	0.016	7.52162E-06	0.000	0.075	0.997	19.005
Subdoligranulum	rs76528319	G	T	0.040	-0.143	0.031	7.41394E-06	0.095	0.097	0.326	28.818
Sutterella	rs1145877	A	G	0.159	-0.074	0.016	7.19949E-06	-0.047	0.078	0.544	26.567
Sutterella	rs11591622	T	G	0.216	-0.069	0.015	6.49857E-06	0.063	0.073	0.388	29.449
Sutterella	rs13173038	A	G	0.221	-0.072	0.015	2.72984E-06	0.073	0.062	0.239	32.584
Sutterella	rs143438747	T	C	0.055	-0.146	0.031	3.28021E-06	0.056	0.101	0.578	40.399
Sutterella	rs2050185	G	A	0.378	0.058	0.013	7.96955E-06	-0.018	0.056	0.747	28.559
Sutterella	rs2321387	G	A	0.530	-0.059	0.012	1.86519E-06	-0.096	0.054	0.074	32.171
Sutterella	rs2613606	C	T	0.394	-0.056	0.012	7.2023E-06	0.147	0.053	0.006	27.179
Sutterella	rs607327	C	T	0.377	0.058	0.013	6.6306E-06	0.074	0.055	0.179	28.848
Sutterella	rs62501473	G	A	0.229	0.069	0.015	5.51851E-06	-0.011	0.061	0.852	31.224
Sutterella	rs7499539	A	G	0.323	0.062	0.013	2.35769E-06	-0.061	0.061	0.317	30.635
Sutterella	rs7638039	T	C	0.233	0.065	0.014	8.66397E-06	-0.007	0.062	0.905	27.329
Sutterella	rs9350083	T	G	0.299	-0.059	0.013	8.22778E-06	-0.030	0.056	0.595	27.089
Terrisporobacter	rs1883097	C	T	0.056	0.226	0.045	4.15789E-07	0.206	0.140	0.141	99.413
Terrisporobacter	rs2569953	A	C	0.420	-0.078	0.017	8.95009E-06	-0.115	0.054	0.033	53.883
Terrisporobacter	rs2872237	C	A	0.434	-0.081	0.018	3.97135E-06	-0.069	0.055	0.207	59.977
Terrisporobacter	rs58405430	G	T	0.115	0.135	0.030	7.9371E-06	-0.111	0.112	0.322	68.048
Terrisporobacter	rs7184125	T	C	0.233	0.091	0.021	8.48103E-06	-0.040	0.060	0.508	54.680
Turicibacter	rs11054680	T	C	0.096	-0.105	0.023	2.30977E-06	-0.030	0.071	0.670	35.122
Turicibacter	rs11666533	C	T	0.080	-0.112	0.025	7.37109E-06	0.039	0.099	0.693	33.542

Turicibacter	rs149744580	A	G	0.053	0.170	0.032	7.00965E-08	-0.150	0.120	0.209	52.995
Turicibacter	rs2834977	T	C	0.173	-0.096	0.021	3.95588E-06	0.099	0.075	0.185	48.486
Turicibacter	rs2952020	G	A	0.223	-0.076	0.017	5.63308E-06	-0.019	0.062	0.767	36.649
Turicibacter	rs3734633	G	A	0.097	-0.121	0.027	5.31914E-06	0.154	0.113	0.173	47.295
Turicibacter	rs4869133	G	A	0.089	0.131	0.027	2.55369E-06	0.027	0.071	0.707	51.058
Turicibacter	rs55756211	T	C	0.116	-0.115	0.024	2.80533E-06	0.001	0.103	0.989	50.086
Turicibacter	rs7199484	G	A	0.332	-0.073	0.016	5.76664E-06	-0.056	0.058	0.336	43.619
Tyzzerella3	rs10898797	C	T	0.157	0.122	0.027	8.85446E-06	-0.019	0.086	0.829	73.027
Tyzzerella3	rs112102233	A	G	0.067	-0.216	0.048	6.17669E-06	0.154	0.126	0.222	107.341
Tyzzerella3	rs1232220	G	T	0.126	-0.144	0.032	7.90792E-06	0.076	0.088	0.387	84.096
Tyzzerella3	rs17706273	T	C	0.152	-0.140	0.027	5.87816E-07	0.085	0.099	0.390	93.671
Tyzzerella3	rs191093	G	A	0.080	0.159	0.035	6.76393E-06	0.181	0.088	0.039	68.112
Tyzzerella3	rs4904512	T	C	0.199	-0.117	0.025	3.09269E-06	-0.054	0.080	0.503	80.526
Tyzzerella3	rs55799124	A	G	0.207	-0.114	0.024	1.3355E-06	0.123	0.061	0.044	79.005
Tyzzerella3	rs67476743	T	G	0.285	0.132	0.022	3.74049E-09	0.040	0.060	0.507	131.564
Tyzzerella3	rs7019909	T	C	0.101	0.144	0.030	1.7612E-06	-0.024	0.083	0.774	69.710
Tyzzerella3	rs7333521	T	C	0.043	-0.207	0.045	4.87602E-06	-0.103	0.151	0.494	64.585
Tyzzerella3	rs75091807	G	T	0.091	-0.185	0.038	1.70674E-06	0.030	0.112	0.787	103.865
Tyzzerella3	rs7561370	T	C	0.122	0.131	0.029	1.51605E-06	-0.086	0.075	0.251	68.166
unknowngenus	rs10517600	T	G	0.536	0.063	0.014	6.82768E-06	0.074	0.055	0.177	35.931
unknowngenus	rs10904722	C	T	0.281	-0.067	0.015	5.04837E-06	0.059	0.063	0.350	33.577
unknowngenus	rs118104867	C	T	0.049	0.214	0.046	3.43598E-06	0.142	0.107	0.184	78.486
unknowngenus	rs13409132	A	G	0.045	-0.165	0.035	4.37231E-06	0.111	0.141	0.433	42.955
unknowngenus	rs17121075	G	A	0.168	0.077	0.017	7.91417E-06	-0.020	0.065	0.757	30.386
unknowngenus	rs2191834	G	T	0.240	0.075	0.016	2.50195E-06	-0.045	0.063	0.476	37.300
unknowngenus	rs28691777	C	T	0.072	0.137	0.027	6.95697E-07	-0.024	0.132	0.857	45.963

unknowngenus	rs34088226	A	G	0.071	-0.118	0.027	7.66216E-06	0.111	0.115	0.334	33.460
unknowngenus	rs55682560	C	T	0.070	-0.132	0.026	4.97034E-07	0.053	0.099	0.596	41.173
unknowngenus	rs6588624	G	A	0.471	-0.066	0.014	1.79287E-06	0.068	0.054	0.206	40.175
unknowngenus	rs66714985	A	C	0.085	0.117	0.025	4.85334E-06	-0.098	0.088	0.265	38.860
unknowngenus	rs7226487	A	G	0.476	-0.064	0.014	3.58282E-06	0.002	0.054	0.976	37.981
unknowngenus	rs7538034	T	G	0.208	-0.079	0.017	2.36706E-06	-0.010	0.073	0.893	37.374
unknowngenus	rs7725895	A	G	0.090	-0.116	0.024	3.94356E-06	-0.039	0.083	0.638	40.461
unknowngenus	rs989682	A	G	0.282	0.070	0.016	6.8471E-06	0.088	0.064	0.164	36.686
unknowngenus	rs11150282	T	C	0.321	0.098	0.020	7.36053E-07	0.017	0.056	0.764	77.480
unknowngenus	rs113884518	T	C	0.049	-0.206	0.046	7.73987E-06	-0.236	0.171	0.168	72.326
unknowngenus	rs28678345	T	C	0.047	0.213	0.047	8.05953E-06	0.034	0.125	0.788	74.436
unknowngenus	rs367480	G	A	0.427	-0.084	0.019	7.52418E-06	0.070	0.056	0.210	63.803
unknowngenus	rs4129395	G	A	0.426	0.090	0.019	1.21742E-06	0.009	0.054	0.862	73.644
unknowngenus	rs789069	A	C	0.172	-0.104	0.023	6.50003E-06	0.027	0.077	0.728	56.811
unknowngenus	rs79790072	T	C	0.057	0.226	0.049	3.5381E-06	-0.119	0.156	0.446	100.834
unknowngenus	rs8028558	A	G	0.405	0.083	0.019	9.78236E-06	0.022	0.056	0.697	61.749
unknowngenus	rs9864379	T	C	0.113	-0.161	0.029	4.65873E-08	-0.143	0.076	0.060	95.435
unknowngenus	rs11779863	G	A	0.207	-0.077	0.017	6.68845E-06	0.128	0.074	0.083	35.997
unknowngenus	rs12566890	T	G	0.095	-0.103	0.024	8.11201E-06	-0.105	0.080	0.188	33.728
unknowngenus	rs13100746	C	T	0.410	0.064	0.014	7.28846E-06	-0.078	0.054	0.146	36.282
unknowngenus	rs17235252	T	C	0.094	-0.122	0.026	2.15879E-06	-0.115	0.083	0.166	46.788
unknowngenus	rs3932485	C	T	0.428	0.063	0.014	9.92857E-06	-0.076	0.055	0.168	35.286
unknowngenus	rs515984	T	C	0.174	-0.088	0.019	6.61366E-06	0.195	0.084	0.019	40.489
unknowngenus	rs638542	G	A	0.286	-0.071	0.016	5.17285E-06	-0.054	0.059	0.358	37.421
unknowngenus	rs74603314	T	C	0.046	0.231	0.049	2.27641E-06	-0.026	0.137	0.850	85.576
unknowngenus	rs7706512	G	A	0.504	0.066	0.014	2.27207E-06	0.000	0.054	1.000	39.703

unknowngenus	rs7801843	A	G	0.129	-0.087	0.019	9.46882E-06	-0.060	0.075	0.418	31.249
unknowngenus	rs7853673	G	A	0.514	-0.062	0.014	6.7325E-06	0.020	0.054	0.704	35.789
unknowngenus	rs949341	G	A	0.320	0.066	0.015	7.73282E-06	0.030	0.060	0.620	34.477
unknowngenus	rs10872669	A	G	0.107	-0.123	0.028	9.4901E-06	0.164	0.093	0.079	53.409
unknowngenus	rs12748533	G	T	0.297	-0.082	0.017	2.59019E-06	0.103	0.059	0.083	51.795
unknowngenus	rs17043785	T	C	0.060	-0.176	0.035	5.11837E-07	0.036	0.094	0.703	64.033
unknowngenus	rs61508842	T	C	0.122	0.123	0.027	7.82782E-06	0.039	0.095	0.684	59.341
unknowngenus	rs689695	C	A	0.366	0.081	0.017	1.28039E-06	-0.099	0.059	0.093	56.631
unknowngenus	rs738193	T	C	0.355	0.085	0.017	3.82163E-07	-0.082	0.056	0.145	60.485
unknowngenus	rs78609301	A	G	0.217	-0.087	0.020	7.09297E-06	0.012	0.059	0.835	46.923
unknowngenus	rs941000	C	T	0.409	0.085	0.016	3.15557E-07	-0.063	0.056	0.257	64.292
unknowngenus	rs11251024	G	A	0.330	0.104	0.021	6.62801E-07	-0.027	0.061	0.654	88.472
unknowngenus	rs11606187	A	G	0.107	-0.155	0.033	3.30586E-06	-0.014	0.077	0.853	84.355
unknowngenus	rs13385922	T	C	0.406	0.093	0.020	3.96829E-06	-0.023	0.056	0.685	76.712
unknowngenus	rs166849	G	A	0.390	0.091	0.020	7.73898E-06	0.015	0.054	0.784	72.688
unknowngenus	rs2172426	C	T	0.447	-0.102	0.020	3.17425E-07	-0.014	0.055	0.800	95.046
unknowngenus	rs267959	A	G	0.343	0.099	0.021	2.61993E-06	0.075	0.060	0.209	81.124
unknowngenus	rs4383094	T	C	0.096	0.149	0.032	4.28134E-06	0.050	0.077	0.519	71.382
unknowngenus	rs60775321	T	C	0.301	-0.096	0.021	7.1046E-06	-0.028	0.059	0.637	71.783
unknowngenus	rs72671304	T	C	0.070	0.172	0.037	3.79578E-06	0.169	0.102	0.096	70.797
unknowngenus	rs7911787	G	T	0.066	-0.223	0.047	3.38824E-06	0.307	0.152	0.044	112.554
unknowngenus	rs8126061	T	C	0.095	-0.159	0.035	7.36334E-06	0.010	0.081	0.902	80.505
unknowngenus	rs9542068	T	C	0.321	0.099	0.022	6.52339E-06	0.060	0.057	0.290	78.810
unknowngenus	rs1044939	C	T	0.103	0.109	0.024	7.01695E-06	-0.073	0.089	0.414	40.503
unknowngenus	rs115372687	A	G	0.087	0.109	0.024	9.12788E-06	0.100	0.095	0.291	34.493
unknowngenus	rs12634544	T	C	0.255	-0.081	0.016	2.60324E-07	-0.040	0.059	0.496	46.115

unknowngenus	rs12930556	C	T	0.449	0.064	0.014	4.54295E-06	-0.127	0.054	0.019	37.492
unknowngenus	rs17291611	A	G	0.173	-0.091	0.019	2.29784E-06	0.023	0.076	0.765	43.143
unknowngenus	rs3010848	G	A	0.336	-0.070	0.015	1.78295E-06	-0.027	0.056	0.625	39.807
unknowngenus	rs35947731	C	T	0.060	-0.131	0.029	5.43191E-06	-0.196	0.093	0.034	35.462
unknowngenus	rs4848741	A	G	0.348	-0.068	0.015	5.52765E-06	0.067	0.063	0.285	38.830
unknowngenus	rs72819830	A	G	0.049	0.227	0.047	1.6142E-06	0.032	0.163	0.843	87.793
unknowngenus	rs111373036	A	G	0.195	-0.086	0.018	1.70306E-06	-0.012	0.076	0.871	42.473
unknowngenus	rs11140959	A	G	0.057	0.190	0.043	6.20124E-06	-0.072	0.078	0.354	71.391
unknowngenus	rs12696594	A	G	0.506	-0.064	0.014	6.10947E-06	-0.052	0.054	0.333	37.187
unknowngenus	rs2136103	T	C	0.304	0.075	0.015	6.72529E-07	-0.014	0.061	0.812	44.303
unknowngenus	rs2599390	G	A	0.170	0.094	0.020	3.03424E-06	0.148	0.081	0.067	45.888
unknowngenus	rs55736413	C	T	0.051	0.158	0.036	8.4481E-06	0.043	0.127	0.736	44.246
unknowngenus	rs7334707	T	G	0.079	0.119	0.027	6.25037E-06	-0.074	0.124	0.547	37.643
unknowngenus	rs79929882	A	G	0.137	-0.104	0.023	5.91837E-06	-0.050	0.081	0.534	47.495
unknowngenus	rs1032598	G	A	0.235	-0.089	0.019	4.07584E-06	-0.016	0.072	0.820	51.893
unknowngenus	rs11941716	A	G	0.161	0.101	0.022	9.0663E-06	-0.023	0.075	0.762	50.922
unknowngenus	rs16843660	A	G	0.046	0.235	0.049	1.75342E-06	-0.257	0.151	0.089	88.530
unknowngenus	rs1962916	A	G	0.394	0.074	0.016	6.13846E-06	-0.028	0.055	0.607	47.785
unknowngenus	rs2336448	T	C	0.379	0.077	0.016	1.42898E-06	0.035	0.054	0.515	51.808
unknowngenus	rs249459	A	G	0.362	0.074	0.017	8.1231E-06	-0.017	0.056	0.760	46.157
unknowngenus	rs2651663	A	G	0.346	-0.076	0.017	5.64147E-06	-0.046	0.055	0.406	48.380
unknowngenus	rs35703006	G	T	0.216	0.093	0.019	9.00771E-07	-0.047	0.062	0.445	53.435
unknowngenus	rs553072	G	A	0.135	0.109	0.023	3.69096E-06	0.123	0.070	0.076	51.272
unknowngenus	rs6514318	T	C	0.085	0.128	0.028	5.3768E-06	0.142	0.124	0.253	46.748
unknowngenus	rs7187855	A	C	0.050	0.200	0.042	2.20601E-06	0.106	0.127	0.404	69.510
unknowngenus	rs921383	G	A	0.551	0.072	0.016	7.72897E-06	0.119	0.055	0.030	47.579

unknowngenus	rs10200320	C	T	0.287	0.064	0.014	5.66074E-06	-0.006	0.066	0.928	30.921
unknowngenus	rs11195523	A	C	0.249	0.069	0.015	2.40123E-06	-0.012	0.062	0.846	32.599
unknowngenus	rs11684166	A	G	0.179	-0.077	0.017	3.49119E-06	0.001	0.070	0.989	31.968
unknowngenus	rs11809762	G	A	0.126	-0.093	0.019	1.68286E-06	-0.002	0.068	0.974	35.398
unknowngenus	rs11904514	G	A	0.077	-0.109	0.025	7.89959E-06	-0.107	0.110	0.331	31.119
unknowngenus	rs12147596	C	T	0.268	-0.072	0.014	2.86206E-07	-0.123	0.058	0.034	37.391
unknowngenus	rs16823675	C	T	0.217	-0.077	0.015	2.33346E-07	0.152	0.066	0.021	36.746
unknowngenus	rs17086536	C	A	0.096	-0.101	0.022	3.36379E-06	-0.006	0.103	0.955	32.551
unknowngenus	rs2939766	A	G	0.409	-0.059	0.013	7.01143E-06	-0.001	0.054	0.982	31.070
unknowngenus	rs34985298	G	A	0.292	-0.062	0.014	8.33749E-06	0.140	0.055	0.011	29.538
unknowngenus	rs35740166	C	T	0.107	-0.112	0.023	8.39977E-07	0.042	0.092	0.646	44.405
unknowngenus	rs4644504	T	C	0.079	-0.097	0.022	5.81965E-06	-0.058	0.107	0.583	24.962
unknowngenus	rs6007642	C	T	0.169	-0.079	0.018	9.9554E-06	-0.130	0.065	0.044	32.318
unknowngenus	rs72700702	T	C	0.137	-0.092	0.019	1.59273E-06	-0.022	0.085	0.799	36.601
unknowngenus	rs76532867	T	C	0.105	0.112	0.024	2.55861E-06	-0.031	0.136	0.822	43.758
unknowngenus	rs1035406	G	A	0.099	-0.111	0.025	9.17532E-06	0.069	0.083	0.406	40.718
unknowngenus	rs11630875	T	C	0.182	0.093	0.020	6.93481E-06	0.093	0.080	0.245	46.907
unknowngenus	rs13336560	C	T	0.456	-0.072	0.016	5.29696E-06	-0.014	0.055	0.795	47.632
unknowngenus	rs1549633	A	C	0.156	0.102	0.022	3.39601E-06	-0.023	0.084	0.782	50.250
unknowngenus	rs2074723	T	C	0.068	0.166	0.038	8.75347E-06	-0.031	0.120	0.794	64.157
unknowngenus	rs3730086	A	G	0.239	0.081	0.018	8.64775E-06	0.070	0.062	0.252	43.468
unknowngenus	rs3754624	C	T	0.162	0.096	0.020	2.15693E-06	0.003	0.070	0.971	46.433
unknowngenus	rs61933850	G	A	0.091	0.165	0.036	7.42875E-06	-0.015	0.079	0.846	82.742
unknowngenus	rs7098347	G	A	0.423	0.071	0.016	7.36472E-06	-0.062	0.054	0.252	44.597
unknowngenus	rs74354280	C	T	0.153	-0.090	0.021	8.7006E-06	-0.004	0.060	0.953	38.886
unknowngenus	rs76784716	A	G	0.072	0.140	0.029	8.43037E-07	0.018	0.085	0.830	47.742

unknowngenus	rs9813022	A	G	0.302	-0.082	0.016	5.10261E-07	-0.033	0.055	0.553	52.509
unknowngenus	rs10060821	T	C	0.277	0.062	0.014	9.00351E-06	-0.003	0.063	0.960	28.683
unknowngenus	rs10782329	C	T	0.291	0.060	0.014	7.89888E-06	0.007	0.058	0.904	27.553
unknowngenus	rs11257188	A	C	0.100	-0.110	0.023	1.84757E-06	-0.118	0.081	0.142	39.811
unknowngenus	rs116151565	A	G	0.094	0.112	0.025	6.20451E-06	-0.091	0.083	0.274	39.110
unknowngenus	rs13147561	A	G	0.097	-0.099	0.023	9.56832E-06	-0.105	0.093	0.256	31.857
unknowngenus	rs142372196	A	G	0.156	-0.074	0.016	5.43706E-06	-0.065	0.090	0.470	26.223
unknowngenus	rs55922055	T	C	0.040	-0.240	0.048	2.27779E-07	0.006	0.152	0.968	80.886
unknowngenus	rs599752	C	T	0.252	-0.063	0.014	4.05511E-06	-0.151	0.060	0.012	27.462
unknowngenus	rs6431586	A	G	0.120	-0.095	0.021	2.72211E-06	0.139	0.098	0.156	35.404
unknowngenus	rs72846345	T	C	0.128	0.088	0.019	2.58479E-06	-0.048	0.097	0.623	31.629
unknowngenus	rs740358	G	T	0.075	-0.130	0.026	9.18134E-07	-0.160	0.109	0.144	42.993
unknowngenus	rs9571849	T	C	0.066	0.137	0.032	8.11643E-06	0.008	0.099	0.936	42.380
unknowngenus	rs999857	G	A	0.193	0.076	0.017	5.23925E-06	0.009	0.061	0.882	33.300
unknowngenus	rs10892421	C	T	0.395	-0.104	0.021	1.38588E-06	0.046	0.054	0.395	94.996
unknowngenus	rs115909738	A	C	0.155	-0.135	0.030	6.49132E-06	-0.030	0.070	0.664	87.524
unknowngenus	rs11691923	G	A	0.456	-0.092	0.021	8.35281E-06	-0.036	0.054	0.497	77.923
unknowngenus	rs12261792	T	G	0.187	-0.114	0.026	9.57331E-06	-0.025	0.072	0.728	72.368
unknowngenus	rs1969793	G	A	0.304	0.103	0.022	3.25688E-06	-0.068	0.058	0.236	82.198
unknowngenus	rs2317372	C	T	0.429	-0.095	0.021	7.92994E-06	0.012	0.055	0.825	80.844
unknowngenus	rs2831909	T	C	0.102	0.132	0.031	5.89797E-06	0.090	0.082	0.274	59.364
unknowngenus	rs4766410	C	T	0.408	0.097	0.021	5.33871E-06	-0.039	0.060	0.515	83.585
unknowngenus	rs66473610	T	G	0.079	0.181	0.040	8.79131E-06	0.086	0.083	0.296	87.724
unknowngenus	rs75285799	T	C	0.123	0.132	0.028	3.24795E-06	-0.138	0.074	0.062	69.565
unknowngenus	rs9534068	A	G	0.355	0.099	0.021	3.06718E-06	-0.062	0.055	0.264	82.229
Veillonella	rs1882878	A	G	0.306	-0.077	0.016	2.98089E-06	-0.107	0.059	0.068	46.188

Veillonella	rs2013594	T	C	0.358	-0.072	0.016	3.42129E-06	-0.096	0.055	0.080	43.882
Veillonella	rs62376424	C	T	0.334	-0.076	0.016	3.65453E-06	-0.007	0.058	0.905	47.521
Veillonella	rs6656807	A	G	0.425	0.070	0.015	5.49694E-06	0.033	0.056	0.554	44.396
Veillonella	rs742016	A	G	0.424	-0.069	0.015	4.65692E-06	-0.014	0.057	0.811	42.554
Victivallis	rs11899949	G	A	0.277	0.131	0.028	2.7671E-06	0.061	0.058	0.295	126.165
Victivallis	rs12512543	A	C	0.121	-0.178	0.037	2.5413E-06	0.077	0.098	0.430	124.751
Victivallis	rs173120	T	C	0.246	0.134	0.029	7.65253E-06	-0.085	0.068	0.212	122.450
Victivallis	rs1882775	A	G	0.224	-0.138	0.031	8.72833E-06	0.038	0.069	0.585	122.583
Victivallis	rs2546432	T	C	0.466	-0.111	0.025	9.93269E-06	-0.043	0.054	0.421	112.745
Victivallis	rs342302	A	G	0.159	-0.153	0.035	8.15946E-06	0.003	0.077	0.965	115.154
Victivallis	rs4764863	G	A	0.500	0.122	0.025	8.2222E-07	-0.058	0.054	0.284	136.499
Victivallis	rs4895919	T	C	0.482	-0.117	0.025	2.75199E-06	-0.035	0.054	0.512	126.037
Victivallis	rs56349194	A	G	0.160	-0.159	0.032	6.25748E-07	-0.076	0.079	0.334	124.678
Victivallis	rs911666	T	C	0.305	-0.119	0.026	7.65252E-06	-0.106	0.058	0.069	110.006
Actinomycetales	rs2889192	G	T	0.207	0.088	0.019	3.96723E-06	0.063	0.075	0.401	47.140
Actinomycetales	rs34583783	G	T	0.102	0.124	0.026	5.53884E-06	-0.069	0.112	0.537	51.718
Actinomycetales	rs35011108	A	G	0.064	0.242	0.050	1.87658E-06	-0.063	0.108	0.558	128.330
Actinomycetales	rs4073240	G	A	0.383	0.075	0.016	5.67895E-06	0.124	0.055	0.025	48.832
Bacillales	rs10233278	T	C	0.413	-0.116	0.025	3.50803E-06	0.007	0.054	0.895	121.051
Bacillales	rs10410917	T	C	0.596	0.115	0.025	5.57348E-06	-0.109	0.055	0.047	116.906
Bacillales	rs11034576	A	G	0.080	0.206	0.045	8.85974E-06	-0.086	0.081	0.291	114.344
Bacillales	rs11207728	G	A	0.203	-0.145	0.032	5.73457E-06	0.067	0.072	0.352	124.738
Bacillales	rs11844714	A	G	0.163	-0.143	0.032	5.06212E-06	0.044	0.066	0.507	103.251
Bacillales	rs1287018	G	A	0.160	0.141	0.032	9.87091E-06	-0.007	0.069	0.924	98.701
Bacillales	rs4617108	A	G	0.061	0.249	0.053	1.97506E-06	-0.034	0.096	0.719	130.224
Bacillales	rs74420793	A	G	0.162	-0.164	0.035	3.07323E-06	0.203	0.087	0.019	135.585

Bacteroidales	rs11146701	A	G	0.425	0.047	0.011	7.08228E-06	0.013	0.056	0.813	20.130
Bacteroidales	rs17343978	A	C	0.260	-0.055	0.012	8.35928E-06	-0.081	0.066	0.221	21.552
Bacteroidales	rs2032750	T	C	0.451	-0.051	0.011	1.91509E-06	-0.113	0.054	0.035	23.504
Bacteroidales	rs4916508	G	A	0.435	-0.047	0.011	8.47116E-06	-0.019	0.054	0.727	19.661
Bacteroidales	rs55773148	G	A	0.053	-0.122	0.024	3.89585E-07	-0.149	0.118	0.208	27.075
Bacteroidales	rs62531359	T	G	0.145	0.066	0.015	9.08665E-06	-0.023	0.070	0.745	19.583
Bacteroidales	rs62575403	C	T	0.044	0.140	0.031	7.06129E-06	-0.158	0.133	0.235	30.114
Bacteroidales	rs72706335	T	C	0.066	-0.222	0.049	7.65519E-06	-0.005	0.174	0.977	111.884
Bacteroidales	rs73975615	G	A	0.011	-0.207	0.044	1.21616E-06	0.065	0.316	0.837	16.962
Bacteroidales	rs7631304	G	A	0.170	-0.065	0.013	8.36781E-07	-0.036	0.076	0.631	21.606
Bacteroidales	rs79585701	A	C	0.129	0.065	0.015	9.98589E-06	0.131	0.077	0.088	17.280
Bacteroidales	rs929878	C	T	0.245	-0.055	0.012	4.73377E-06	0.045	0.065	0.495	20.407
Bifidobacteriales	rs10831953	G	A	0.333	0.054	0.012	9.94742E-06	0.035	0.058	0.554	23.568
Bifidobacteriales	rs12446429	T	C	0.136	0.081	0.019	8.52886E-06	0.068	0.069	0.324	28.361
Bifidobacteriales	rs13020688	G	A	0.353	0.058	0.012	1.57375E-06	0.003	0.058	0.964	28.614
Bifidobacteriales	rs182549	C	T	0.491	0.117	0.013	5.942E-20	-0.077	0.055	0.163	126.491
Bifidobacteriales	rs4957061	T	C	0.465	0.057	0.012	1.1517E-06	-0.033	0.055	0.546	29.680
Bifidobacteriales	rs540489	T	G	0.234	-0.063	0.014	5.36811E-06	-0.038	0.070	0.585	26.307
Bifidobacteriales	rs55888705	A	G	0.387	0.054	0.012	8.66208E-06	-0.074	0.059	0.213	25.092
Bifidobacteriales	rs6899771	A	G	0.095	-0.091	0.020	7.27995E-06	-0.011	0.089	0.905	26.497
Bifidobacteriales	rs7174549	C	T	0.356	0.055	0.012	6.8678E-06	-0.005	0.056	0.923	25.628
Bifidobacteriales	rs7322849	T	C	0.096	0.111	0.020	1.74032E-08	0.145	0.093	0.121	39.216
Bifidobacteriales	rs857444	C	T	0.361	0.055	0.012	3.82492E-06	-0.129	0.056	0.020	25.994
Burkholderiales	rs1511453	A	G	0.064	0.091	0.020	8.00097E-06	-0.323	0.124	0.009	18.145
Burkholderiales	rs1928341	A	G	0.422	0.051	0.011	4.51508E-06	-0.052	0.054	0.344	23.088
Burkholderiales	rs2321387	G	A	0.530	-0.051	0.011	3.25787E-06	-0.096	0.054	0.074	23.656

Burkholderiales	rs2613606	C	T	0.394	-0.050	0.011	4.13357E-06	0.147	0.053	0.006	21.872
Burkholderiales	rs4033856	C	T	0.129	0.083	0.017	5.66823E-07	0.165	0.095	0.083	28.708
Burkholderiales	rs6087811	T	G	0.082	-0.102	0.020	2.88374E-07	0.020	0.090	0.821	28.377
Burkholderiales	rs62191117	A	G	0.206	0.068	0.013	2.79099E-07	-0.016	0.066	0.808	27.760
Burkholderiales	rs62395635	T	C	0.071	0.110	0.024	2.90228E-06	0.021	0.113	0.850	29.118
Burkholderiales	rs75242906	C	T	0.054	-0.121	0.028	9.74519E-06	0.011	0.098	0.913	27.316
Burkholderiales	rs7638039	T	C	0.233	0.058	0.013	4.83593E-06	-0.007	0.062	0.905	22.124
Clostridiales	rs10774377	A	G	0.334	0.052	0.011	3.80899E-06	-0.138	0.054	0.011	22.343
Clostridiales	rs112334273	G	A	0.246	0.064	0.013	4.07257E-07	0.012	0.060	0.845	27.815
Clostridiales	rs13105690	T	C	0.267	-0.053	0.012	9.36806E-06	-0.025	0.060	0.678	20.009
Clostridiales	rs13179700	T	C	0.355	0.051	0.011	3.52111E-06	0.009	0.057	0.873	21.948
Clostridiales	rs1842454	G	A	0.207	-0.054	0.013	9.91744E-06	-0.035	0.069	0.608	17.800
Clostridiales	rs2273429	A	G	0.107	-0.073	0.015	4.16871E-06	-0.137	0.087	0.118	18.560
Clostridiales	rs290772	G	A	0.062	0.084	0.020	9.99759E-06	-0.034	0.119	0.775	15.062
Clostridiales	rs6442336	C	T	0.244	-0.055	0.012	9.62568E-06	-0.077	0.062	0.210	20.291
Clostridiales	rs6814436	T	C	0.132	0.074	0.015	9.06288E-07	-0.003	0.077	0.967	23.186
Clostridiales	rs6815608	T	C	0.085	0.104	0.021	3.71989E-07	0.012	0.075	0.873	30.792
Clostridiales	rs72738886	T	C	0.078	0.087	0.019	8.41577E-06	0.098	0.101	0.334	19.655
Clostridiales	rs72915163	T	C	0.265	-0.058	0.012	1.39104E-06	0.011	0.066	0.871	24.106
Coriobacteriales	rs11073596	T	G	0.340	0.051	0.011	8.14449E-06	0.062	0.055	0.267	21.449
Coriobacteriales	rs11250875	T	C	0.209	0.061	0.013	4.82844E-06	0.059	0.065	0.362	22.380
Coriobacteriales	rs11656361	A	C	0.129	0.077	0.018	8.02017E-06	-0.049	0.069	0.476	24.690
Coriobacteriales	rs12974142	G	A	0.108	0.079	0.018	8.51232E-06	-0.057	0.105	0.588	22.120
Coriobacteriales	rs13307134	C	T	0.236	0.057	0.013	7.79931E-06	-0.089	0.072	0.216	21.161
Coriobacteriales	rs1397793	G	A	0.386	-0.050	0.011	9.77381E-06	-0.016	0.059	0.785	21.651
Coriobacteriales	rs1816223	A	G	0.211	-0.059	0.013	4.84114E-06	0.060	0.067	0.374	20.988

Coriobacteriales	rs240104	T	C	0.245	-0.060	0.013	1.51603E-06	-0.062	0.060	0.295	24.702
Coriobacteriales	rs2442778	G	A	0.046	-0.116	0.026	9.02703E-06	0.035	0.122	0.772	21.700
Coriobacteriales	rs3025411	A	G	0.104	0.093	0.021	8.26912E-06	-0.043	0.088	0.623	29.487
Coriobacteriales	rs34739816	G	T	0.082	0.097	0.021	3.88358E-06	-0.142	0.114	0.215	25.603
Coriobacteriales	rs67561917	A	G	0.142	-0.071	0.015	5.39129E-06	-0.007	0.070	0.924	22.845
Coriobacteriales	rs719099	A	G	0.136	0.078	0.016	5.43317E-07	-0.017	0.090	0.855	26.182
Coriobacteriales	rs8010111	G	A	0.065	-0.103	0.023	6.89624E-06	-0.149	0.097	0.123	23.715
Desulfovibrionales	rs11599763	T	C	0.390	-0.055	0.012	2.60698E-06	-0.043	0.055	0.439	26.894
Desulfovibrionales	rs17791387	A	G	0.141	-0.073	0.015	2.25294E-06	0.137	0.091	0.135	23.569
Desulfovibrionales	rs186073	T	C	0.427	0.053	0.012	8.74432E-06	0.001	0.055	0.981	25.173
Desulfovibrionales	rs2692012	A	G	0.064	0.112	0.025	2.27021E-06	-0.164	0.118	0.165	27.560
Desulfovibrionales	rs2838334	G	A	0.323	0.057	0.012	4.16791E-06	0.116	0.056	0.040	26.009
Desulfovibrionales	rs3935584	C	T	0.496	-0.052	0.012	7.1974E-06	-0.066	0.054	0.220	25.254
Desulfovibrionales	rs4506934	C	T	0.112	-0.095	0.020	2.42998E-06	0.014	0.083	0.863	33.293
Desulfovibrionales	rs6058181	C	T	0.123	0.084	0.017	2.5277E-07	-0.056	0.073	0.438	27.801
Desulfovibrionales	rs62020470	A	G	0.259	-0.057	0.013	7.50981E-06	-0.161	0.070	0.021	23.214
Desulfovibrionales	rs72647048	T	C	0.116	-0.077	0.017	9.00294E-06	-0.085	0.084	0.314	22.519
Desulfovibrionales	rs9928243	C	A	0.425	-0.054	0.012	3.96636E-06	0.028	0.054	0.601	26.639
Enterobacteriales	rs11026530	T	C	0.134	0.082	0.019	9.42684E-06	0.152	0.075	0.044	28.868
Enterobacteriales	rs2374342	C	A	0.357	0.058	0.013	4.51618E-06	-0.032	0.054	0.554	28.649
Enterobacteriales	rs35673018	G	A	0.094	0.090	0.020	7.63109E-06	0.148	0.094	0.114	25.412
Enterobacteriales	rs504442	T	G	0.139	0.084	0.019	5.17092E-06	-0.051	0.088	0.562	31.179
Enterobacteriales	rs62210023	A	G	0.352	0.061	0.013	3.13163E-06	-0.029	0.056	0.612	30.846
Enterobacteriales	rs78143293	A	G	0.144	-0.085	0.017	1.19564E-06	0.029	0.082	0.725	32.626
Enterobacteriales	rs79757635	C	A	0.161	0.076	0.017	9.31857E-06	0.085	0.079	0.281	28.554
Erysipelotrichales	rs1074800	A	G	0.399	0.049	0.011	6.14583E-06	-0.128	0.054	0.019	21.345

Erysipelotrichales	rs10781552	C	T	0.312	-0.055	0.012	2.33114E-06	-0.055	0.060	0.352	24.043		
Erysipelotrichales	rs17530232	A	G	0.077	0.103	0.022	2.79123E-06	-0.198	0.119	0.096	27.557		
Erysipelotrichales	rs1884466	C	T	0.451	-0.048	0.011	9.52701E-06	-0.067	0.054	0.211	20.546		
Erysipelotrichales	rs2300774	G	A	0.408	0.052	0.011	8.95466E-07	-0.100	0.054	0.062	24.363		
Erysipelotrichales	rs290833	T	G	0.369	-0.050	0.011	8.029E-06	-0.007	0.054	0.897	21.145		
Erysipelotrichales	rs35161940	T	C	0.127	-0.081	0.017	1.8451E-06	-0.023	0.087	0.792	26.493		
Erysipelotrichales	rs4078432	C	T	0.181	-0.061	0.013	4.23104E-06	0.032	0.071	0.656	20.173		
Erysipelotrichales	rs56970041	T	G	0.111	0.072	0.016	5.40344E-06	0.241	0.111	0.030	19.043		
Erysipelotrichales	rs62504403	C	T	0.236	0.068	0.013	1.12265E-07	-0.123	0.068	0.068	30.697		
Erysipelotrichales	rs7234058	T	C	0.097	-0.095	0.019	9.12341E-07	0.056	0.093	0.545	28.886		
Erysipelotrichales	rs7826267	T	G	0.075	-0.084	0.020	9.28449E-06	-0.152	0.109	0.165	17.843		
Erysipelotrichales	rs8003149	C	T	0.282	0.054	0.012	4.08475E-06	-0.011	0.057	0.848	21.597		
Gastranaerophilales	rs11150282	T	C	0.321	0.098	0.020	7.36053E-07	0.017	0.056	0.764	77.480		
Gastranaerophilales	rs113884518	T	C	0.049	-0.206	0.046	7.73987E-06	-0.236	0.171	0.168	72.326		
Gastranaerophilales	rs28678345	T	C	0.047	0.213	0.047	8.05953E-06	0.034	0.125	0.788	74.436		
Gastranaerophilales	rs367480	G	A	0.427	-0.084	0.019	7.52418E-06	0.070	0.056	0.210	63.803		
Gastranaerophilales	rs4129395	G	A	0.426	0.090	0.019	1.21742E-06	0.009	0.054	0.862	73.644		
Gastranaerophilales	rs789069	A	C	0.172	-0.104	0.023	6.50003E-06	0.027	0.077	0.728	56.811		
Gastranaerophilales	rs79790072	T	C	0.057	0.226	0.049	3.5381E-06	-0.119	0.156	0.446	100.834		
Gastranaerophilales	rs8028558	A	G	0.405	0.083	0.019	9.78236E-06	0.022	0.056	0.697	61.749		
Gastranaerophilales	rs9864379	T	C	0.113	-0.161	0.029	4.65873E-08	-0.143	0.076	0.060	95.435		
Lactobacillales	rs11110282	A	G	0.068	-0.102	0.022	3.95665E-06	0.063	0.123	0.608	24.267		
Lactobacillales	rs11627423	C	A	0.390	0.050	0.011	5.08753E-06	-0.014	0.056	0.804	21.769		
Lactobacillales	rs11730038	G	A	0.241	-0.061	0.013	5.09746E-06	0.057	0.059	0.339	24.592		
Lactobacillales	rs12797734	T	C	0.253	0.057	0.013	7.76523E-06	0.035	0.063	0.580	22.620		
Lactobacillales	rs1595463	C	A	0.487	0.048	0.011	7.44354E-06	0.067	0.054	0.211	21.114		

Lactobacillales	rs2370083	G	T	0.089	-0.081	0.018	8.33495E-06	0.072	0.110	0.514	19.211
Lactobacillales	rs2952251	A	G	0.253	-0.063	0.012	3.36461E-07	-0.020	0.064	0.755	27.456
Lactobacillales	rs34989881	A	G	0.066	0.113	0.025	4.09354E-06	0.095	0.126	0.447	28.996
Lactobacillales	rs35344081	G	A	0.241	0.064	0.013	4.16066E-07	-0.026	0.061	0.663	27.699
Lactobacillales	rs4028634	T	C	0.425	0.053	0.011	1.34806E-06	-0.003	0.056	0.953	25.496
Lactobacillales	rs57872228	C	T	0.171	-0.069	0.015	2.58252E-06	-0.120	0.083	0.148	24.673
Lactobacillales	rs74663707	C	T	0.073	0.098	0.022	8.39558E-06	0.002	0.110	0.986	23.873
Lactobacillales	rs77558518	A	G	0.065	-0.106	0.022	1.67435E-06	-0.038	0.091	0.677	25.155
Lactobacillales	rs78938557	T	C	0.068	0.106	0.023	2.31087E-06	0.302	0.160	0.060	25.781
Lactobacillales	rs9581006	C	T	0.046	0.226	0.047	1.76596E-06	-0.234	0.141	0.097	81.937
Methanobacteriales	rs10202904	T	G	0.467	-0.122	0.024	3.0143E-07	-0.046	0.055	0.401	136.342
Methanobacteriales	rs10424197	G	A	0.325	-0.111	0.025	9.27887E-06	0.102	0.062	0.098	100.167
Methanobacteriales	rs4257531	G	A	0.114	0.164	0.036	7.44344E-06	-0.100	0.087	0.252	101.024
Methanobacteriales	rs6508769	T	C	0.165	0.154	0.034	8.22529E-06	-0.018	0.075	0.808	119.869
Methanobacteriales	rs6776814	T	C	0.090	-0.200	0.041	1.63062E-06	0.007	0.188	0.971	119.808
Methanobacteriales	rs73068003	G	T	0.122	-0.158	0.035	8.44583E-06	0.193	0.090	0.033	98.987
Methanobacteriales	rs73457410	A	G	0.067	0.215	0.044	1.40855E-06	-0.170	0.109	0.118	106.337
Methanobacteriales	rs75208022	C	T	0.077	-0.227	0.049	5.92103E-06	0.123	0.091	0.176	134.787
Methanobacteriales	rs894996	C	A	0.072	0.217	0.045	1.87557E-06	-0.096	0.105	0.360	115.524
MollicutesRF9	rs11779863	G	A	0.207	-0.077	0.017	6.68845E-06	0.128	0.074	0.083	35.997
MollicutesRF9	rs12566890	T	G	0.095	-0.103	0.024	8.11201E-06	-0.105	0.080	0.188	33.728
MollicutesRF9	rs13100746	C	T	0.410	0.064	0.014	7.28846E-06	-0.078	0.054	0.146	36.282
MollicutesRF9	rs17235252	T	C	0.094	-0.122	0.026	2.15879E-06	-0.115	0.083	0.166	46.788
MollicutesRF9	rs3932485	C	T	0.428	0.063	0.014	9.92857E-06	-0.076	0.055	0.168	35.286
MollicutesRF9	rs515984	T	C	0.174	-0.088	0.019	6.61366E-06	0.195	0.084	0.019	40.489
MollicutesRF9	rs638542	G	A	0.286	-0.071	0.016	5.17285E-06	-0.054	0.059	0.358	37.421

MollicutesRF9	rs74603314	T	C	0.046	0.231	0.049	2.27641E-06	-0.026	0.137	0.850	85.576
MollicutesRF9	rs7706512	G	A	0.504	0.066	0.014	2.27207E-06	0.000	0.054	1.000	39.703
MollicutesRF9	rs7801843	A	G	0.129	-0.087	0.019	9.46882E-06	-0.060	0.075	0.418	31.249
MollicutesRF9	rs7853673	G	A	0.514	-0.062	0.014	6.7325E-06	0.020	0.054	0.704	35.789
MollicutesRF9	rs949341	G	A	0.320	0.066	0.015	7.73282E-06	0.030	0.060	0.620	34.477
NB1n	rs11251024	G	A	0.330	0.104	0.021	6.62801E-07	-0.027	0.061	0.654	88.472
NB1n	rs11606187	A	G	0.107	-0.155	0.033	3.30586E-06	-0.014	0.077	0.853	84.355
NB1n	rs13385922	T	C	0.406	0.093	0.020	3.96829E-06	-0.023	0.056	0.685	76.712
NB1n	rs166849	G	A	0.390	0.091	0.020	7.73898E-06	0.015	0.054	0.784	72.688
NB1n	rs2172426	C	T	0.447	-0.102	0.020	3.17425E-07	-0.014	0.055	0.800	95.046
NB1n	rs267959	A	G	0.343	0.099	0.021	2.61993E-06	0.075	0.060	0.209	81.124
NB1n	rs4383094	T	C	0.096	0.149	0.032	4.28134E-06	0.050	0.077	0.519	71.382
NB1n	rs60775321	T	C	0.301	-0.096	0.021	7.1046E-06	-0.028	0.059	0.637	71.783
NB1n	rs72671304	T	C	0.070	0.172	0.037	3.79578E-06	0.169	0.102	0.096	70.797
NB1n	rs7911787	G	T	0.066	-0.223	0.047	3.38824E-06	0.307	0.152	0.044	112.554
NB1n	rs8126061	T	C	0.095	-0.159	0.035	7.36334E-06	0.010	0.081	0.902	80.505
NB1n	rs9542068	T	C	0.321	0.099	0.022	6.52339E-06	0.060	0.057	0.290	78.810
Pasteurellales	rs10965428	C	A	0.080	-0.120	0.026	4.29151E-06	-0.040	0.116	0.732	38.667
Pasteurellales	rs111582866	G	A	0.101	-0.114	0.026	7.06627E-06	-0.019	0.095	0.837	43.501
Pasteurellales	rs12050685	A	G	0.360	-0.067	0.015	9.18513E-06	-0.028	0.059	0.635	38.110
Pasteurellales	rs16970009	A	G	0.044	0.187	0.043	7.31531E-06	0.312	0.190	0.101	53.974
Pasteurellales	rs4822728	T	C	0.437	0.069	0.015	4.71847E-06	-0.027	0.053	0.614	42.506
Pasteurellales	rs6972479	A	G	0.243	-0.078	0.018	7.74817E-06	0.009	0.066	0.893	41.277
Pasteurellales	rs72756943	G	A	0.086	0.140	0.030	3.35218E-06	0.069	0.107	0.522	56.283
Pasteurellales	rs73139353	A	C	0.065	-0.223	0.048	8.71314E-06	-0.045	0.095	0.636	110.449
Pasteurellales	rs76022354	C	T	0.033	0.243	0.050	1.82858E-06	-0.288	0.123	0.019	68.900

Pasteurellales	rs78909003	T	C	0.050	-0.241	0.050	2.04893E-06	-0.125	0.118	0.288	101.298
Pasteurellales	rs9382510	C	T	0.246	-0.088	0.017	2.476E-07	0.065	0.061	0.292	52.985
Pasteurellales	rs9895850	T	C	0.050	-0.176	0.041	9.08251E-06	0.009	0.132	0.947	54.064
Pasteurellales	rs9938097	T	C	0.367	-0.071	0.016	8.22604E-06	0.137	0.055	0.013	43.088
Rhodospirillales	rs1035406	G	A	0.099	-0.115	0.025	4.07163E-06	0.069	0.083	0.406	43.315
Rhodospirillales	rs11591293	G	T	0.420	0.072	0.016	4.68529E-06	-0.059	0.054	0.274	46.774
Rhodospirillales	rs11630875	T	C	0.182	0.095	0.020	3.70379E-06	0.093	0.080	0.245	48.890
Rhodospirillales	rs13336560	C	T	0.456	-0.070	0.016	9.74686E-06	-0.014	0.055	0.795	44.096
Rhodospirillales	rs1549633	A	C	0.156	0.100	0.022	3.88024E-06	-0.023	0.084	0.782	48.125
Rhodospirillales	rs3730086	A	G	0.239	0.080	0.018	7.98252E-06	0.070	0.062	0.252	42.780
Rhodospirillales	rs3754624	C	T	0.162	0.094	0.020	2.68367E-06	0.003	0.070	0.971	44.221
Rhodospirillales	rs4278423	T	C	0.106	0.105	0.023	3.9804E-06	-0.116	0.110	0.294	38.685
Rhodospirillales	rs61933850	G	A	0.091	0.165	0.036	6.99876E-06	-0.015	0.079	0.846	82.218
Rhodospirillales	rs7001029	C	T	0.093	0.121	0.026	2.83071E-06	-0.099	0.093	0.288	45.375
Rhodospirillales	rs76784716	A	G	0.072	0.136	0.028	1.31382E-06	0.018	0.085	0.830	45.230
Rhodospirillales	rs77304857	C	A	0.112	-0.100	0.022	6.02031E-06	-0.079	0.068	0.242	36.447
Rhodospirillales	rs9813022	A	G	0.302	-0.083	0.016	3.07213E-07	-0.033	0.055	0.553	53.538
Selenomonadales	rs1135612	G	A	0.245	0.053	0.012	9.26307E-06	0.065	0.066	0.328	19.035
Selenomonadales	rs13086907	G	A	0.200	0.063	0.013	1.95488E-06	-0.011	0.065	0.868	22.957
Selenomonadales	rs1643968	T	C	0.280	-0.057	0.011	4.14596E-07	-0.061	0.056	0.271	23.673
Selenomonadales	rs1649999	A	G	0.097	0.075	0.017	7.5812E-06	-0.059	0.091	0.518	18.106
Selenomonadales	rs2834062	A	G	0.365	0.049	0.011	8.4392E-06	0.014	0.059	0.813	20.349
Selenomonadales	rs4463806	T	C	0.204	-0.054	0.013	7.80662E-06	0.023	0.068	0.738	17.627
Selenomonadales	rs4722181	T	G	0.476	0.050	0.011	2.00112E-06	0.003	0.054	0.955	23.019
Selenomonadales	rs60274479	T	C	0.223	-0.066	0.013	1.1623E-06	0.002	0.068	0.982	27.665
Selenomonadales	rs61249479	A	C	0.088	0.078	0.017	2.95471E-06	0.004	0.074	0.958	17.700

Selenomonadales	rs71405394	G	A	0.052	-0.114	0.024	2.16631E-06	0.121	0.106	0.253	23.471
Selenomonadales	rs73232831	G	A	0.064	-0.152	0.031	1.87452E-06	-0.003	0.144	0.981	50.490
Selenomonadales	rs9423647	G	A	0.515	0.048	0.011	6.06114E-06	-0.026	0.054	0.629	20.970
Verrucomicrobiales	rs111862613	T	C	0.166	0.091	0.020	3.73846E-06	0.024	0.072	0.741	41.865
Verrucomicrobiales	rs117107102	A	G	0.045	0.205	0.043	2.91821E-06	-0.052	0.128	0.686	65.849
Verrucomicrobiales	rs11729256	T	C	0.239	0.075	0.015	6.7309E-07	0.075	0.071	0.288	37.535
Verrucomicrobiales	rs12908520	G	A	0.456	0.062	0.013	2.17163E-06	0.032	0.054	0.558	34.922
Verrucomicrobiales	rs2602429	C	T	0.222	0.075	0.016	2.58297E-06	0.007	0.061	0.903	35.366
Verrucomicrobiales	rs4242783	G	A	0.287	0.069	0.015	2.63612E-06	-0.100	0.060	0.093	35.750
Verrucomicrobiales	rs4936098	A	G	0.340	0.065	0.014	1.12221E-06	-0.013	0.056	0.815	34.714
Verrucomicrobiales	rs61779207	G	A	0.178	-0.076	0.017	6.72285E-06	0.000	0.065	0.995	30.896
Verrucomicrobiales	rs74542928	T	C	0.079	0.112	0.024	1.63222E-06	0.209	0.125	0.093	33.438
Verrucomicrobiales	rs9349825	A	G	0.279	-0.070	0.015	2.53687E-06	0.046	0.068	0.502	36.666
Verrucomicrobiales	rs941682	G	A	0.311	-0.063	0.014	9.61003E-06	0.095	0.060	0.112	31.393
Victivallales	rs1002941	G	A	0.279	0.105	0.023	8.14836E-06	-0.007	0.062	0.906	81.795
Victivallales	rs11770843	C	T	0.259	0.109	0.023	1.90729E-06	-0.031	0.057	0.589	84.765
Victivallales	rs17114848	G	A	0.109	0.152	0.032	4.05865E-06	0.059	0.090	0.509	83.280
Victivallales	rs2031282	A	G	0.170	0.122	0.027	4.3826E-06	0.034	0.071	0.630	77.819
Victivallales	rs2825714	A	G	0.157	-0.137	0.029	1.72211E-06	0.050	0.071	0.485	92.161
Victivallales	rs62570196	C	T	0.069	-0.216	0.044	1.07926E-06	0.069	0.132	0.599	110.348
Victivallales	rs72640280	A	G	0.054	0.220	0.049	5.18035E-06	-0.040	0.117	0.734	90.822
Victivallales	rs77599476	A	G	0.066	0.230	0.048	1.86133E-06	-0.090	0.119	0.445	120.007
Actinobacteria	rs11766971	T	C	0.505	0.048	0.011	9.39927E-06	-0.035	0.054	0.515	20.829
Actinobacteria	rs12528285	C	T	0.094	0.081	0.018	5.68792E-06	0.081	0.086	0.344	20.567
Actinobacteria	rs13192624	T	C	0.278	-0.052	0.012	9.32576E-06	0.035	0.062	0.577	20.161
Actinobacteria	rs1397793	G	A	0.386	-0.052	0.011	3.73769E-06	-0.016	0.059	0.785	23.653

Actinobacteria	rs4429415	C	T	0.357	0.058	0.011	2.05146E-07	-0.061	0.054	0.255	28.549
Actinobacteria	rs55888705	A	G	0.387	0.053	0.011	1.31242E-06	-0.074	0.059	0.213	24.858
Actinobacteria	rs6496870	T	C	0.355	0.051	0.011	4.61743E-06	-0.003	0.056	0.956	21.933
Actinobacteria	rs6743026	T	C	0.174	0.059	0.013	9.8833E-06	-0.134	0.069	0.051	18.333
Actinobacteria	rs74037001	G	A	0.111	-0.082	0.017	6.7124E-07	-0.002	0.094	0.984	24.389
Actinobacteria	rs75211493	G	A	0.072	0.084	0.018	9.27186E-06	-0.003	0.103	0.978	17.251
Actinobacteria	rs7570971	A	C	0.510	0.087	0.011	1.41347E-14	-0.060	0.055	0.277	69.080
Actinobacteria	rs80124826	T	C	0.063	-0.124	0.028	8.75422E-06	0.371	0.164	0.023	33.254
Actinobacteria	rs857444	C	T	0.361	0.051	0.011	3.80349E-06	-0.129	0.056	0.020	21.795
Actinobacteria	rs9833771	T	C	0.558	0.049	0.011	4.0744E-06	0.072	0.054	0.182	21.754
Bacteroidetes	rs17343978	A	C	0.260	-0.056	0.012	7.2247E-06	-0.081	0.066	0.221	21.865
Bacteroidetes	rs2032750	T	C	0.451	-0.051	0.011	1.70835E-06	-0.113	0.054	0.035	23.732
Bacteroidetes	rs62531359	T	G	0.145	0.066	0.015	8.42316E-06	-0.023	0.070	0.745	19.706
Bacteroidetes	rs62575403	C	T	0.044	0.145	0.031	2.95953E-06	-0.158	0.133	0.235	32.471
Bacteroidetes	rs6586324	T	C	0.453	0.048	0.011	7.36878E-06	0.019	0.054	0.728	20.772
Bacteroidetes	rs72706335	T	C	0.066	-0.223	0.049	7.13111E-06	-0.005	0.174	0.977	112.678
Bacteroidetes	rs73512608	G	A	0.053	-0.123	0.024	2.53891E-07	-0.149	0.118	0.208	27.796
Bacteroidetes	rs73846128	A	G	0.170	-0.066	0.013	4.78296E-07	-0.037	0.076	0.630	22.863
Bacteroidetes	rs73975615	G	A	0.011	-0.207	0.044	1.20411E-06	0.065	0.316	0.837	16.985
Bacteroidetes	rs929878	C	T	0.245	-0.054	0.012	6.5112E-06	0.045	0.065	0.495	19.777
Cyanobacteria	rs12555298	G	A	0.175	0.097	0.022	8.08628E-06	0.143	0.071	0.046	50.405
Cyanobacteria	rs2585223	T	C	0.153	0.111	0.025	8.85734E-06	0.028	0.082	0.738	59.167
Cyanobacteria	rs584122	C	T	0.067	-0.152	0.033	4.22775E-06	0.029	0.114	0.799	52.670
Cyanobacteria	rs61972390	T	C	0.168	0.107	0.024	9.11371E-06	0.059	0.078	0.448	59.263
Cyanobacteria	rs7148504	G	T	0.305	0.080	0.018	6.62391E-06	0.042	0.055	0.438	49.960
Cyanobacteria	rs76531781	T	C	0.050	-0.232	0.049	2.87336E-06	0.078	0.135	0.563	93.603

Cyanobacteria	rs789068	G	A	0.172	-0.111	0.021	1.57171E-07	0.027	0.077	0.728	65.033
Cyanobacteria	rs9864379	T	C	0.113	-0.139	0.027	2.03334E-07	-0.143	0.076	0.060	71.398
Euryarchaeota	rs10202904	T	G	0.467	-0.116	0.023	6.1888E-07	-0.046	0.055	0.401	123.645
Euryarchaeota	rs11022995	G	A	0.453	-0.104	0.023	7.73001E-06	0.050	0.054	0.350	98.321
Euryarchaeota	rs34928225	T	C	0.076	0.200	0.043	4.33093E-06	0.055	0.092	0.553	102.736
Euryarchaeota	rs45498998	G	A	0.185	-0.132	0.029	5.32453E-06	0.060	0.074	0.418	96.646
Euryarchaeota	rs6064552	T	C	0.198	-0.124	0.028	9.3378E-06	0.009	0.069	0.891	89.355
Euryarchaeota	rs6508769	T	C	0.165	0.151	0.034	8.12354E-06	-0.018	0.075	0.808	115.216
Euryarchaeota	rs7015093	G	A	0.263	-0.118	0.026	7.1987E-06	0.074	0.061	0.224	99.809
Euryarchaeota	rs76029318	T	C	0.065	0.215	0.044	1.04622E-06	-0.160	0.109	0.144	102.891
Euryarchaeota	rs7635189	G	A	0.331	0.120	0.026	4.635E-06	0.088	0.060	0.143	117.667
Euryarchaeota	rs77658038	A	C	0.117	-0.160	0.034	4.7542E-06	-0.033	0.066	0.618	97.926
Euryarchaeota	rs894996	C	A	0.072	0.204	0.044	5.11931E-06	-0.096	0.105	0.360	101.598
Firmicutes	rs112334273	G	A	0.246	0.063	0.013	9.26452E-07	0.012	0.060	0.845	26.785
Firmicutes	rs2273429	A	G	0.107	-0.070	0.015	9.26239E-06	-0.137	0.087	0.118	17.349
Firmicutes	rs2332027	G	A	0.500	-0.048	0.010	4.04546E-06	-0.020	0.055	0.716	21.385
Firmicutes	rs2547978	A	G	0.484	0.047	0.011	8.56845E-06	-0.046	0.055	0.410	19.896
Firmicutes	rs3792064	G	A	0.080	0.090	0.018	6.75498E-07	-0.165	0.120	0.169	21.638
Firmicutes	rs3852931	C	T	0.466	-0.048	0.010	4.52594E-06	0.033	0.056	0.552	21.237
Firmicutes	rs4750583	A	G	0.182	0.062	0.014	5.78565E-06	0.051	0.069	0.456	20.734
Firmicutes	rs56199908	T	C	0.054	-0.186	0.041	8.66656E-06	-0.005	0.118	0.964	64.841
Firmicutes	rs6814436	T	C	0.132	0.068	0.015	6.80463E-06	-0.003	0.077	0.967	19.488
Firmicutes	rs6815608	T	C	0.085	0.094	0.021	7.23859E-06	0.012	0.075	0.873	24.878
Firmicutes	rs7247191	T	C	0.138	-0.071	0.016	4.73101E-06	0.040	0.090	0.658	22.235
Firmicutes	rs72738886	T	C	0.078	0.086	0.019	7.67884E-06	0.098	0.101	0.334	19.622
Firmicutes	rs72771021	C	T	0.050	-0.141	0.031	5.12438E-06	0.104	0.107	0.330	34.678

Firmicutes	rs8085381	G	A	0.147	0.065	0.015	8.67253E-06	-0.113	0.069	0.103	19.300
Lentisphaerae	rs1002941	G	A	0.279	0.108	0.023	4.3088E-06	-0.007	0.062	0.906	85.971
Lentisphaerae	rs11770843	C	T	0.259	0.112	0.023	1.13813E-06	-0.031	0.057	0.589	88.645
Lentisphaerae	rs17114848	G	A	0.109	0.149	0.032	6.76994E-06	0.059	0.090	0.509	79.584
Lentisphaerae	rs2031282	A	G	0.170	0.120	0.027	5.86463E-06	0.034	0.071	0.630	75.341
Lentisphaerae	rs2825714	A	G	0.157	-0.138	0.029	1.49772E-06	0.050	0.071	0.485	93.265
Lentisphaerae	rs60995569	T	G	0.090	-0.161	0.034	9.19393E-06	-0.009	0.086	0.918	77.431
Lentisphaerae	rs62570196	C	T	0.069	-0.217	0.044	9.64474E-07	0.069	0.132	0.599	111.225
Lentisphaerae	rs72640280	A	G	0.054	0.220	0.049	5.19196E-06	-0.040	0.117	0.734	90.804
Lentisphaerae	rs77599476	A	G	0.066	0.230	0.048	1.90263E-06	-0.090	0.119	0.445	119.682
Proteobacteria	rs10750258	A	C	0.394	-0.049	0.011	8.71899E-06	0.016	0.055	0.778	21.149
Proteobacteria	rs11126162	T	C	0.089	-0.077	0.019	9.25979E-06	-0.005	0.097	0.958	17.590
Proteobacteria	rs11715072	G	A	0.289	-0.052	0.012	6.90409E-06	-0.102	0.058	0.081	20.357
Proteobacteria	rs12150865	C	T	0.475	0.051	0.011	1.54085E-06	0.015	0.055	0.784	23.972
Proteobacteria	rs12467198	C	T	0.342	0.050	0.011	6.30669E-06	0.025	0.054	0.646	20.511
Proteobacteria	rs2347697	G	T	0.318	0.050	0.011	4.26914E-06	-0.026	0.058	0.656	20.093
Proteobacteria	rs2532663	G	A	0.063	-0.126	0.026	7.46812E-07	0.141	0.089	0.113	34.003
Proteobacteria	rs3890996	T	G	0.539	-0.047	0.011	6.94663E-06	0.063	0.054	0.239	20.553
Proteobacteria	rs4340090	C	T	0.125	-0.067	0.015	9.99003E-06	-0.065	0.083	0.433	17.951
Proteobacteria	rs6707783	C	T	0.115	0.085	0.019	8.08593E-06	0.110	0.093	0.237	27.044
Proteobacteria	rs72771021	C	T	0.050	0.142	0.031	7.18097E-06	0.104	0.107	0.330	34.860
Proteobacteria	rs922773	C	T	0.140	-0.080	0.016	3.67958E-07	-0.048	0.089	0.586	28.607
Tenericutes	rs10108398	G	A	0.291	0.077	0.015	1.09E-06	-0.001	0.060	0.993	44.901
Tenericutes	rs11890098	A	G	0.256	0.074	0.015	9.56571E-07	-0.057	0.060	0.339	38.739
Tenericutes	rs12566890	T	G	0.095	-0.101	0.023	3.65095E-06	-0.105	0.080	0.188	32.439
Tenericutes	rs17214486	C	A	0.402	0.061	0.014	6.60866E-06	-0.105	0.057	0.068	32.844

Tenericutes	rs2464826	A	C	0.126	0.094	0.021	8.39505E-06	0.032	0.085	0.705	36.130
Tenericutes	rs28537087	G	A	0.164	0.082	0.019	8.07399E-06	-0.071	0.062	0.258	33.946
Tenericutes	rs3768491	A	G	0.286	-0.068	0.015	4.23352E-06	-0.050	0.059	0.397	34.826
Tenericutes	rs4885016	T	C	0.157	-0.082	0.018	7.26986E-06	0.039	0.079	0.626	32.683
Tenericutes	rs6043847	T	C	0.070	-0.115	0.025	4.55476E-06	-0.102	0.114	0.373	31.429
Tenericutes	rs72901605	T	C	0.186	-0.084	0.018	3.2579E-06	0.029	0.084	0.728	39.422
Tenericutes	rs74603314	T	C	0.046	0.222	0.046	1.55689E-06	-0.026	0.137	0.850	78.912
Tenericutes	rs78169027	A	G	0.108	-0.108	0.024	5.8761E-06	0.067	0.113	0.552	41.623
Verrucomicrobia	rs11252894	A	C	0.198	0.078	0.016	1.10556E-06	-0.110	0.063	0.080	35.845
Verrucomicrobia	rs117107102	A	G	0.045	0.204	0.043	2.67935E-06	-0.052	0.128	0.686	65.591
Verrucomicrobia	rs11729256	T	C	0.239	0.070	0.015	2.23402E-06	0.075	0.071	0.288	32.425
Verrucomicrobia	rs12512971	A	C	0.049	0.171	0.040	9.81381E-06	0.122	0.101	0.225	49.853
Verrucomicrobia	rs12908520	G	A	0.456	0.059	0.013	3.40448E-06	0.032	0.054	0.558	32.260
Verrucomicrobia	rs2602429	C	T	0.222	0.076	0.015	8.7104E-07	0.007	0.061	0.903	37.028
Verrucomicrobia	rs3995795	C	T	0.318	0.061	0.014	9.72209E-06	-0.032	0.055	0.562	29.739
Verrucomicrobia	rs45598138	C	A	0.052	-0.144	0.031	2.19127E-06	-0.099	0.169	0.557	37.311
Verrucomicrobia	rs61779207	G	A	0.178	-0.076	0.016	5.27971E-06	0.000	0.065	0.995	30.647
Verrucomicrobia	rs74542928	T	C	0.079	0.116	0.023	4.08375E-07	0.209	0.125	0.093	35.766
Verrucomicrobia	rs76430504	T	C	0.056	-0.118	0.025	3.4956E-06	-0.127	0.123	0.300	26.686
Verrucomicrobia	rs9349825	A	G	0.279	-0.066	0.014	6.27486E-06	0.046	0.068	0.502	32.239

MR, Mendelian randomization; ITP, Immune thrombocytopenia; SNP, single nucleotide polymorphism; MAF, minor allele frequency; SE, standard error.

Table S2 Full result of MR estimates for the association between gut microbiota and ITP.

Bacterial taxa (exposure)	MR method	No.of SNP	OR	OR_95%CI(L)	OR_95%CI(U)	*P* value
Actinobacteria	Inverse variance weighted	14	0.658	0.408	1.060	0.085
Actinobacteria	Weighted median	14	0.522	0.272	1.002	0.051
Actinobacteria	MR Egger	14	1.301	0.338	5.001	0.708
Alphaproteobacteria	Inverse variance weighted	6	0.966	0.529	1.765	0.911
Alphaproteobacteria	MR Egger	6	0.798	0.096	6.601	0.844
Alphaproteobacteria	Weighted median	6	0.972	0.454	2.080	0.942
Bacilli	MR Egger	18	0.303	0.084	1.091	0.086
Bacilli	Inverse variance weighted	18	1.060	0.645	1.741	0.818
Bacilli	Weighted median	18	1.006	0.508	1.993	0.987
Bacteroidia	Weighted median	12	1.297	0.559	3.008	0.545
Bacteroidia	Inverse variance weighted	12	1.538	0.835	2.835	0.167
Bacteroidia	MR Egger	12	0.679	0.162	2.837	0.607
Betaproteobacteria	Inverse variance weighted	9	1.080	0.424	2.751	0.871
Betaproteobacteria	Weighted median	9	0.906	0.359	2.284	0.834
Betaproteobacteria	MR Egger	9	0.411	0.019	8.694	0.586
Clostridia	MR Egger	11	4.434	0.256	76.844	0.333

Clostridia	Weighted median	11	1.195	0.521	2.741	0.675
Clostridia	Inverse variance weighted	11	1.162	0.613	2.204	0.646
Coriobacteriia	MR Egger	14	0.507	0.052	4.961	0.570
Coriobacteriia	Inverse variance weighted	14	0.996	0.570	1.740	0.988
Coriobacteriia	Weighted median	14	0.792	0.374	1.677	0.542
Deltaproteobacteria	MR Egger	12	0.054	0.003	0.960	0.075
Deltaproteobacteria	Weighted median	12	0.950	0.395	2.283	0.909
Deltaproteobacteria	Inverse variance weighted	12	1.246	0.598	2.596	0.558
Erysipelotrichia	Inverse variance weighted	12	0.610	0.280	1.331	0.214
Erysipelotrichia	Weighted median	12	0.672	0.284	1.591	0.366
Erysipelotrichia	MR Egger	12	0.457	0.014	15.261	0.671
Gammaproteobacteria	Inverse variance weighted	7	0.718	0.255	2.020	0.530
Gammaproteobacteria	MR Egger	7	0.869	0.023	33.342	0.943
Gammaproteobacteria	Weighted median	7	0.782	0.269	2.273	0.652
Lentisphaeria	MR Egger	8	0.667	0.170	2.613	0.582
Lentisphaeria	Weighted median	8	0.778	0.485	1.247	0.297
Lentisphaeria	Inverse variance weighted	8	0.871	0.593	1.278	0.479
Melainabacteria	MR Egger	10	1.776	0.549	5.750	0.366
Melainabacteria	Weighted median	10	1.180	0.710	1.963	0.523
Melainabacteria	Inverse variance weighted	10	1.279	0.868	1.884	0.214
Methanobacteria	Weighted median	9	0.594	0.379	0.930	0.023
Methanobacteria	MR Egger	9	0.398	0.107	1.476	0.211
Methanobacteria	Inverse variance weighted	9	0.631	0.452	0.881	0.007
Mollicutes	Weighted median	12	0.865	0.458	1.632	0.654
Mollicutes	Inverse variance weighted	12	0.867	0.538	1.399	0.559
Mollicutes	MR Egger	12	1.897	0.375	9.605	0.457

Negativicutes	MR Egger	12	0.428	0.054	3.368	0.439
Negativicutes	Inverse variance weighted	12	0.961	0.527	1.755	0.898
Negativicutes	Weighted median	12	1.005	0.478	2.110	0.990
Verrucomicrobiae	Weighted median	11	0.936	0.484	1.808	0.844
Verrucomicrobiae	Inverse variance weighted	11	0.915	0.548	1.527	0.734
Verrucomicrobiae	MR Egger	11	1.565	0.261	9.394	0.636
Acidaminococcaceae	Weighted median	7	0.859	0.397	1.859	0.699
Acidaminococcaceae	Inverse variance weighted	7	0.964	0.530	1.755	0.904
Acidaminococcaceae	MR Egger	7	0.678	0.096	4.801	0.713
Actinomycetaceae	Weighted median	4	0.988	0.438	2.227	0.977
Actinomycetaceae	MR Egger	4	0.317	0.079	1.270	0.246
Actinomycetaceae	Inverse variance weighted	4	1.238	0.503	3.048	0.643
Alcaligenaceae	Weighted median	11	1.996	0.849	4.694	0.113
Alcaligenaceae	MR Egger	11	1.971	0.103	37.790	0.663
Alcaligenaceae	Inverse variance weighted	11	2.396	1.276	4.500	0.007
Bacteroidaceae	Inverse variance weighted	8	0.720	0.355	1.461	0.364
Bacteroidaceae	Weighted median	8	0.632	0.249	1.602	0.334
Bacteroidaceae	MR Egger	8	0.443	0.011	18.650	0.684
BacteroidalesS24	MR Egger	8	1.503	0.233	9.713	0.683
BacteroidalesS24	Weighted median	8	0.489	0.268	0.891	0.019
BacteroidalesS24	Inverse variance weighted	8	0.520	0.325	0.830	0.006
Bifidobacteriaceae	MR Egger	11	1.447	0.209	10.023	0.717
Bifidobacteriaceae	Weighted median	11	0.764	0.379	1.543	0.453
Bifidobacteriaceae	Inverse variance weighted	11	0.821	0.463	1.457	0.501
Christensenellaceae	MR Egger	11	0.997	0.393	2.528	0.996
Christensenellaceae	Inverse variance weighted	11	1.331	0.825	2.149	0.242

Christensenellaceae	Weighted median	11	1.229	0.650	2.323	0.526
Clostridiaceae1	MR Egger	10	0.361	0.068	1.925	0.267
Clostridiaceae1	Inverse variance weighted	10	1.052	0.591	1.870	0.864
Clostridiaceae1	Weighted median	10	1.563	0.701	3.484	0.275
ClostridialesvadinBB60group	Inverse variance weighted	15	0.937	0.626	1.402	0.752
ClostridialesvadinBB60group	Weighted median	15	0.829	0.467	1.472	0.522
ClostridialesvadinBB60group	MR Egger	15	1.113	0.369	3.358	0.853
Coriobacteriaceae	MR Egger	14	0.507	0.052	4.961	0.570
Coriobacteriaceae	Inverse variance weighted	14	0.996	0.570	1.740	0.988
Coriobacteriaceae	Weighted median	14	0.792	0.372	1.687	0.546
Defluviitaleaceae	Weighted median	11	1.662	0.901	3.067	0.104
Defluviitaleaceae	Inverse variance weighted	11	1.033	0.661	1.614	0.886
Defluviitaleaceae	MR Egger	11	1.680	0.352	8.020	0.532
Desulfovibrionaceae	MR Egger	9	0.069	0.004	1.220	0.111
Desulfovibrionaceae	Inverse variance weighted	9	1.097	0.488	2.465	0.823
Desulfovibrionaceae	Weighted median	9	0.832	0.330	2.099	0.696
Enterobacteriaceae	MR Egger	7	38.841	0.409	3693.030	0.176
Enterobacteriaceae	Weighted median	7	0.690	0.240	1.984	0.491
Enterobacteriaceae	Inverse variance weighted	7	1.436	0.628	3.284	0.391
Erysipelotrichaceae	Inverse variance weighted	12	0.610	0.280	1.331	0.214
Erysipelotrichaceae	Weighted median	12	0.672	0.268	1.682	0.396
Erysipelotrichaceae	MR Egger	12	0.457	0.014	15.261	0.671
FamilyXI	Inverse variance weighted	6	1.263	0.887	1.798	0.195
FamilyXI	MR Egger	6	1.512	0.172	13.322	0.729
FamilyXI	Weighted median	6	1.369	0.883	2.123	0.160
FamilyXIII	Inverse variance weighted	7	0.886	0.383	2.048	0.778

FamilyXIII	Weighted median	7	0.608	0.202	1.829	0.376
FamilyXIII	MR Egger	7	0.201	0.009	4.638	0.362
Lachnospiraceae	MR Egger	16	0.278	0.036	2.129	0.238
Lachnospiraceae	Weighted median	16	0.723	0.318	1.643	0.438
Lachnospiraceae	Inverse variance weighted	16	0.545	0.307	0.967	0.038
Lactobacillaceae	Weighted median	8	1.057	0.568	1.968	0.861
Lactobacillaceae	MR Egger	8	0.690	0.209	2.274	0.564
Lactobacillaceae	Inverse variance weighted	8	1.072	0.674	1.705	0.769
Methanobacteriaceae	MR Egger	9	0.398	0.107	1.476	0.211
Methanobacteriaceae	Inverse variance weighted	9	0.631	0.452	0.881	0.007
Methanobacteriaceae	Weighted median	9	0.594	0.373	0.946	0.028
Oxalobacteraceae	Inverse variance weighted	13	1.386	0.969	1.982	0.074
Oxalobacteraceae	Weighted median	13	1.072	0.692	1.660	0.757
Oxalobacteraceae	MR Egger	13	1.845	0.456	7.466	0.409
Pasteurellaceae	Weighted median	13	1.203	0.699	2.069	0.505
Pasteurellaceae	MR Egger	13	1.378	0.535	3.550	0.520
Pasteurellaceae	Inverse variance weighted	13	0.888	0.570	1.382	0.599
Peptococcaceae	Weighted median	9	1.239	0.643	2.387	0.521
Peptococcaceae	Inverse variance weighted	9	1.233	0.747	2.034	0.413
Peptococcaceae	MR Egger	9	0.872	0.219	3.476	0.852
Peptostreptococcaceae	Weighted median	13	1.258	0.654	2.419	0.491
Peptostreptococcaceae	MR Egger	13	0.552	0.187	1.627	0.304
Peptostreptococcaceae	Inverse variance weighted	13	0.895	0.557	1.438	0.646
Porphyromonadaceae	Weighted median	9	0.539	0.186	1.559	0.254
Porphyromonadaceae	Inverse variance weighted	9	0.968	0.454	2.063	0.933
Porphyromonadaceae	MR Egger	9	1.339	0.037	48.890	0.878

Prevotellaceae	Inverse variance weighted	16	1.286	0.803	2.059	0.295
Prevotellaceae	Weighted median	16	1.701	0.869	3.329	0.121
Prevotellaceae	MR Egger	16	1.297	0.233	7.214	0.771
Rhodospirillaceae	Inverse variance weighted	14	0.799	0.547	1.166	0.244
Rhodospirillaceae	MR Egger	14	0.769	0.163	3.622	0.746
Rhodospirillaceae	Weighted median	14	0.900	0.557	1.453	0.667
Rikenellaceae	Weighted median	16	1.114	0.542	2.293	0.769
Rikenellaceae	MR Egger	16	0.559	0.120	2.615	0.473
Rikenellaceae	Inverse variance weighted	16	1.160	0.701	1.919	0.563
Ruminococcaceae	Weighted median	10	1.017	0.456	2.266	0.967
Ruminococcaceae	Inverse variance weighted	10	1.096	0.613	1.958	0.757
Ruminococcaceae	MR Egger	10	0.628	0.179	2.197	0.487
Streptococcaceae	Weighted median	11	0.635	0.280	1.441	0.278
Streptococcaceae	MR Egger	11	0.892	0.072	11.038	0.931
Streptococcaceae	Inverse variance weighted	11	0.714	0.388	1.315	0.280
unknownfamily	Inverse variance weighted	9	1.191	0.793	1.789	0.401
unknownfamily	MR Egger	9	2.179	0.646	7.343	0.249
unknownfamily	Weighted median	9	1.176	0.691	2.002	0.551
unknownfamily	MR Egger	12	2.009	0.338	11.950	0.461
unknownfamily	Inverse variance weighted	12	0.897	0.501	1.605	0.714
unknownfamily	Weighted median	12	0.973	0.487	1.946	0.938
unknownfamily	Inverse variance weighted	12	1.157	0.839	1.597	0.374
unknownfamily	MR Egger	12	0.715	0.193	2.642	0.625
unknownfamily	Weighted median	12	1.154	0.757	1.759	0.507
Veillonellaceae	MR Egger	17	0.795	0.303	2.086	0.648
Veillonellaceae	Inverse variance weighted	17	1.156	0.709	1.882	0.561

Veillonellaceae	Weighted median	17	1.091	0.589	2.023	0.782
Verrucomicrobiaceae	Weighted median	11	0.936	0.480	1.826	0.846
Verrucomicrobiaceae	Inverse variance weighted	11	0.915	0.548	1.528	0.735
Verrucomicrobiaceae	MR Egger	11	1.568	0.261	9.406	0.635
Victivallaceae	MR Egger	11	1.144	0.304	4.307	0.847
Victivallaceae	Weighted median	11	0.945	0.662	1.349	0.755
Victivallaceae	Inverse variance weighted	11	0.969	0.728	1.291	0.832
Actinomyces	Inverse variance weighted	7	0.839	0.455	1.548	0.574
Actinomyces	Weighted median	7	0.760	0.389	1.483	0.421
Actinomyces	MR Egger	7	0.603	0.120	3.024	0.566
Adlercreutzia	Inverse variance weighted	7	1.826	1.010	3.299	0.046
Adlercreutzia	MR Egger	7	0.229	0.022	2.436	0.276
Adlercreutzia	Weighted median	7	1.753	0.809	3.800	0.155
Akkermansia	Inverse variance weighted	11	0.916	0.549	1.529	0.737
Akkermansia	MR Egger	11	1.583	0.264	9.478	0.627
Akkermansia	Weighted median	11	0.938	0.463	1.903	0.860
Alistipes	Weighted median	12	0.906	0.385	2.136	0.822
Alistipes	Inverse variance weighted	12	0.763	0.396	1.468	0.418
Alistipes	MR Egger	12	0.579	0.025	13.601	0.741
Allisonella	MR Egger	8	4.414	0.300	64.996	0.321
Allisonella	Inverse variance weighted	8	0.581	0.377	0.895	0.014
Allisonella	Weighted median	8	0.584	0.378	0.902	0.015
Alloprevotella	MR Egger	5	4.398	0.145	133.007	0.457
Alloprevotella	Weighted median	5	1.254	0.772	2.037	0.361
Alloprevotella	Inverse variance weighted	5	1.316	0.889	1.949	0.170
Anaerofilum	MR Egger	10	1.588	0.229	11.012	0.652

Anaerofilum	Weighted median	10	1.158	0.746	1.797	0.513
Anaerofilum	Inverse variance weighted	10	1.233	0.863	1.761	0.250
Anaerostipes	MR Egger	13	7.335	0.886	60.693	0.092
Anaerostipes	Inverse variance weighted	13	1.294	0.717	2.334	0.392
Anaerostipes	Weighted median	13	1.224	0.559	2.678	0.613
Anaerotruncus	Weighted median	12	1.829	0.729	4.589	0.198
Anaerotruncus	MR Egger	12	4.469	0.338	59.002	0.282
Anaerotruncus	Inverse variance weighted	12	1.198	0.583	2.460	0.623
Bacteroides	MR Egger	8	0.443	0.011	18.650	0.684
Bacteroides	Weighted median	8	0.632	0.255	1.566	0.322
Bacteroides	Inverse variance weighted	8	0.720	0.355	1.461	0.364
Barnesiella	MR Egger	12	0.957	0.102	8.930	0.970
Barnesiella	Inverse variance weighted	12	1.159	0.671	2.000	0.597
Barnesiella	Weighted median	12	0.929	0.461	1.873	0.837
Bifidobacterium	Inverse variance weighted	11	0.606	0.337	1.089	0.094
Bifidobacterium	Weighted median	11	0.579	0.274	1.221	0.151
Bifidobacterium	MR Egger	11	2.113	0.319	14.005	0.458
Bilophila	Weighted median	13	0.947	0.471	1.903	0.878
Bilophila	MR Egger	13	1.193	0.091	15.569	0.895
Bilophila	Inverse variance weighted	13	1.116	0.662	1.882	0.680
Blautia	Weighted median	12	1.770	0.771	4.063	0.178
Blautia	MR Egger	12	0.652	0.136	3.123	0.604
Blautia	Inverse variance weighted	12	1.439	0.780	2.654	0.244
Butyricicoccus	Inverse variance weighted	8	0.767	0.405	1.450	0.414
Butyricicoccus	MR Egger	8	1.028	0.295	3.583	0.967
Butyricicoccus	Weighted median	8	0.900	0.397	2.042	0.801

Butyricimonas	Inverse variance weighted	13	0.930	0.574	1.505	0.766
Butyricimonas	Weighted median	13	1.067	0.551	2.067	0.848
Butyricimonas	MR Egger	13	0.769	0.137	4.309	0.771
Butyrivibrio	MR Egger	15	1.971	0.715	5.429	0.212
Butyrivibrio	Weighted median	15	0.971	0.711	1.327	0.856
Butyrivibrio	Inverse variance weighted	15	0.974	0.769	1.232	0.823
CandidatusSoleaferrea	MR Egger	9	0.079	0.000	16.411	0.382
CandidatusSoleaferrea	Weighted median	9	1.547	0.845	2.833	0.157
CandidatusSoleaferrea	Inverse variance weighted	9	1.465	0.863	2.486	0.157
Catenibacterium	MR Egger	4	0.096	0.000	32.181	0.512
Catenibacterium	Weighted median	4	0.806	0.454	1.431	0.462
Catenibacterium	Inverse variance weighted	4	0.744	0.468	1.184	0.213
ChristensenellaceaeR	Inverse variance weighted	8	1.711	0.807	3.628	0.162
ChristensenellaceaeR	Weighted median	8	1.815	0.639	5.156	0.263
ChristensenellaceaeR	MR Egger	8	4.761	0.337	67.325	0.292
Clostridiuminnocuumgroup	MR Egger	8	2.832	0.497	16.148	0.286
Clostridiuminnocuumgroup	Inverse variance weighted	8	0.947	0.670	1.340	0.760
Clostridiuminnocuumgroup	Weighted median	8	0.903	0.553	1.475	0.684
Clostridiumsensustricto1	Inverse variance weighted	6	1.224	0.655	2.288	0.526
Clostridiumsensustricto1	Weighted median	6	1.613	0.730	3.563	0.237
Clostridiumsensustricto1	MR Egger	6	0.485	0.089	2.644	0.450
Collinsella	Inverse variance weighted	9	1.268	0.642	2.501	0.494
Collinsella	MR Egger	9	0.259	0.020	3.277	0.331
Collinsella	Weighted median	9	1.198	0.453	3.166	0.716
Coprobacter	Inverse variance weighted	11	0.793	0.536	1.174	0.247
Coprobacter	MR Egger	11	0.444	0.096	2.059	0.327

Coprobacter	Weighted median	11	0.700	0.413	1.187	0.186
Coprococcus1	Weighted median	12	0.854	0.417	1.749	0.667
Coprococcus1	Inverse variance weighted	12	0.815	0.476	1.397	0.458
Coprococcus1	MR Egger	12	1.438	0.372	5.565	0.610
Coprococcus2	Inverse variance weighted	8	0.522	0.283	0.963	0.037
Coprococcus2	MR Egger	8	0.165	0.001	20.734	0.492
Coprococcus2	Weighted median	8	0.515	0.227	1.164	0.111
Coprococcus3	Inverse variance weighted	8	1.745	0.833	3.656	0.140
Coprococcus3	MR Egger	8	1.385	0.017	113.006	0.889
Coprococcus3	Weighted median	8	1.341	0.486	3.703	0.571
DefluviitaleaceaeUCG011	MR Egger	9	3.786	0.627	22.875	0.190
DefluviitaleaceaeUCG011	Inverse variance weighted	9	1.189	0.712	1.983	0.509
DefluviitaleaceaeUCG011	Weighted median	9	1.702	0.872	3.321	0.119
Desulfovibrio	Weighted median	10	0.836	0.434	1.614	0.594
Desulfovibrio	Inverse variance weighted	10	0.761	0.467	1.239	0.272
Desulfovibrio	MR Egger	10	1.688	0.398	7.159	0.497
Dialister	Inverse variance weighted	11	1.155	0.684	1.951	0.590
Dialister	MR Egger	11	4.624	0.541	39.552	0.196
Dialister	Weighted median	11	1.142	0.544	2.394	0.726
Dorea	Inverse variance weighted	10	0.795	0.325	1.945	0.616
Dorea	MR Egger	10	1.409	0.103	19.209	0.804
Dorea	Weighted median	10	0.852	0.300	2.416	0.763
Eggerthella	Weighted median	9	1.313	0.762	2.263	0.326
Eggerthella	Inverse variance weighted	9	1.073	0.696	1.656	0.749
Eggerthella	MR Egger	9	1.726	0.221	13.506	0.619
Eisenbergiella	Inverse variance weighted	11	0.951	0.664	1.362	0.786

Eisenbergiella	MR Egger	11	0.358	0.024	5.396	0.477
Eisenbergiella	Weighted median	11	1.015	0.624	1.651	0.951
Enterorhabdus	Weighted median	6	0.929	0.462	1.868	0.837
Enterorhabdus	Inverse variance weighted	6	0.907	0.513	1.604	0.736
Enterorhabdus	MR Egger	6	0.576	0.128	2.596	0.513
Erysipelatoclostridium	Weighted median	15	1.355	0.759	2.420	0.304
Erysipelatoclostridium	MR Egger	15	0.935	0.128	6.837	0.948
Erysipelatoclostridium	Inverse variance weighted	15	1.228	0.752	2.005	0.411
ErysipelotrichaceaeUCG003	Weighted median	16	1.403	0.740	2.661	0.299
ErysipelotrichaceaeUCG003	Inverse variance weighted	16	1.149	0.730	1.808	0.548
ErysipelotrichaceaeUCG003	MR Egger	16	1.051	0.304	3.634	0.938
Escherichia	MR Egger	10	0.236	0.029	1.943	0.217
Escherichia	Inverse variance weighted	10	1.170	0.563	2.432	0.674
Escherichia	Weighted median	10	1.534	0.683	3.446	0.300
Eubacteriumbrachygroup	Inverse variance weighted	10	1.334	0.947	1.879	0.100
Eubacteriumbrachygroup	Weighted median	10	1.204	0.755	1.921	0.436
Eubacteriumbrachygroup	MR Egger	10	2.213	0.537	9.119	0.303
Eubacteriumcoprostanoligenesgroup	MR Egger	12	0.338	0.033	3.503	0.384
Eubacteriumcoprostanoligenesgroup	Weighted median	12	1.629	0.697	3.807	0.260
Eubacteriumcoprostanoligenesgroup	Inverse variance weighted	12	1.586	0.859	2.929	0.141
Eubacteriumeligensgroup	Weighted median	6	1.345	0.503	3.601	0.555
Eubacteriumeligensgroup	MR Egger	6	1.903	0.106	34.068	0.685
Eubacteriumeligensgroup	Inverse variance weighted	6	1.133	0.524	2.450	0.751
Eubacteriumfissicatenagroup	MR Egger	9	1.002	0.167	6.000	0.998
Eubacteriumfissicatenagroup	Weighted median	9	1.047	0.677	1.620	0.836

Eubacteriumfissicatenagroup	Inverse variance weighted	9	0.959	0.680	1.355	0.814
Eubacteriumhalliigroup	Weighted median	14	0.618	0.309	1.233	0.172
Eubacteriumhalliigroup	Inverse variance weighted	14	0.650	0.402	1.049	0.078
Eubacteriumhalliigroup	MR Egger	14	0.811	0.298	2.207	0.689
Eubacteriumnodatumgroup	MR Egger	11	1.178	0.337	4.119	0.803
Eubacteriumnodatumgroup	Inverse variance weighted	11	1.039	0.785	1.377	0.787
Eubacteriumnodatumgroup	Weighted median	11	1.122	0.763	1.650	0.560
Eubacteriumoxidoreducensgroup	Weighted median	5	0.803	0.390	1.655	0.553
Eubacteriumoxidoreducensgroup	Inverse variance weighted	5	0.872	0.466	1.633	0.669
Eubacteriumoxidoreducensgroup	MR Egger	5	0.628	0.044	8.933	0.754
Eubacteriumrectalegroup	MR Egger	8	0.315	0.023	4.261	0.418
Eubacteriumrectalegroup	Inverse variance weighted	8	0.965	0.473	1.969	0.922
Eubacteriumrectalegroup	Weighted median	8	1.149	0.432	3.054	0.780
Eubacteriumruminantiumgroup	MR Egger	18	1.004	0.352	2.860	0.995
Eubacteriumruminantiumgroup	Inverse variance weighted	18	0.724	0.529	0.990	0.043
Eubacteriumruminantiumgroup	Weighted median	18	0.825	0.553	1.232	0.347
Eubacteriumventriosumgroup	MR Egger	14	0.702	0.074	6.632	0.763
Eubacteriumventriosumgroup	Weighted median	14	0.851	0.433	1.672	0.640
Eubacteriumventriosumgroup	Inverse variance weighted	14	0.980	0.581	1.653	0.939
Eubacteriumxylanophilumgroup	Weighted median	9	1.134	0.530	2.426	0.747
Eubacteriumxylanophilumgroup	Inverse variance weighted	9	0.929	0.534	1.614	0.793
Eubacteriumxylanophilumgroup	MR Egger	9	2.513	0.481	13.141	0.311
Faecalibacterium	Weighted median	10	1.215	0.626	2.356	0.565
Faecalibacterium	Inverse variance weighted	10	1.116	0.669	1.862	0.674
Faecalibacterium	MR Egger	10	0.973	0.358	2.643	0.958
FamilyXIIIAD3011group	Weighted median	13	0.978	0.489	1.955	0.949

FamilyXIIIAD3011group	MR Egger	13	1.104	0.093	13.078	0.939
FamilyXIIIAD3011group	Inverse variance weighted	13	1.095	0.650	1.844	0.733
FamilyXIIIUCG001	Inverse variance weighted	8	1.138	0.585	2.212	0.703
FamilyXIIIUCG001	MR Egger	8	10.137	1.568	65.551	0.051
FamilyXIIIUCG001	Weighted median	8	1.111	0.483	2.553	0.805
Flavonifractor	Inverse variance weighted	5	0.658	0.272	1.588	0.352
Flavonifractor	MR Egger	5	3.059	0.080	116.718	0.590
Flavonifractor	Weighted median	5	0.798	0.311	2.047	0.639
Fusicatenibacter	Weighted median	18	0.675	0.332	1.373	0.278
Fusicatenibacter	MR Egger	18	1.468	0.196	10.970	0.713
Fusicatenibacter	Inverse variance weighted	18	0.942	0.557	1.592	0.823
Gordonibacter	Weighted median	11	1.326	0.902	1.947	0.151
Gordonibacter	MR Egger	11	1.366	0.395	4.717	0.634
Gordonibacter	Inverse variance weighted	11	1.351	1.008	1.809	0.044
Haemophilus	Weighted median	9	0.769	0.410	1.444	0.415
Haemophilus	Inverse variance weighted	9	0.709	0.440	1.140	0.155
Haemophilus	MR Egger	9	0.935	0.308	2.845	0.910
Holdemanella	Weighted median	11	0.832	0.496	1.396	0.486
Holdemanella	Inverse variance weighted	11	1.007	0.613	1.656	0.977
Holdemanella	MR Egger	11	0.731	0.167	3.205	0.687
Holdemania	Weighted median	14	0.906	0.522	1.573	0.726
Holdemania	Inverse variance weighted	14	1.097	0.734	1.641	0.651
Holdemania	MR Egger	14	0.452	0.138	1.482	0.214
Howardella	Inverse variance weighted	8	0.820	0.538	1.250	0.356
Howardella	Weighted median	8	0.791	0.500	1.251	0.315
Howardella	MR Egger	8	2.352	0.515	10.750	0.312

Hungatella	Weighted median	5	1.272	0.636	2.543	0.496
Hungatella	MR Egger	5	75.589	4.498	1270.219	0.057
Hungatella	Inverse variance weighted	5	1.536	0.784	3.010	0.211
Intestinibacter	Weighted median	15	0.789	0.448	1.389	0.411
Intestinibacter	Inverse variance weighted	15	0.808	0.520	1.256	0.344
Intestinibacter	MR Egger	15	0.802	0.191	3.363	0.768
Intestinimonas	MR Egger	16	0.553	0.140	2.175	0.411
Intestinimonas	Inverse variance weighted	16	0.666	0.412	1.075	0.096
Intestinimonas	Weighted median	16	0.721	0.400	1.300	0.277
Lachnoclostridium	Weighted median	13	1.076	0.501	2.310	0.851
Lachnoclostridium	MR Egger	13	0.533	0.077	3.700	0.538
Lachnoclostridium	Inverse variance weighted	13	0.763	0.432	1.349	0.352
LachnospiraceaeFCS020group	MR Egger	12	0.568	0.112	2.872	0.510
LachnospiraceaeFCS020group	Inverse variance weighted	12	1.174	0.637	2.164	0.608
LachnospiraceaeFCS020group	Weighted median	12	0.825	0.412	1.653	0.587
LachnospiraceaeNC2004group	Weighted median	9	1.165	0.672	2.018	0.586
LachnospiraceaeNC2004group	MR Egger	9	2.273	0.417	12.374	0.374
LachnospiraceaeNC2004group	Inverse variance weighted	9	1.140	0.751	1.729	0.539
LachnospiraceaeND3007group	Inverse variance weighted	3	0.490	0.150	1.597	0.237
LachnospiraceaeND3007group	MR Egger	3	206.460	0.000	8.302E+11	0.719
LachnospiraceaeND3007group	Weighted median	3	0.746	0.161	3.458	0.708
LachnospiraceaeNK4A136group	Inverse variance weighted	15	0.964	0.616	1.508	0.871
LachnospiraceaeNK4A136group	MR Egger	15	1.236	0.505	3.022	0.650
LachnospiraceaeNK4A136group	Weighted median	15	1.095	0.577	2.079	0.780
LachnospiraceaeUCG001	MR Egger	13	1.771	0.192	16.330	0.624
LachnospiraceaeUCG001	Inverse variance weighted	13	1.295	0.791	2.119	0.304

LachnospiraceaeUCG001	Weighted median	13	1.388	0.778	2.477	0.267
LachnospiraceaeUCG004	Inverse variance weighted	12	1.193	0.672	2.115	0.547
LachnospiraceaeUCG004	MR Egger	12	1.508	0.125	18.163	0.753
LachnospiraceaeUCG004	Weighted median	12	1.400	0.635	3.087	0.405
LachnospiraceaeUCG008	Weighted median	10	0.777	0.463	1.306	0.341
LachnospiraceaeUCG008	Inverse variance weighted	10	0.965	0.649	1.435	0.859
LachnospiraceaeUCG008	MR Egger	10	0.439	0.059	3.290	0.446
LachnospiraceaeUCG010	Weighted median	8	0.641	0.268	1.535	0.318
LachnospiraceaeUCG010	MR Egger	8	1.017	0.138	7.515	0.987
LachnospiraceaeUCG010	Inverse variance weighted	8	0.661	0.342	1.279	0.219
Lachnospira	Weighted median	6	0.642	0.186	2.219	0.484
Lachnospira	Inverse variance weighted	6	0.584	0.184	1.857	0.362
Lachnospira	MR Egger	6	7.388	0.005	10003.070	0.616
Lactobacillus	Weighted median	8	1.138	0.646	2.003	0.655
Lactobacillus	MR Egger	8	0.747	0.247	2.266	0.625
Lactobacillus	Inverse variance weighted	8	1.154	0.757	1.759	0.505
Lactococcus	Inverse variance weighted	8	1.097	0.761	1.582	0.619
Lactococcus	MR Egger	8	0.468	0.088	2.503	0.409
Lactococcus	Weighted median	8	1.099	0.683	1.769	0.697
Marvinbryantia	MR Egger	10	0.434	0.032	5.861	0.547
Marvinbryantia	Weighted median	10	0.938	0.421	2.090	0.875
Marvinbryantia	Inverse variance weighted	10	0.823	0.436	1.554	0.548
Methanobrevibacter	Weighted median	6	0.719	0.397	1.302	0.276
Methanobrevibacter	Inverse variance weighted	6	0.849	0.558	1.290	0.443
Methanobrevibacter	MR Egger	6	0.212	0.044	1.016	0.124
Odoribacter	Weighted median	7	1.049	0.386	2.851	0.925

Odoribacter	MR Egger	7	7.351	0.813	66.435	0.136
Odoribacter	Inverse variance weighted	7	1.111	0.547	2.257	0.770
Olsenella	MR Egger	10	2.615	1.011	6.767	0.083
Olsenella	Weighted median	10	1.259	0.828	1.913	0.282
Olsenella	Inverse variance weighted	10	1.163	0.862	1.569	0.322
Oscillibacter	MR Egger	13	1.078	0.232	5.005	0.925
Oscillibacter	Weighted median	13	0.963	0.562	1.648	0.889
Oscillibacter	Inverse variance weighted	13	1.040	0.697	1.554	0.846
Oscillospira	Weighted median	7	1.046	0.485	2.258	0.908
Oscillospira	MR Egger	7	3.535	0.189	66.175	0.437
Oscillospira	Inverse variance weighted	7	0.891	0.423	1.875	0.761
Oxalobacter	MR Egger	11	1.817	0.256	12.911	0.565
Oxalobacter	Weighted median	11	0.925	0.601	1.422	0.721
Oxalobacter	Inverse variance weighted	11	0.999	0.669	1.494	0.997
Parabacteroides	Inverse variance weighted	5	0.619	0.158	2.424	0.491
Parabacteroides	MR Egger	5	193.819	0.131	2.876E+05	0.252
Parabacteroides	Weighted median	5	0.392	0.120	1.286	0.122
Paraprevotella	Weighted median	13	1.034	0.640	1.673	0.890
Paraprevotella	Inverse variance weighted	13	1.046	0.723	1.515	0.810
Paraprevotella	MR Egger	13	0.936	0.219	4.005	0.930
Parasutterella	Inverse variance weighted	14	0.831	0.524	1.319	0.433
Parasutterella	Weighted median	14	1.144	0.624	2.100	0.664
Parasutterella	MR Egger	14	0.387	0.110	1.356	0.163
Peptococcus	Weighted median	12	0.949	0.584	1.541	0.831
Peptococcus	MR Egger	12	0.634	0.169	2.379	0.515
Peptococcus	Inverse variance weighted	12	1.086	0.774	1.523	0.633

Phascolarctobacterium	Inverse variance weighted	8	0.897	0.499	1.611	0.715
Phascolarctobacterium	MR Egger	8	0.813	0.053	12.388	0.886
Phascolarctobacterium	Weighted median	8	1.047	0.495	2.216	0.904
Prevotella7	MR Egger	11	1.341	0.131	13.697	0.810
Prevotella7	Inverse variance weighted	11	1.109	0.758	1.623	0.595
Prevotella7	Weighted median	11	1.075	0.713	1.621	0.728
Prevotella9	Weighted median	15	0.974	0.579	1.641	0.922
Prevotella9	MR Egger	15	0.997	0.332	2.994	0.996
Prevotella9	Inverse variance weighted	15	0.953	0.653	1.390	0.802
RikenellaceaeRC9gutgroup	MR Egger	11	0.896	0.155	5.183	0.905
RikenellaceaeRC9gutgroup	Weighted median	11	1.083	0.738	1.590	0.684
RikenellaceaeRC9gutgroup	Inverse variance weighted	11	1.028	0.775	1.362	0.850
Romboutsia	Inverse variance weighted	13	0.930	0.560	1.545	0.780
Romboutsia	Weighted median	13	0.954	0.490	1.857	0.889
Romboutsia	MR Egger	13	0.525	0.121	2.283	0.409
Roseburia	MR Egger	14	1.459	0.219	9.704	0.703
Roseburia	Weighted median	14	0.690	0.308	1.542	0.365
Roseburia	Inverse variance weighted	14	0.958	0.527	1.744	0.889
Ruminiclostridium5	Weighted median	11	0.750	0.310	1.816	0.524
Ruminiclostridium5	MR Egger	11	0.123	0.007	2.172	0.186
Ruminiclostridium5	Inverse variance weighted	11	0.985	0.477	2.033	0.967
Ruminiclostridium6	Inverse variance weighted	15	1.104	0.653	1.866	0.712
Ruminiclostridium6	MR Egger	15	0.968	0.251	3.734	0.963
Ruminiclostridium6	Weighted median	15	0.864	0.434	1.719	0.676
Ruminiclostridium9	MR Egger	8	11.180	0.132	946.401	0.327
Ruminiclostridium9	Weighted median	8	0.737	0.275	1.974	0.544

Ruminiclostridium9	Inverse variance weighted	8	0.663	0.252	1.742	0.404
RuminococcaceaeNK4A214group	Weighted median	13	1.342	0.642	2.806	0.434
RuminococcaceaeNK4A214group	MR Egger	13	0.244	0.043	1.399	0.142
RuminococcaceaeNK4A214group	Inverse variance weighted	13	1.025	0.604	1.740	0.927
RuminococcaceaeUCG002	Weighted median	20	1.223	0.679	2.202	0.502
RuminococcaceaeUCG002	MR Egger	20	0.561	0.192	1.640	0.305
RuminococcaceaeUCG002	Inverse variance weighted	20	1.056	0.699	1.594	0.797
RuminococcaceaeUCG003	MR Egger	12	2.441	0.244	24.445	0.465
RuminococcaceaeUCG003	Inverse variance weighted	12	1.745	0.889	3.424	0.106
RuminococcaceaeUCG003	Weighted median	12	2.020	0.908	4.493	0.085
RuminococcaceaeUCG004	MR Egger	11	4.804	0.343	67.310	0.274
RuminococcaceaeUCG004	Weighted median	11	0.813	0.433	1.524	0.518
RuminococcaceaeUCG004	Inverse variance weighted	11	1.143	0.708	1.845	0.585
RuminococcaceaeUCG005	MR Egger	13	0.860	0.137	5.419	0.876
RuminococcaceaeUCG005	Inverse variance weighted	13	0.816	0.422	1.579	0.546
RuminococcaceaeUCG005	Weighted median	13	0.871	0.418	1.814	0.713
RuminococcaceaeUCG009	MR Egger	11	2.934	0.554	15.542	0.238
RuminococcaceaeUCG009	Inverse variance weighted	11	1.284	0.832	1.981	0.259
RuminococcaceaeUCG009	Weighted median	11	1.447	0.819	2.556	0.203
RuminococcaceaeUCG010	Inverse variance weighted	6	1.454	0.744	2.842	0.273
RuminococcaceaeUCG010	MR Egger	6	1.128	0.180	7.074	0.904
RuminococcaceaeUCG010	Weighted median	6	1.511	0.697	3.276	0.296
RuminococcaceaeUCG011	Inverse variance weighted	8	0.769	0.556	1.064	0.112
RuminococcaceaeUCG011	Weighted median	8	0.752	0.483	1.171	0.207
RuminococcaceaeUCG011	MR Egger	8	0.635	0.125	3.223	0.604
RuminococcaceaeUCG013	Inverse variance weighted	11	1.409	0.668	2.973	0.369

RuminococcaceaeUCG013	MR Egger	11	0.322	0.043	2.391	0.297
RuminococcaceaeUCG013	Weighted median	11	0.879	0.384	2.015	0.761
RuminococcaceaeUCG014	MR Egger	10	0.888	0.221	3.571	0.872
RuminococcaceaeUCG014	Inverse variance weighted	10	1.169	0.659	2.074	0.593
RuminococcaceaeUCG014	Weighted median	10	1.214	0.587	2.512	0.601
Ruminococcus1	Inverse variance weighted	10	0.928	0.521	1.652	0.800
Ruminococcus1	MR Egger	10	1.396	0.300	6.490	0.682
Ruminococcus1	Weighted median	10	1.062	0.481	2.345	0.882
Ruminococcus2	Inverse variance weighted	15	0.746	0.479	1.160	0.193
Ruminococcus2	Weighted median	15	0.755	0.422	1.349	0.342
Ruminococcus2	MR Egger	15	0.633	0.218	1.834	0.414
Ruminococcusgauvreauiigroup	Inverse variance weighted	11	0.702	0.411	1.198	0.195
Ruminococcusgauvreauiigroup	Weighted median	11	0.549	0.269	1.121	0.100
Ruminococcusgauvreauiigroup	MR Egger	11	0.433	0.048	3.876	0.473
Ruminococcusgnavusgroup	Weighted median	11	0.847	0.495	1.448	0.544
Ruminococcusgnavusgroup	Inverse variance weighted	11	0.859	0.572	1.291	0.464
Ruminococcusgnavusgroup	MR Egger	11	2.280	0.326	15.950	0.428
Ruminococcustorquesgroup	Weighted median	7	0.874	0.291	2.624	0.810
Ruminococcustorquesgroup	MR Egger	7	2.276	0.154	33.698	0.576
Ruminococcustorquesgroup	Inverse variance weighted	7	0.872	0.372	2.045	0.753
Sellimonas	Weighted median	9	0.809	0.546	1.200	0.292
Sellimonas	MR Egger	9	0.559	0.077	4.039	0.583
Sellimonas	Inverse variance weighted	9	0.889	0.645	1.226	0.473
Senegalimassilia	Inverse variance weighted	5	1.109	0.571	2.157	0.760
Senegalimassilia	MR Egger	5	8.159	0.696	95.607	0.193
Senegalimassilia	Weighted median	5	1.056	0.451	2.474	0.900

Slackia	MR Egger	6	1.319	0.010	176.160	0.917
Slackia	Inverse variance weighted	6	0.875	0.446	1.720	0.699
Slackia	Weighted median	6	0.858	0.412	1.790	0.684
Streptococcus	Weighted median	12	0.680	0.313	1.478	0.330
Streptococcus	MR Egger	12	0.596	0.068	5.238	0.651
Streptococcus	Inverse variance weighted	12	0.784	0.436	1.412	0.418
Subdoligranulum	Inverse variance weighted	11	1.133	0.639	2.006	0.669
Subdoligranulum	MR Egger	11	0.294	0.068	1.279	0.137
Subdoligranulum	Weighted median	11	0.976	0.458	2.076	0.949
Sutterella	MR Egger	12	0.592	0.034	10.328	0.727
Sutterella	Inverse variance weighted	12	0.806	0.428	1.516	0.503
Sutterella	Weighted median	12	0.754	0.368	1.544	0.440
Terrisporobacter	MR Egger	5	1.143	0.071	18.519	0.931
Terrisporobacter	Weighted median	5	2.350	0.969	5.699	0.059
Terrisporobacter	Inverse variance weighted	5	1.617	0.720	3.631	0.245
Turicibacter	Inverse variance weighted	9	0.854	0.523	1.395	0.529
Turicibacter	MR Egger	9	0.258	0.035	1.883	0.223
Turicibacter	Weighted median	9	1.123	0.584	2.160	0.728
Tyzzerella3	Inverse variance weighted	12	0.873	0.601	1.267	0.474
Tyzzerella3	MR Egger	12	1.665	0.197	14.042	0.649
Tyzzerella3	Weighted median	12	0.848	0.532	1.353	0.490
unknowngenus	Weighted median	15	0.829	0.479	1.436	0.503
unknowngenus	MR Egger	15	1.113	0.369	3.358	0.853
unknowngenus	Inverse variance weighted	15	0.937	0.626	1.402	0.752
unknowngenus	Weighted median	9	1.176	0.678	2.039	0.564
unknowngenus	Inverse variance weighted	9	1.191	0.793	1.789	0.401

unknowngenus	MR Egger	9	2.179	0.646	7.343	0.249
unknowngenus	MR Egger	12	2.009	0.338	11.950	0.461
unknowngenus	Weighted median	12	0.973	0.508	1.864	0.934
unknowngenus	Inverse variance weighted	12	0.897	0.501	1.605	0.714
unknowngenus	Weighted median	8	0.489	0.261	0.917	0.026
unknowngenus	MR Egger	8	1.503	0.233	9.713	0.683
unknowngenus	Inverse variance weighted	8	0.520	0.325	0.830	0.006
unknowngenus	Inverse variance weighted	12	1.157	0.839	1.597	0.374
unknowngenus	Weighted median	12	1.154	0.749	1.776	0.516
unknowngenus	MR Egger	12	0.715	0.193	2.642	0.625
unknowngenus	Inverse variance weighted	9	1.051	0.532	2.076	0.886
unknowngenus	Weighted median	9	1.259	0.599	2.647	0.543
unknowngenus	MR Egger	9	4.793	0.684	33.560	0.159
unknowngenus	Inverse variance weighted	8	1.105	0.673	1.815	0.693
unknowngenus	Weighted median	8	0.863	0.451	1.649	0.655
unknowngenus	MR Egger	8	0.477	0.128	1.775	0.312
unknowngenus	Weighted median	12	1.325	0.713	2.460	0.373
unknowngenus	MR Egger	12	0.673	0.153	2.959	0.612
unknowngenus	Inverse variance weighted	12	1.253	0.794	1.976	0.332
unknowngenus	Inverse variance weighted	15	1.019	0.574	1.808	0.948
unknowngenus	Weighted median	15	1.021	0.529	1.969	0.951
unknowngenus	MR Egger	15	3.125	0.168	58.252	0.459
unknowngenus	Weighted median	12	1.029	0.615	1.721	0.914
unknowngenus	Inverse variance weighted	12	1.034	0.700	1.529	0.865
unknowngenus	MR Egger	12	0.801	0.198	3.246	0.763
unknowngenus	Inverse variance weighted	13	1.340	0.805	2.231	0.260

unknowngenus	Weighted median	13	1.088	0.559	2.120	0.803
unknowngenus	MR Egger	13	0.810	0.184	3.568	0.786
unknowngenus	Inverse variance weighted	11	0.896	0.644	1.245	0.512
unknowngenus	MR Egger	11	2.596	0.479	14.078	0.297
unknowngenus	Weighted median	11	0.947	0.594	1.511	0.820
Veillonella	MR Egger	5	107.602	0.000	8.208E+08	0.603
Veillonella	Inverse variance weighted	5	2.045	1.032	4.053	0.040
Veillonella	Weighted median	5	1.573	0.603	4.100	0.355
Victivallis	Inverse variance weighted	10	1.090	0.796	1.490	0.592
Victivallis	MR Egger	10	0.334	0.030	3.695	0.397
Victivallis	Weighted median	10	1.204	0.777	1.864	0.406
Actinomycetales	Weighted median	4	0.990	0.433	2.263	0.980
Actinomycetales	Inverse variance weighted	4	1.239	0.503	3.052	0.641
Actinomycetales	MR Egger	4	0.316	0.079	1.272	0.246
Bacillales	MR Egger	8	0.850	0.238	3.037	0.811
Bacillales	Weighted median	8	0.727	0.476	1.111	0.141
Bacillales	Inverse variance weighted	8	0.661	0.482	0.906	0.010
Bacteroidales	MR Egger	12	0.679	0.162	2.837	0.607
Bacteroidales	Inverse variance weighted	12	1.538	0.835	2.835	0.167
Bacteroidales	Weighted median	12	1.297	0.536	3.139	0.565
Bifidobacteriales	MR Egger	11	1.447	0.209	10.023	0.717
Bifidobacteriales	Weighted median	11	0.764	0.369	1.582	0.469
Bifidobacteriales	Inverse variance weighted	11	0.821	0.463	1.457	0.501
Burkholderiales	MR Egger	9	3.325	0.182	60.853	0.445
Burkholderiales	Weighted median	9	0.850	0.346	2.090	0.723
Burkholderiales	Inverse variance weighted	9	0.602	0.234	1.548	0.292

Clostridiales	Inverse variance weighted	12	1.179	0.633	2.193	0.604
Clostridiales	Weighted median	12	1.187	0.530	2.655	0.677
Clostridiales	MR Egger	12	2.912	0.187	45.421	0.463
Coriobacteriales	Weighted median	14	0.792	0.374	1.677	0.543
Coriobacteriales	MR Egger	14	0.507	0.052	4.961	0.570
Coriobacteriales	Inverse variance weighted	14	0.996	0.570	1.740	0.988
Desulfovibrionales	Inverse variance weighted	11	1.297	0.592	2.842	0.515
Desulfovibrionales	MR Egger	11	0.072	0.004	1.389	0.115
Desulfovibrionales	Weighted median	11	0.951	0.409	2.209	0.906
Enterobacteriales	Weighted median	7	0.690	0.256	1.859	0.463
Enterobacteriales	Inverse variance weighted	7	1.436	0.628	3.284	0.391
Enterobacteriales	MR Egger	7	38.841	0.409	3.693E+03	0.176
Erysipelotrichales	Weighted median	12	0.672	0.273	1.654	0.387
Erysipelotrichales	MR Egger	12	0.457	0.014	15.261	0.671
Erysipelotrichales	Inverse variance weighted	12	0.610	0.280	1.331	0.214
Gastranaerophilales	Weighted median	9	1.176	0.666	2.076	0.577
Gastranaerophilales	MR Egger	9	2.179	0.646	7.343	0.249
Gastranaerophilales	Inverse variance weighted	9	1.191	0.793	1.789	0.401
Lactobacillales	MR Egger	15	0.501	0.132	1.903	0.328
Lactobacillales	Weighted median	15	0.942	0.451	1.965	0.873
Lactobacillales	Inverse variance weighted	15	1.019	0.608	1.708	0.943
Methanobacteriales	MR Egger	9	0.398	0.107	1.476	0.211
Methanobacteriales	Weighted median	9	0.594	0.380	0.927	0.022
Methanobacteriales	Inverse variance weighted	9	0.631	0.452	0.881	0.007
MollicutesRF9	Inverse variance weighted	12	0.897	0.501	1.605	0.714
MollicutesRF9	MR Egger	12	2.009	0.338	11.950	0.461

MollicutesRF9	Weighted median	12	0.973	0.501	1.888	0.936
NB1n	Weighted median	12	1.154	0.754	1.764	0.510
NB1n	Inverse variance weighted	12	1.157	0.839	1.597	0.374
NB1n	MR Egger	12	0.715	0.193	2.642	0.625
Pasteurellales	MR Egger	13	1.378	0.535	3.550	0.520
Pasteurellales	Weighted median	13	1.203	0.684	2.113	0.521
Pasteurellales	Inverse variance weighted	13	0.888	0.570	1.382	0.599
Rhodospirillales	Inverse variance weighted	13	1.018	0.691	1.501	0.926
Rhodospirillales	Weighted median	13	1.018	0.604	1.715	0.946
Rhodospirillales	MR Egger	13	0.669	0.146	3.056	0.614
Selenomonadales	Weighted median	12	1.005	0.465	2.171	0.991
Selenomonadales	Inverse variance weighted	12	0.961	0.527	1.755	0.898
Selenomonadales	MR Egger	12	0.428	0.054	3.368	0.439
Verrucomicrobiales	Inverse variance weighted	11	0.915	0.548	1.527	0.734
Verrucomicrobiales	MR Egger	11	1.565	0.261	9.394	0.636
Verrucomicrobiales	Weighted median	11	0.936	0.475	1.843	0.848
Victivallales	MR Egger	8	0.667	0.170	2.613	0.582
Victivallales	Inverse variance weighted	8	0.871	0.593	1.278	0.479
Victivallales	Weighted median	8	0.778	0.488	1.239	0.290
Actinobacteria	Weighted median	14	0.506	0.233	1.095	0.084
Actinobacteria	Inverse variance weighted	14	0.523	0.283	0.968	0.039
Actinobacteria	MR Egger	14	0.367	0.026	5.135	0.471
Bacteroidetes	Weighted median	10	1.169	0.476	2.873	0.733
Bacteroidetes	Inverse variance weighted	10	1.362	0.709	2.615	0.353
Bacteroidetes	MR Egger	10	0.619	0.147	2.611	0.532
Cyanobacteria	Weighted median	8	1.445	0.776	2.690	0.246

Cyanobacteria	Inverse variance weighted	8	1.476	0.929	2.345	0.099
Cyanobacteria	MR Egger	8	0.559	0.108	2.888	0.514
Euryarchaeota	Inverse variance weighted	11	0.919	0.685	1.232	0.572
Euryarchaeota	MR Egger	11	0.695	0.190	2.549	0.597
Euryarchaeota	Weighted median	11	0.910	0.615	1.346	0.636
Firmicutes	MR Egger	14	0.898	0.230	3.497	0.879
Firmicutes	Weighted median	14	1.022	0.496	2.106	0.954
Firmicutes	Inverse variance weighted	14	0.875	0.519	1.473	0.615
Lentisphaerae	Weighted median	9	0.816	0.513	1.298	0.390
Lentisphaerae	Inverse variance weighted	9	0.889	0.621	1.273	0.521
Lentisphaerae	MR Egger	9	0.668	0.170	2.626	0.581
Proteobacteria	Weighted median	12	1.467	0.643	3.349	0.362
Proteobacteria	Inverse variance weighted	12	1.176	0.660	2.095	0.582
Proteobacteria	MR Egger	12	1.058	0.204	5.487	0.948
Tenericutes	Weighted median	12	0.865	0.454	1.648	0.659
Tenericutes	MR Egger	12	1.897	0.375	9.605	0.457
Tenericutes	Inverse variance weighted	12	0.867	0.538	1.399	0.559
Verrucomicrobia	Inverse variance weighted	12	1.185	0.734	1.913	0.487
Verrucomicrobia	MR Egger	12	2.025	0.574	7.147	0.299
Verrucomicrobia	Weighted median	12	1.094	0.576	2.077	0.783

MR, Mendelian randomization; ITP, Immune thrombocytopenia; SNP, single nucleotide polymorphism; OR, odds ratio; CI, confidence interval; U, Upper limitation; L, Lower limitation; IVW, inverse variance weighted; ML, maximum likelihood.

Table S3 The heterogeneity of gut microbiota instrumental variables.

Type	Bacterial taxa (exposure)	ID	Cochran's Q	df	*P-value*
genus	*LachnospiraceaeNC2004group*	11316	4.38	8	0.82
class	*Actinobacteria*	419	12.76	13	0.47
genus	*LachnospiraceaeND3007group*	11317	1.58	2	0.45
class	*Alphaproteobacteria*	2379	3.25	5	0.66
genus	*LachnospiraceaeNK4A136group*	11319	11.06	14	0.68
class	*Bacilli*	1673	19.22	17	0.32
genus	*LachnospiraceaeUCG001*	11321	16.68	12	0.16
class	*Bacteroidia*	912	10.97	11	0.45
genus	*LachnospiraceaeUCG004*	11324	11.32	11	0.42
class	*Clostridia*	1859	10.82	10	0.37
genus	*LachnospiraceaeUCG008*	11328	6.57	9	0.68
class	*Coriobacteriia*	809	10.63	13	0.64
genus	*Lactobacillus*	1837	7.62	7	0.37
class	*Deltaproteobacteria*	3087	17.68	11	0.09
genus	*Lactococcus*	1851	3.06	7	0.88
class	*Gammaproteobacteria*	3303	10.95	6	0.09
genus	*Marvinbryantia*	2005	11.47	9	0.24
class	*Lentisphaeria*	2250	1.94	7	0.96
genus	*Methanobrevibacter*	123	4.26	5	0.51
class	*Melainabacteria*	1589	8.29	9	0.51
genus	*Odoribacter*	952	6.13	6	0.41
class	*Methanobacteria*	119	7.16	8	0.52
genus	*Olsenella*	822	9.16	9	0.42
class	*Mollicutes*	3920	9.33	11	0.59
genus	*Oscillibacter*	2063	10.17	12	0.60
class	*Negativicutes*	2164	4.31	11	0.96
genus	*Oxalobacter*	2978	16.95	10	0.08
class	*Verrucomicrobiae*	4029	10.30	10	0.41
genus	*Parabacteroides*	954	9.72	4	0.05
family	*Acidaminococcaceae*	2166	6.30	6	0.39
genus	*Paraprevotella*	962	13.83	12	0.31
family	*Actinomycetaceae*	421	6.02	3	0.11
genus	*Parasutterella*	2892	16.42	13	0.23
family	*Alcaligenaceae*	2875	9.39	10	0.50
genus	*Peptococcus*	2037	11.70	11	0.39
family	*Bacteroidaceae*	917	5.08	7	0.65
genus	*Phascolarctobacterium*	2168	2.61	7	0.92
family	*BacteroidalesS24*	11173	5.17	7	0.64
genus	*Prevotella7*	11182	16.93	10	0.08

family	*Bifidobacteriaceae*	433	12.72	10	0.24
genus	*Prevotella9*	11183	11.83	14	0.62
family	*Christensenellaceae*	1866	3.55	10	0.97
genus	*RikenellaceaeRC9gutgroup*	11191	8.26	10	0.60
family	*Clostridiaceae1*	1869	8.74	9	0.46
genus	*Romboutsia*	11347	7.37	12	0.83
family	*ClostridialesvadinBB60group*	11286	11.84	14	0.62
genus	*Roseburia*	2012	14.78	13	0.32
family	*Coriobacteriaceae*	811	10.63	13	0.64
genus	*Ruminiclostridium5*	11355	13.51	10	0.20
family	*Defluviitaleaceae*	1924	9.92	10	0.45
genus	*Ruminiclostridium6*	11356	16.67	14	0.27
family	*Enterobacteriaceae*	3469	7.84	6	0.25
genus	*Ruminiclostridium9*	11357	11.93	7	0.10
family	*FamilyXIII*	1957	6.75	6	0.34
genus	*RuminococcaceaeNK4A214group*	11358	12.04	12	0.44
family	*Lachnospiraceae*	1987	16.31	15	0.36
genus	*RuminococcaceaeUCG002*	11360	15.54	19	0.69
family	*Lactobacillaceae*	1836	8.58	7	0.28
genus	*RuminococcaceaeUCG003*	11361	19.06	11	0.06
family	*Methanobacteriaceae*	121	7.16	8	0.52
genus	*RuminococcaceaeUCG004*	11362	10.68	10	0.38
family	*Pasteurellaceae*	3689	17.51	12	0.13
genus	*RuminococcaceaeUCG009*	11366	11.56	10	0.32
family	*Peptococcaceae*	2024	3.99	8	0.86
genus	*RuminococcaceaeUCG010*	11367	0.45	5	0.99
family	*Peptostreptococcaceae*	2042	10.54	12	0.57
genus	*RuminococcaceaeUCG011*	11368	5.41	7	0.61
family	*Porphyromonadaceae*	943	8.11	8	0.42
genus	*RuminococcaceaeUCG013*	11370	16.77	10	0.08
family	*Prevotellaceae*	960	12.50	15	0.64
genus	*RuminococcaceaeUCG014*	11371	10.68	9	0.30
family	*Rhodospirillaceae*	2717	4.49	13	0.98
genus	*Ruminococcus1*	11373	6.99	9	0.64
family	*Rikenellaceae*	967	14.42	15	0.49
genus	*Ruminococcus2*	11374	5.59	14	0.98
family	*Ruminococcaceae*	2050	6.81	9	0.66
genus	*Ruminococcusgauvreauiigroup*	11342	4.07	10	0.94
family	*Streptococcaceae*	1850	4.50	10	0.92
genus	*Ruminococcusgnavusgroup*	14376	11.66	10	0.31
family	*unknownfamily*	100000<br>1214	7.36	8	0.50
genus	*Ruminococcustorquesgroup*	14377	6.28	6	0.39
family	*unknownfamily*	100000	17.85	11	0.09

		5471			
genus	*Sellimonas*	14369	10.09	8	0.26
family	*unknownfamily*	100000	9.92	11	0.54
		6161			
genus	*Senegalimassilia*	11160	4.33	4	0.36
family	*Verrucomicrobiaceae*	4036	10.31	10	0.41
genus	*Slackia*	825	8.16	5	0.15
family	*Victivallaceae*	2255	4.26	10	0.94
genus	*Streptococcus*	1853	7.92	11	0.72
genus	*Actinomyces*	423	9.37	6	0.15
genus	*Subdoligranulum*	2070	6.64	10	0.76
genus	*Akkermansia*	4037	10.31	10	0.41
genus	*Sutterella*	2896	16.24	11	0.13
genus	*Alistipes*	968	10.31	11	0.50
genus	*Terrisporobacter*	11348	7.25	4	0.12
genus	*Allisonella*	2174	13.82	7	0.05
genus	*Turicibacter*	2162	6.29	8	0.62
genus	*Alloprevotella*	961	0.54	4	0.97
genus	*Tyzzerella3*	11335	13.57	11	0.26
genus	*Anaerofilum*	2053	3.30	9	0.95
genus	*unknowngenus*	100000	11.84	14	0.62
		0073			
genus	*Anaerostipes*	1991	12.26	12	0.42
genus	*unknowngenus*	100000	7.36	8	0.50
		1215			
genus	*Bacteroides*	918	5.08	7	0.65
genus	*unknowngenus*	100000	17.85	11	0.09
		5472			
genus	*Barnesiella*	944	2.15	11	1.00
genus	*unknowngenus*	100000	5.17	7	0.64
		5479			
genus	*Bilophila*	3170	7.37	12	0.83
genus	*unknowngenus*	100000	9.92	11	0.54
		6162			
genus	*Blautia*	1992	8.30	11	0.69
genus	*unknowngenus*	1868	13.73	8	0.09
genus	*Butyricicoccus*	2055	4.13	7	0.76
genus	*unknowngenus*	2001	5.95	7	0.55
genus	*Butyricimonas*	945	11.26	12	0.51
genus	*unknowngenus*	2041	13.77	11	0.25
genus	*Butyrivibrio*	1993	6.97	14	0.94
genus	*unknowngenus*	2071	21.91	14	0.08
genus	*CandidatusSoleaferrea*	11350	13.24	8	0.10
genus	*unknowngenus*	2755	5.29	11	0.92

genus	*Catenibacterium*	2153	2.22	3	0.53
genus	*unknowngenus*	826	14.40	12	0.28
genus	*ChristensenellaceaeR*	11283	6.69	7	0.46
genus	*unknowngenus*	959	9.97	10	0.44
genus	*Clostridiuminnocuumgroup*	14397	5.56	7	0.59
genus	*Veillonella*	2198	2.60	4	0.63
genus	*Clostridiumsensustricto1*	1873	3.82	5	0.58
genus	*Victivallis*	2256	9.73	9	0.37
genus	*Collinsella*	815	7.61	8	0.47
order	*Actinomycetales*	420	6.02	3	0.11
genus	*Coprobacter*	949	8.39	10	0.59
order	*Bacillales*	1674	5.39	7	0.61
genus	*Coprococcus1*	11301	4.29	11	0.96
order	*Bacteroidales*	913	10.97	11	0.45
genus	*Coprococcus2*	11302	3.51	7	0.83
order	*Bifidobacteriales*	432	12.72	10	0.24
genus	*Coprococcus3*	11303	7.08	7	0.42
order	*Clostridiales*	1863	11.77	11	0.38
genus	*DefluviitaleaceaeUCG011*	11287	8.43	8	0.39
order	*Coriobacteriales*	810	10.63	13	0.64
genus	*Desulfovibrio*	3173	5.21	9	0.82
order	*Desulfovibrionales*	3156	17.04	10	0.07
genus	*Dialister*	2183	9.90	10	0.45
order	*Enterobacteriales*	3468	7.84	6	0.25
genus	*Dorea*	1997	15.92	9	0.07
order	*Gastranaerophilales*	1591	7.36	8	0.50
genus	*Eggerthella*	819	10.22	8	0.25
order	*Lactobacillales*	1800	12.98	14	0.53
genus	*Eisenbergiella*	11304	9.89	10	0.45
order	*Methanobacteriales*	120	7.16	8	0.52
genus	*Enterorhabdus*	820	1.19	5	0.95
order	*MollicutesRF9*	11579	17.85	11	0.09
genus	*Erysipelatoclostridium*	11381	21.25	14	0.10
order	*NB1n*	3953	9.92	11	0.54
genus	*ErysipelotrichaceaeUCG003*	11384	9.29	15	0.86
order	*Pasteurellales*	3688	17.51	12	0.13
genus	*Escherichia*	3504	14.36	9	0.11
order	*Rhodospirillales*	2667	8.72	12	0.73
genus	*Eubacteriumbrachygroup*	11296	10.05	9	0.35
order	*Selenomonadales*	2165	4.31	11	0.96
genus	*Eubacteriumcoprostanoligenesgroup*	11375	9.78	11	0.55
order	*Verrucomicrobiales*	4030	10.30	10	0.41
genus	*Eubacteriumeligensgroup*	14372	3.78	5	0.58
order	*Victivallales*	2254	1.94	7	0.96

genus	*Eubacteriumfissicatenagroup*	14373	4.34	8	0.83
phylum	*Actinobacteria*	400	16.44	13	0.23
genus	*Eubacteriumhalliigroup*	11338	12.35	13	0.50
phylum	*Bacteroidetes*	905	9.05	9	0.43
genus	*Eubacteriumnodatumgroup*	11297	5.91	10	0.82
phylum	*Cyanobacteria*	1500	6.63	7	0.47
genus	*Eubacteriumoxidoreducensgroup*	11339	5.93	4	0.20
phylum	*Euryarchaeota*	55	9.19	10	0.51
genus	*Eubacteriumrectalegroup*	14374	5.73	7	0.57
phylum	*Firmicutes*	1672	10.62	13	0.64
genus	*Eubacteriumruminantiumgroup*	11340	10.67	17	0.87
phylum	*Lentisphaerae*	2238	2.04	8	0.98
genus	*Eubacteriumventriosumgroup*	11341	2.95	13	1.00
phylum	*Proteobacteria*	2375	10.48	11	0.49
genus	*Eubacteriumxylanophilumgroup*	14375	7.91	8	0.44
phylum	*Tenericutes*	3919	9.33	11	0.59
genus	*Faecalibacterium*	2057	5.02	9	0.83
phylum	*Verrucomicrobia*	3982	10.73	11	0.47
genus	*FamilyXIIIAD3011group*	11293	6.50	12	0.89
class	*Betaproteobacteria*	2867	15.39	8	0.05
genus	*FamilyXIIIUCG001*	11294	8.35	7	0.30
class	*Erysipelotrichia*	2147	17.68	11	0.09
genus	*Flavonifractor*	2059	6.05	4	0.20
family	*Erysipelotrichaceae*	2149	17.68	11	0.09
genus	*Fusicatenibacter*	11305	19.06	17	0.33
family	*FamilyXI*	1936	2.91	5	0.71
genus	*Gordonibacter*	821	5.48	10	0.86
family	*Oxalobacteraceae*	2966	16.68	12	0.16
genus	*Haemophilus*	3698	9.59	8	0.29
family	*Veillonellaceae*	2172	21.62	16	0.16
genus	*Holdemanella*	11393	17.43	10	0.07
genus	*Adlercreutzia*	812	6.83	6	0.34
genus	*Holdemania*	2157	7.48	13	0.88
genus	*Anaerotruncus*	2054	15.51	11	0.16
genus	*Hungatella*	11306	8.36	4	0.08
genus	*Bifidobacterium*	436	12.89	10	0.23
genus	*Intestinibacter*	11345	6.65	14	0.95
genus	*Howardella*	2000	12.63	7	0.08
genus	*Intestinimonas*	2062	19.41	15	0.20
genus	*LachnospiraceaeUCG010*	11330	6.32	7	0.50
genus	*Lachnoclostridium*	11308	11.02	12	0.53
genus	*Oscillospira*	2064	9.29	6	0.16
genus	*Lachnospira*	2004	8.69	5	0.12
genus	*RuminococcaceaeUCG005*	11363	21.81	12	0.04

genus	*LachnospiraceaeFCS020group*	11314	16.26	11	0.13
order	*Burkholderiales*	2874	16.29	8	0.04
family	*Desulfovibrionaceae*	3169	12.34	8	0.14
order	*Erysipelotrichales*	2148	17.68	11	0.09

Df, degree of freedom.

Table S4 Directional horizontal pleiotropy assessed by intercept term in MR Egger regression of the association between gut microbiota and ITP.

Bacterial taxa (exposure)	egger_intercept	se	*pval*
Actinobacteria	-0.05	0.05	0.31
Alphaproteobacteria	0.02	0.10	0.86
Bacilli	0.10	0.05	0.06
Bacteroidia	0.06	0.05	0.24
Clostridia	-0.09	0.09	0.37
Coriobacteriia	0.05	0.08	0.56
Deltaproteobacteria	0.21	0.10	0.05
Gammaproteobacteria	-0.01	0.14	0.92
Lentisphaeria	0.04	0.10	0.70
Melainabacteria	-0.04	0.07	0.58
Methanobacteria	0.08	0.11	0.50
Mollicutes	-0.07	0.07	0.35
Negativicutes	0.05	0.07	0.44
Verrucomicrobiae	-0.04	0.07	0.55
Acidaminococcaceae	0.04	0.10	0.72
Actinomycetaceae	0.18	0.08	0.16
Alcaligenaceae	0.01	0.10	0.90
Bacteroidaceae	0.03	0.12	0.80
BacteroidalesS24	-0.11	0.09	0.29
Bifidobacteriaceae	-0.04	0.07	0.56
Christensenellaceae	0.03	0.04	0.50
Clostridiaceae1	0.08	0.06	0.22
ClostridialesvadinBB60group	-0.02	0.05	0.75
Coriobacteriaceae	0.05	0.08	0.56
Defluviitaleaceae	-0.05	0.08	0.54
Desulfovibrionaceae	0.19	0.10	0.09
Enterobacteriaceae	-0.24	0.17	0.21
FamilyXIII	0.10	0.10	0.38
Lachnospiraceae	0.04	0.06	0.51
Lactobacillaceae	0.06	0.08	0.46
Methanobacteriaceae	0.08	0.11	0.50
Pasteurellaceae	-0.06	0.06	0.33
Peptococcaceae	0.03	0.06	0.61
Peptostreptococcaceae	0.04	0.04	0.35
Porphyromonadaceae	-0.02	0.11	0.86
Prevotellaceae	0.00	0.06	0.99
Rhodospirillaceae	0.00	0.08	0.96
Rikenellaceae	0.05	0.06	0.34
Ruminococcaceae	0.05	0.06	0.35

Streptococcaceae	-0.02	0.10	0.86
unknownfamily	-0.07	0.07	0.34
unknownfamily	-0.07	0.07	0.37
unknownfamily	0.06	0.08	0.47
Verrucomicrobiaceae	-0.04	0.07	0.55
Victivallaceae	-0.02	0.10	0.81
Actinomyces	0.04	0.09	0.68
Akkermansia	-0.04	0.07	0.55
Alistipes	0.02	0.10	0.86
Allisonella	-0.29	0.19	0.19
Alloprevotella	-0.17	0.24	0.54
Anaerofilum	-0.03	0.11	0.80
Anaerostipes	-0.11	0.07	0.12
Bacteroides	0.03	0.12	0.80
Barnesiella	0.01	0.08	0.87
Bilophila	0.00	0.09	0.96
Blautia	0.06	0.06	0.31
Butyricicoccus	-0.03	0.05	0.61
Butyricimonas	0.02	0.08	0.83
Butyrivibrio	-0.10	0.07	0.18
CandidatusSoleaferrea	0.27	0.25	0.32
Catenibacterium	0.26	0.38	0.56
ChristensenellaceaeR	-0.08	0.10	0.46
Clostridiuminnocuumgroup	-0.15	0.12	0.26
Clostridiumsensustricto1	0.10	0.08	0.31
Collinsella	0.12	0.09	0.24
Coprobacter	0.06	0.08	0.46
Coprococcus1	-0.05	0.05	0.39
Coprococcus2	0.08	0.18	0.65
Coprococcus3	0.01	0.13	0.92
DefluviitaleaceaeUCG011	-0.12	0.09	0.23
Desulfovibrio	-0.08	0.07	0.28
Dialister	-0.11	0.08	0.22
Dorea	-0.04	0.09	0.66
Eggerthella	-0.05	0.12	0.66
Eisenbergiella	0.10	0.14	0.49
Enterorhabdus	0.06	0.10	0.56
Erysipelatoclostridium	0.02	0.08	0.79
ErysipelotrichaceaeUCG003	0.01	0.06	0.88
Escherichia	0.13	0.08	0.15
Eubacteriumbrachygroup	-0.07	0.09	0.49
Eubacteriumcoprostanoligenesgroup	0.10	0.07	0.21
Eubacteriumeligensgroup	-0.04	0.12	0.73
Eubacteriumfissicatenagroup	-0.01	0.12	0.96

Eubacteriumhalliigroup	-0.02	0.04	0.63
Eubacteriumnodatumgroup	-0.02	0.09	0.84
Eubacteriumoxidoreducensgroup	0.04	0.15	0.82
Eubacteriumrectalegroup	0.07	0.08	0.41
Eubacteriumruminantiumgroup	-0.03	0.05	0.53
Eubacteriumventriosumgroup	0.03	0.09	0.77
Eubacteriumxylanophilumgroup	-0.09	0.07	0.25
Faecalibacterium	0.02	0.05	0.76
FamilyXIIIAD3011group	0.00	0.10	1.00
FamilyXIIIUCG001	-0.19	0.08	0.05
Flavonifractor	-0.13	0.15	0.46
Fusicatenibacter	-0.03	0.07	0.66
Gordonibacter	0.00	0.09	0.99
Haemophilus	-0.04	0.07	0.60
Holdemanella	0.04	0.08	0.66
Holdemania	0.09	0.06	0.15
Hungatella	-0.51	0.19	0.07
Intestinibacter	0.00	0.06	0.99
Intestinimonas	0.02	0.06	0.78
Lachnoclostridium	0.03	0.07	0.71
Lachnospira	-0.15	0.22	0.52
LachnospiraceaeFCS020group	0.06	0.06	0.37
LachnospiraceaeNC2004group	-0.08	0.10	0.44
LachnospiraceaeND3007group	-0.36	0.66	0.69
LachnospiraceaeNK4A136group	-0.02	0.03	0.54
LachnospiraceaeUCG001	-0.03	0.10	0.78
LachnospiraceaeUCG004	-0.02	0.08	0.85
LachnospiraceaeUCG008	0.08	0.11	0.46
Lactobacillus	0.05	0.07	0.44
Lactococcus	0.12	0.12	0.35
Marvinbryantia	0.06	0.11	0.63
Methanobrevibacter	0.21	0.11	0.15
Odoribacter	-0.15	0.08	0.14
Olsenella	-0.12	0.07	0.12
Oscillibacter	0.00	0.07	0.96
Oxalobacter	-0.09	0.15	0.56
Parabacteroides	-0.50	0.32	0.22
Paraprevotella	0.01	0.08	0.88
Parasutterella	0.07	0.05	0.22
Peptococcus	0.07	0.09	0.43
Phascolarctobacterium	0.01	0.12	0.94
Prevotella7	-0.03	0.17	0.87
Prevotella9	0.00	0.06	0.93
RikenellaceaeRC9gutgroup	0.02	0.12	0.88

Romboutsia	0.05	0.06	0.43
Roseburia	-0.03	0.07	0.65
Ruminiclostridium5	0.13	0.09	0.18
Ruminiclostridium6	0.01	0.06	0.84
Ruminiclostridium9	-0.18	0.14	0.25
RuminococcaceaeNK4A214group	0.11	0.06	0.12
RuminococcaceaeUCG002	0.05	0.04	0.23
RuminococcaceaeUCG003	-0.03	0.09	0.77
RuminococcaceaeUCG004	-0.12	0.11	0.31
RuminococcaceaeUCG009	-0.08	0.08	0.34
RuminococcaceaeUCG010	0.02	0.07	0.79
RuminococcaceaeUCG011	0.03	0.11	0.82
RuminococcaceaeUCG013	0.12	0.08	0.16
RuminococcaceaeUCG014	0.03	0.06	0.68
Ruminococcus1	-0.03	0.06	0.59
Ruminococcus2	0.01	0.04	0.74
Ruminococcusgauvreauiigroup	0.04	0.08	0.67
Ruminococcusgnavusgroup	-0.11	0.11	0.34
Ruminococcustorquesgroup	-0.07	0.09	0.49
Sellimonas	0.07	0.15	0.66
Senegalimassilia	-0.19	0.11	0.20
Slackia	-0.04	0.25	0.88
Streptococcus	0.02	0.09	0.80
Subdoligranulum	0.11	0.05	0.08
Sutterella	0.02	0.10	0.83
Terrisporobacter	0.04	0.14	0.81
Turicibacter	0.13	0.11	0.26
Tyzzerella3	-0.09	0.15	0.56
unknowngenus	-0.02	0.05	0.75
unknowngenus	-0.07	0.07	0.34
unknowngenus	-0.07	0.07	0.37
unknowngenus	-0.11	0.09	0.29
unknowngenus	0.06	0.08	0.47
unknowngenus	-0.14	0.09	0.15
unknowngenus	0.10	0.07	0.22
unknowngenus	0.06	0.07	0.41
unknowngenus	-0.09	0.12	0.46
unknowngenus	0.03	0.07	0.72
unknowngenus	0.05	0.07	0.49
unknowngenus	-0.12	0.10	0.24
Veillonella	-0.29	0.59	0.66
Victivallis	0.16	0.16	0.36
Actinomycetales	0.18	0.08	0.16
Bacillales	-0.04	0.10	0.70

Bacteroidales	0.06	0.05	0.24
Bifidobacteriales	-0.04	0.07	0.56
Clostridiales	-0.06	0.09	0.52
Coriobacteriales	0.05	0.08	0.56
Desulfovibrionales	0.19	0.10	0.08
Enterobacteriales	-0.24	0.17	0.21
Gastranaerophilales	-0.07	0.07	0.34
Lactobacillales	0.06	0.05	0.28
Methanobacteriales	0.08	0.11	0.50
MollicutesRF9	-0.07	0.07	0.37
NB1n	0.06	0.08	0.47
Pasteurellales	-0.06	0.06	0.33
Rhodospirillales	0.04	0.08	0.59
Selenomonadales	0.05	0.07	0.44
Verrucomicrobiales	-0.04	0.07	0.55
Victivallales	0.04	0.10	0.70
Actinobacteria	0.02	0.08	0.79
Bacteroidetes	0.06	0.05	0.26
Cyanobacteria	0.12	0.10	0.27
Euryarchaeota	0.04	0.09	0.68
Firmicutes	0.00	0.05	0.97
Lentisphaerae	0.04	0.10	0.68
Proteobacteria	0.01	0.06	0.90
Tenericutes	-0.07	0.07	0.35
Verrucomicrobia	-0.05	0.06	0.39
Betaproteobacteria	0.07	0.11	0.53
Erysipelotrichia	0.02	0.11	0.87
Erysipelotrichaceae	0.02	0.11	0.87
FamilyXI	-0.03	0.15	0.88
Oxalobacteraceae	-0.04	0.10	0.69
Veillonellaceae	0.04	0.04	0.39
Adlercreutzia	0.19	0.11	0.14
Anaerotruncus	-0.09	0.08	0.32
Bifidobacterium	-0.10	0.07	0.21
Howardella	-0.17	0.12	0.21
LachnospiraceaeUCG010	-0.03	0.08	0.67
Oscillospira	-0.14	0.15	0.38
RuminococcaceaeUCG005	0.00	0.08	0.95
Burkholderiales	-0.13	0.11	0.26
Erysipelotrichales	0.02	0.11	0.87

MR, Mendelian randomization; PE, preeclampsia-eclampsia; SE, standard error.

Table S5 MR-PRESSO analysis for the association between gut microbiota and ITP.

Bacterial taxa (exposure)	MR Analysis	Causal Estimate	SD	T	*P-value*	RSSobs	Global test *P*-value	Remove SNP
Actinobacteria	MR-PRESSO	-0.42	0.24	-1.74	0.11	14.62	0.50	
Alphaproteobacteria	MR-PRESSO	-0.03	0.25	-0.14	0.90	4.44	0.71	
Bacilli	MR-PRESSO	0.06	0.25	0.23	0.82	22.05	0.30	
Bacteroidia	MR-PRESSO	0.43	0.31	1.38	0.19	13.20	0.45	
Betaproteobacteria	MR-PRESSO	-0.21	0.52	-0.41	0.69	26.99	0.01	
Betaproteobacteria	Outlier-corrected	0.08	0.48	0.16	0.88	18.33	0.08	1 SNP
Clostridia	MR-PRESSO	0.15	0.33	0.46	0.66	12.94	0.39	
Coriobacteriia	MR-PRESSO	0.00	0.26	-0.02	0.99	12.37	0.65	
Deltaproteobacteria	MR-PRESSO	0.22	0.37	0.59	0.57	20.68	0.11	
Erysipelotrichia	MR-PRESSO	-0.34	0.43	-0.79	0.44	27.24	0.03	
Erysipelotrichia	Outlier-corrected	-0.49	0.40	-1.24	0.24	20.88	0.10	1 SNP
Gammaproteobacteria	MR-PRESSO	-0.33	0.53	-0.63	0.55	14.23	0.12	
Lentisphaeria	MR-PRESSO	-0.14	0.10	-1.34	0.22	2.50	0.97	
Melainabacteria	MR-PRESSO	0.25	0.19	1.30	0.23	10.34	0.51	
Methanobacteria	MR-PRESSO	-0.46	0.16	-2.86	0.02	9.25	0.56	
Mollicutes	MR-PRESSO	-0.14	0.22	-0.63	0.54	11.02	0.60	
Negativicutes	MR-PRESSO	-0.04	0.19	-0.20	0.84	5.17	0.96	
Verrucomicrobiae	MR-PRESSO	-0.09	0.26	-0.34	0.74	12.00	0.45	
Acidaminococcaceae	MR-PRESSO	-0.04	0.31	-0.12	0.91	8.04	0.44	

Actinomycetaceae	MR-PRESSO	0.21	0.46	0.46	0.67	12.47	0.25	
Alcaligenaceae	MR-PRESSO	0.87	0.31	2.81	0.02	11.33	0.53	
Bacteroidaceae	MR-PRESSO	-0.33	0.31	-1.07	0.32	6.32	0.68	
BacteroidalesS24	MR-PRESSO	-0.65	0.21	-3.19	0.02	6.68	0.67	
Bifidobacteriaceae	MR-PRESSO	-0.20	0.29	-0.67	0.52	15.74	0.25	
Christensenellaceae	MR-PRESSO	0.29	0.15	1.97	0.08	4.00	0.98	
Clostridiaceae1	MR-PRESSO	0.05	0.29	0.17	0.87	11.23	0.45	
ClostridialesvadinBB60group	MR-PRESSO	-0.06	0.19	-0.34	0.74	14.04	0.61	
Coriobacteriaceae	MR-PRESSO	0.00	0.26	-0.02	0.99	12.37	0.64	
Defluviitaleaceae	MR-PRESSO	0.03	0.23	0.14	0.89	12.07	0.46	
Desulfovibrionaceae	MR-PRESSO	0.09	0.41	0.22	0.83	15.37	0.15	
Enterobacteriaceae	MR-PRESSO	0.36	0.42	0.86	0.42	10.77	0.26	
Erysipelotrichaceae	MR-PRESSO	-0.34	0.43	-0.79	0.44	27.24	0.03	
Erysipelotrichaceae	Outlier-corrected	-0.49	0.40	-1.24	0.24	20.88	0.12	1 SNP
FamilyXI	MR-PRESSO	0.21	0.24	0.88	0.41	20.85	0.03	
FamilyXI	Outlier-corrected	0.23	0.14	1.70	0.15	4.05	0.73	2 SNP
FamilyXIII	MR-PRESSO	-0.12	0.43	-0.28	0.79	9.30	0.37	
Lachnospiraceae	MR-PRESSO	-0.61	0.29	-2.07	0.06	18.60	0.38	
Lactobacillaceae	MR-PRESSO	0.07	0.24	0.29	0.78	10.56	0.34	
Methanobacteriaceae	MR-PRESSO	-0.46	0.16	-2.86	0.02	9.25	0.55	
Oxalobacteraceae	MR-PRESSO	0.20	0.21	0.96	0.35	30.15	0.02	
Oxalobacteraceae	Outlier-corrected	0.33	0.18	1.79	0.10	19.74	0.20	1SNP
Pasteurellaceae	MR-PRESSO	-0.12	0.23	-0.53	0.61	21.18	0.13	

Peptococcaceae	MR-PRESSO	0.21	0.18	1.16	0.28	5.13	0.86	
Peptostreptococcaceae	MR-PRESSO	-0.11	0.23	-0.49	0.63	12.68	0.55	
Porphyromonadaceae	MR-PRESSO	-0.03	0.39	-0.08	0.94	10.38	0.43	
Prevotellaceae	MR-PRESSO	0.25	0.22	1.15	0.27	14.07	0.66	
Rhodospirillaceae	MR-PRESSO	-0.23	0.11	-1.98	0.07	5.19	0.98	
Rikenellaceae	MR-PRESSO	0.15	0.25	0.59	0.56	16.88	0.46	
Ruminococcaceae	MR-PRESSO	0.09	0.26	0.36	0.73	8.19	0.68	
Streptococcaceae	MR-PRESSO	-0.34	0.21	-1.61	0.14	5.57	0.93	
unknownfamily	MR-PRESSO	0.17	0.20	0.88	0.41	9.58	0.51	
unknownfamily	MR-PRESSO	-0.11	0.30	-0.37	0.72	20.93	0.10	
unknownfamily	MR-PRESSO	0.15	0.16	0.94	0.37	11.43	0.57	
Veillonellaceae	MR-PRESSO	0.00	0.28	0.01	0.99	33.21	0.04	
Veillonellaceae	Outlier-corrected	0.14	0.25	0.58	0.57	23.86	0.18	1 SNP
Verrucomicrobiaceae	MR-PRESSO	-0.09	0.26	-0.34	0.74	12.00	0.46	
Victivallaceae	MR-PRESSO	-0.03	0.10	-0.33	0.75	5.04	0.94	
Actinomyces	MR-PRESSO	-0.18	0.31	-0.56	0.59	12.05	0.20	
Adlercreutzia	MR-PRESSO	0.31	0.40	0.78	0.46	20.81	0.03	
Adlercreutzia	Outlier-corrected	0.60	0.30	1.99	0.09	9.35	0.39	1 SNP
Akkermansia	MR-PRESSO	-0.09	0.26	-0.34	0.74	12.01	0.45	
Alistipes	MR-PRESSO	-0.27	0.32	-0.84	0.42	12.02	0.53	
Allisonella	MR-PRESSO	-0.54	0.22	-2.46	0.04	17.24	0.09	
Alloprevotella	MR-PRESSO	0.27	0.07	3.75	0.02	0.83	0.98	
Anaerofilum	MR-PRESSO	0.21	0.11	1.90	0.09	4.08	0.95	
Anaerostipes	MR-PRESSO	0.26	0.30	0.86	0.41	14.21	0.45	

Anaerotruncus	MR-PRESSO	-0.21	0.41	-0.51	0.62	31.00	0.02	
Anaerotruncus	Outlier-corrected	0.18	0.37	0.49	0.63	18.39	0.19	1 SNP
Bacteroides	MR-PRESSO	-0.33	0.31	-1.07	0.32	6.32	0.70	
Barnesiella	MR-PRESSO	0.15	0.12	1.20	0.26	2.57	1.00	
Bifidobacterium	MR-PRESSO	-0.18	0.32	-0.56	0.59	26.53	0.04	
Bifidobacterium	Outlier-corrected	-0.50	0.30	-1.67	0.12	15.31	0.30	1 SNP
Bilophila	MR-PRESSO	0.11	0.21	0.53	0.61	8.59	0.84	
Blautia	MR-PRESSO	0.36	0.27	1.34	0.21	9.96	0.69	
Butyricicoccus	MR-PRESSO	-0.27	0.25	-1.06	0.32	5.49	0.77	
Butyricimonas	MR-PRESSO	-0.07	0.24	-0.31	0.76	13.30	0.50	
Butyrivibrio	MR-PRESSO	-0.03	0.08	-0.32	0.76	7.90	0.94	
CandidatusSoleaferrea	MR-PRESSO	0.38	0.27	1.41	0.19	16.75	0.13	
Catenibacterium	MR-PRESSO	-0.30	0.20	-1.45	0.24	4.05	0.56	
ChristensenellaceaeR	MR-PRESSO	0.54	0.38	1.43	0.20	8.27	0.52	
Clostridiuminnocuumgroup	MR-PRESSO	-0.05	0.16	-0.34	0.74	7.31	0.60	
Clostridiumsensustricto1	MR-PRESSO	0.20	0.28	0.72	0.50	6.55	0.53	
Collinsella	MR-PRESSO	0.24	0.34	0.70	0.50	9.64	0.49	
Coprobacter	MR-PRESSO	-0.23	0.18	-1.26	0.23	10.20	0.60	
Coprococcus1	MR-PRESSO	-0.20	0.17	-1.19	0.26	5.10	0.96	
Coprococcus2	MR-PRESSO	-0.65	0.22	-2.94	0.02	4.60	0.84	
Coprococcus3	MR-PRESSO	0.56	0.38	1.47	0.18	9.56	0.42	
DefluviitaleaceaeUCG011	MR-PRESSO	0.17	0.26	0.66	0.53	10.72	0.40	
Desulfovibrio	MR-PRESSO	-0.27	0.19	-1.44	0.18	6.68	0.80	
Dialister	MR-PRESSO	0.14	0.27	0.54	0.60	12.06	0.46	

Dorea	MR-PRESSO	-0.23	0.46	-0.50	0.63	19.26	0.09
Eggerthella	MR-PRESSO	0.07	0.22	0.32	0.76	12.98	0.27
Eisenbergiella	MR-PRESSO	-0.05	0.18	-0.27	0.79	11.80	0.46
Enterorhabdus	MR-PRESSO	-0.10	0.14	-0.69	0.52	1.99	0.92
Erysipelatoclostridium	MR-PRESSO	0.21	0.25	0.82	0.42	24.32	0.10
ErysipelotrichaceaeUCG003	MR-PRESSO	0.14	0.18	0.76	0.46	10.57	0.86
Escherichia	MR-PRESSO	0.16	0.37	0.42	0.68	17.25	0.14
Eubacteriumbrachygroup	MR-PRESSO	0.29	0.17	1.65	0.13	12.39	0.37
Eubacteriumcoprostanoligenesgroup	MR-PRESSO	0.46	0.30	1.56	0.15	11.81	0.55
Eubacteriumeligensgroup	MR-PRESSO	0.12	0.34	0.37	0.73	5.55	0.59
Eubacteriumfissicatenagroup	MR-PRESSO	-0.04	0.13	-0.32	0.76	5.46	0.83
Eubacteriumhalliigroup	MR-PRESSO	-0.43	0.24	-1.81	0.09	14.54	0.51
Eubacteriumnodatumgroup	MR-PRESSO	0.04	0.11	0.35	0.73	7.08	0.82
Eubacteriumoxidoreducensgroup	MR-PRESSO	-0.14	0.32	-0.43	0.69	9.35	0.25
Eubacteriumrectalegroup	MR-PRESSO	-0.04	0.33	-0.11	0.92	7.58	0.58
Eubacteriumruminantiumgroup	MR-PRESSO	-0.32	0.13	-2.56	0.02	11.91	0.88
Eubacteriumventriosumgroup	MR-PRESSO	-0.02	0.13	-0.16	0.87	3.41	1.00
Eubacteriumxylanophilumgroup	MR-PRESSO	-0.07	0.28	-0.26	0.80	9.80	0.46
Faecalibacterium	MR-PRESSO	0.11	0.19	0.56	0.59	5.82	0.86
FamilyXIIIAD3011group	MR-PRESSO	0.09	0.20	0.46	0.65	7.76	0.88
FamilyXIIIUCG001	MR-PRESSO	0.13	0.34	0.38	0.71	11.26	0.31
Flavonifractor	MR-PRESSO	-0.42	0.45	-0.93	0.40	9.13	0.25
Fusicatenibacter	MR-PRESSO	-0.06	0.27	-0.22	0.83	21.20	0.34
Gordonibacter	MR-PRESSO	0.30	0.11	2.72	0.02	6.67	0.86
Haemophilus	MR-PRESSO	-0.34	0.24	-1.42	0.19	13.68	0.28

Holdemanella	MR-PRESSO	0.01	0.25	0.03	0.98	20.36	0.08	
Holdemania	MR-PRESSO	0.09	0.16	0.60	0.56	8.72	0.88	
Howardella	MR-PRESSO	-0.10	0.23	-0.45	0.67	22.32	0.03	
Howardella	Outlier-corrected	-0.20	0.21	-0.92	0.39	15.89	0.11	1 SNP
Hungatella	MR-PRESSO	0.43	0.34	1.25	0.28	13.38	0.12	
Intestinibacter	MR-PRESSO	-0.21	0.15	-1.37	0.19	7.53	0.95	
Intestinimonas	MR-PRESSO	-0.41	0.24	-1.66	0.12	21.88	0.22	
Lachnoclostridium	MR-PRESSO	-0.27	0.28	-0.97	0.35	12.61	0.57	
LachnospiraceaeFCS020group	MR-PRESSO	0.16	0.31	0.51	0.62	19.01	0.15	
LachnospiraceaeNC2004group	MR-PRESSO	0.13	0.16	0.83	0.43	5.66	0.81	
LachnospiraceaeNK4A136group	MR-PRESSO	-0.04	0.20	-0.18	0.86	12.31	0.71	
LachnospiraceaeUCG001	MR-PRESSO	0.26	0.25	1.03	0.32	19.37	0.18	
LachnospiraceaeUCG004	MR-PRESSO	0.18	0.29	0.60	0.56	13.56	0.42	
LachnospiraceaeUCG008	MR-PRESSO	-0.04	0.17	-0.21	0.84	8.40	0.67	
LachnospiraceaeUCG010	MR-PRESSO	-0.33	0.48	-0.69	0.51	27.50	0.01	
LachnospiraceaeUCG010	Outlier-corrected	-0.41	0.32	-1.29	0.24	8.79	0.49	2 SNP
Lachnospira	MR-PRESSO	-0.54	0.59	-0.91	0.40	13.36	0.14	
Lactobacillus	MR-PRESSO	0.14	0.22	0.67	0.53	9.48	0.42	
Lactococcus	MR-PRESSO	0.09	0.12	0.75	0.48	3.85	0.89	
Marvinbryantia	MR-PRESSO	-0.19	0.32	-0.60	0.56	14.52	0.24	
Methanobrevibacter	MR-PRESSO	-0.16	0.20	-0.83	0.44	6.45	0.51	
Odoribacter	MR-PRESSO	0.11	0.36	0.29	0.78	8.23	0.45	
Olsenella	MR-PRESSO	0.15	0.15	0.99	0.35	11.62	0.43	
Oscillibacter	MR-PRESSO	0.04	0.19	0.21	0.84	11.90	0.61	

Oscillospira	MR-PRESSO	0.17	0.42	0.42	0.69	20.04	0.04	
Oscillospira	Outlier-corrected	-0.12	0.38	-0.30	0.77	12.34	0.19	1 SNP
Oxalobacter	MR-PRESSO	0.00	0.21	0.00	1.00	20.27	0.09	
Parabacteroides	MR-PRESSO	-0.48	0.70	-0.69	0.53	14.19	0.09	
Paraprevotella	MR-PRESSO	0.05	0.19	0.24	0.81	16.42	0.32	
Parasutterella	MR-PRESSO	-0.18	0.24	-0.78	0.45	20.34	0.20	
Peptococcus	MR-PRESSO	0.08	0.17	0.48	0.64	14.50	0.37	
Phascolarctobacterium	MR-PRESSO	-0.11	0.18	-0.60	0.57	3.48	0.92	
Prevotella7	MR-PRESSO	0.10	0.19	0.53	0.61	20.55	0.08	
Prevotella9	MR-PRESSO	-0.05	0.18	-0.27	0.79	13.10	0.67	
RikenellaceaeRC9gutgroup	MR-PRESSO	0.03	0.13	0.21	0.84	9.94	0.61	
Romboutsia	MR-PRESSO	-0.07	0.20	-0.36	0.73	8.77	0.83	
Roseburia	MR-PRESSO	-0.04	0.31	-0.14	0.89	17.07	0.34	
Ruminiclostridium5	MR-PRESSO	-0.02	0.37	-0.04	0.97	16.43	0.20	
Ruminiclostridium6	MR-PRESSO	0.10	0.27	0.37	0.72	18.91	0.30	
Ruminiclostridium9	MR-PRESSO	-0.41	0.49	-0.83	0.43	15.78	0.12	
RuminococcaceaeNK4A214group	MR-PRESSO	0.02	0.27	0.09	0.93	14.02	0.46	
RuminococcaceaeUCG002	MR-PRESSO	0.05	0.19	0.29	0.78	16.99	0.70	
RuminococcaceaeUCG003	MR-PRESSO	0.56	0.34	1.62	0.13	22.29	0.08	
RuminococcaceaeUCG004	MR-PRESSO	0.13	0.24	0.55	0.60	12.75	0.42	
RuminococcaceaeUCG005	MR-PRESSO	-0.02	0.37	-0.04	0.97	34.86	0.00	
RuminococcaceaeUCG005	Outlier-corrected	-0.20	0.34	-0.60	0.56	24.60	0.06	1 SNP
RuminococcaceaeUCG009	MR-PRESSO	0.25	0.22	1.13	0.29	14.20	0.32	
RuminococcaceaeUCG010	MR-PRESSO	0.37	0.10	3.65	0.01	0.66	0.99	

RuminococcaceaeUCG011	MR-PRESSO	-0.26	0.15	-1.81	0.11	6.98	0.64
RuminococcaceaeUCG013	MR-PRESSO	0.34	0.38	0.90	0.39	19.69	0.10
RuminococcaceaeUCG014	MR-PRESSO	0.16	0.29	0.53	0.61	12.09	0.39
Ruminococcus1	MR-PRESSO	-0.07	0.26	-0.29	0.78	8.71	0.63
Ruminococcus2	MR-PRESSO	-0.29	0.14	-2.06	0.06	6.25	0.98
Ruminococcusgauvreauiigroup	MR-PRESSO	-0.35	0.17	-2.03	0.07	5.00	0.94
Ruminococcusgnavusgroup	MR-PRESSO	-0.15	0.21	-0.73	0.48	13.90	0.33
Ruminococcustorquesgroup	MR-PRESSO	-0.14	0.44	-0.32	0.76	8.95	0.38
Sellimonas	MR-PRESSO	-0.12	0.16	-0.72	0.49	12.61	0.28
Senegalimassilia	MR-PRESSO	0.10	0.34	0.31	0.77	7.00	0.39
Slackia	MR-PRESSO	-0.13	0.34	-0.39	0.72	12.29	0.17
Streptococcus	MR-PRESSO	-0.24	0.25	-0.95	0.36	9.49	0.73
Subdoligranulum	MR-PRESSO	0.12	0.24	0.52	0.61	8.20	0.75
Sutterella	MR-PRESSO	-0.22	0.32	-0.67	0.52	19.16	0.15
Terrisporobacter	MR-PRESSO	0.48	0.41	1.16	0.31	10.97	0.18
Turicibacter	MR-PRESSO	-0.16	0.22	-0.71	0.50	7.95	0.63
Tyzzerella3	MR-PRESSO	-0.14	0.19	-0.72	0.49	16.55	0.26
unknowngenus	MR-PRESSO	-0.06	0.19	-0.34	0.74	14.04	0.59
unknowngenus	MR-PRESSO	0.17	0.20	0.88	0.41	9.58	0.51
unknowngenus	MR-PRESSO	-0.11	0.30	-0.37	0.72	20.93	0.10
unknowngenus	MR-PRESSO	-0.65	0.21	-3.19	0.02	6.68	0.67
unknowngenus	MR-PRESSO	0.15	0.16	0.94	0.37	11.43	0.58
unknowngenus	MR-PRESSO	0.05	0.35	0.14	0.89	17.44	0.10
unknowngenus	MR-PRESSO	0.10	0.23	0.43	0.68	9.05	0.48
unknowngenus	MR-PRESSO	0.23	0.23	0.97	0.35	16.63	0.25
unknowngenus	MR-PRESSO	0.02	0.29	0.07	0.95	25.61	0.07

unknowngenus	MR-PRESSO	0.03	0.14	0.24	0.81	6.09	0.92	
unknowngenus	MR-PRESSO	0.29	0.26	1.13	0.28	16.75	0.30	
unknowngenus	MR-PRESSO	-0.11	0.17	-0.66	0.53	12.20	0.44	
Veillonella	MR-PRESSO	0.72	0.28	2.54	0.06	4.11	0.66	
Victivallis	MR-PRESSO	0.09	0.16	0.54	0.60	12.02	0.39	
Actinomycetales	MR-PRESSO	0.21	0.46	0.47	0.67	12.49	0.26	
Bacillales	MR-PRESSO	-0.41	0.14	-2.93	0.02	6.82	0.67	
Bacteroidales	MR-PRESSO	0.43	0.31	1.38	0.19	13.20	0.45	
Bifidobacteriales	MR-PRESSO	-0.20	0.29	-0.67	0.52	15.74	0.26	
Burkholderiales	MR-PRESSO	-0.29	0.49	-0.59	0.57	24.75	0.02	
Burkholderiales	Outlier-corrected	-0.51	0.48	-1.05	0.32	19.57	0.07	1 SNP
Clostridiales	MR-PRESSO	0.16	0.32	0.52	0.61	13.90	0.40	
Coriobacteriales	MR-PRESSO	0.00	0.26	-0.02	0.99	12.37	0.63	
Desulfovibrionales	MR-PRESSO	0.26	0.40	0.65	0.53	20.21	0.08	
Enterobacteriales	MR-PRESSO	0.36	0.42	0.86	0.42	10.77	0.27	
Erysipelotrichales	MR-PRESSO	-0.34	0.43	-0.79	0.44	27.24	0.03	
Erysipelotrichales	Outlier-corrected	-0.49	0.40	-1.24	0.24	20.88	0.10	1 SNP
Gastranaerophilales	MR-PRESSO	0.17	0.20	0.88	0.41	9.58	0.50	
Lactobacillales	MR-PRESSO	0.02	0.25	0.07	0.94	15.39	0.50	
Methanobacteriales	MR-PRESSO	-0.46	0.16	-2.86	0.02	9.25	0.55	
MollicutesRF9	MR-PRESSO	-0.11	0.30	-0.37	0.72	20.93	0.10	
NB1n	MR-PRESSO	0.15	0.16	0.94	0.37	11.43	0.57	
Pasteurellales	MR-PRESSO	-0.12	0.23	-0.53	0.61	21.18	0.13	
Rhodospirillales	MR-PRESSO	0.02	0.17	0.11	0.92	10.00	0.75	

Selenomonadales	MR-PRESSO	-0.04	0.19	-0.20	0.84	5.17	0.95
Verrucomicrobiales	MR-PRESSO	-0.09	0.26	-0.34	0.74	12.00	0.44
Victivallales	MR-PRESSO	-0.14	0.10	-1.34	0.22	2.50	0.97
Actinobacteria	MR-PRESSO	-0.65	0.31	-2.07	0.06	18.63	0.27
Bacteroidetes	MR-PRESSO	0.31	0.33	0.93	0.38	11.32	0.44
Cyanobacteria	MR-PRESSO	0.39	0.23	1.69	0.13	8.95	0.47
Euryarchaeota	MR-PRESSO	-0.08	0.14	-0.59	0.57	11.14	0.52
Firmicutes	MR-PRESSO	-0.13	0.24	-0.56	0.59	11.95	0.67
Lentisphaerae	MR-PRESSO	-0.12	0.09	-1.27	0.24	2.55	0.98
Proteobacteria	MR-PRESSO	0.16	0.29	0.56	0.58	13.22	0.45
Tenericutes	MR-PRESSO	-0.14	0.22	-0.63	0.54	11.02	0.61
Verrucomicrobia	MR-PRESSO	0.17	0.24	0.70	0.50	12.82	0.47
LachnospiraceaeND3007group	Not enough instrumental variables						

MR, Mendelian randomization; PE, preeclampsia and eclampsia; SD, standard deviation; RSSobs, observed residual sum of squares; SNP, single nucleotide polymorphism.

Table S6 Instrumental variables used in the MR analysis of the association between ITP and gut microbiota.

Bacterial taxa (outcome)	SNP	Effect allele	Other allele	MAF	Beta	SE	*P-value*	Beta	SE	P-value	SNP_F
Methanobacteria	rs12459122	C	T	0.15	0.30	0.06	1.62177E-06	0.06	0.03	0.10	8113.45
Methanobacteria	rs2432628	C	T	0.66	0.27	0.05	6.36898E-07	0.01	0.02	0.83	11439.82
Methanobacteria	rs5992838	G	A	0.37	-0.27	0.06	1.30752E-06	-0.02	0.02	0.39	12288.27
Methanobacteria	rs71645168	C	T	0.22	-0.30	0.07	8.63257E-06	-0.01	0.03	0.75	10680.57
Methanobacteria	rs73409912	T	C	0.16	-0.40	0.08	7.16143E-07	0.00	0.03	0.97	15237.43
Alcaligenaceae	rs12459122	C	T	0.15	0.30	0.06	1.62177E-06	0.01	0.02	0.43	8113.45
Alcaligenaceae	rs145081815	C	A	0.06	0.49	0.10	2.67609E-06	-0.02	0.04	0.56	9335.97
Alcaligenaceae	rs2432628	C	T	0.66	0.27	0.05	6.36898E-07	0.00	0.01	0.74	11439.82
Alcaligenaceae	rs5992838	G	A	0.37	-0.27	0.06	1.30752E-06	-0.01	0.01	0.57	12288.27
Alcaligenaceae	rs71645168	C	T	0.22	-0.30	0.07	8.63257E-06	0.00	0.01	0.77	10680.57
Alcaligenaceae	rs73409912	T	C	0.16	-0.40	0.08	7.16143E-07	0.03	0.01	0.06	15237.43
Lachnospiraceae	rs12459122	C	T	0.15	0.30	0.06	1.62177E-06	0.02	0.01	0.14	8113.45
Lachnospiraceae	rs145081815	C	A	0.06	0.49	0.10	2.67609E-06	-0.01	0.04	0.67	9335.97
Lachnospiraceae	rs2432628	C	T	0.66	0.27	0.05	6.36898E-07	0.00	0.01	0.67	11439.82
Lachnospiraceae	rs5992838	G	A	0.37	-0.27	0.06	1.30752E-06	0.01	0.01	0.55	12288.27
Lachnospiraceae	rs71645168	C	T	0.22	-0.30	0.07	8.63257E-06	-0.01	0.01	0.54	10680.57
Lachnospiraceae	rs73409912	T	C	0.16	-0.40	0.08	7.16143E-07	-0.02	0.01	0.14	15237.43
Methanobacteriaceae	rs12459122	C	T	0.15	0.30	0.06	1.62177E-06	0.06	0.03	0.10	8113.45
Methanobacteriaceae	rs2432628	C	T	0.66	0.27	0.05	6.36898E-07	0.01	0.02	0.83	11439.82
Methanobacteriaceae	rs5992838	G	A	0.37	-0.27	0.06	1.30752E-06	-0.02	0.02	0.39	12288.27

Methanobacteriaceae	rs71645168	C	T	0.22	-0.30	0.07	8.63257E-06	-0.01	0.03	0.75	10680.57
Methanobacteriaceae	rs73409912	T	C	0.16	-0.40	0.08	7.16143E-07	0.00	0.03	0.97	15237.43
Coprococcus2	rs12459122	C	T	0.15	0.30	0.06	1.62177E-06	0.01	0.02	0.67	8113.45
Coprococcus2	rs145081815	C	A	0.06	0.49	0.10	2.67609E-06	-0.02	0.05	0.82	9335.97
Coprococcus2	rs2432628	C	T	0.66	0.27	0.05	6.36898E-07	0.00	0.01	0.99	11439.82
Coprococcus2	rs5992838	G	A	0.37	-0.27	0.06	1.30752E-06	0.01	0.01	0.46	12288.27
Coprococcus2	rs71645168	C	T	0.22	-0.30	0.07	8.63257E-06	0.01	0.02	0.48	10680.57
Coprococcus2	rs73409912	T	C	0.16	-0.40	0.08	7.16143E-07	-0.01	0.02	0.38	15237.43
Gordonibacter	rs12459122	C	T	0.15	0.30	0.06	1.62177E-06	0.01	0.03	0.82	8113.45
Gordonibacter	rs2432628	C	T	0.66	0.27	0.05	6.36898E-07	0.00	0.02	0.97	11439.82
Gordonibacter	rs5992838	G	A	0.37	-0.27	0.06	1.30752E-06	0.01	0.02	0.68	12288.27
Gordonibacter	rs71645168	C	T	0.22	-0.30	0.07	8.63257E-06	0.01	0.03	0.74	10680.57
Gordonibacter	rs73409912	T	C	0.16	-0.40	0.08	7.16143E-07	-0.02	0.03	0.68	15237.43
Veillonella	rs12459122	C	T	0.15	0.30	0.06	1.62177E-06	-0.01	0.02	0.61	8113.45
Veillonella	rs145081815	C	A	0.06	0.49	0.10	2.67609E-06	-0.09	0.05	0.06	9335.97
Veillonella	rs2432628	C	T	0.66	0.27	0.05	6.36898E-07	0.01	0.02	0.59	11439.82
Veillonella	rs5992838	G	A	0.37	-0.27	0.06	1.30752E-06	0.00	0.02	0.82	12288.27
Veillonella	rs71645168	C	T	0.22	-0.30	0.07	8.63257E-06	0.02	0.02	0.29	10680.57
Veillonella	rs73409912	T	C	0.16	-0.40	0.08	7.16143E-07	0.00	0.02	0.84	15237.43
Bacillales	rs12459122	C	T	0.15	0.30	0.06	1.62177E-06	0.00	0.04	0.98	8113.45
Bacillales	rs2432628	C	T	0.66	0.27	0.05	6.36898E-07	-0.02	0.03	0.54	11439.82
Bacillales	rs5992838	G	A	0.37	-0.27	0.06	1.30752E-06	0.01	0.03	0.62	12288.27
Bacillales	rs71645168	C	T	0.22	-0.30	0.07	8.63257E-06	0.04	0.03	0.15	10680.57
Bacillales	rs73409912	T	C	0.16	-0.40	0.08	7.16143E-07	-0.02	0.03	0.72	15237.43
Methanobacteriales	rs12459122	C	T	0.15	0.30	0.06	1.62177E-06	0.06	0.03	0.10	8113.45
Methanobacteriales	rs2432628	C	T	0.66	0.27	0.05	6.36898E-07	0.01	0.02	0.83	11439.82

Methanobacteriales	rs5992838	G	A	0.37	-0.27	0.06	1.30752E-06	-0.02	0.02	0.39	12288.27
Methanobacteriales	rs71645168	C	T	0.22	-0.30	0.07	8.63257E-06	-0.01	0.03	0.75	10680.57
Methanobacteriales	rs73409912	T	C	0.16	-0.40	0.08	7.16143E-07	0.00	0.03	0.97	15237.43
Actinobacteria	rs12459122	C	T	0.15	0.30	0.06	1.62177E-06	0.03	0.01	0.07	8113.45
Actinobacteria	rs145081815	C	A	0.06	0.49	0.10	2.67609E-06	0.04	0.04	0.29	9335.97
Actinobacteria	rs2432628	C	T	0.66	0.27	0.05	6.36898E-07	0.00	0.01	0.77	11439.82
Actinobacteria	rs5992838	G	A	0.37	-0.27	0.06	1.30752E-06	0.00	0.01	0.68	12288.27
Actinobacteria	rs71645168	C	T	0.22	-0.30	0.07	8.63257E-06	0.00	0.01	0.96	10680.57
Actinobacteria	rs73409912	T	C	0.16	-0.40	0.08	7.16143E-07	0.00	0.01	0.95	15237.43

MR, Mendelian randomization; ITP, Immune thrombocytopenia; SNP, single nucleotide polymorphism; MAF, minor allele frequency; SE, standard error.

Table S7 Full result of MR estimates for the association between ITP and gut microbiota.

Bacterial taxa (outcome)	MR method	No.of SNP	OR	OR 95%CI(L)	OR 95%CI(U)	*P value*
Methanobacteria	Inverse variance weighted	5	1.05	0.97	1.14	0.23
Methanobacteria	Weighted median	5	1.02	0.93	1.12	0.69
Methanobacteria	MR Egger	5	0.93	0.53	1.63	0.81
Alcaligenaceae	MR Egger	6	0.83	0.66	1.04	0.18
Alcaligenaceae	Inverse variance weighted	6	0.99	0.95	1.02	0.47
Alcaligenaceae	Weighted median	6	0.99	0.94	1.04	0.65
Lachnospiraceae	MR Egger	6	1.14	0.92	1.42	0.29
Lachnospiraceae	Inverse variance weighted	6	1.02	0.98	1.05	0.37
Lachnospiraceae	Weighted median	6	1.01	0.97	1.06	0.55
Methanobacteriaceae	Inverse variance weighted	5	1.05	0.97	1.14	0.23
Methanobacteriaceae	Weighted median	5	1.02	0.92	1.12	0.69
Methanobacteriaceae	MR Egger	5	0.93	0.53	1.63	0.81
Coprococcus2	MR Egger	6	1.09	0.84	1.43	0.55
Coprococcus2	Weighted median	6	0.99	0.94	1.05	0.85
Coprococcus2	Inverse variance weighted	6	1.00	0.95	1.04	0.87
Gordonibacter	MR Egger	5	1.19	0.68	2.08	0.59
Gordonibacter	Inverse variance weighted	5	1.00	0.92	1.08	0.98
Gordonibacter	Weighted median	5	1.00	0.91	1.10	0.99
Veillonella	MR Egger	6	0.82	0.61	1.11	0.27
Veillonella	Inverse variance weighted	6	0.98	0.93	1.03	0.42
Veillonella	Weighted median	6	0.99	0.93	1.06	0.83
Bacillales	Inverse variance weighted	5	0.96	0.88	1.05	0.36

Bacillales	Weighted median	5	0.96	0.86	1.07	0.42
Bacillales	MR Egger	5	1.31	0.71	2.39	0.45
Methanobacteriales	Inverse variance weighted	5	1.05	0.97	1.14	0.23
Methanobacteriales	Weighted median	5	1.02	0.92	1.13	0.70
Methanobacteriales	MR Egger	5	0.93	0.53	1.63	0.81
Actinobacteria	Inverse variance weighted	6	1.02	0.98	1.05	0.40
Actinobacteria	MR Egger	6	1.07	0.86	1.34	0.57
Actinobacteria	Weighted median	6	1.00	0.96	1.05	0.91

MR, Mendelian randomization; ITP, Immune thrombocytopenia; SNP, single nucleotide polymorphism; OR, odds ratio; CI, confidence interval; L, Lower limitation; U.Upper limitation.

Table S8 The heterogeneity of gut microbiota instrumental variables.

**Bacterial taxa (outcome)**	**Method**	**Q**	**df**	***P value***
Methanobacteria	Inverse variance weighted	2.09	4.00	0.72
Alcaligenaceae	Inverse variance weighted	4.00	5.00	0.55
Lachnospiraceae	Inverse variance weighted	4.13	5.00	0.53
Methanobacteriaceae	Inverse variance weighted	2.09	4.00	0.72
Coprococcus2	Inverse variance weighted	1.94	5.00	0.86
Gordonibacter	Inverse variance weighted	0.65	4.00	0.96
Veillonella	Inverse variance weighted	4.16	5.00	0.53
Bacillales	Inverse variance weighted	2.26	4.00	0.69
Methanobacteriales	Inverse variance weighted	2.09	4.00	0.72
Actinobacteria	Inverse variance weighted	3.95	5.00	0.56

Df, degree of freedom.

Table S9 Directional horizontal pleiotropy assessed by intercept term in MR Egger regression of the association between ITP and gut microbiota.

**Bacterial taxa (outcome)**	**Egger_intercept**	**SE**	***P-value***
Methanobacteria	0.04	0.09	0.70
Alcaligenaceae	0.05	0.04	0.20
Lachnospiraceae	-0.04	0.03	0.34
Methanobacteriaceae	0.04	0.09	0.70
Coprococcus2	-0.03	0.04	0.53
Gordonibacter	-0.05	0.09	0.59
Veillonella	0.05	0.05	0.31
Bacillales	-0.09	0.09	0.39
Methanobacteriales	0.04	0.09	0.70
Actinobacteria	-0.02	0.03	0.65

MR, Mendelian randomization; ITP, Immune thrombocytopenia; SE, standard error.

Table S10 MR-PRESSO analysis for the association between ITP and gut microbiota.

**Bacterial taxa (outcome)**	**MR Analysis**	**Causal Estimate**	**SD**	**T**	***P-value***	**RSSobs**	**Global test *P*-value**
Methanobacteria	MR-PRESSO	0.05	0.03	1.67	0.17	3.00	0.76
Methanobacteriaceae	MR-PRESSO	0.05	0.03	1.67	0.17	3.00	0.75
Methanobacteriales	MR-PRESSO	0.05	0.03	1.67	0.17	3.00	0.76
Bacillales	MR-PRESSO	-0.04	0.03	-1.22	0.29	3.68	0.66
Veillonella	MR-PRESSO	-0.02	0.02	-0.88	0.42	5.38	0.60
Lachnospiraceae	MR-PRESSO	0.02	0.02	1.00	0.36	6.33	0.52
Actinobacteria	MR-PRESSO	0.02	0.02	0.95	0.38	5.34	0.60
Alcaligenaceae	MR-PRESSO	-0.01	0.02	-0.81	0.45	6.34	0.53
Coprococcus2	MR-PRESSO	0.00	0.01	-0.26	0.80	3.05	0.83
Gordonibacter	MR-PRESSO	0.00	0.02	-0.07	0.95	1.08	0.95

MR, Mendelian randomization; ITP, Immune thrombocytopenia; SD, standard deviation; RSSobs, observed residual sum of squares.

Table S11 Estimation of the Steiger direction test from 211 taxa to ITP.

Exposure	SNP_r2. exposure	SNP_r2. outcome	Direction	Steiger *p*
Methanobacteria	0.0106	4.28002E-05	TRUE	1.91375E-37
Alcaligenaceae	0.0133	4.96423E-05	TRUE	1.45342E-46
Lachnospiraceae	0.0185	6.20808E-05	TRUE	6.92251E-65
Methanobacteriaceae	0.0106	4.28002E-05	TRUE	1.91375E-37
Coprococcus2	0.0091	2.3195E-05	TRUE	4.24356E-33
Gordonibacter	0.0126	2.82383E-05	TRUE	2.01259E-45
Veillonella	0.0058	2.01196E-05	TRUE	2.6948E-21
Bacillales	0.0091	3.54727E-05	TRUE	2.34274E-32
Methanobacteriales	0.0106	4.28002E-05	TRUE	1.91375E-37
Actinobacteria	0.0184	6.45619E-05	TRUE	1.8451E-64

Table S12 Lead SNPs

uniq ID	chr	pos
1:4210455:A:G	1	4210455
1:61827534:A:G	1	61827534
1:83136096:A:G	1	83136096
1:112139008:C:T	1	112139008
1:117031337:G:T	1	117031337
1:178945043:A:G	1	178945043
1:210628449:G:T	1	210628449
1:214031969:A:C	1	214031969
1:223688236:A:C	1	223688236
2:30158494:A:C	2	30158494
2:102249583:C:T	2	102249583
2:125440268:G:T	2	125440268
2:135837906:A:C	2	135837906
2:136822223:C:T	2	136822223
2:136873549:A:T	2	136873549
2:213756191:C:T	2	213756191
2:220362557:C:T	2	220362557
2:232469722:A:G	2	232469722
2:239900775:A:G	2	239900775
2:239936384:C:T	2	239936384
3:2086167:A:G	3	2086167
3:15053083:C:T	3	15053083
3:32362998:C:T	3	32362998
3:70588939:C:T	3	70588939
3:125544032:A:T	3	125544032
3:180544880:C:T	3	180544880
3:187450354:A:T	3	187450354
3:193456274:C:G	3	193456274
4:1517826:A:G	4	1517826
4:8142607:A:C	4	8142607
4:10245354:A:G	4	10245354
4:45642485:C:T	4	45642485
4:75867283:A:T	4	75867283
4:83635863:A:G	4	83635863
4:104418307:A:C	4	104418307
4:127766709:A:G	4	127766709
4:131125786:C:T	4	131125786
4:136768501:A:C	4	136768501
4:186906801:C:T	4	186906801
5:32348593:C:T	5	32348593
5:33114638:C:T	5	33114638
5:34794789:A:G	5	34794789

5:78856074:A:T	5	78856074
5:90471451:A:G	5	90471451
5:104233461:A:G	5	104233461
5:119914285:C:T	5	119914285
5:173497796:C:T	5	173497796
5:178160975:A:G	5	178160975
6:14617591:C:T	6	14617591
6:46664437:C:T	6	46664437
6:92775210:C:T	6	92775210
6:113129609:C:T	6	113129609
6:138305038:A:T	6	138305038
6:161957425:C:G	6	161957425
7:10773932:G:T	7	10773932
7:18970607:A:G	7	18970607
7:24464799:A:T	7	24464799
7:47144505:A:G	7	47144505
7:49423832:A:G	7	49423832
7:104214487:A:C	7	104214487
7:111294074:C:T	7	111294074
7:117496144:C:T	7	117496144
7:129433205:C:G	7	129433205
7:130436459:C:T	7	130436459
7:136937189:A:G	7	136937189
7:155002747:C:T	7	155002747
8:13253770:C:G	8	13253770
9:16806694:C:G	9	16806694
9:25554068:C:T	9	25554068
9:72179847:C:T	9	72179847
9:82485327:G:T	9	82485327
9:88787144:A:G	9	88787144
9:89306305:A:G	9	89306305
9:100113683:C:T	9	100113683
9:133791605:C:T	9	133791605
10:103392503:C:T	10	103392503
10:129715759:A:G	10	129715759
11:2956783:A:G	11	2956783
11:20763367:G:T	11	20763367
11:38085711:A:G	11	38085711
11:44302154:C:T	11	44302154
11:89349287:A:T	11	89349287
12:1396151:C:G	12	1396151
12:20378911:C:G	12	20378911
12:21338861:C:T	12	21338861
12:29024534:C:T	12	29024534

12:43701132:C:G	12	43701132
12:69332975:A:G	12	69332975
12:129984555:C:G	12	129984555
12:131024079:C:T	12	131024079
13:38004672:G:T	13	38004672
13:41956181:A:G	13	41956181
13:58545246:A:C	13	58545246
14:23847194:A:G	14	23847194
14:49001226:A:G	14	49001226
14:66593668:A:T	14	66593668
14:86833255:A:T	14	86833255
14:106939916:A:C	14	106939916
15:71522509:C:T	15	71522509
15:92467422:C:T	15	92467422
16:76559184:A:G	16	76559184
16:81776768:A:G	16	81776768
16:85945839:C:T	16	85945839
16:89248299:A:T	16	89248299
17:10148653:A:T	17	10148653
17:52957210:A:G	17	52957210
17:80469064:A:G	17	80469064
18:26569901:A:G	18	26569901
19:5300465:A:G	19	5300465
19:13425799:C:T	19	13425799
19:14970441:C:T	19	14970441
19:28827760:C:T	19	28827760
19:29408858:C:T	19	29408858
19:46439321:A:G	19	46439321
20:5826474:A:G	20	5826474
20:29836081:C:G	20	29836081
20:30596130:G:T	20	30596130
20:36876868:A:G	20	36876868
20:49266770:A:T	20	49266770
21:28684912:C:G	21	28684912
21:30010673:A:G	21	30010673
22:45604800:A:G	22	45604800

Table S13 IVW results of MR estimates for the association between gut microbiota and ITP.

Bacterial taxa (exposure)	*P* value	FDR
Acidaminococcaceae	0.904	0.963
phylum-Actinobacteria	0.039	0.350
class-Actinobacteria	0.085	0.681
Actinomyces	0.574	0.907
Actinomycetaceae	0.643	0.963
Actinomycetales	0.641	0.916
Adlercreutzia	0.046	0.907
Akkermansia	0.737	0.907
Alcaligenaceae	0.007	0.073
Alistipes	0.418	0.907
Allisonella	0.014	0.907
Alloprevotella	0.170	0.907
Alphaproteobacteria	0.911	0.972
Anaerofilum	0.250	0.907
Anaerostipes	0.392	0.907
Anaerotruncus	0.623	0.907
Bacillales	0.010	0.102
Bacilli	0.818	0.972
Bacteroidaceae	0.364	0.944
Bacteroidales	0.167	0.916
BacteroidalesS247group	0.006	0.073
Bacteroides	0.364	0.907
Bacteroidetes	0.353	0.615
Bacteroidia	0.167	0.685
Barnesiella	0.597	0.907
Betaproteobacteria	0.871	0.972
Bifidobacteriaceae	0.501	0.963
Bifidobacteriales	0.501	0.916
Bifidobacterium	0.094	0.907
Bilophila	0.680	0.907
Blautia	0.244	0.907
Burkholderiales	0.292	0.916
Butyricicoccus	0.414	0.907
Butyricimonas	0.766	0.907
Butyrivibrio	0.823	0.907
CandidatusSoleaferrea	0.157	0.907
Catenibacterium	0.213	0.907
Christensenellaceae	0.242	0.857
ChristensenellaceaeR7group	0.162	0.907
Clostridia	0.646	0.972
Clostridiaceae1	0.864	0.963

Clostridiales	0.604	0.916
ClostridialesvadinBB60group	0.752	0.963
Clostridiuminnocuumgroup	0.760	0.907
Clostridiumsensustricto1	0.526	0.907
Collinsella	0.494	0.907
Coprobacter	0.247	0.907
Coprococcus1	0.458	0.907
Coprococcus2	0.037	0.907
Coprococcus3	0.140	0.907
Coriobacteriaceae	0.988	0.988
Coriobacteriales	0.988	0.988
Coriobacteriia	0.988	0.988
Cyanobacteria	0.099	0.447
Defluviitaleaceae	0.886	0.963
DefluviitaleaceaeUCG011	0.509	0.907
Deltaproteobacteria	0.558	0.972
Desulfovibrio	0.272	0.907
Desulfovibrionaceae	0.823	0.963
Desulfovibrionales	0.515	0.916
Dialister	0.590	0.907
Dorea	0.616	0.907
Eggerthella	0.749	0.907
Eisenbergiella	0.786	0.907
Enterobacteriaceae	0.391	0.944
Enterobacteriales	0.391	0.916
Enterorhabdus	0.736	0.907
Erysipelatoclostridium	0.411	0.907
Erysipelotrichaceae	0.214	0.857
ErysipelotrichaceaeUCG003	0.548	0.907
Erysipelotrichales	0.214	0.916
Erysipelotrichia	0.214	0.685
EscherichiaShigella	0.674	0.907
Eubacteriumbrachygroup	0.100	0.907
Eubacteriumcoprostanoligenesgroup	0.141	0.907
Eubacteriumeligensgroup	0.751	0.907
Eubacteriumfissicatenagroup	0.814	0.907
Eubacteriumhalliigroup	0.078	0.907
Eubacteriumnodatumgroup	0.787	0.907
Eubacteriumoxidoreducensgroup	0.669	0.907
Eubacteriumrectalegroup	0.922	0.959
Eubacteriumruminantiumgroup	0.043	0.907
Eubacteriumventriosumgroup	0.939	0.963
Eubacteriumxylanophilumgroup	0.793	0.907
Euryarchaeota	0.572	0.615

Faecalibacterium	0.674	0.907
FamilyXI	0.195	0.857
FamilyXIII	0.778	0.963
FamilyXIIIAD3011group	0.733	0.907
FamilyXIIIUCG001	0.703	0.907
Firmicutes	0.615	0.615
Flavonifractor	0.352	0.907
Fusicatenibacter	0.823	0.907
Gammaproteobacteria	0.530	0.972
Gastranaerophilales	0.401	0.916
Gordonibacter	0.044	0.907
Haemophilus	0.155	0.907
Holdemanella	0.977	0.986
Holdemania	0.651	0.907
Howardella	0.356	0.907
Hungatella	0.211	0.907
Intestinibacter	0.344	0.907
Intestinimonas	0.096	0.907
Lachnoclostridium	0.352	0.907
Lachnospira	0.362	0.907
Lachnospiraceae	0.038	0.304
LachnospiraceaeFCS020group	0.608	0.907
LachnospiraceaeNC2004group	0.539	0.907
LachnospiraceaeND3007group	0.237	0.907
LachnospiraceaeNK4A136group	0.871	0.926
LachnospiraceaeUCG001	0.304	0.907
LachnospiraceaeUCG004	0.547	0.907
LachnospiraceaeUCG008	0.859	0.921
LachnospiraceaeUCG010	0.219	0.907
Lactobacillaceae	0.769	0.963
Lactobacillales	0.943	0.988
Lactobacillus	0.505	0.907
Lactococcus	0.619	0.907
Lentisphaerae	0.521	0.615
Lentisphaeria	0.479	0.972
Marvinbryantia	0.548	0.907
Melainabacteria	0.214	0.685
Methanobacteria	0.007	0.110
Methanobacteriaceae	0.007	0.073
Methanobacteriales	0.007	0.102
Methanobrevibacter	0.443	0.907
Mollicutes	0.559	0.972
MollicutesRF9	0.714	0.918
NB1n	0.374	0.916

Negativicutes	0.898	0.972
Odoribacter	0.770	0.907
Olsenella	0.322	0.907
Oscillibacter	0.846	0.919
Oscillospira	0.761	0.907
Oxalobacter	0.997	0.997
Oxalobacteraceae	0.074	0.473
Parabacteroides	0.491	0.907
Paraprevotella	0.810	0.907
Parasutterella	0.433	0.907
Pasteurellaceae	0.599	0.963
Pasteurellales	0.599	0.916
Peptococcaceae	0.413	0.944
Peptococcus	0.633	0.907
Peptostreptococcaceae	0.646	0.963
Phascolarctobacterium	0.715	0.907
Porphyromonadaceae	0.933	0.963
Prevotella7	0.595	0.907
Prevotella9	0.802	0.907
Prevotellaceae	0.295	0.857
Proteobacteria	0.582	0.615
Rhodospirillaceae	0.244	0.857
Rhodospirillales	0.926	0.988
Rikenellaceae	0.563	0.963
RikenellaceaeRC9gutgroup	0.850	0.919
Romboutsia	0.780	0.907
Roseburia	0.889	0.936
Ruminiclostridium5	0.967	0.984
Ruminiclostridium6	0.712	0.907
Ruminiclostridium9	0.404	0.907
Ruminococcaceae	0.757	0.963
RuminococcaceaeNK4A214group	0.927	0.959
RuminococcaceaeUCG002	0.797	0.907
RuminococcaceaeUCG003	0.106	0.907
RuminococcaceaeUCG004	0.585	0.907
RuminococcaceaeUCG005	0.546	0.907
RuminococcaceaeUCG009	0.259	0.907
RuminococcaceaeUCG010	0.273	0.907
RuminococcaceaeUCG011	0.112	0.907
RuminococcaceaeUCG013	0.369	0.907
RuminococcaceaeUCG014	0.593	0.907
Ruminococcus1	0.800	0.907
Ruminococcus2	0.193	0.907
Ruminococcusgauvreauiigroup	0.195	0.907

Ruminococcusgnavusgroup	0.464	0.907
Ruminococcustorquesgroup	0.753	0.907
Selenomonadales	0.898	0.988
Sellimonas	0.473	0.907
Senegalimassilia	0.760	0.907
Slackia	0.699	0.907
Streptococcaceae	0.280	0.857
Streptococcus	0.418	0.907
Subdoligranulum	0.669	0.907
Sutterella	0.503	0.907
Tenericutes	0.559	0.615
Terrisporobacter	0.245	0.907
Turicibacter	0.529	0.907
Tyzzerella3	0.474	0.907
unknownfamily	0.374	0.859
unknownfamily	0.401	0.859
unknownfamily	0.714	0.940
unknowngenus	0.006	0.092
unknowngenus	0.260	0.859
unknowngenus	0.332	0.859
unknowngenus	0.374	0.859
unknowngenus	0.401	0.859
unknowngenus	0.512	0.940
unknowngenus	0.693	0.940
unknowngenus	0.714	0.940
unknowngenus	0.752	0.940
unknowngenus	0.865	0.948
unknowngenus	0.886	0.948
unknowngenus	0.948	0.948
Veillonella	0.040	0.907
Veillonellaceae	0.561	0.963
Verrucomicrobia	0.487	0.615
Verrucomicrobiaceae	0.735	0.963
Verrucomicrobiae	0.734	0.972
Verrucomicrobiales	0.734	0.918
Victivallaceae	0.832	0.963
Victivallales	0.479	0.916
Victivallis	0.592	0.907

MR, Mendelian randomization; ITP, Immune thrombocytopenia; IVW，Inverse variance weighted; FDR, false discovery rate

Table S14 Results of MVMR under each attribute of gut microbiota.

Attribute	Bacterial taxa (exposure)	No.of SNP	Beta	Se	OR	OR_95%CI(L)	OR_95%CI(U)	*P* value
Genus	*Gordonibacter*	11	0.307	0.106	1.360	1.104	1.674	0.004
	Coprococcus2	7	-0.596	0.241	0.551	0.343	0.885	0.014
	Veillonella	5	0.537	0.248	1.711	1.053	2.780	0.030
Family	*Lachnospiraceae*	14	-0.520	0.321	0.594	0.317	1.116	0.106
	Alcaligenaceae	10	0.862	0.346	2.369	1.202	4.669	0.013
	Methanobacteriaceae	9	-0.311	0.170	0.733	0.525	1.022	0.067
Order	*Methanobacteriales*	7	-0.369	0.189	0.691	0.477	1.002	0.051
	Bacillales	8	-0.428	0.161	0.652	0.475	0.894	0.008
